# C2-Symmetrical Terphenyl Derivatives as Small Molecule Inhibitors of Programmed Cell Death 1/Programmed Death Ligand 1 Protein–Protein Interaction

**DOI:** 10.3390/molecules29112646

**Published:** 2024-06-04

**Authors:** Joanna Klimek, Oskar Kruc, Joanna Ceklarz, Beata Kamińska, Bogdan Musielak, Robin van der Straat, Alexander Dӧmling, Tad A. Holak, Damian Muszak, Justyna Kalinowska-Tłuścik, Łukasz Skalniak, Ewa Surmiak

**Affiliations:** 1 Department of Organic Chemistry, Faculty of Chemistry, Jagiellonian University, Gronostajowa St. 2, 30-387 Cracow, Poland; joanna.klimek@doctoral.uj.edu.pl (J.K.); oskar.kruc@doctoral.uj.edu.pl (O.K.); beata.kaminska@doctoral.uj.edu.pl (B.K.); bogdan.musielak@uj.edu.pl (B.M.); tadholak@uj.edu.pl (T.A.H.); damian.muszak@uj.edu.pl (D.M.); justyna.kalinowska-tluscik@uj.edu.pl (J.K.-T.); 2 Doctoral School of Exact and Natural Sciences, Jagiellonian University, Prof. St. Łojasiewicza St. 11, 30-348 Cracow, Poland; 3 Department of Drug Design, University of Groningen, 9713 AV Groningen, The Netherlands; r.van.der.straat@rug.nl; 4 Institute of Molecular and Translational Medicine, Faculty of Medicine and Dentistry and Czech Advanced Technology and Research Institute, Palackӯ University in Olomouc, Křížkovského 511/8, 779 00 Olomouc, Czech Republic; alexander.domling@upol.cz

**Keywords:** PD-L1, immune checkpoint, small molecule inhibitor, cancer, C2-symmetrical ligands

## Abstract

The PD-1/PD-L1 complex is an immune checkpoint responsible for regulating the natural immune response, but also allows tumors to escape immune surveillance. Inhibition of the PD-1/PD-L1 axis positively contributes to the efficacy of cancer treatment. The only available therapeutics targeting PD-1/PD-L1 are monoclonal antibody-based drugs, which have several limitations. Therefore, small molecule compounds are emerging as an attractive alternative that can potentially overcome the drawbacks of mAb-based therapy. In this article, we present a novel class of small molecule compounds based on the terphenyl scaffold that bind to PD-L1. The general architecture of the presented structures is characterized by axial symmetry and consists of three elements: an m-terphenyl core, an additional aromatic ring, and a solubilizing agent. Using molecular docking, we designed a series of final compounds, which were subsequently synthesized and tested in HTRF assay and NMR binding assay to evaluate their activity. In addition, we performed an in-depth analysis of the mutual arrangement of the phenyl rings of the terphenyl core within the binding pocket of PD-L1 and found several correlations between the plane angle values and the affinity of the compounds towards the protein.

## 1. Introduction

Cancer is a major disease affecting the human population in modern society and is the second leading cause of death worldwide. According to the American Cancer Society’s October 2023 projections, there will be nearly 2,000,000 new cases of cancer and more than 600,000 deaths from cancer in the United States by the end of 2024 [1].

Despite a deeper understanding of cancer pathogenesis which led to the development of several potent therapeutics, the search for new and effective treatment is still necessary. Recently, a particularly promising strategy for an effective anti-cancer fight, called immune checkpoint blockade therapy (ICBT), has emerged. It is a groundbreaking approach leading to the stimulation and strengthening of the human immune system’s response against tumor cells. Amongst many different ICBTs, the blockade of the PD-1/PD-L1 immune checkpoint (IC) pathway became one of the most attractive strategies [2,3,4,5].

Programmed death 1(PD-1, CD279) is a monomeric, type I transmembrane glycoprotein that belongs to the B7-CD28 immunoreceptor family. It is expressed on the surfaces of various cells, e.g., T cells, B cells, monocytes, and more [6,7,8,9]. The natural ligands of PD-1 receptor include programmed death ligand 1 (PD-L1, B7-H1, CD274) and programmed death ligand 2 (PD-L2, B7-DC, CD273). Expression of these proteins has been proved not only in many different types of human healthy cells but also in malignant cells, e.g., melanoma, lung, or kidney cancer [10]. Binding of PD-1 to one of its natural ligands results in suppression of T-cell activation and proliferation, leading in consequence to decreased immune system response. In physiological conditions, the PD-1/PD-L1 axis is responsible for maintaining self-antigen tolerance, preventing autoimmune disease development, and reducing healthy tissue damage during infection. However, engagement of the PD-1 receptor by cancer cells allows them to escape from immune surveillance by negative regulation of T cells. The preservation of T-cell activity and functionality is achieved by the interruption of the PD-1/PD-L1 binding. Consequently, the therapeutic strategy based on targeting the PD-1/PD-L1 axis with inhibitors specific to either protein holds significant promise for advancing the efficacy of tumor treatment [8,11,12].

In recent years, the Food and Drug Administration (FDA) approved seven monoclonal-antibody-based (mAb) pharmaceutics for clinical use, which are capable of effectively binding to PD-1 or PD-L1 [3,13,14,15]. However, using mAb-based therapy might result in dangerous immune-related adverse events (irAE) and could affect the immune tolerance system in healthy tissues [16]. Moreover, an increase in antibody size tends to decrease tumor penetration. Another issue is also the complex production process which causes high mAb treatment prices [17]. Therefore, small molecule inhibitors (SMIs) have become a promising alternative to currently available immunoglobulin-based products since they have better oral bioavailability, may penetrate the tumor surface more effectively, and are characterized by faster elimination from the body [18].

Nevertheless, the development of successful SMIs of the PD-1/PD-L1 interaction still remains a significant challenge, mostly due to the large protein–protein interaction area to be covered (1970 Å^2^) and the relatively flat binding surface (no ligand-binding cavity defined) [19]. The first major advancement in the PD-1/PD-L1 small molecule inhibitors design has been made by Bristol-Myers Squibb Co. (BMS, New York, NY, USA) [20,21]. This company patented, among others, biphenyl-based structures, with defined inhibitory activity in the PD-1/PD-L1 complex formation. The determined IC_50_ values for these compounds varied in a wide range from ~0.6 nM to ~200 nM. In vitro studies confirmed the ability of BMS molecules to inhibit the protein–ligand complex formation. Additionally, the molecular mechanism of their action was determined, which usually involves PD-L1 homodimerization [22]. This was confirmed by X-ray crystal structure analyses of several biaryl-based small molecules/PD-L1 dimer co-crystals [23,24,25]. The observed twofold symmetry of the homodimer became the basis for the design of the first symmetrical inhibitors. The study conducted by Basu et al. showed that symmetric modification of BMS-202 centered on 2,2′-dimethyl-1,1′-biphenyl core was nearly four times more potent than the original precursor [26]. A similar correlation was also given by Kawashita et al. where an improvement in activity against PD-1/PD-L1 complex formation was observed for the symmetrized BMS-1327 structure [27]. Subsequently, a standout in the field of SMIs became Compound A (ARB-272572, Arbutus Biopharma Co., Vancouver, BC, Canada), featuring a symmetrical design built on a biphenyl core. It demonstrated distinctive activity both in vivo and in vitro [28].

Since the first successful announcement, several postulated chemotypes have been presented by BMS Co., Incyte Co. (Wilmington, DE, USA), Arbutus Biopharma Co., and many other companies. These chemotypes can be classified according to similarities in their scaffolds. Among the proposed structures, the majority are based on the biphenyl core, which manifests in various iterations including for example short, elongated, or symmetrical compounds. Another significant group consists of molecules containing the terphenyl and quaterphenyl motifs, also in different variations [29]. Despite the extensive diversity of PD-L1 inhibitors only a few, such us compounds: CA-170 [30], GS-4224 [31], INCB086550 [32,33] and MAX-10181 [34] entered clinical trials phase I and II for the treatment of advanced tumors and lymphomas [35]. 

Encouraged by the promising bioactivity of short terphenyl molecules described previously by us [36,37], and others [38,39] we decided to explore further the terphenyl scaffold. Considering the high affinity towards PD-L1 demonstrated by symmetric Compound A, as well as the general trend observed for the longer, symmetric inhibitors, we designed a series of C2-symmetrical terphenyl compounds SMIs that support further investigation on the terphenyl derivatives symmetry impact on binding modes to the PD-L1 protein. The selected structures contained a terphenyl core decorated with methyl or chlorine substituents in different positions of the aromatic ring. The distal terphenyl rings are substituted in ortho, meta, or para positions with additional aromatic rings and solubilizing fragments on both ends. With such a structural model, we wanted to study the effects of symmetry and substitution patterns on the compound’s activity and its improvement in comparison to the existing short structures. For this purpose, we developed a new class of C2-symmetric *m*-terphenyl-based compounds that are expected to be effective inhibitors of the PD-1/PD-L1 pathway. The investigation was supported by the molecular docking study of the designed structures to the binding pocket of the PD-L1 homodimer. Meanwhile, we also conducted synthesis, followed by activity evaluation HTRF assay, resulting in IC_50_ values estimation and NMR binding confirmation. To find the explanation of the obtained results at the molecular level, an in-depth analysis of the mutual arrangement of terphenyl core phenyl rings inside the binding pocket was conducted. This study revealed compelling correlations between the geometrical structure and the biological activity of the molecules.

## 2. Results and Discussion

### 2.1. Chemical Synthesis of C2-Symmetrical Inhibitors of PD-1/PD-L1 Interactions 

**Figure 1:**
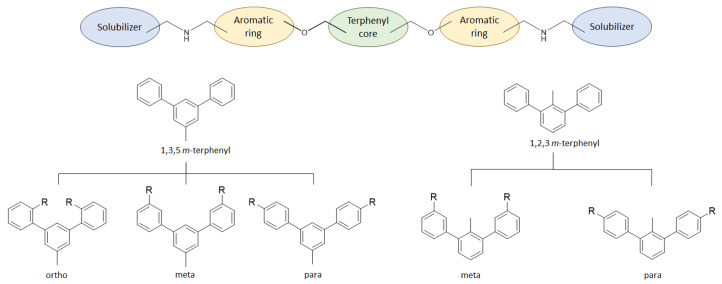
Schematic arrangement of the designed series of the m-terphenyl derivatives. The structures were based on 1,3,5- or 1,2,3-*m*-terphenyl cores. Within each series, compounds were identified featuring substitutions at the meta, para, or ortho positions.

The general scaffold of the compounds studied here consisted of three elements: *m*-terphenyl core, symmetrically elongated by an additional aromatic ring linked by an ether bond and terminated with solubilizer in the form of ethanolamine or N-(2-aminoethyl)acetamide (Figure 1).

We synthesized two series of compounds based on 1,3,5-substituted *m*-terphenyl and 1,2,3-substituted *m*-terphenyl. Within both series, compounds can be categorized based on the ortho, meta, and para position of the additional (non-terphenyl) aromatic ring (R-substituent) towards the central core.

**Figure 2:**
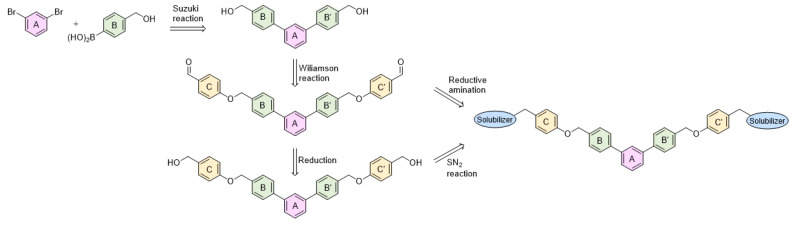
A general overview of the synthesis pathway leading to the final compounds. The individual aromatic rings in the compound were labeled with corresponding colors: the central m-terphenyl ring in pink (A), the distal m-terphenyl rings in green (B and B’), and additional terminal rings in yellow (C and C’).

To achieve the desired final product, a synthetic approach involving 3 or 4 stages was applied *(*Figure 2). The initial steps of the synthesis were similar for all compounds and based on the cross-coupling reactions. Full synthetic schemes (Schemes S1–S4) and conditions for all intermediate compounds together with structures of the final compounds (Table S1) are available in the Supporting Information.

Depending on the series, the starting substrates were commercially available 3,5-dibromotoluene and 2,6-dibromotoluene, which in the Suzuki reaction with corresponding borane, gave the *m*-terphenyl core substituted in *1,3,5*- (**1a**–**1e**) or *1,2,3*-position (**1f**–**1i**, **1k**). Compound (**1j**) was prepared using the same methodology, although 1,3-dibromo-2-chlorobenzene was applied as a substrate, which resulted in a chlorine atom in the central ring of the *m*-terphenyl core. Subsequently, prepared intermediates were used in the Williamson reaction to result in a group of alcohols (**3a, 3c**) and aldehydes (**2a**–**2o**). In the last step, the reductive amination with NaBH_3_CN was used to convert aldehydes (**2b, 2e, 2f, 2k, 2l**) into final products terminated with N-(2-aminoethyl)acetamide (**4c, 5a, 6a, 7j, 7k**) and ethanolamine (**7l**). In the case of compounds (**2a**, **2c**, **2d**, **2g**–**2j**, **2m**–**2o**), reduction with LiBH_4_ was first performed to provide alcohols (**3b**, **3d**, **3e–3l**). Then, isolated compounds (**3a–3l**) were taken into reaction with SOCl_2_, followed by nucleophilic substitution with the appropriate amine. Consequently, a series of final compounds containing N-(2-aminoethyl)acetamide (**4a, 4b, 4d–4f, 5a, 6a, 7a, 7c, 7e, 7h, 7j, 7k, 7m, 8b**) and ethanolamine (**7b, 7d, 7f, 7i, 7l, 7n, 8a, 8c**) was obtained. Structure (**7g**) was synthesized by the conversion of intermediate (**3h**) into corresponding chloride by SOCl_3_, followed by SN_2_ reaction with serinol.

### 2.2. HTRF-Based Structure–Activity Relationship (SAR) Results and Correlations

All synthesized molecules were tested in the homogeneous time-resolved fluorescence (HTRF) assay to assess their activity as inhibitors of the PD-1/PD-L1 complex (Table 1, Table 2 and Table 3). This evaluation was conducted in scouting modes, employing concentrations of 5 μM and 0.5 μM. Based on the obtained measurements, the IC_50_ values were estimated for each tested compound.

The results of the HTRF assay show that the mere substitution of presented *m*-terphenyls in the *1*,*3*,*5* or *1*,*2*,*3* position does not affect the compound’s activity significantly. However, modifications within the molecular core contribute to the observed activity differences. Generally introducing halogen substituent into the main terphenyl core positively influenced molecular affinity, which is consistent with previously observed trends [36,40]. In the 1,3,5-*m*-terphenyl series, adding two fluorine atoms to the molecular core (compound **4d**) improved the compound’s activity (IC_50_ = 2.08 μM) compared to analogs lacking halogens (compounds **4a**, **4b**, **4c**, **4e**, **4f**).

Similarly, within the 1,2,3-*m*-terphenyl in para-substituted series, the incorporation of a chlorine atom into the central terphenyl ring results in the derivative **7m**, which demonstrated enhanced activity, reaching IC_50_ = 0.69 µM. Nevertheless, a structural alteration in this molecule, involving the substitution of the solubilizer with ethanolamine (compound **7n**), resulted in a diminished level of activity (IC_50_ = 2.04 µM). It was found that additional methoxy groups in the molecular core positively influenced activity as well. The compound **7b**, featuring ethanolamine as the solubilizer, demonstrated IC_50_ = 0.74 µM. However, altering the terminal fragment of the molecule to acetamide resulted in a minor deterioration in biological activity to IC_50_ = 1.36 µM for **7a**.

In the studied series, a discernible correlation between variations in solubilizer type and biological activity was not evident. Even the more polar solubilizers such as serinol (compound **7g**) failed to impart a substantial impact on the activity, in comparison to compounds featuring ethanolamine (**7f**) and amide (**7e**).

According to the obtained HTRF results, the observed level of biological activity is strongly related to the position of additional aromatic rings with respect to the main *m*-terphenyl core. A clear correlation was observed between substitution in the ortho, meta, or para positions and the estimated IC_50_ of the studied molecules. The para-substituted compounds are characterized by the most linear structure and exhibit the best activities. Among them, the compound **7j** with bromine substituent in the additional aromatic rings and acetamide as solubilizer achieved the best IC_50_ estimated result at the level of 0.31 µM. Changing the entire aromatic substituent from para to meta position (derivative **4c**) resulted in a significant decrease in the compound’s activity, as observed by raising the IC_50_ value to 20.52 µM. As predicted based on molecular modeling, the compound **5a**, with ortho-substituted aromatic moiety, adopts unfavorable conformation leading to ineffective interaction with the PD-L1 homodimer-generated binding pocket and thus exhibits the worst activity among all compared isomers.

**Figure 3:**
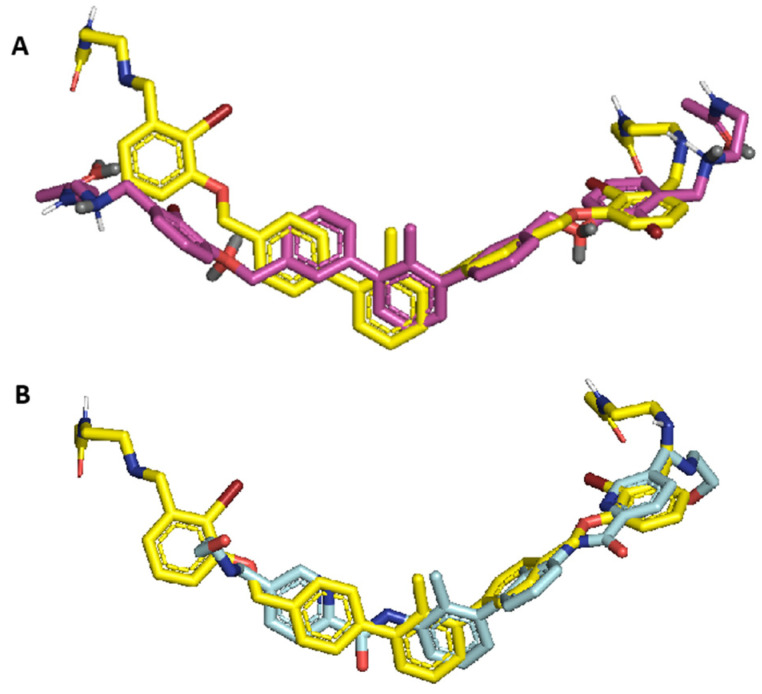
(**A**)—Compound **7j** (yellow) and its’ meta-substituted analog (purple) best-docked poses superposition. (**B**)—Superposition of compound **7j**’s best-docked pose (yellow) and native ligand from 6VQN protein (cyan).

We have tried to analyze the effect of the substitution pattern of the rings B and B’ on the position of the molecule in the binding pocket. Therefore, we compared the compound **7j** with its modeled meta- and ortho-substituted isomers. As expected, the meta-substituted compound’s position was slightly different, which translated to a lower molecular docking score. The ortho-substituted compound did not fit into the binding pocket of PD-L1 due to the unfavorable position of aromatic rings C and C’. We concluded that the para configuration is the most optimal for the C2-symmetrical *m*-terphenyl scaffold, which is in line with experimental data from the HTRF assay. The overlayed para-(**7j**) and meta-isomers are shown in Figure 3 and Figure S1.

### 2.3. NMR Binding Assay

**Figure 4:**
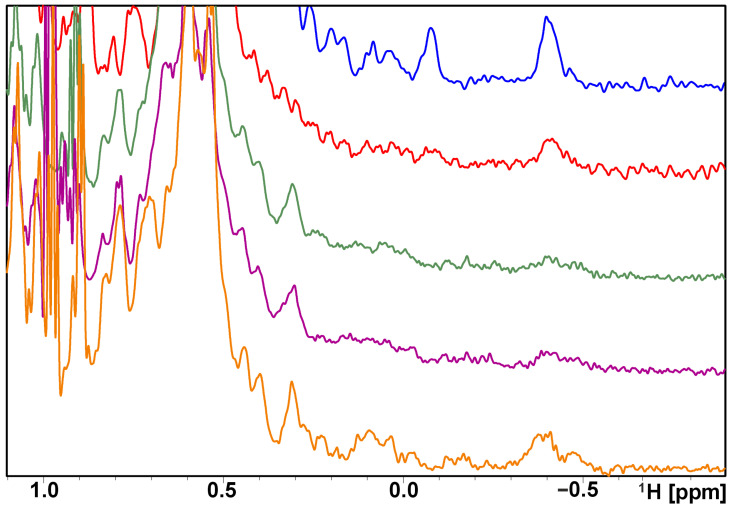
^1^H NMR spectra of the aliphatic part of the human PDL1 protein (blue) with compounds **7k** (red), **8b** (green) in a molar ratio of 1:1, and with positive control compounds **5b** [37] (purple) and **4a** [36] (orange) containing a terphenyl scaffold, previously described by us as a positive control.

The affinities of compounds **7k** and **8b** selected from SMIs described herein were tested towards PD-L1 using ^1^H NMR spectroscopy, alongside **5b [37]**, **4a** [36], and BMS-1166 [20,21,22] (Figure 4 and Figure S2). Compounds **5b** and **4a** were chosen as positive controls as their structures consist of an *m*-terphenyl scaffold and activity has been verified in our previous work [36,37] (Figure 4, Structures of reference compounds are available in Supplementary Materials Table S1). Titration of PD-L1 with increasing amounts of the compounds resulted in perturbations of the proton chemical shifts (Figure S2). We observed a significant broadening of the PD-L1 proton signals in the aliphatic part of the proton spectra, which is characteristic of the oligomerization of PD-L1, as previously observed by us for fragments and compounds containing biphenyl and terphenyl scaffolds [24,36,41]. The affinity of the test compounds for the PD-L1 protein is confirmed by the noticeable changes in bandwidth and signal intensity in the NMR spectra after the addition of the tested compounds to the protein. Observed changes are in agreement with our previous findings for biphenyl and terphenyl-based PD-L1 inhibitors that cause oligomerization of the protein. For such compounds, only a broadening of the resonance line of the signals in ^1^H NMR spectra can be observed, and it is retained in the spectra upon the addition of the higher inhibitor ratio [23,36,37].

### 2.4. Molecular Docking

**Figure 5:**
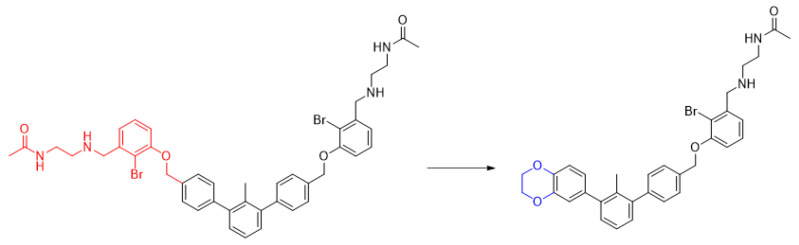
Example of the long, symmetric and short, nonsymmetric analogs; (**left**)—**7j**, (**right**)—**7j-half**.

Compound A, as one of the most prominent small molecule inhibitors of the PD-1/PD-L1 interaction, is an important base for ligand structure optimization in the search for C2-symmetric compounds with an improved activity [28]. Thus, all the *m*-terphenyl derivatives presented in this paper were docked onto the homodimeric structure of PD-L1 retrieved from its complex with Compound A (PDB ID: 6VQN). The short analogs of the C2-symmetric structures were generated by deleting part of the molecule beyond the B *m*-terphenyl ring and replacing it with a benzodioxane moiety, characteristic for the first-generation short PD-L1 SMIs [20,21]. This was done to compare the docking results for long, symmetric and short, nonsymmetric scaffold (Figure 5). Generally, the “halved” analogs performed worse in terms of scoring function results.

**Figure 6:**
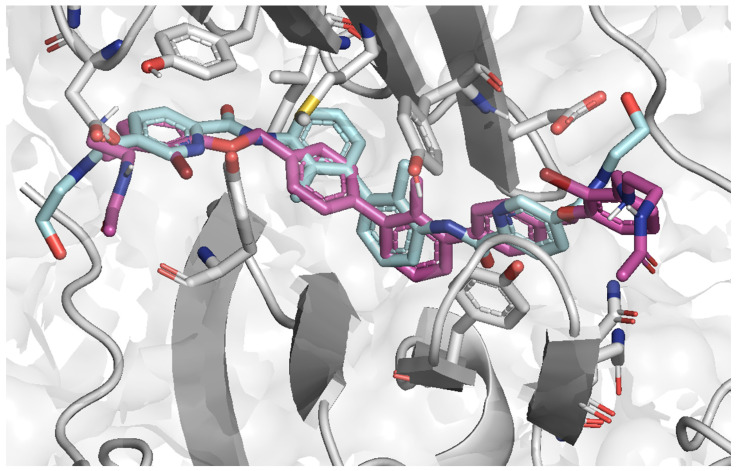
Superposition of the best docking poses of Compound A (6VQN) [28]; (aquamarine) and **7j** (purple) in the PD-L1 binding pocket (grey).

The docked poses of the reference ligands are in high accordance with the native conformation of Compound A observed in the crystal structure, especially concerning the positioning of the biphenyl core. This suggests good modeling performance. For the *m*-terphenyl molecules, the highest deviation from the crystal structure pose is observed in the solubilizer orientation. This finding is consistent with our previously reported results [37]. The conformational variety of the solubilizer is due to the increased ethanolamine group freedom resulting in a less tight binding of this molecular fragment to the protein. Considering this acceptable displacement of the terminal group, the theoretical model performs well in assessing the binding in the main molecular *m*-terphenyl/biphenyl core (Figure 6). At the same time, the scoring function results may be misleading due to the orientation and substituent variability of the solubilizing part of the molecule. This may result in the formation of additional interactions which, in consequence, may increase the scoring function value. However, due to the dynamic nature of this ending fragment and its contact with solvent molecules, the interaction may be incidental rather than leading to the stabilizing effect in the modeled complex.

**Figure 7:**
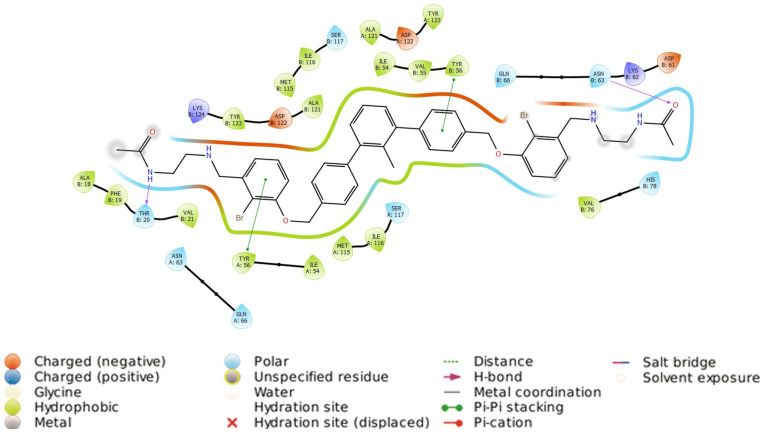
Two-dimensional protein–ligand interaction map of 6VQN and best docked pose of **7j**.

We analyzed the binding mode of **7j** to the PD-L1 homodimer obtained during the docking procedure. The ligand forms mainly hydrophobic contacts with *B*Ile_54_, *B*Tyr_56_, *B*Lys_62_, *A*Met_115_, *B*Met_115_, *A*Ala_121_, *B*Ala_121_, *A*Tyr_123_, and *B*Tyr_123_. These amino acids form the core of the binding pocket of the investigated protein system. Additionally, the molecule interacts with *A*Tyr_56_, *A*Asp_61_, *B*Asp_61_, *B*Lys_124,_ and *B*Arg_125_ through hydrogen bonds. The 2D map of the protein–ligand interactions is shown in Figure 7.

The main amino acids that stabilize the ligand within the binding site are Tyr_56_ of both homodimer units (*A* and *B*). They form π–π interactions with ligands’ aromatic fragments. However, this interaction is not symmetrical, as *B*Tyr_56_ and *A*Tyr_56_ interact with B’ and C rings, respectively. The part of the molecule containing the C’ ring is thus deeply extended into the more polar part of the *B* homodimer unit’s binding site fragment, forming interactions with *B*Asp_61_, *B*Lys_124,_ and *B*Arg_125_. In contrast to the biphenyl moiety of Compound A, the *m*-terphenyl core breaks the symmetry of the binding pocket. This result prompts that the C2-symmetrical *m*-terphenyl ligands cannot achieve the desired symmetrical binding mode. Thus, the second generation of non-symmetrical *m*-terphenyl scaffold can be even more advantageous, in terms of fitting to the symmetrical binding pocket, leading to more active ligands.

### 2.5. Ligands’ Pose Geometry Analysis

**Figure 8:**
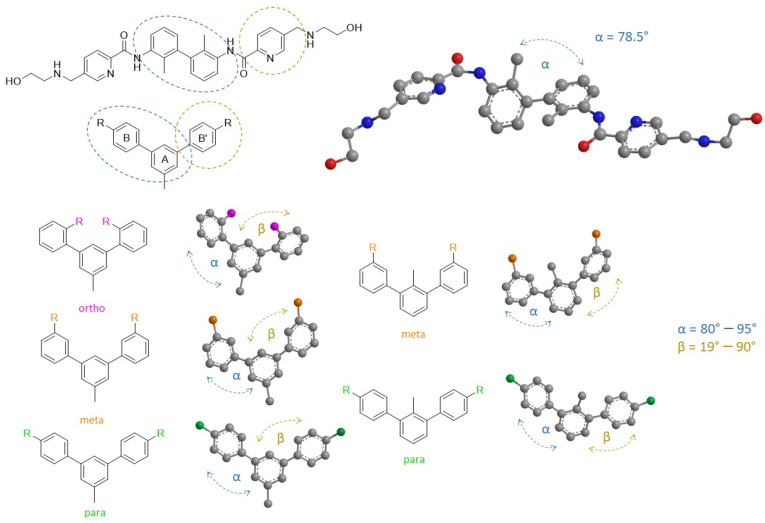
Schematic representation of the calculated angles in the Compound A molecule and the m-terphenyl derivatives. The α angle (blue) between rings A and B, which superpose with the biphenyl core of Compound A, takes optimal values between 80°–95° for most biologically active m-terphenyls. The β (yellow) angle between rings A and B’ that corresponds to position one of the pyridine rings of Compound A demonstrates favorable values from 19° to 90°.

Triggered by the variety of the docking scores for the studied C2-symmetrical structures, we analyzed the conformational differences of the *m*-terphenyl core geometry in search of a possible explanation. Thus, for each compound, the angles between the aromatic rings within the m-terphenyl moiety in the best-scored poses were measured. The first angle (α) was assigned between mean planes of the A and B rings which superpose with the biphenyl moiety of Compound A in the binding pocket (Figure 8 and Figure S3). This angle in the biphenyl core taken from the 6VQN structure is 78.5° (all measured angle values are available in Table S2). Interestingly, the re-docking result for the random starting geometry of Compound A achieved 59.5°. However, the superposed biphenyl RMSD being 0.192 implies a strong similarity between both compared poses.

This suggests that for the symmetrical inhibitors based on biphenyl core, the conformational freedom allowing twisting of the rings and retaining the relative orientation of the π–electron systems in the homodimer pocket is possible. The situation is different for the studied compounds. We found that α angle in C2-symmetric *m*-terphenyl varies between 45° and 95°, with a statistical maximum in the range of 78°–95°. The B’ ring superposes onto one of the pyridine rings of Compound A. Thus, to further evaluate the *m*-terphenyl poses, we define the angle between rings A and B’ as β. The value of this angle is usually noticeably smaller than α, introducing a degree of asymmetry in the *m*-terphenyl spatial conformation. The observed values of β are in the range 5°–65°. However, the most prominent compounds in the HTRF test adopt this angle in the range of 9°–19° for the docked poses. This suggests that the optimal C2-terphenyl set of α and β angles is around 78°–95° and 9°–19°, respectively. In contrast, for the ligand–protein crystal structures 7NLD [36] and 8R6Q [37] with short, nonsymmetrical *m*-terphenyl ligands, both angles are more similar with values between 43–64°. We docked the native ligand of 7NLD, and in our model, angles α and β are 78.2° and 17.6°, respectively. This significant difference suggests a possible conformational flexibility for short m-terphenyls, in comparison to the C2-symmetric ligands, which can be related to more constrained structural demands based on the number and type of protein–ligand interactions. The smaller size of “halved” compounds allows for more flexibility in the binding pocket, but at the same time, they lack the second terminal fragment that could have the potential to stabilize the ligand’s position in the binding site by providing more protein-ligand interactions. Although most of the generated “halved” compounds followed the estimated optimal angle values of α and β for C2-terphenyls, a few of them adopted angles akin to the 7NLD and 8R6Q native ligands. However, since the docking scores were generally visibly lower than their elongated C2-symmetric analogs, the tradeoff between lower strain and binding affinity is far less favorable in “halved” compounds.

Such geometrical arrangement is rarer for C2-symmetric *m*-terphenyl ligands due to the abovementioned conformational demands. Nevertheless, the sum of α and β is in most cases in the range of 90–120°, which is consistent among all investigated structures. The observed increased rigidity in terms of spatial orientation of the ligand in the binding pocket leads to heightened susceptibility to substitution position/substituent type in the B/B’ ring, with the para-, meta-, and ortho-substitution being characteristic for the most active, moderately active, and inactive compounds respectively. As a confirmation of this observation, the meta- and ortho-analogs of **7j** were additionally prepared and docked onto PD-L1 homodimer, showing the para-substituted compounds preference over other isomers and *ortho*-substituted not fitting to the binding pocket at all, as it was mentioned in the HTRF section (Section 2.2). The β angle for *meta*-substituted B/B’ ring tends to be higher and more similar to short *m*-terphenyls (including the generated “halved” structures), being in the range 20–40°, which lowers both the scoring function and HTRF results. Generally, the ortho-substituted structures could not fit the binding site. The docking results for the “halved” analogs show that the α and β angle diversity is greater, in contrast to long C2-symmetric *m*-terphenyls, for which the activity may be increased but imposes more geometrical constraints on the ligand’s structure. Taking into account the herein-mentioned results, it is possible to define general trends in the spatial fitting and positioning of the elongated, C2-symmetric scaffold. However, it is difficult to derive precise geometrical rules for PD-L1 ligands based solely on the analysis of selected angles in SMI’s structures.

## 3. Materials and Methods

### 3.1. Chemical Synthesis

All the reagents and solvents that were used for the synthesis and characterization of the compounds were received from commercial suppliers such as Sigma Aldrich (MilliporeSigma, Burlington, MA, USA,), Alfa-Aesar (Ward Hill, MA, USA), Acros Organic (Thermo Fisher Scientific, Waltham, MA, USA), and Ambeed Inc (Arlington Heights, IL, USA), and they were used without further purification. Nuclear magnetic resonance (NMR) spectra were recorded using Bruker Avance III 600 (Bruker, Billerica, MA, USA) (^1^H NMR at 600 MHz and ^13^C NMR at 151 MHz) or Jeol 400 (^1^H NMR at 400 MHz and ^13^C NMR at 101 MHz) at 300 K. Chemical shifts (δ) were reported in ppm and coupling constants (*J*) in Hz. All chemical shifts were analyzed in correspondence to the solvent peaks (DMSO-d_6_, MeOD-d_4_, CDCl_3,_ DMF-d_7_). Infrared spectra (IR) were measured using the Nicolet IR 200 spectrometer (Thermo Fisher Scientific, Waltham, MA, USA) with the ATR technique. Thin-layer chromatography (TLC) was performed on aluminum sheets precoated Silica Gel 60 F_254_ (MilliporeSigma, Burlington, MA, USA ). Techniques of visualization of TLC plates included radiation with a UV lamp at 254 nm wavelength. Purification of compounds was conducted by flash chromatography on the Grace Reveleris X2 Flash Chromatography System with the Grace Resolv Silica Cartridges (BÜCHI Labortechnik AG, Flawil, Switzerland). SFC-MS chromatograms were measured on a Waters Investigator Supercritical Fluid Chromatograph with a 3100 MS detector (ESI) (Waters Associates, Milford, MA, USA) using a solvent system of methanol and CO_2_ on a 2-Ethylpyridine column (4.6 × 250 mm, 5 µm particle size). The purity of all final compounds determined using chromatographic LC-MS was >90%. Peaks were integrated using Waters software MassLynx 4.1. High-resolution mass spectrometry (HRMS) analysis was carried out with the microTOF-QII spectrometer using the ESI ionization technique (Bruker, Billerica, MA, USA). Detailed synthesis protocols for intermediate products along with their respective characterization can be found in the Supporting Information.

#### 3.1.1. General Procedure for Final Product Synthesis: Method A. Reductive Amination

Dialdehyde (**2b**, **2e**, **2f**, **2k**, **2l**) (1 eq.), appropriate amine (10 eq.), glacial acetic acid (0.1 mL for 0.5 mmol), and anhydrous DMF were placed in a round-bottom flask under argon atmosphere. The reaction mixture was stirred at room temperature for 4 h. After this time, NaBH_3_CN (10 eq.) was added and the reaction mixture was stirred further at room temperature for 48 h. When the process was completed, the water was added, and the extraction with ethyl acetate followed. Organic phases were combined, dried over anhydrous MgSO_4_, and evaporated. Crude product was purified by column chromatography, giving the final products **4c**, **5a**, **6a**, **7j**, **7k**, and **7l** with 23–45% yields.

#### 3.1.2. General Procedure for Final Product Synthesis: Method B. Nucleophilic Substitution

Appropriate alcohol (**3a**–**3l**) (1.0 eq) was placed in a round-bottom flask with anhydrous dichloromethane and drops of anhydrous DMF (0.2 mL for 2 mmol alcohol) and cooled in an ice bath. Then, thionyl chloride (10 eq.) was added dropwise. The reaction was stirred for approximately 2 h at room temperature and controlled by TLC. When all substrate disappeared, the reaction was purred on to 1 M NaOH and extracted with dichloromethane. Organic phases were collected, dried over anhydrous MgSO_4_ and evaporated. The crude product was used without further purification in the next step.

Crude benzyl chloride was dissolved in anhydrous DMF and appropriate amine (10.0 eq.) dissolved in anhydrous DMF was added together with diisopropylethylamine (DIPEA, 4.0 eq.). The reaction was stirred in 80 °C overnight. After completion of the reaction, it was poured into water and extracted with chloroform. Organic phases were collected, dried over anhydrous MgSO_4_, and evaporated. Crude products were purified via column chromatography, giving the final compounds **4a**, **4b**, **4d**–**4f**, **7a**–**7i**, **7m**, **7n,** and **8a**–**8c** with yields of 15–66%.

N,N’-(((((((5′-methyl-[1,1′:3′,1′′-terphenyl]-3,3′′-diyl)bis(methylene))bis(oxy))bis(3,1-phenylene))bis(methylene))bis(azanediyl))bis(ethane-2,1-diyl))diacetamide (**4a**)

Method B: Compound **3a** (0.40 g, 0.77 mmol 1.0 eq.), SOCl_2_ (0.56 mL, 7.7 mmol, 10 eq.), N-(2-Aminoethyl)acetamide (0.74 mL, 7.7 mmol, 10.0 eq), DIPEA (0.54 mL, 3.0 mmol, 4.0 eq). The crude product was purified via column chromatography (SiO_2_, ethyl acetate to ethyl acetate/methanol, 1:2), giving Compound **4a** as a colorless solid with a 37% (0.20 g) yield.

R_f_ = 0.20 (SiO_2_, methanol); ^1^H NMR (600 MHz, CDCl_3_) δ (ppm): 7.43 (d, *J* = 8.2 Hz, 4H), 7.33 (d, *J* = 8.2 Hz, 4H), 7.24–7.19 (m, 3H), 7.18–7.15 (m, 2H), 6.92 (s, 2H), 6.87–6.84 (m, 4H), 5.99 (s, 2H), 5.05 (s, 4H), 3.71 (s, 4H), 3.28 (q, *J* = 5.6 Hz, 4H), 2.71 (t, *J* = 5.8 Hz, 4H), 2.06 (s, 3H), 1.90 (s, 6H); ^13^C NMR (151 MHz, CDCl_3_) δ (ppm): 170.4, 159.2, 142.6, 142.3, 141.7, 135.6, 133.1, 129.8, 129.3, 127.5, 125.6, 120.9, 114.9, 113.5, 70.0, 53.5, 48.0, 39.2, 23.5, 18.9; IR (ATR) (cm^−1^): 3423, 3284, 2927, 1639, 1557, 1442, 1261, 1169, 1012, 791; SFC-MS (ESI): t_R_ = 4.69 min, calculated for C_43_H_48_N_4_O_4_ (*m*/*z*) [M+H]^+^ 685.4, found for [M+H]^+^ 685.4; HRMS (ESI): calculated for C_43_H_48_N_4_O_4_ (*m*/*z*) [M+H]^+^ 685.3748, found for [M+H]^+^ 685.3750.

N,N’-(((((((5′-methyl-[1,1′:3′,1′′-terphenyl]-3,3′′-diyl)bis(methylene))bis(oxy))bis(2-methoxy-4,1-phenylene))bis(methylene))bis(azanediyl))bis(ethane-2,1-diyl))diacetamide (**4b**)

Method B: Compound **3b** (0.65 g, 1.10 mmol 1.0 eq.), SOCl_2_ (0.82 mL, 11.0 mmol, 10 eq.), N-(2-Aminoethyl)acetamide (1.09 mL, 11.0 mmol, 10.0 eq), DIPEA (0.79 mL, 4.4 mmol, 4.0 eq). The crude product was purified by column chromatography (SiO_2_, ethyl acetate to ethyl acetate/methanol, 1:4), giving Compound **4b** as a yellowish oil with a 51% (0.43 g) yield.

R_f_ = 0.41 (SiO_2_, CHCl_3_/7M NH_3_ in MeOH, 10:1); ^1^H NMR (600 MHz, CDCl_3_) δ (ppm): 7.29–7.22 (m, 5H), 7.15–7.11 (m, 4H), 7.05 (d, *J* = 7.2 Hz, 2H), 6.72–6.68 (m, 4H), 6.60 (dd, *J* = 8.1, 1.9 Hz, 2H), 5.88 (s, 2H), 5.02 (s, 4H), 3.72 (s, 6H), 3.54 (s, 4H), 3.16 (q, *J* = 5.6 Hz, 4H), 2.58 (t, *J* = 5.8 Hz, 4H), 1.88 (s, 3H), 1.79 (s, 6H); ^13^C NMR (151 MHz, CDCl_3_) δ (ppm): 170.4, 162.3, 149.9, 147.4, 142.7, 142.7, 137.2, 133.4, 133.1, 129.2, 129.0, 128.5, 128.5, 126.0, 125.5, 120.4, 114.4, 112.1, 71.4, 56.1, 53.4, 48.1, 39.3, 23.4, 18.8; IR (ATR) (cm^−1^): 3291, 3063, 2933, 1652, 1512, 1463, 1373, 1263, 1226, 1139, 1032, 783, 709; SFC-MS (ESI): t_R_ = 1.45 min, calculated for C_45_H_52_N_4_O_6_ (*m*/*z*) [M+H]^+^ 745.4, found for [M+H]^+^ 745.4; HRMS (ESI): calculated for C_45_H_52_N_4_O_6_ (*m*/*z*) [M+H]^+^ 745.3960, found for [M+H]^+^ 745.3961.

N,N’-(((((((5′-methyl-[1,1′:3′,1′′-terphenyl]-3,3′′-diyl)bis(methylene))bis(oxy))bis(2-bromo-3,1-phenylene))bis(methylene))bis(azanediyl))bis(ethane-2,1-diyl))diacetamide (**4c**)

Method A: Compound **2b** (0.30 g, 0.45 mmol 1.0 eq.), N-(2-Aminoethyl)acetamide (0.48 mL, 4.5 mmol, 10.0 eq.), NaBH_3_CN (0.28 g, 4.5 mmol, 10.0 eq.). The crude product was purified by column chromatography (SiO_2_, SiO_2_, chloroform to chloroform/7M NH_3_ in MeOH, 10:1), giving Compound **4c** as a white solid with a 33% (0.38 g) yield.

R_f_ = 0.31 (SiO_2_, CHCl_3_/7M NH_3_ in MeOH, 25:1); ^1^H NMR (600 MHz, MeOD-d_4_) δ (ppm): 7.49–7.47 (m, 4H), 7.45 (t, *J* = 7.8 Hz, 2H), 7.30–7.26 (m, 5H), 7.19 (d, *J* = 7.6 Hz, 2H), 7.08–7.02 (m, 4H), 5.24 (s, 4H), 3.90 (s, 4H), 3.32 (m, 4H), 2.72 (t, *J* = 6.3 Hz, 4H), 2.02 (s, 3H), 1.92 (s, 6H); ^13^C NMR (151 MHz, CDCl_3_) δ (ppm): 170.4, 155.5, 142.8, 142.7, 140.7, 136.5, 133.1, 129.3, 129.2, 128.6, 128.3, 128.1, 125.7, 125.6, 123.0, 114.4, 112.9, 71.2, 53.7, 47.7, 39.1, 23.5, 18.9; IR (ATR) (cm^−1^): 3413, 3285, 3070, 2925, 1644, 1569, 1435, 1372, 1270, 1028, 783, 709; SFC-MS (ESI): t_R_ = 5.10 min, calculated for C_43_H_46_Br_2_N_4_O_4_ (*m*/*z*) [M+H]^+^ 841.2, found for [M+H]^+^ 841.2; HRMS (ESI): calculated for C_43_H_46_Br_2_N_4_O_4_ (*m*/*z*) [M+H]^+^ 841.1959, found for [M+H]^+^ 841.1957.

N,N’-(((((((2,2′′-difluoro-5′-methyl-[1,1′:3′,1′′-terphenyl]-3,3′′-diyl)bis(methylene))bis(oxy))bis(3,1-phenylene))bis(methylene))bis(azanediyl))bis(ethane-2,1-diyl))diacetamide (**4d**)

Method B: Compound **3c** (0.88 g, 1.60 mmol 1.0 eq.), SOCl_2_ (1.15 mL, 16.0 mmol, 10 eq.), N-(2-Aminoethyl)acetamide (1.53 mL, 16.0 mmol, 10.0 eq), DIPEA (1.11 mL, 6.4 mmol, 4.0 eq). The crude product was purified by column chromatography (SiO_2_, chloroform to chloroform/7M NH_3_ in MeOH, 10:1), giving Compound **4d** as a yellowish oil with a 38% (0.45 g) yield.

R_f_ = 0.45 (SiO_2_, CHCl_3_/7M NH_3_ in MeOH, 10:1); ^1^H NMR (600 MHz, DMSO-d_6_) δ (ppm): 7.78 (t, *J* = 5.0 Hz, 2H), 7.64 (bs, 1H), 7.61–7.54 (m, 2H), 7.52 (s, 1H), 7.48 (bs, 1H), 7.43 (s, 1H), 7.39–7.30 (m, 2H), 7.23 (t, *J* = 7.8 Hz, 2H), 7.04 (s, 2H), 6.91 (t, 8.8 Hz, 4H), 5.19 (s, 4H), 3.66 (s, 4H), 3.11 (q, *J* = 6.2 Hz, 4H), 2.51 (m, 4H), 2.45 (s, 3H), 1.77 (s, 6H); ^13^C NMR (151 MHz, CDCl_3_) δ (ppm): 170.4, 158.9, 158.8, 158.3, 156.7, 142.0, 138.4, 135.8, 131.5, 130.8, 129.7, 129.4, 129.1, 129.0, 129.0, 125.0, 124.9, 124.5, 124.3, 121.0, 121.0, 114.8, 114.7, 113.5, 113.4, 64.1, 64.0, 63.8, 53.5, 48.1, 39.3, 23.4, 21.7; IR (ATR) (cm^−1^): 3292, 3070, 2930, 1650, 1584, 1487, 1449, 1376, 1259, 1156, 1033, 783; SFC-MS (ESI): t_R_ = 4.60 min, calculated for C_43_H_46_F_2_N_4_O_4_ (*m*/*z*) [M+H]^+^ 721.4, found for [M+H]^+^ 721.3; HRMS (ESI): calculated for C_43_H_46_F_2_N_4_O_4_ (*m*/*z*) [M+H]^+^ 721.3560, found for [M+H]^+^ 721.3557.

N,N’-(((((((2,2′′,5′-trimethyl-[1,1′:3′,1′′-terphenyl]-3,3′′-diyl)bis(methylene))bis(oxy))bis(2-bromo-3,1-phenylene))bis(methylene))bis(azanediyl))bis(ethane-2,1-diyl))diacetamide (**4e**)

Method B*:* Compound **3d** (0.30 g, 0.43 mmol 1.0 eq.), SOCl_2_ (0.31 mL, 4.3 mmol, 10 eq.), N-(2-Aminoethyl)acetamide (0.41 mL, 4.3 mmol, 10.0 eq), DIPEA (0.30 mL, 1.7 mmol, 4.0 eq). The crude product was purified by column chromatography (SiO_2_, ethyl acetate to ethyl acetate/7M NH_3_ in methanol, 10:1), giving Compound **4e** as a colorless solid with a 66% (0.25 g) yield.

R_f_ = 0.23 (SiO_2_, ethyl acetate/methanol, 2:1); ^1^H NMR (600 MHz, CDCl_3_) δ (ppm): 7.55–7.51 (m, 2H), 7.30–7.27 (m, 4H), 7.24 (d, *J* = 7.9 Hz, 2H), 7.14–7.12 (m, 2H), 7.08 (bs, 1H), 6.98 (dd, *J* = 7.6, 1.2 Hz, 2H), 6.95 (dd, *J* = 8.2, 1.3 Hz, 2H), 6.18 (bs, 2H), 5.16 (s, 4H), 3.89 (s, 4H), 3.34 (q, *J* = 5.5 Hz, 4H), 2.76 (t, *J* = 5.8 Hz, 4H), 2.45 (s, 3H), 2.33 (s, 6H), 1.97 (s, 6H); ^13^C NMR (151 MHz, CDCl_3_) δ (ppm): 170.4, 155.6, 142.9, 141.7, 140.9, 137.7, 135.0, 133.9, 133.3, 130.2, 128.9, 128.1, 127.9, 127.5, 125.7, 122.9, 114.3, 112.6, 70.2, 53.8, 47.7, 39.2, 23.5, 22.8, 16.4; IR (ATR) (cm^−1^): 3349, 3295, 3161, 2919, 1644, 1569, 1452, 1396, 1294, 1274, 1091, 1027, 866, 772, 789, 714; SFC-MS (ESI): t_R_ = 2.06 min, calculated for C_45_H_50_Br_2_N_4_O_4_ (*m*/*z*) [M+H]^+^ 869.2, found for [M+H]^+^ 869.2; HRMS (ESI): calculated for C_45_H_50_Br_2_N_4_O_4_ (*m*/*z*) [M+H]^+^ 869.2272, found for [M+H]^+^ 869.2269.

N,N’-(((((((2,2′′,5′-trimethyl-[1,1′:3′,1′′-terphenyl]-3,3′′-diyl)bis(methylene))bis(oxy))bis(2,5-difluoro-4,1-phenylene))bis(methylene))bis(azanediyl))bis(ethane-2,1-diyl))diacetamide (**4f**)

Method B Compound **3e** (0.27 g, 0.44 mmol 1.0 eq.), SOCl_2_ (0.32 mL, 4.4 mmol, 10 eq.), N-(2-Aminoethyl)acetamide (0.42 mL, 4.4 mmol, 10.0 eq), DIPEA (0.30 mL, 1.7 mmol, 4.0 eq). The crude product was purified by column chromatography (SiO_2_, ethyl acetate to ethyl acetate/methanol, 2:1), giving the final compound, **4f,** as a light yellow solid with a 36% (0.16 g) yield.

R_f_ = 0.43 (SiO_2_, ethyl acetate/methanol, 2:1); ^1^H NMR (600 MHz, CDCl_3_) δ (ppm): 7.41 (dd, *J* = 7.3, 1.4 Hz, 2H), 7.31–7.24 (m, 4H), 7.12 (bs, 2H), 7.09–7.04 (m, 3H), 6.80 (dd, *J* = 10.8, 6.9 Hz, 2H), 6.05 (bs, 2H), 5.13 (s, 4H), 3.75 (s, 4H), 3.34 (q, *J* = 5.6 Hz, 4H), 2.75 (t, *J* = 5.8 Hz, 4H), 2.45 (s, 3H), 2.31 (s, 6H), 1.98 (6H); ^13^C NMR (151 MHz, CDCl_3_) δ (ppm): 170.4, 157.5, 155.9, 149.9, 148.3, 146.6, 146.5, 146.5, 143.1, 141.6, 137.8, 134.5, 134.4, 130.6, 128.9, 128.0, 127.8, 125.8, 119.3, 119.2, 119.2, 119.1, 117.1, 117.1, 117.0, 118.0, 103.7, 103.6, 71.0, 47.9, 46.2, 39.2, 23.4, 21.6, 16.4; IR (ATR) (cm^−1^): 3450, 3294, 2930, 1638, 1510, 1420, 1375, 1333, 1218, 1165, 1111, 993, 871, 794, 718; SFC-MS (ESI): t_R_ = 1.68 min, calculated for C_45_H_48_F_4_N_4_O_4_ (*m*/*z*) [M+H]^+^ 785.4, found for [M+H]^+^ 785.4; HRMS (ESI): calculated for C_45_H_48_F_4_N_4_O_4_ (*m*/*z*) [M+H]^+^ 785.3684, found for [M+H]^+^ 785.3681.

N,N’-(((((((5′-methyl-[1,1′:3′,1′′-terphenyl]-2,2′′-diyl)bis(methylene))bis(oxy))bis(3-methoxy-2,1-phenylene))bis(methylene))bis(azanediyl))bis(ethane-2,1-diyl))diacetamide (**5a**)

Method A: Compound **2e** (0.40 g, 0.70 mmol 1.0 eq.), N-(2-Aminoethyl)acetamide (0.67 mL, 7.0 mmol, 10.0 eq.), NaBH_3_CN (0.44 g, 7.0 mmol, 10.0 eq.). The crude product was purified by column chromatography (SiO_2_, chloroform/methanol, 9:1), giving Compound **5a** as a white solid with a 25% (0.13 g) yield.

R_f_ = 0.20 (SiO_2_, chloroform/methanol, 5:1); ^1^H NMR (600 MHz, CDCl_3_) δ (ppm): 7.52 (d, *J* = 6.6 Hz, 2H), 7.43–7.32 (m, *J* = 19.0, 11.9, 7.0 Hz, 7H), 7.14 (bs, 2H), 6.99 (t, *J* = 7.8 Hz, 2H), 6.86–6.78 (m, *J* = 18.2, 7.7 Hz, 4H), 6.74 (s, 2H), 5.06 (s, 4H), 3.71 (s, 6H), 3.47 (s, 4H), 3.15–3.05 (m, 4H), 2.53–2.45 (m, 4H), 2.40 (s, 3H), 1.85 (s, 6H); ^13^C NMR (151 MHz, CDCl_3_) δ (ppm)): 170.6, 152.5, 145.8, 141.8, 140.4, 137.8, 134.7, 130.2, 130.1, 129.1, 128.3, 127.7, 127.4, 124.3, 121.9, 112.2, 72.5, 55.6, 47.6, 47.5, 38.0, 23.1, 21.5; IR (ATR) (cm^−1^): 3407, 3273, 2929, 2362, 1653, 1559, 1477, 1370, 1272, 1078, 971, 762; SFC-MS (ESI): t_R_ = 3.78 min, calculated for C_45_H_52_N_4_O_6_ (*m*/*z*) [M+H]^+^ 745.4, found for [M+H]^+^ 745.4; HRMS (ESI): calculated for C_45_H_52_N_4_O_6_ (*m*/*z*) [M+H]^+^ 745.3960, found for [M+H]^+^ 745.3960.

N,N’-(((((((3,3′′-dimethoxy-5′-methyl-[1,1′:3′,1′′-terphenyl]-4,4′′-diyl)bis(methylene))bis(oxy))bis(3-methoxy-2,1-phenylene))bis(methylene))bis(azanediyl))bis(ethane-2,1-diyl))diacetamide (**6a**)

Method A: Compound **2f** (0.21 g, 0.3 mmol 1.0 eq.), N-(2-Aminoethyl)acetamide (0.32 mL, 3.0 mmol, 10.0 eq.), glacial acetic acid (0.07 mL, 1.22 mmol, 4.0 eq.), NaBH_3_CN (0.21 g, 3.0 mmol, 10.0 eq.). The crude product was purified by column chromatography (SiO_2_, ethyl acetate to ethyl acetate/methanol, 2:1), giving the final compound, **6a,** as a yellowish solid with a 34% (0.26 g) yield.

R_f_ = 0.10 (SiO_2_, chloroform/methanol, 5:1); ^1^H NMR (600 MHz, MeOD-d_4_) δ (ppm): 7.52 (s, 1H), 7.34 (s, 2H), 7.29 (d, *J* = 7.7 Hz, 2H), 7.15 (d, *J* = 1.3 Hz, 2H), 7.12 (dd, *J* = 7.7, 1.5 Hz, 2H), 6.95–6.89 (m, 4H), 6.74 (dd, *J* = 7.4, 1.5 Hz, 2H), 5.02 (s, 4H), 3.82 (s, 6H), 3.80 (s, 6H), 3.55 (s, 4H), 3.11 (t, *J* = 6.4 Hz, 4H), 2.48 (t, *J* = 6.4 Hz, 4H), 2.37 (s, 3H), 1.72 (s, 6H); ^13^C NMR (151 MHz, MeOD-d_4_) δ (ppm): 172.1, 158.1, 152.9, 146.1, 143.1, 141.5, 138.8, 132.3, 130.7, 126.8, 124.9, 123.8, 122.8, 121.7, 118.9, 112.0, 109.2, 69.3, 55.0, 54.8, 48.5, 43.6, 38.3, 21.1, 20.3; IR (ATR) (cm^−1^): 3291, 2930, 2362, 1654, 1575, 1477, 1272, 1078, 1036, 974, 751; SFC-MS (ESI): t_R_ = 4.20 min, calculated for C_47_H_56_N_4_O_8_ (*m*/*z*) [M+H]^+^ 805.4, found for [M+H]^+^ 805.4; HRMS (ESI): calculated for C_47_H_56_N_4_O_8_ (*m*/*z*) [M+H]^+^ 805.4171, found for [M+H]^+^ 805.4170.

N,N’-(((((((3,3′′-dimethoxy-2′-methyl-[1,1′:3′,1′′-terphenyl]-4,4′′-diyl)bis(methylene))bis(oxy))bis(3-methoxy-4,1-phenylene))bis(methylene))bis(azanediyl))bis(ethane-2,1-diyl))diacetamide (**7a**)

Method B: Compound **3f** (0.21 g, 0.32 mmol, 1.0 eq.), SOCl_2_ (0.24 mL, 3.3 mmol, 10.0 eq.), *N*-(2-aminoethyl)acetamide (0.34 g, 3.4 mmol, 10.0 eq.), DIPEA (0.23 mL, 1.32 mmol, 4.0 eq.). The crude product was purified by column chromatography (SiO_2_, chloroform/methanol, 7:3), giving the final compound, **7a,** as a yellowish solid with a 46% (0.12 g) yield.

R_f_ = 0.20 (SiO_2_, chloroform/methanol, 8:2); ^1^H NMR (600 MHz, CDCl_3_) δ (ppm): 7.44 (bs, 2H), 7.25–7.10 (m, 3H), 7.03–6.73 (m, 10H), 5.16 (s, 4H), 3.85 (s, 6H), 3.83 (s, 6H), 3.66 (bs, 4H), 2.71 (s, 4H), 2.06 (s, 4H), 1.92 (s, 3H), 1.21 (s, 6H); IR (ATR) (cm^−1^): 3385, 2921, 2851, 1652, 1571, 1514, 1463, 1403, 1262, 1228, 1139, 1034, 832, 799; SFC-MS (ESI): t_R_ = 1.51 min, calculated for C_47_H_56_N_4_O_8_ (*m*/*z*) [M+H]^+^ 805.4, found for [M+H]^+^ 805.4; HRMS (ESI): calculated for C_47_H_56_N_4_O_8_ (*m*/*z*) [M+H]^+^ 805.4171, found for [M+H]^+^ 805.4169.

2,2′-((((((3,3′′-dimethoxy-2′-methyl-[1,1′:3′,1′′-terphenyl]-4,4′′-diyl)bis(methylene))bis(oxy))bis(3-methoxy-4,1-phenylene))bis(methylene))bis(azanediyl))bis(ethan-1-ol) (**7b**)

Method B: Compound **3f** (0.15 g, 0.24 mmol, 1.0 eq.), SOCl_2_ (0.17 mL, 2.4 mmol, 10.0 eq.), ethanolamine (0.14 g, 2.4 mmol, 10.0 eq.), DIPEA (0.17 g, 0.94 mmol, 4.0 eq.). The crude product was purified by column chromatography (SiO_2_, chloroform to chloroform/7M NH_3_ in methanol, 8:2), giving the final compound, **7b,** as a yellowish solid with a 21% (0.04 g) yield.

R_f_ = 0.18 (SiO_2_, chloroform/methanol, 7:3); ^1^H NMR (600 MHz, CDCl_3_) δ (ppm): 7.51 (d, *J* = 7.7 Hz, 2H), 7.29–7.27 (m, 1H), 7.25–7.23 (m, 2H), 6.95 (dd, *J* = 7.7, 1.4 Hz, 2H), 6.94–6.89 (m, 4H), 6.87 (d, *J* = 1.3. Hz, 2H), 6.81 (dd, *J* = 8.1, 1.9 Hz, 2H), 5.22 (s, 4H), 3.91 (s, 6H), 3.87 (s, 6H), 3.74 (s, 4H), 3.65 (t, *J* = 5.1 Hz, 4H), 2.80 (t, *J* = 5.2 Hz, 4H), 2.13 (s, 3H); ^13^C NMR (151 MHz, CDCl_3_) δ (ppm): 156.5, 149.8, 147.8, 143.2, 143.0, 132.5, 129.1, 128.3, 125.4, 124.1, 121.8, 120.6, 113.9, 112.1, 111.6, 66.1, 60.8, 56.2, 55.6, 53.3, 50.5, 25.0, 18.9; IR (ATR) (cm^−1^): 3312, 2926, 2852, 2362, 2343, 1687, 1574, 1516, 1462, 1403, 1264, 1226, 1142, 1033, 799; SFC-MS (ESI): t_R_ = 1.19 min, calculated for C_43_H_50_N_2_O_8_ (*m*/*z*) [M+H]^+^ 723.4, found for [M+H]^+^ 723.4; HRMS (ESI): calculated for C_43_H_50_N_2_O_8_ (*m*/*z*) [M+H]^+^ 723.3640, found for [M+H]^+^ 723.3643.

N,N’-(((((((2,2′,2′′-trimethyl-[1,1′:3′,1′′-terphenyl]-4,4′′-diyl)bis(methylene))bis(oxy))bis(3-methoxy-4,1-phenylene))bis(methylene))bis(azanediyl))bis(ethane-2,1-diyl))diacetamide (**7c**)

Method B: Compound **3g** (0.20 g, 0.33 mmol, 1.0 eq.), SOCl_2_ (0.24 mL, 3.3 mmol, 10.0 eq.), *N*-(2-aminoethyl)acetamide (0.34 g, 3.3 mmol, 10.0 eq.), DIPEA (0.23 mL, 1.32 mmol, 4.0 eq.). The crude product was purified by column chromatography (SiO_2_, chloroform to chloroform/methanol, 1:9), giving the final compound, **7c,** as a yellowish solid with a 42% (0.11 g) yield.

R_f_ = 0.23 (SiO_2_, chloroform/methanol, 7:3); ^1^H NMR (600 MHz, DMSO-d_6_) δ (ppm) (mixture of rotamers): 7.84–7.74 (m, 2H), 7.38 (s, 2H), 7.33–7.31 (m, 2H), 7.16 (t, *J* = 7.9 Hz, 2H), 7.10 (d, *J* = 7.5 Hz, 2H), 7.04–6.95 (m, 4H), 6.82 (d, *J* = 6.7 Hz, 1H), 5.05 (s, 4H), 3.78 (s, 6H), 3.61 (s, 4H), 3.12 (q, *J* = 6.3 Hz, 4H), 2.07, and 2.05 (singlets, 6H, from rotamers), 1.79 (s, 6H), 1.68 and 1.66 (singlets, 3H from rotamers); ^13^C NMR (151 MHz, DMSO-d_6_) δ (ppm) (mixture of rotamers): 169.5, 149.5, 147.1, 141.8, 141.4, 136.8, 135.7, 134.4, 129.8, 129.7, 129.4, 128.6, 125.8, 120.3, 113.8, 112.5, 70.4, 55.9, 52.9, 48.5, 29.5, 23.1, 20.1, 19.8, 17.5; IR (ATR) (cm^−1^): 3292, 3078, 2922, 2851, 2360, 1679, 1586, 1511, 1464, 1423, 1339, 1266, 1136, 1032, 806, 735; SFC-MS (ESI): t_R_ = 4.49 min, calculated for C_47_H_56_N_4_O_6_ (*m*/*z*) [M+H]^+^ 773.4, found for [M+H]^+^ 773.4; HRMS (ESI): calculated for C_47_H_56_N_4_O_6_ (*m*/*z*) [M+H]^+^ 773.4273, found for [M+H]^+^ 773.4271.

2,2′-((((((2,2′,2′′-trimethyl-[1,1′:3′,1′′-terphenyl]-4,4′′-diyl)bis(methylene))bis(oxy))bis(3-methoxy-4,1-phenylene))bis(methylene))bis(azanediyl))bis(ethan-1-ol) (**7d**)

Method B: Compound **3g** (0.20 g, 0.33 mmol, 1.0 eq.), SOCl_2_ (0.42 mL, 3.31 mmol, 10.0 eq.), ethanolamine (0.20 g, 3.3 mmol, 10.0 eq.), DIPEA (0.23 mL, 1.3 mmol, 4.0 eq.). The crude product was purified by column chromatography (SiO_2_, chloroform to chloroform/methanol, 1:9), giving the final compound, **7d,** as a yellowish solid with a 42% (0.10 g) yield.

R_f_ = 0.24 (SiO_2_, methanol); ^1^H NMR (600 MHz, DMSO-d_6_) δ (ppm) (mixture of rotamers): 7.38 (s, 2H), 7.35–7.27 (m, 3H), 7.23–7.12 (m, 2H), 7.09 (d, *J* = 7.5 Hz, 2H), 7.02–6.97 (m, 4H), 6.82 (d, *J* = 8.1 Hz, 2H), 5.04 (s, 4H), 3.77 (s, 6H), 3.63 (s, 4H), 3.46 (t, *J* = 5.8 Hz, 4H), 2.55 (t, *J* = 5.8 Hz, 4H), 2.06 and 2.05 (singlets, 6H, from rotamers), 1.68 and 1.66 (singlets, 3H, from rotamers); ^13^C NMR (151 MHz, DMSO-d_6_) δ (ppm) (mixture of rotamers): 149.5, 147.1, 141.8, 141.4, 136.8, 135.7, 134.5, 129.7, 129.4, 128.6, 125.8, 120.3, 113.8, 112.4, 70.4, 60.9, 55.9, 53.2, 51.5, 29.5, 20.1, 19.8, 17.5; IR (ATR) (cm^−1^): 3350, 3059, 2921, 2851, 2362, 1682, 1587, 1511, 1464, 1424, 1339, 1266, 1136, 1031, 806, 735; SFC-MS (ESI): t_R_ = 4.78 min, calculated for C_43_H_50_N_2_O_6_ (*m*/*z*) [M+H]^+^ 691.4, found for [M+H]^+^ 691.4; HRMS (ESI): calculated for C_43_H_50_N_2_O_6_ (*m*/*z*) [M+H]^+^ 691.3742, found for [M+H]^+^ 691.3743.

N,N’-(((((((2′,3,3′′-trimethyl-[1,1′:3′,1′′-terphenyl]-4,4′′-diyl)bis(methylene))bis(oxy))bis(3-methoxy-4,1-phenylene))bis(methylene))bis(azanediyl))bis(ethane-2,1-diyl))diacetamide (**7e**)

Method B: Compound **3h** (0.25 g, 0.41 mmol, 1.0 eq.), SOCl_2_ (0.30 mL, 4.1 mmol, 10.0 eq.), *N*-(2-aminoethyl)acetamide (0.42 g, 4.1 mmol, 10.0 eq.), DIPEA (0.28 mL, 1.65 mmol, 4.0 eq.). The crude product was purified by column chromatography (SiO_2_, chloroform to chloroform/methanol, 8:2), giving the final compound, **7e,** as a colorless solid with a 44% (0.14 g) yield.

R_f_ = 0.26 (SiO_2_, methanol); ^1^H NMR (600 MHz, CDCl_3_) δ (ppm): 7.46 (d, *J* = 7.7 Hz, 2H), 7.21–7.18 (m, 5H), 6.94 (d, *J* = 8.1 Hz, 2H), 6.89 (s, 2H), 6.83 (dd, *J* = 8.1, 1.6 Hz, 2H), 6.09 (bs, 2H), 5.13 (s, 4H), 3.89 (s, *J* = 6.6 Hz, 6H), 3.74 (s, 4H), 3.38–3.31 (m, 4H), 2.78 (t, *J* = 5.8 Hz, 4H), 2.43 (s, 6H), 2.12 (s, 3H), 1.97 (s, 6H); ^13^C NMR (151 MHz, CDCl_3_) δ (ppm): 170.3, 150.0, 147.6, 142.6, 142.3, 136.3, 133.5, 133.3, 131.4, 128.9, 128.4, 127.0, 125.3, 120.3, 114.3, 112.1, 69.8, 56.0, 53.3, 50.7, 48.0, 39.2, 23.3, 19.0, 18.8; IR (ATR) (cm^−1^): 3292, 2972, 2929, 2357, 1648, 1560, 1519, 1463, 1265, 1137, 1038, 995, 803; SFC-MS (ESI): t_R_ = 1.65 min, calculated for C_47_H_56_N_4_O_6_ (*m*/*z*) [M+H]^+^ 773.4, found for [M+H]^+^ 773.4; HRMS (ESI): calculated for C_47_H_56_N_4_O_6_ (*m*/*z*) [M+H]^+^ 773.4273, found for [M+H]^+^ 773.4273.

2,2′-((((((2′,3,3′′-trimethyl-[1,1′:3′,1′′-terphenyl]-4,4′′-diyl)bis(methylene))bis(oxy))bis(3-methoxy-4,1-phenylene))bis(methylene))bis(azanediyl))bis(ethan-1-ol) (**7f**)

Method B: Compound **3h** (0.25 g, 0.41 mmol, 1.0 eq.), SOCl_2_ (0.30 mL, 4.13 mmol, 10.0 eq.), ethanolamine (0.25 g, 4.13 mmol, 10.0 eq.), DIPEA (0.28 mL, 1.7 mmol, 4.0 eq.). The crude product was purified by column chromatography (SiO_2_, chloroform to chloroform/methanol, 8:2), giving the final compound, **7f,** as a colorless solid with a 42% (0.12 g) yield.

R_f_ = 0.27 (SiO_2_, methanol); ^1^H NMR (600 MHz, CDCl_3_) δ (ppm): 7.39 (d, *J* = 7.7 Hz, 2H), 7.19–7.17 (m, 1H), 7.15–7.11 (m, 5H), 7.11 (s, 1H), 6.86 (s, 1H), 6.86–6.82 (m, 3H), 6.76 (dd, *J* = 8.1, 1.6 Hz, 2H), 5.05 (s, 4H), 3.82 (s, 6H), 3.69 (s, 4H), 3.59 (t, *J* = 5.2 Hz, 4H), 2.74 (t, *J* = 5.1 Hz, 4H), 2.36 (s, 6H), 2.05 (s, 3H); ^13^C NMR (151 MHz, CDCl_3_) δ (ppm): 150.0, 147.6, 142.6, 142.3, 136.3, 133.5, 131.4, 128.9, 128.4, 127.0, 125.3, 120.3, 114.3, 112.0, 69.9, 61.0, 56.0, 53.3, 50.5, 19.0, 18.8; IR (ATR) (cm^−1^): 3332, 3010, 2930, 1594, 1520, 1463, 1405, 1266, 1138, 1037, 993, 891, 841, 803; SFC-MS (ESI): t_R_ = 1.63 min, calculated for C_43_H_50_N_2_O_6_ (*m*/*z*) [M+H]^+^ 691.4, found for [M+H]^+^ 691.4; HRMS (ESI): calculated for C_43_H_50_N_2_O_6_ (*m*/*z*) [M+H]^+^ 691.3742, found for [M+H]^+^ 691.3741

2,2′-((((((2′,3,3′′-trimethyl-[1,1′:3′,1′′-terphenyl]-4,4′′-diyl)bis(methylene))bis(oxy))bis(3-methoxy-4,1-phenylene))bis(methylene))bis(azanediyl))bis(propane-1,3-diol) (**7g**)

Method B: Compound **3h** (0.20 g, 0.33 mmol, 1.0 eq.), SOCl_2_ (0.24 mL, 3.3 mmol, 10.0 eq.), serinol (0.30 g, 3.3 mmol, 10.0 eq.), DIPEA (0.23 mL, 1.32 mmol, 4.0 eq.). The crude product was purified by column chromatography (SiO_2_, chloroform to chloroform/methanol, 9:1), giving the final compound, **7g,** as a colorless solid with a 15% (0.04 g) yield.

R_f_ = 0.32 (SiO_2_, methanol); ^1^H NMR (600 MHz, DMF-d_7_) δ (ppm): 7.60 (d, *J* = 7.6 Hz, 2H), 7.42–7.24 (m, 9H), 7.22–7.15 (m, 2H), 7.10 (d, *J* = 8.1 Hz, 2H), 5.20 (s, 4H), 4.15 (s, 4H), 3.86 (s, 6H), 3.79–3.71 (m, 8H), 2.48 (s, 6H), 2.18 (s, 2H), 1.28 (bs, 3H); ^13^C NMR (151 MHz, CDCl_3_) δ (ppm): 154.0, 152.3, 146.5, 146.4, 140.4, 137.1, 135.2, 132.8, 132.3, 130.8, 129.1, 125.5, 118.1, 116.7, 73.6, 63.7, 63.3, 59.8, 53.9, 33.5, 32.0, 22.7, 22.5; IR (ATR) (cm^−1^): 3676, 3308, 2989, 2921, 2365, 1684, 1593, 1520, 1463, 1419, 1266, 1138, 1038, 862, 738; SFC-MS (ESI): t_R_ = 1.27 min, calculated for C_45_H_54_N_2_O_8_ (*m*/*z*) [M+H]^+^ 751.4, found for [M+H]^+^ 751.4; HRMS (ESI): calculated for C_45_H_54_N_2_O_8_ (*m*/*z*) [M+H]^+^ 751.3953, found for [M+H]^+^ 751.3954.

N,N’-(((((((2′,3,3′′-trimethyl-[1,1′:3′,1′′-terphenyl]-4,4′′-diyl)bis(methylene))bis(oxy))bis(2,5-difluoro-4,1-phenylene))bis(methylene))bis(azanediyl))bis(ethane-2,1-diyl))diacetamide (**7h**)

Method B: Compound **3i** (0.23 g, 0.37 mmol, 1.0 eq.), SOCl_2_ (0.25 mL, 3.7 mmol, 10.0 eq.), N-(2-aminoethyl)acetamide (0.37 g, 3.7 mmol, 10.0 eq.), DIPEA (0.25 mL, 1.46 mmol, 4.0 eq.). The crude product was purified by column chromatography (SiO_2_, chloroform to chloroform/7M NH_3_ in methanol, 9:1), giving the final compound, **7h,** as a colorless solid with a = = 16% (0.04 g) yield.

R_f_ = 0.28 (SiO_2_, chloroform/methanol, 7:3); ^1^H NMR (600 MHz, CDCl_3_) δ (ppm): 7.48 (d, *J* = 7.5 Hz, 2H), 7.33–7.27 (m, 2H), 7.24 (d, *J* = 7.4 Hz, 4H), 7.11 (dd, *J* = 10.5, 7.1 Hz, 2H), 6.85 (dd, *J* = 10.0, 7.1 Hz, 2H), 6.07 (s, 1H), 5.14 (s, 4H), 3.78 (s, 4H), 3.37 (d, *J* = 5.1 Hz, 4H), 2.78 (d, *J* = 5.1 Hz, 4H), 2.47 (s, 6H), 2.15 (s, 3H), 2.01 (s, 6H); ^13^C NMR (151 MHz, CDCl_3_) δ (ppm): 170.3, 157.4, 155.8, 149.8, 148.2, 146.4, 142.8, 142.4, 136.6, 132.9, 132.3, 131.6, 129.0, 128.6, 127.1, 125.4, 119.3, 117.0, 103.7, 70.3, 47.8, 46.1, 39.1, 23.3, 19.0, 18.8; IR (ATR) (cm^−1^): 3286, 3084, 2929, 2854, 2362, 2343, 1637, 1559, 1521, 1459, 1375, 1336, 1294, 1217, 1165, 1118, 990, 883, 834, 791; SFC-MS (ESI): t_R_ = 4.95 min, calculated for C_45_H_48_F_4_N_4_O_4_ (*m*/*z*) [M+H]^+^ 785.4, found for [M+H]^+^ 785.4; HRMS (ESI): calculated for C_45_H_48_F_4_N_4_O_4_ (*m*/*z*) [M+H]^+^ 785.3684, found for [M+H]^+^ 785.3684.

2,2′-((((((2′,3,3′′-trimethyl-[1,1′:3′,1′′-terphenyl]-4,4′′-diyl)bis(methylene))bis(oxy))bis(2,5-difluoro-4,1-phenylene))bis(methylene))bis(azanediyl))bis(ethan-1-ol) (**7i**)

Method B: Compound **3i** (0.23 g, 0.37 mmol, 1.0 eq.), SOCl_2_ (0.25 mL, 3.65 mmol, 10.0 eq.), ethanolamine (0.22 g, 3.7 mmol, 10.0 eq.), DIPEA (0.25 mL, 1.5 mmol, 4.0 eq.). The crude product was purified by column chromatography (SiO_2_, chloroform to chloroform/7M NH_3_ in methanol, 9:1), giving the final compound, **7i**, as a colorless solid with a 21% (0.05 g) yield.

R_f_ = 0.25 (SiO_2_, chloroform/methanol, 7:3); ^1^H NMR (600 MHz, CDCl_3_) δ (ppm): 7.37 (d, *J* = 7.7 Hz, 2H), 7.22–7.19 (m, *J* = 9.8, 5.0 Hz, 1H), 7.18–7.09 (m, 6H), 7.02 (dd, *J* = 11.1, 6.9 Hz, 2H), 6.74 (dd, *J* = 10.7, 6.9 Hz, 2H), 5.04 (s, 4H), 3.74 (s, 4H), 3.60 (t, *J* = 5.1 Hz, 4H), 2.72 (t, *J* = 5.2 Hz, 4H), 2.36 (s, 6H), 2.05 (s, 3H); ^13^C NMR (151 MHz, CDCl_3_) δ (ppm): 157.4, 155.8, 149.9, 148.2, 146.4, 142.8, 142.5, 136.6, 132.9, 132.3, 131.6, 129.0, 128.6, 127.1, 125.4, 119.3, 116.9, 103.6, 103.5, 70.3, 61.0, 50.3, 46.1, 19.0, 18.8; IR (ATR) (cm^−1^): 3219, 2924, 2868, 2362, 2337, 1637, 1521, 1459, 1422, 1336, 1217, 1165, 1105, 882, 833, 792; SFC-MS (ESI): t_R_ = 1.79 min, calculated for C_41_H_42_F_4_N_2_O_4_ (*m*/*z*) [M+H]^+^ 703.3, found for [M+H]^+^ 703.3; HRMS (ESI): calculated for C_41_H_42_F_4_N_2_O_4_ (*m*/*z*) [M+H]^+^ 703.3153, found for [M+H]^+^ 703.3151.

N,N’-(((((((2′-methyl-[1,1′:3′,1′′-terphenyl]-4,4′′-diyl)bis(methylene))bis(oxy))bis(2-bromo-3,1-phenylene))bis(methylene))bis(azanediyl))bis(ethane-2,1-diyl))diacetamide (**7j**)

Method A: Compound **2k** (0.20 g, 0.30 mmol 1.0 eq.), N-(2-Aminoethyl)acetamide (0.29 mL, 3.0 mmol, 10.0 eq.), NaBH_3_CN (0.18 g, 3.0 mmol, 10.0 eq.). The crude product was purified by column chromatography (SiO_2_, ethyl acetate to ethyl acetate/methanol, 2:1), giving the final compound, **7j**, as a white solid with a 45% (0.11 g) yield.

R_f_ = 0.23 (SiO_2_, ethyl acetate/methanol, 1:1); ^1^H NMR (600 MHz, DMSO-d_6_) δ (ppm): 7.81 (t, *J* = 5.7 Hz, 2H), 7.57 (d, *J* = 8.1 Hz, 4H), 7.43 (d, *J* = 8.1 Hz, 4H), 7.37–7.29 (m, 3H), 7.23 (d, *J* = 7.6 Hz, 2H), 7.17–7.10 (m, 4H), 5.26 (s, 4H), 3.77 (s, 4H), 3.14 (q, *J* = 6.3 Hz, 4H), 2.57 (t, *J* = 6.5 Hz, 4H), 2.09 (s, 3H), 1.79 (s, 6H); ^13^C NMR (151 MHz, DMSO-d_6_) δ (ppm): 169.1, 154.4, 142.0, 141.3, 141.2, 135.5, 132.2, 129.3, 128.9, 127.9, 127.1, 125.7, 121.8, 112.8, 112.2, 69.9, 52.7, 48.2, 45.2, 22.7, 18.6; IR (ATR) (cm^−1^): 3309, 3065, 2920, 2860, 1685, 1591, 1515, 1464, 1379, 1263, 1235, 1139, 1033, 790; SFC-MS (ESI): t_R_ = 1.90 min, calculated for C_43_H_46_Br_2_N_4_O_4_ (*m*/*z*) [M+H]+ 841.2, found for [M+H]+ 841.2; HRMS (ESI): calculated for C_43_H_46_Br_2_N_4_O_4_ (*m*/*z*) [M+H]^+^ 841.1959, found for [M+H]^+^ 841.1956.

N,N’-(((((((2′-methyl-[1,1′:3′,1′′-terphenyl]-4,4′′-diyl)bis(methylene))bis(oxy))bis(2-methoxy-4,1-phenylene))bis(methylene))bis(azanediyl))bis(ethane-2,1-diyl))diacetamide (**7k**)

Method A: Compound **2l** (0.28 g, 0.49 mmol 1.0 eq.), N-(2-Aminoethyl)acetamide (0.47 mL, 4.9 mmol, 10.0 eq.), NaBH_3_CN (0.30 g, 4.9 mmol, 10.0 eq.). The crude product was purified by column chromatography (SiO_2_, ethyl acetate to ethyl acetate/methanol, 2:1), giving the final compound, **7k**, as a yellowish solid with a 24% (0.09 g) yield.

R_f_ = 0.30 (SiO_2_, methanol/7M NH_3_ in MeOH, 20:1); ^1^H NMR (600 MHz, DMSO-d_6_) δ (ppm): 7.79 (t, *J* = 5.2 Hz, 2H), 7.52 (d, *J* = 8.1 Hz, 4H), 7.41 (d, *J* = 8.1 Hz, 4H), 7.33 (t, *J* = 7.5 Hz, 1H), 7.22 (d, *J* = 7.6 Hz, 2H), 7.03–6.97 (m, 4H), 6.82 (dd, *J* = 8.2, 1.6 Hz, 2H), 5.10 (s, 4H), 3.78 (s, 6H), 3.63 (s, 4H), 3.13 (q, *J* = 6.3 Hz, 4H), 2.53 (t, *J* = 6.7 Hz, 4H), 2.09 (m, 3H), 1.78 (s, 6H); ^13^C NMR (151 MHz, DMSO-d_6_) δ (ppm): 173.6, 169.1, 152.4, 145.4, 142.2, 141.3, 136.6, 134.5, 132.2, 129.1, 128.8, 128.0, 125.7, 123.9, 121.0, 111.4, 73.8, 55.8, 48.4, 47.3, 23.5, 22.6, 18.6; IR (ATR) (cm^−1^): 3298, 2931, 2857, 1591, 1516, 1464, 1419, 1381, 1263, 1232, 1139, 1033, 790; SFC-MS (ESI): t_R_ = 1.35 min, calculated for C_45_H_52_N_4_O_6_ (*m*/*z*) [M+H]+ 745.4, found for [M+H]+ 745.4; HRMS (ESI): calculated for C_45_H_52_N_4_O_6_ (*m*/*z*) [M+H]^+^ 745.3960, found for [M+H]^+^ 745.3959.

2,2′-((((((2′-methyl-[1,1′:3′,1′′-terphenyl]-4,4′′-diyl)bis(methylene))bis(oxy))bis(2-methoxy-4,1-phenylene))bis(methylene))bis(azanediyl))bis(ethan-1-ol) (**7l**)

Method A: Compound **2l** (0.28 g, 0.49 mmol 1.0 eq.), N-(2-Aminoethyl)acetamide (0.47 mL, 4.9 mmol, 10.0 eq.), NaBH_3_CN (0.30 g, 4.9 mmol, 10.0 eq.). The crude product was purified by column chromatography (SiO_2_, ethyl acetate to ethyl acetate/methanol, 2:1) giving the final compound **7l** as a yellowish solid with 23% (0.08 g) yield.

R_f_ = 0.25 (SiO_2_, methanol/7M NH_3_ in MeOH, 20:1); ^1^H NMR (600 MHz, DMSO-d_6_) δ (ppm): 7.53 (d, *J* = 8.1 Hz, 4H), 7.42 (d, *J* = 8.1 Hz, 4H), 7.36–7.31 (m, 1H), 7.22 (dd, *J* = 7.6, 2.9 Hz, 2H), 7.04–6.98 (m, 4H), 6.89–6.81 (m, 2H), 5.11 (s, 4H), 4.47 (bs, 2H), 3.78 (s, 6H), 3.65 (s, 4H), 3.47 (m, 4H), 2.57 (t, *J* = 5.8 Hz, 4H), 2.09 (s, 3H); ^13^C NMR (151 MHz, DMSO-d_6_) δ (ppm): 149.0, 146.6, 142.1, 141.2, 136.0, 133.7, 132.2, 129.2, 128.9, 127.7, 125.7, 119.9, 113.3, 112.1, 69.8, 60.3, 55.5, 52.6, 50.9, 18.7; IR (ATR) (cm^−1^): 3313, 2922, 2856, 1591, 1517, 1464, 1381, 1263, 1236, 1140, 1034, 878, 790; SFC-MS (ESI): t_R_ = 1.60 min, calculated for C_41_H_46_N_2_O_6_ (*m*/*z*) [M+H]^+^ 663.3, found for [M+H]^+^ 663.3; HRMS (ESI): calculated for C_41_H_46_N_2_O_6_ (*m*/*z*) [M+H]^+^ 663.3429, found for [M+H]^+^ 663.3427.

N,N’-(((((((2′-chloro-[1,1′:3′,1′′-terphenyl]-4,4′′-diyl)bis(methylene))bis(oxy))bis(2-methoxy-4,1-phenylene))bis(methylene))bis(azanediyl))bis(ethane-2,1-diyl))diacetamide (**7m**)

Method B: Compound **3j** (0.25 g, 0.4 mmol, 1.0 eq.), SOCl_2_ (0.65 mL, 8.9 mmol, 21.5 eq.), N-(2-aminoethyl)acetamide (0.43 g, 4.2 mmol, 10.1 eq.), DIPEA (0.35 mL, 2.01 mmol, 4.8 eq.). The crude product was purified by maceration with acetonitrile, giving the final compound, **7m,** as a yellowish solid with a 30% (0.09 g) yield.

R_f_ = 0.45 (SiO_2_, methanol/7M NH_3_ in MeOH, 20:1); ^1^H NMR (600 MHz, DMSO-d_6_) δ (ppm): 7.78 (t, *J* = 5.1 Hz, 2H), 7.54 (d, *J* = 8.2 Hz, 4H), 7.51–7.48 (m, 5H), 7.41 (d, *J* = 7.5 Hz, 2H), 7.00–6.98 (m, 4H), 6.82 (dd, *J* = 8.2, 1.6 Hz, 2H), 5.11 (s, 4H), 3.78 (s, 6H), 3.61 (s, 4H), 3.12 (q, *J* = 6.3 Hz, 4H), 2.51 (t, *J* = 5.3 Hz, 4H), 1.78 (s, 6H); ^13^C NMR (151 MHz, DMSO-d_6_) δ (ppm): 169.1, 149.0, 146.6, 140.8, 138.9, 136.9, 133.8, 130.6, 129.8, 129.4, 127.6, 127.2, 119.9, 113.3, 112.1, 69.7, 55.5, 52.4, 48.0, 38.7, 22.7; IR (ATR) (cm^−1^): 3276, 3073, 2933, 2856, 1637, 1513, 1460, 1377, 1260, 1228, 1136, 1019, 1000, 790; SFC-MS (ESI): t_R_ = 1.66 min, calculated for C_44_H_49_ClN_4_O_6_ (*m*/*z*) [M+H]^+^ 765.3, found for [M+H]^+^ 765.3; HRMS (ESI): calculated for C_44_H_49_ClN_4_O_6_ (*m*/*z*) [M+H]^+^ 765.3413, found for [M+H]^+^ 765.3412.

2,2′-((((((2′-chloro-[1,1′:3′,1′′-terphenyl]-4,4′′-diyl)bis(methylene))bis(oxy))bis(2-methoxy-4,1-phenylene))bis(methylene))bis(azanediyl))bis(ethan-1-ol) (**7n**)

Method B: Compound **3j** (0.24 g, 0.4 mmol, 1.0 eq.), SOCl_2_ (0.6 mL, 8.2 mmol, 20.7 eq.), ethanolamine (0.3 mL, 4.9 mmol, 12.5 eq.), DIPEA (0.35 mL, 2.7 mmol, 6.8 eq.). The crude product was purified by maceration with acetonitrile, giving the final compound, **7n,** as a light yellow solid with a 51% (0.14 g) yield.

R_f_ = 0.25 (SiO_2_, methanol/7M NH_3_ in MeOH, 20:1); ^1^H NMR (600 MHz, DMSO-d_6_) δ (ppm): 7.54 (d, *J* = 8.4 Hz, 4H), 7.51–7.45 (m, 5H), 7.43–7.39 (m, 2H), 7.03–6.98 (m, 4H), 6.83 (dd, *J* = 8.7, 1.9 Hz, 2H), 5.11 (s, 4H), 3.78 (s, 6H), 3.66 (s, 4H), 3.47 (t, *J* = 5.7 Hz, 4H), 2.58 (t, *J* = 5.8 Hz, 4H); ^13^C NMR (151 MHz, DMSO-d_6_) δ (ppm): 149.0, 146.6, 140.8, 138.9, 136.8, 133.3, 130.6, 129.8, 129.4, 127.6, 127.2, 120.0, 133.3, 112.1, 69.7, 60.1, 55.5, 52.5, 50.8; IR (ATR) (cm^−1^): 3288, 2933, 2863, 1591, 1515, 1464, 1420, 1379, 1262, 1233, 1139, 1032, 791; SFC-MS (ESI): t_R_ = 1.63 min, calculated for C_40_H_43_ClN_2_O_6_ (*m*/*z*) [M+H]^+^ 683.3, found for [M+H]^+^ 683.3; HRMS (ESI): calculated for C_40_H_43_ClN_2_O_6_ (*m*/*z*) [M+H]^+^ 683.2882, found for [M+H]^+^ 683.2882.

2,2′-((((((2′-methyl-[1,1′:3′,1′′-terphenyl]-3,3′′-diyl)bis(methylene))bis(oxy))bis(2-bromo-3,1-phenylene))bis(methylene))bis(azanediyl))bis(ethan-1-ol) (**8a**)

Method B: Compound **3k** (0.20 g, 0.30 mmol, 1.0 eq), thionyl chloride (0.21 mL, 3.0 mmol, 10.0 eq), ethanolamine (0.18 mL, 3.0 mmol, 10.0 eq), DIPEA (0.21 mL, 1.2 mmol, 4.0 eq). The crude product was purified by column chromatography (SiO_2_, ethyl acetate to ethyl acetate/methanol, 2:1), giving the final compound, **8a**, as a light yellow solid with a 40% (0.09 g) yield.

R_f_ = 0,10 (SiO_2_, dichloromethane/methanol, 5:1); ^1^H NMR (400 MHz, CDCl_3_) δ (ppm): 7.52 (s, 1H), 7.49–7.42 (m, 5H), 7.35–7.26 (m, 4H), 7.24–7.19 (m, 3H), 7.07 (d, *J* = 6.4 Hz, 2H), 6.92 (d, *J* = 8.3 Hz, 2H), 5.20 (s, 4H), 3.99 (s, 4H), 3.72–3.63 (m, 4H), 2.87–2.75 (m, 4H), 2.06 (s, 3H); ^13^C NMR (101 MHz, CDCl_3_) δ (ppm): 155.4, 142.7, 142.7, 136.2, 133.1, 129.2, 129.1, 128.6, 128.6, 128.4, 128.2, 125.8, 125.7, 125.5, 123.2, 114.6, 113.3, 71.1, 59.9, 52.8, 50.0, 18.9; IR (ATR) (cm^−1^): 3298, 3059, 2925, 2857, 2361, 2343, 1570, 1458, 1375, 1270, 1029, 782, 709; SFC-MS (ESI): t_R_ = 2.17 min, calculated for C_39_H_40_Br_2_N_2_O_4_ (*m*/*z*) [M+H]^+^ 759.1, found for [M+H]^+^ 759.1; HRMS (ESI): calculated for C_39_H_40_Br_2_N_2_O_4_ (*m*/*z*) [M+H]^+^ 759.1428, found for [M+H]^+^ 759.1431.

N,N’-(((((((2′-methyl-[1,1′:3′,1′′-terphenyl]-3,3′′-diyl)bis(methylene))bis(oxy))bis(3-methoxy-2,1-phenylene))bis(methylene))bis(azanediyl))bis(ethane-2,1-diyl))diacetamide (**8b**)

Method B: Compound **3l** (0.30 g, 0.52 mmol, 1.0 eq), thionyl chloride (0.38 mL, 0.62 mmol, 10.0 eq), N-(2-aminoethyl)acetamide (0.50 mL, 5.2 mmol, 10.0 eq), DIPEA (0.36 mL, 2.1 mmol, 4.0 eq). The crude product was purified by column chromatography (SiO_2_, ethyl acetate to ethyl acetate/methanol, 1:2), giving the final compound, **8b**, as a light yellow solid with a 52% (0.20 g). yield

R_f_ = 0.10 (SiO_2_, dichloromethane/methanol, 5:1); ^1^H NMR (600 MHz, CDCl_3_) δ (ppm): 7.46–7.42 (m, 4H), 7.39 (bs, 2H), 7.35–7.31 (m, 2H), 7.30–7.26 (m, 1H), 7.20 (d, *J* = 7.5 Hz, 2H), 7.06–7.01 (m, 2H), 6.92–6.84 (m, 4H), 5.10 (s, 4H), 3.90 (s, 6H), 3.69 (s, 4H), 3.28–3.19 (m, 4H), 2.65–2.61 (m, 4H), 2.06 (s, 3H), 1.86 (s, 6H); ^13^C NMR (151 MHz, CDCl_3_) δ (ppm): 170.4, 152.7, 146.0, 142.6, 137.5, 133.5, 132.8, 129.3, 129.2, 129.1, 128.4, 126.9, 125.4, 124.2, 121.9, 111.8, 74.9, 55.8, 48.6, 47.7, 38.9, 23.1, 18.7; IR (ATR) (cm^−1^): 3272, 3067, 2931, 1361, 1654, 1559, 1481, 1458, 1437, 1375, 1274, 1184, 1081, 1041, 785; SFC-MS (ESI): t_R_ = 1.25 min, calculated for C_45_H_52_N_4_O_6_ (*m*/*z*) [M+H]^+^ 745.4, found for [M+H]^+^ 745.4; HRMS (ESI): calculated for C_45_H_52_N_4_O_6_ (*m*/*z*) [M+H]^+^ 745.3960, found for [M+H]^+^ 745.3962.

2,2′-((((((2′-methyl-[1,1′:3′,1′′-terphenyl]-3,3′′-diyl)bis(methylene))bis(oxy))bis(3-methoxy-2,1-phenylene))bis(methylene))bis(azanediyl))bis(ethan-1-ol) (**8c**)

Method B: Compounds **3l** (0.30 g, 0.52 mmol, 1.0 eq), thionyl chloride (0.38 mL, 6.2 mmol, 10.0 eq.), ethanolamine (0.31 mL, 5.2 mmol, 10.0 eq), DIPEA (0.36 mL, 2.1 mmol, 4.0 eq). The crude product was purified by column chromatography (SiO_2_, ethyl acetate to ethyl acetate/methanol, 2:1), giving the final compound, **8c**, as a light yellow solid with a 47% (0.16 g) yield.

R_f_ = 0.10 (SiO_2_, dichloromethane/methanol, 5:1); ^1^H NMR (600 MHz, CDCl_3_) δ (ppm): 7.47–7.37 (m, 6H), 7.35–7.23 (m, 4H), 7.17 (dd, *J* = 4.7, 2.0 Hz, 1H), 7.06–6.95 (m, 4H), 6.95–6.88 (m, 2H), 5.24 (bs, 2H), 5.14 (s, 4H), 3.88 (s, 6H), 3.83 (s, 4H), 3.64–3.57 (m, 4H), 2.75–2.66 (m, 4H), 2.07 (s, 3H); ^13^C NMR (151 MHz, CDCl_3_) δ (ppm): 152.6, 146.1, 142.7, 142.5, 136.9, 133.0, 129.8, 129.3, 129.0, 128.4, 128.2, 127.2, 125.4, 124.6, 122.5, 113.0, 75.0, 58.4, 55.9, 49.2, 46.5, 18.7; IR (ATR) (cm^−1^): 3310, 2933, 1670, 1585, 1479, 1364, 1271, 1208, 1182, 1081, 784, 134, 709; SFC-MS (ESI): t_R_ = 1.19 min, calculated for C_41_H_46_N_2_O_6_ (*m*/*z*) [M+H]^+^ 663.3, found for [M+H]^+^ 663.3; HRMS (ESI): calculated for C_41_H_46_N_2_O_6_ (*m*/*z*) [M+H]^+^ 663.3429, found for [M+H]^+^ 663.3427.

### 3.2. Homogenous Time-Resolved Fluorescence

The certified cis-bio assay kit was used to perform the HTRF assay. Following the standard protocol, the measurement was carried out at a 20 µL final volume with a 5 nM concentration of PD-L1 and a 50 nM concentration of PD-1 in the final formulation. Separate dilution series were performed to calculate the half-maximal inhibitory concentration (IC_50_) of the most potent compounds. Analyte and detection reagents (anti-analyte conjugated donor and acceptor conjugated antibody) were placed in the proper wells on the microplate as suggested in the cis-bio protocol. The plate was incubated for 1 h and then read on an HTRF-certified Tecan Spark 20M microplate reader. Output data were subjected to background subtraction on negative control (without PD-1) and normalization in correlation to the positive control (without inhibitor) and were then averaged. The resulting data points were then fitted with Hill’s equation to generate IC_50_ using Mathematica 12. Compounds were routinely tested in scouting mode (inhibitor concentration 50 and 5 µmol). The inhibitory constants for those compounds were approximated by translation of the Hill’s fit of fully described compound (**7b**, **7j**, **7m**) to match the experimental datapoints. Since all compounds reported here are closely related, the slope of Hill’s fit that determines the shape of the curve is therefore similar across them.

### 3.3. Protein Expression and Purification

The BL21 *E. coli* strain was transformed with the pET-21b plasmid containing the gene encoding the human PD-L1 protein (amino acids 18-134). The bacteria were cultured in LB at 37 °C until OD_600_ of 0.6, when the recombinant protein production was induced with 1 mM IPTG. The protein production was continued overnight at 28 °C. Inclusion bodies were collected by centrifugation, washed twice with 50 mM Tris-HCl pH 8.0 containing 200 mM NaCl, 10 mM EDTA, 10 mM 2- mercaptoethanol, and 0.5% Triton X-100, followed by a single wash with the same buffer without Triton X100. The washed inclusion bodies were resuspended overnight in 50 mM Tris-HCl pH 8.0, 6 M GuHCl, 200 mM NaCl, and 10 mM 2-mercaptoethanol and clarified with centrifugation. Refolding of PD-L1 was performed by drop-wise dilution into 0.1 M Tris-HCl pH 8.0 containing 1 M L-arginine hydrochloride, 2 mM EDTA, 0.25 mM oxidized glutathione, and 0.25 mM reduced glutathione. The refolded protein was dialyzed 3 times against 10 mM Tris-HCl pH 8.0 containing 20 mM NaCl and purified by size exclusion chromatography using Superdex 75 column equilibrated with 10 mM Tris, pH 8.0 20 mM NaCl. The purity of the refolded protein was evaluated by SDS-PAGE, and its correct folding was assessed by NMR (Figure S4).

### 3.4. NMR Binding Assay

NMR spectra were recorded in 10 mM Tris, pH 8.0 20 mM NaCl containing 10% (*v*/*v*) of D_2_O added to the samples to provide the lock signal. Water suppression was carried out using the WATERGATE sequence. All the spectra were recorded at 300 K using a Bruker AVANCE 600 MHz spectrometer (Bruker, Billerica, MA, USA) with the cryoplatform.

The spectra were recorded at the ligand/protein ratio of 1:1 unless stated otherwise. The samples were prepared by adding small amounts of a 50 mM ligand stock solution in DMSO to the protein solution (0.20 mL) of PD-L1 fragment at a concentration of 0.2 mM. Ligands used as a positive control, **5b** [37] and **4a** [36], were obtained by us according to the previous protocols [36,37]. Spectra were visualized using TopSpin 4.0.2.

### 3.5. Molecular Docking

The molecular docking procedure was carried out on the PD-L1/Compound A co-crystal structure [28] in GOLD [42] (PDB ID: 6VQN). This structure was chosen from PD-L1/SMIs complex structures found in the Protein Data Bank [43], based on the resolution criterion and symmetry of the organic ligand (Compound A is a C2-symmetrical molecule). All the designed *m*-terphenyl derivatives presented in this paper were docked onto the homodimeric structure of the pre-processed 6VQN protein with incomplete homodimers with 0.5 Compound A molecules and all non-protein molecules removed (mainly water molecules). The positions of all hydrogen atoms were calculated and added to the protein before docking. The docking region was defined based on the native ligand position, including all protein atoms within a 6 Å radius from the organic molecule. The docking was performed in semi-flexible mode. The genetic algorithm was applied for the ligand’s conformational search. The short analogs of the C2-symmetric structures were generated by deleting one part of the molecule beyond the terphenyl ring and replacing it with a benzodioxane moiety. The native Compound A in conformation observed for the selected protein–ligand crystal structure, and the randomly generated and minimized structure of Compound A were used as reference ligands. ChemPLP was used as the primary scoring function. Ligand–protein interaction for the best-docked pose of the compound **7j** and PD-L1 was analyzed with the use of Maestro software 13.2.128 (Schrödinger Release 2024-1: Maestro, Schrödinger, LLC, New York, NY, USA, 2024) and PLIP server (version 2.3.0) [44].

## 4. Conclusions

We have successfully synthesized 25 final C2-symmetrical compounds based on either 1,3,5-*m*-terphenyl or 1,2,3-*m*-terphenyl cores with additional substituents as aromatic rings in ortho, meta, and para positions. Subsequently, the HTRF assay and NMR binding assay show that the obtained compounds bind to PD-L1. Furthermore, we presented a description of the simulated ligand–protein complex formation within the binding pocket and the interactions between compound **7j** and the PD-L1 protein. In addition, we observed correlations between the conformational differences of the *m*-terphenyl core geometry, which led us to determine the planar angle ranges for the terphenyl moiety substituted at the meta, ortho, and para positions, corresponding to the biological activities of the compounds.

We found that the symmetrization of elongated terphenyl compounds to match the symmetry of Compound A did not increase their protein affinity. The symmetrical nature of PD-L1 homodimer leads to the conclusion that small molecule symmetrization should be beneficial, resulting in optimal interaction within the binding pocket. Nevertheless, the nature of symmetrical elongated *m*-terphenyl derivatives would not exploit these advantages due to their asymmetrical positioning inside the PD-L1 binding pocket, in contrast to the biphenyl-based symmetrical inhibitors, such as Compound A. This asymmetry comprises the interactions between the molecule and the protein, leading to reduced biological efficacy compared to Compound A. The observed asymmetry is expressed in the different values of the planar angles within the terphenyl core, which determine the behavior of the molecule in the binding pocket. The observed correlation between the angles within the *m*-terphenyl moiety and its interaction with the protein can be used in subsequent research as an additional parameter in the optimization of the structure of new inhibitors. Such an analysis can serve as a predictive factor in the design of molecules, particularly those based on the *m*-terphenyl core. Further attempts at symmetrization in the case of elongated *m*-terphenyls are unlikely to yield positive results. Consequently, asymmetric elongation should yield better results, and shorter, asymmetric terphenyl compounds are more likely to succeed as their structure should fit better into the protein-binding pocket.

## Figures and Tables

**Table 1:** The PD-1/PD-L1 inhibitory activities of the obtained 1,3,5 *m*-terphenyl compounds substituted in meta, *ortho*, and para positions in the HTRF assay.

Name	R^1^	R^2^	R^3^	HTRF ([%) ^1^		IC_50_ Estimated (µM)
				5 µM	0.5 µM	
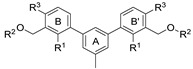						
**4a**	H	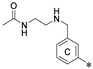	H	56.8 ± 15.4	80.4 ± 18.8	7.72
**4b**	H	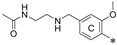	H	62.1 ± 8.4	97.5 ± 6.0	7.41
**4c**	H	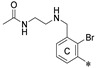	H	70.8 ± 18.8	88.6 ± 14.0	20.52
**4d**	F	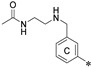	H	22.5 ± 9.17	89.9 ± 19.8	2.08
**4e**	CH_3_	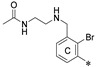	H	94.2 ± 2.3	93.9 ± 5.5	not active
**4f**	CH_3_	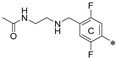	H	28.2 ± 1.5	85.7 ± 10.9	2.18
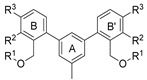						
**5a**	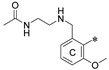	H	H	78.4 ± 6.4	84.3 ± 13.1	36.61
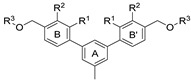						
**6a**	H	OCH_3_	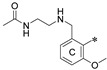	59.2 ± 2.6	86.1 ± 4.5	9.01

^1^ Given HTRF values present the percentage of dissociated PD-1/PD-L1 complex.

**Table 2:** The PD-1/PD-L1 inhibitory activities of the obtained 1,2,3 *m*-terphenyl compounds substituted in para position in the HTRF Assay.

Name	R	R^1^	R^2^	R^3^	HTRF [%] ^1^		IC_50_ Estimated [µM]
					5 µM	0.5 µM	
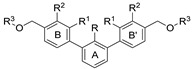							
**7a**	CH_3_	H	OCH_3_	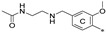	23.8 ± 0.3	72.5 ± 8.1	1.36
**7b ^2^**	CH_3_	H	OCH_3_	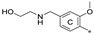	11.9 ± 1.0	60.9 ± 3.9	0.74
**7c**	CH_3_	CH_3_	H	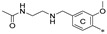	77.1 ± 0.7	82.1 ± 8.0	33.13
**7d**	CH_3_	CH_3_	H	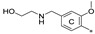	35.7 ± 3.3	86.1 ± 12.7	2.75
**7e**	CH_3_	H	CH_3_	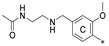	55.3 ± 13.4	93.8 ± 3.6	6.19
**7f**	CH_3_	H	CH_3_	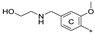	46.4 ± 9.6	93.1 ± 17.4	4.39
**7g**	CH_3_	H	CH_3_	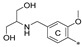	53.7 ± 1.7	94.5 ± 18.1	5.74
**7h**	CH_3_	H	CH_3_	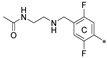	69.0 ± 0.9	84.1 ± 26.4	18.80
**7i**	CH_3_	H	CH_3_	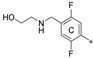	42.2 ± 7.2	78.5 ± 7.3	3.09
**7j ^2^**	CH_3_	H	H	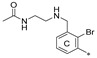	11.1 ± 0.3	40.4 ± 1.5	0.31
**7k**	CH_3_	H	H	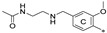	75.1 ± 11.0	95.5 ± 15.2	20.02
**7l**	CH_3_	H	H	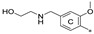	59.8 ± 13.5	97.9 ± 11.2	6.68
**7m ^2^**	Cl	H	H	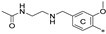	35.0 ± 11.7	51.6 ± 1.6	0.69
**7n**	Cl	H	H	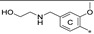	40.0 ± 9.9	67.1 ± 14.1	2.04

^1^ Given HTRF values present the percentage of dissociated PD-1/PD-L1 complex; ^2^ for compounds **7b**, **7j**, and **7m,** IC_50_ values were determined in experiments performed at six different inhibitor concentrations (ranging from 0.1 to 50 µM), employing two independent measurements and Hill curve fitting.

**Table 3:** The PD-1/PD-L1 inhibitory activities of the obtained 1,2,3 *m*-terphenyl compounds substituted in meta position in the HTRF assay.

Name	R	R^1^	R^2^	R^3^	HTRF [%] ^1^		IC_50_ Estimated [µM]
					5 µM	0.5 µM	
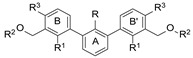							
**8a**	CH_3_	H	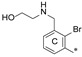	H	23.0 ± 0.1	94.6 ± 0.1	2.43
**8b**	CH_3_	H	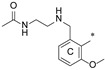	H	73.6 ± 5.3	88.9 ± 3.8	25.1
**8c**	CH_3_	H	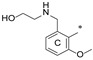	H	61.1 ± 4.5	103.9 ± 3.3	5.96

^1^ Given HTRF values present the percentage of dissociated PD-1/PD-L1 complex.

## Data Availability

The data presented in this study are available on request from the corresponding authors.

## Supplementary Materials

The following supporting information can be downloaded at: https://www.mdpi.com/article/10.3390/molecules29112646/s1, Scheme S1: Synthesis pathway leading to *meta*-1,3,5-*m*-terphenyl derivatives **4a**–**4f**; Scheme S2*:* Synthesis pathway leading to *ortho*- and *para*-1,3,5-*m*-terphenyl derivatives **5a** and **6a**; Scheme S3*:* Synthesis pathway leading to *para*-1,2,3-*m*-terphenyl derivatives **7a**–**7n**; Scheme S4*:* Synthesis pathway leading to *meta*-1,2,3 m-terphenyl derivatives; Table S1: Final compounds structures; Table S2: Molecular docking score and α and β angle values measured for the final compounds and analyzed structures; Figure S1: The superposition of native 6VQN ligand and the best-docked pose of the re-docked random starting geometry of Compound A; Figure S2: ^1^H NMR spectra of the aliphatic part of the human PD-L1 protein with compounds **7k** in a molar ratio of 1:1, 1:10 and with BMS1166; Figure S3: Schematic representation of the calculated angles in the halved-compounds and the *m*-terphenyl short derivatives 7NLD and 8R6Q; Figure S4: Analysis of PD-L1; (A) elution profile, (B) SDS-PAGE analysis of the increasing concentration (C) ^1^H NMR spectrum of PD-L1.
