# Supplementary material for: C2-Symmetrical Terphenyl Derivatives as Small Molecule Inhibitors of Programmed Cell Death 1/Programmed Death Ligand 1 Protein–Protein Interaction
Source: Molecules. 2024 Jun 4;29(11):2646. doi: 10.3390/molecules29112646 (PMC11173618; doi:10.3390/molecules29112646)

Supporting Information

C2-symmetrical terphenyl derivatives as small-molecular inhibitors of Programmed Cell Death-1/Programmed Death-Ligand 1 Protein-Protein Interaction

Joanna Klimek[1,2], Oskar Kruc[1,2] , Joanna Ceklarz[1], Beata Kamińska[1,2], Bogdan Musielak[1], Robin van der Straat[3], Alexander Dömling[4], Tad A. Holak[1], Damian Muszak[1], Justyna Kalinowska-Tłuścik[1], Łukasz Skalniak[1*], Ewa Surmiak[1*]

[1] Jagiellonian University, Faculty of Chemistry, Department of Organic Chemistry, Gronostajowa St 2, 30-387 Cracow, Poland

[2] Jagiellonian University, Doctoral School of Exact and Natural Sciences, Prof. St. Łojasiewicza St 11, 30-348, Cracow, Poland

[3] Department of Drug Design, University of Groningen, 9713 AV Groningen, The Netherlands

[4] Institute of Molecular and Translational Medicine, Faculty of Medicine and Dentistry and Czech Advanced Technology and Research Institute, Palackỳ University in Olomouc, Křížkovského 511/8, 779 00 Olomouc, Czech Republic

*Correspondence: ewa.surmiak@uj.edu.pl, lukasz.skalniak@uj.edu.pl

Table of content

1. Supporting schemes, figures and tables. 2

Scheme S1. Synthesis pathway leading to *meta*-1,3,5-*m*-terphenyl derivatives **4a-4f**.[1] 2

Scheme S2. Synthesis pathway leading to *ortho*- and *para*- 1,3,5-*m*-terphenyl derivatives **5a** and **6a**.[2] 3

Scheme S3. Synthesis pathway leading to *para*-1,2,3-*m*-terphenyl derivatives **7a-7n**[3] 4

Scheme S4. Synthesis pathway leading to *meta*-1,2,3 m-terphenyl derivatives[4] 5

Table S1. Final compounds structures 6

Table S2. Molecular docking score and α and β angle values measured for the final compounds and analyzed structures. 10

Figure S1. The superposition of native 6VQN ligand and the best-docked pose of the re-docked random starting geometry of Compound A 12

Figure S2. ^{1}H NMR spectra of the aliphatic part of the human PD-L1 protein with compounds 7k in a molar ratio of 1:1, 1:10 and with BMS1166 13

Figure S3. Schematic representation of the calculated angles in the halved-compounds and the *m*-terphenyl short derivatives 7NLD and 8R6Q. 14

Figure S4. Analysis of PD-L1; A) elution profile, B) SDS-PAGE analysis of the increasing concentration C) ^{1}H NMR spectrum of PD-L1 15

2. Synthesis of the intermediates 16

2.1. General procedure of Suzuki reaction 16

2.2 General procedure for Williamson reaction 20

2.3. General procedure for aldehyde reduction 28

3. Copies of the HTRF plot for compounds 7b, 7j, 7m 33

4. Copies of ^{1}H and ^{13}C NMR spectra and SFC/MS results for final compounds 35

1. Supporting schemes, figures and tables.

Scheme S1. Synthesis pathway leading to *meta*-1,3,5-*m*-terphenyl derivatives **4a-4f**.[1]

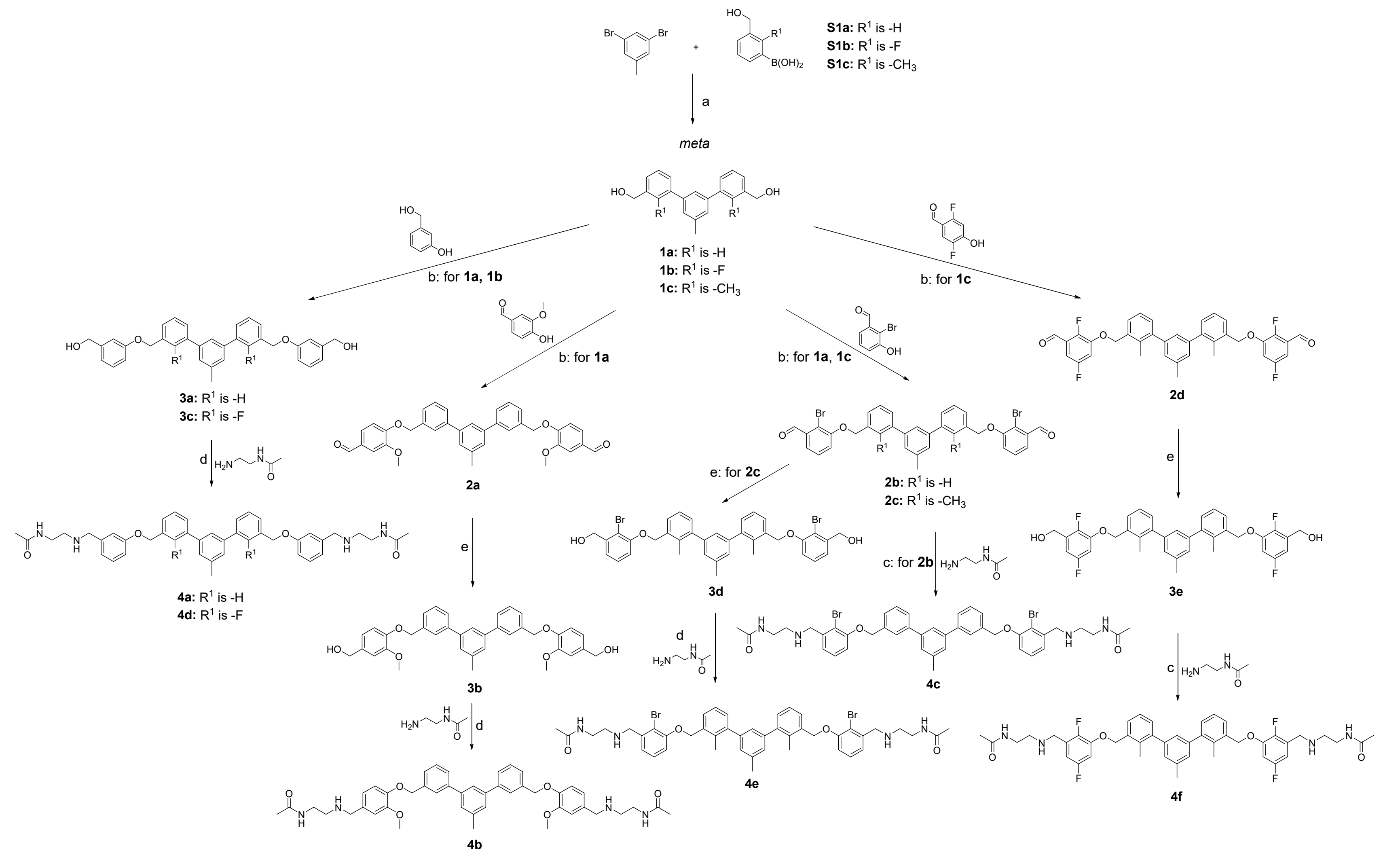

[1]Reagents and conditions a) 3,5-dibromotoluen (1 eq.), borane (2.5 eg.) K_2CO_3 or Cs_2CO_3 (4 eq.), Pd(dppf)Cl_2*DCM orPd(PPh_3)$_4$ (0.01-0.05 eq.),dioxan:H_2O (2:1), 80°C, 4H; b) i: benzyl alcohol (1 eq.), $SOCl_2$ (10 eq.), DMF catal., DCM, RT, 3H ii: benzyl chloride (crude from 1st step), phenol (2-3 eq.), K_2CO_3 or Cs_2CO_3 (4 eq.), DMF, 80°C, o.n.; c) dialdehyde (1 eq.), amine (10 eq.), $NaBH_3CN$ (10 eq.), CH_3COOH catal., DMF, RT, 48H; d) i: dialcohol (1 eq.), $SOCl_2$ (10 eq.), DMF catal., DCM, RT, 2H, ii: benzyl chloride (crude from 1st step), amine (10 eq.), DIPEA (4 eq.), DMF, 80°C, o.n.; e) dialdehyde (1eq.), $NaBH_4$ (2-3 eq.), MeOH:DCM (1:1), RT, o.n

Scheme S2. Synthesis pathway leading to *ortho*- and *para*- 1,3,5-*m*-terphenyl derivatives **5a** and **6a**.[2]

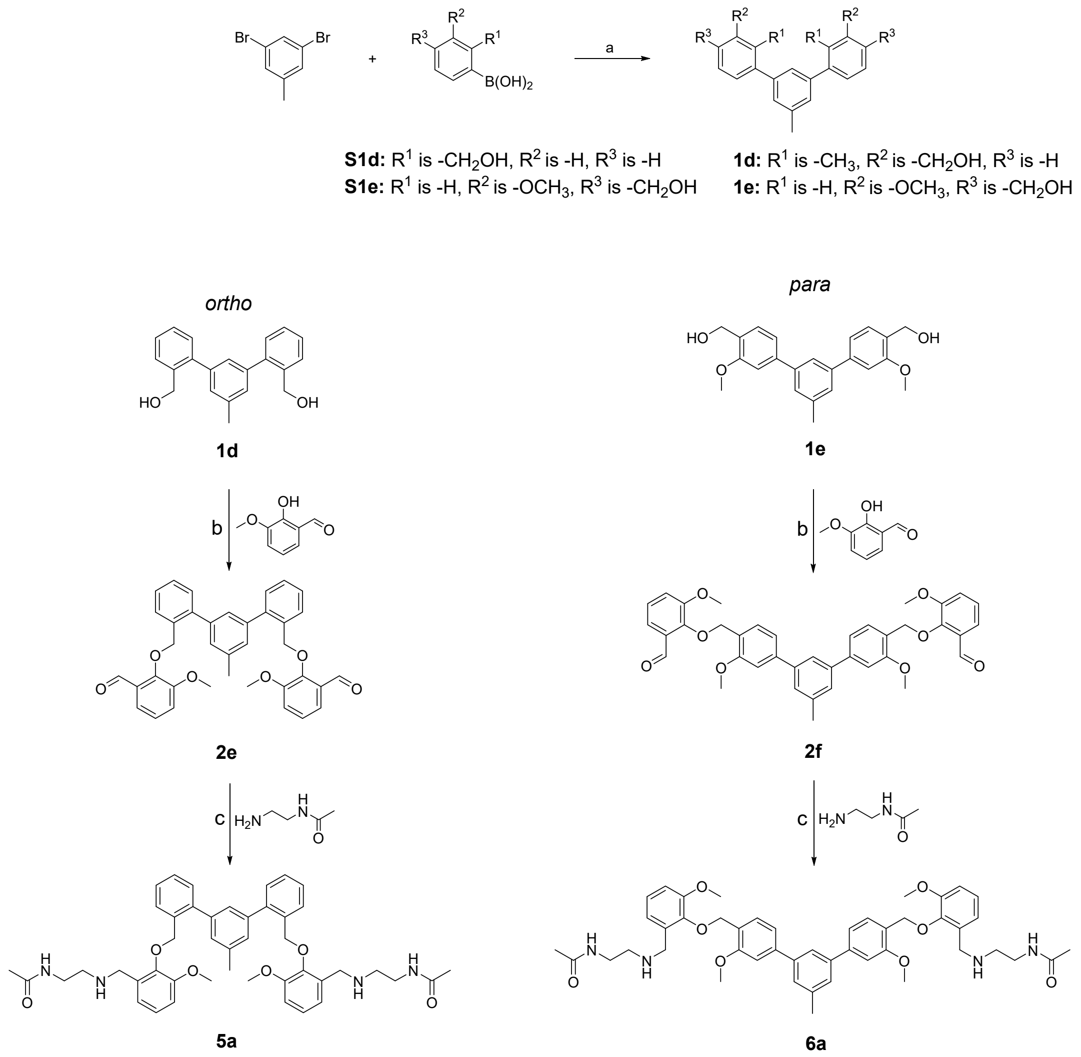

[2]Reagents and conditions a) 3,5-dibromotoluen (1 eq.), borane (2.5 eg.) K_2CO_3 or Cs_2CO_3 (4 eq.), Pd(dppf)Cl_2*DCM orPd(PPh_3)$_4$ (0.01-0.05 eq.),dioxan:H_2O (2:1), 80°C, 4H; b) i: benzyl alcohol (1 eq.), $SOCl_2$ (10 eq.), DMF catal., DCM, RT, 3H ii: benzyl chloride (crude from 1st step), phenol (2-3 eq.), K_2CO_3 or Cs_2CO_3 (4 eq.), DMF, 80°C, o.n.; c) dialdehyde (1 eq.), amine (10 eq.), $NaBH_3CN$ (10 eq.), CH_3COOH catal., DMF, RT, 48H

Scheme S3. Synthesis pathway leading to *para*-1,2,3-*m*-terphenyl derivatives **7a**-**7n**[3]

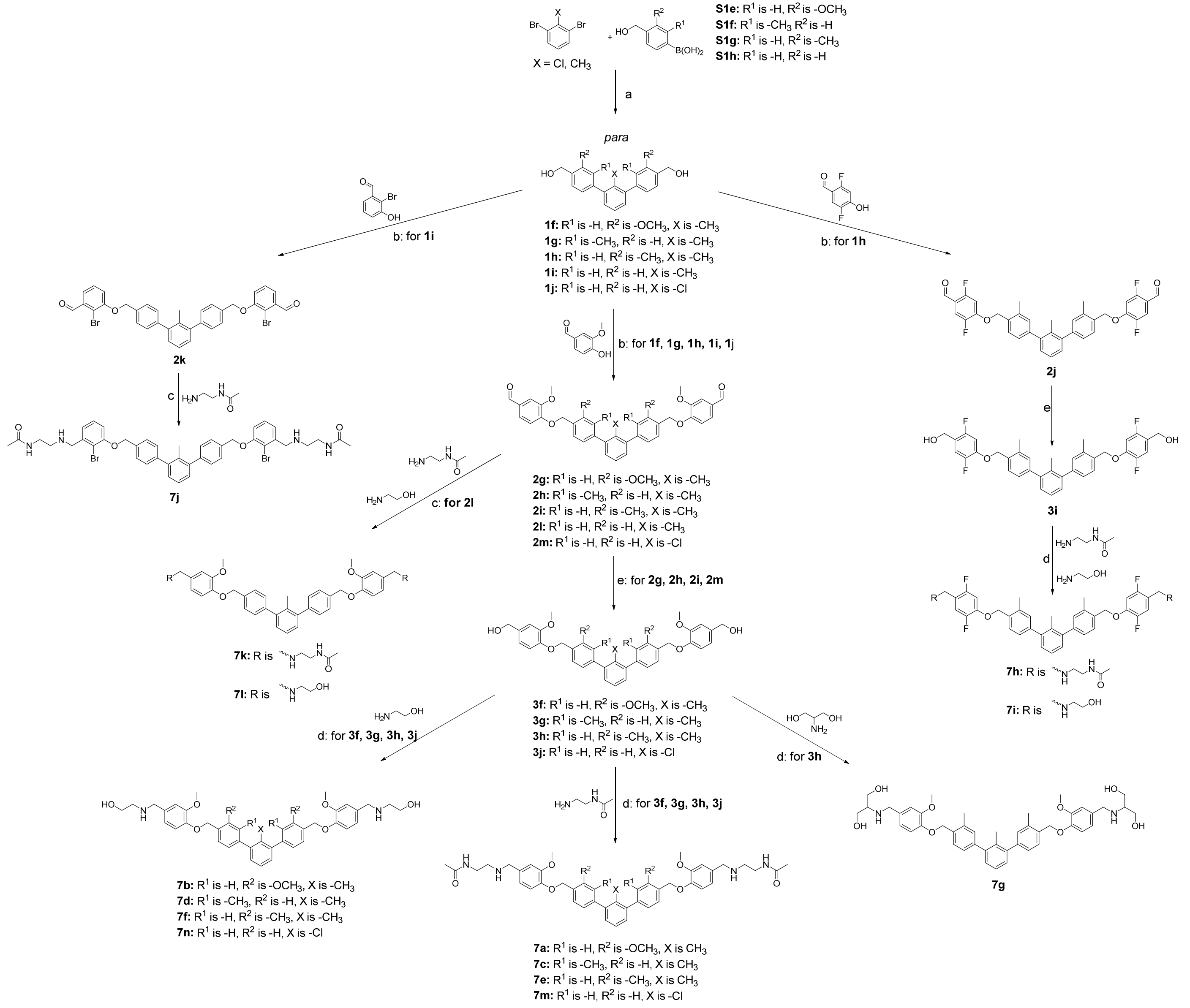

[3]Reagents and conditions a) 2,6-dibromotoluen (1 eq.), borane (2.5 eg.) K_2CO_3 or Cs_2CO_3 (4 eq.), Pd(dppf)Cl_2*DCM or Pd(PPh_3)4 (0.01-0.05 eq.) ,dioxan:H_2O (2:1), 80°C, 4H; b) i: benzyl alcohol (1 eq.), $SOCl_2$ (10 eq.), DMF catal., DCM, RT, 3H, ii: benzyl chloride (crude from 1st step), phenol (2-3 eq.), K_2CO_3 or Cs_2CO_3 (4 eq.), DMF, 80°C, o.n.; c: dialdehyde (1 eq.), amine (10 eq.), $NaBH_3CN$ (10 eq.), CH_3COOH catal., DMF, RT, 48H; d) i: dialcohol (1 eq.), $SOCl_2$ (10 eq.), DMF catal., DCM, RT, 2H, ii: benzyl chloride (crude from 1st step), amine (10 eq.), DIPEA (4 eq.), DMF, 80°C, o.n.; e) dialdehyde (1eq.), $NaBH_4$ (2-3 eq.), MeOH:DCM (1:1), RT, o.n

Scheme S4. Synthesis pathway leading to *meta*-1,2,3 m-terphenyl derivatives[4]

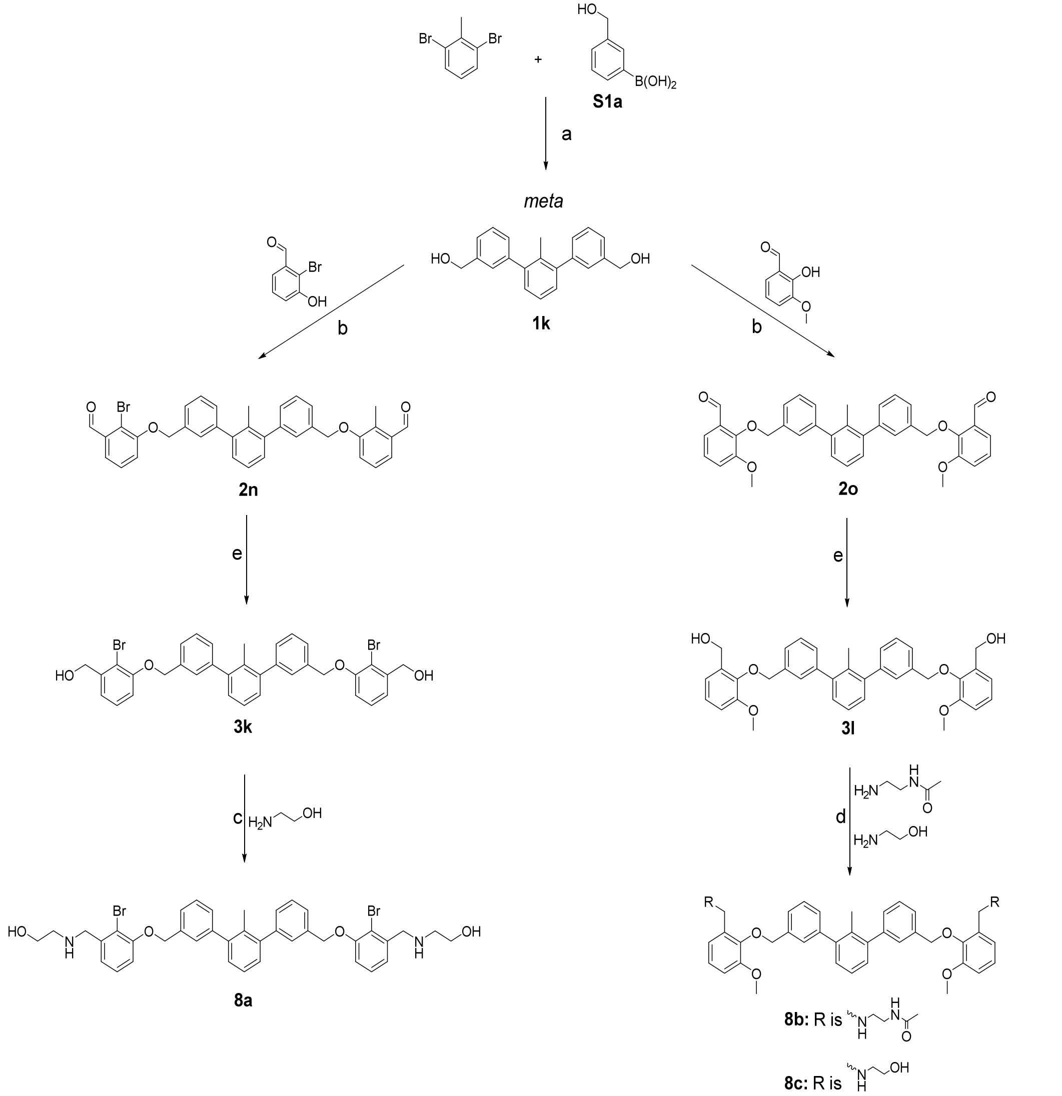

[4]Reagents and conditions a) 2,6-dibromotoluen (1 eq.), borane (2.5 eg.) K_2CO_3 or Cs_2CO_3 (4 eq.), Pd(dppf)Cl_2*DCM or Pd(PPh_3)4 (0.01-0.05 eq.), dioxan:H_2O (2:1), 80°C, 4H; b) i: benzyl alcohol (1 eq.), $SOCl_2$ (10 eq.), DMF catal., DCM, RT, 3H, ii: benzyl chloride (crude from 1st step), phenol (2-3 eq.), K_2CO_3 or Cs_2CO_3 (4 eq.), DMF, 80°C, o.n.; c: dialdehyde (1 eq.), amine (10 eq.), $NaBH_3CN$ (10 eq.), CH_3COOH catal., DMF, RT, 48H; d) i: dialcohol (1 eq.), $SOCl_2$ (10 eq.), DMF catal., DCM, RT, 2H, ii: benzyl chloride (crude from 1st step), amine (10 eq.), DIPEA (4 eq.), DMF, 80°C, o.n.; e) dialdehyde (1eq.), $NaBH_4$ (2-3 eq.), MeOH:DCM (1:1), RT, o.n

Table S1. Final compounds structures

Name	Structure
**4a**	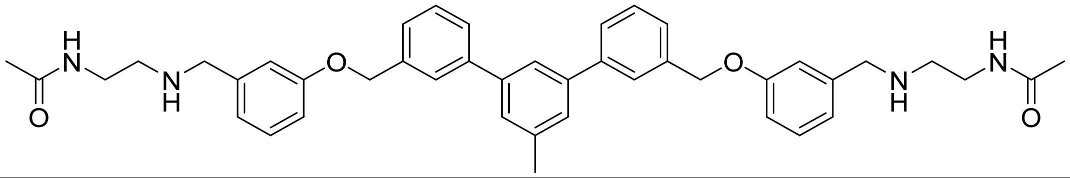
**4b**	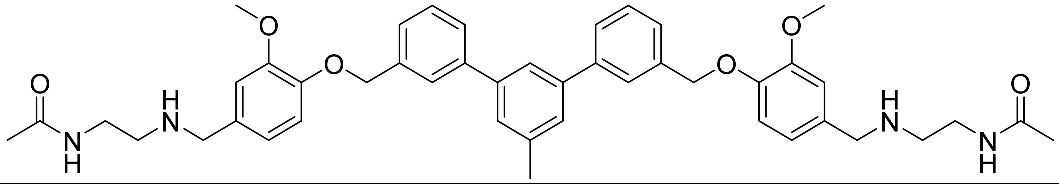
**4c**	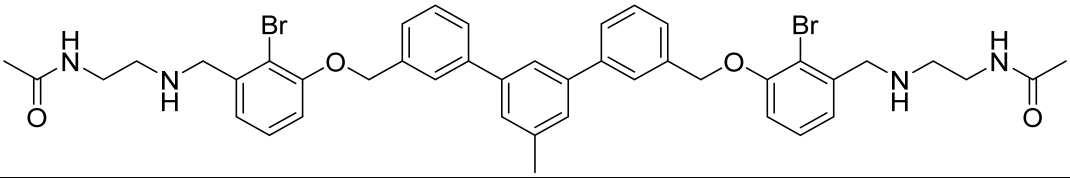
**4d**	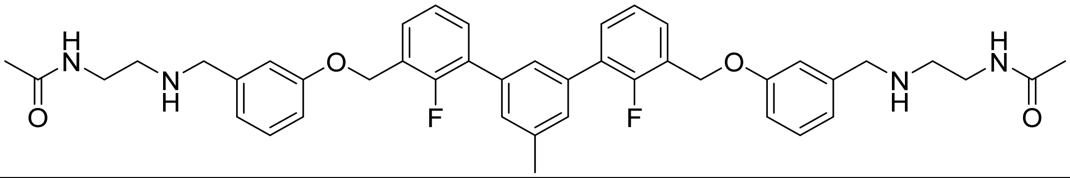
**4e**	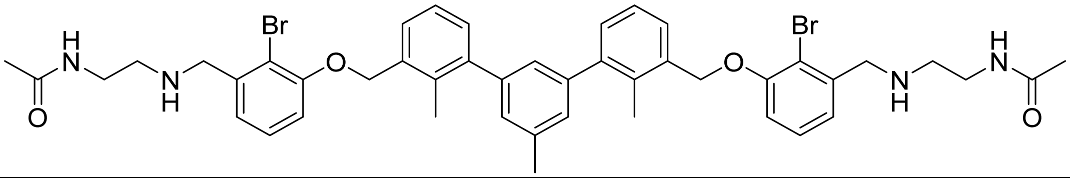
**4f**	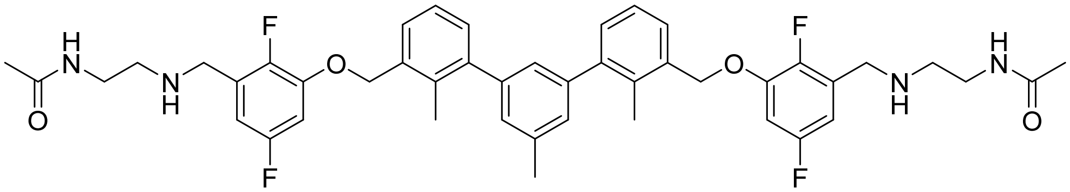
**5a**	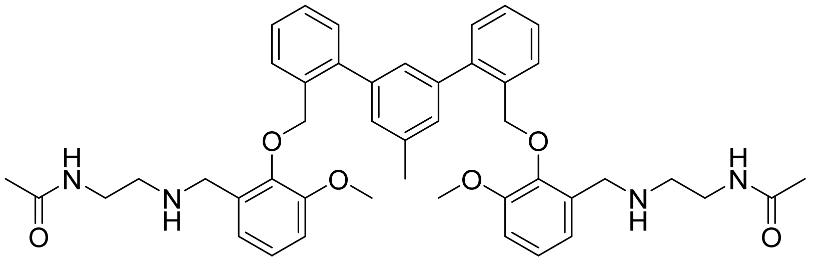
**6a**	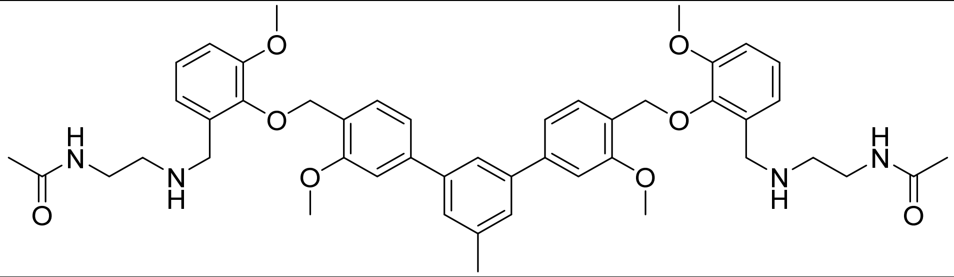

**7a**	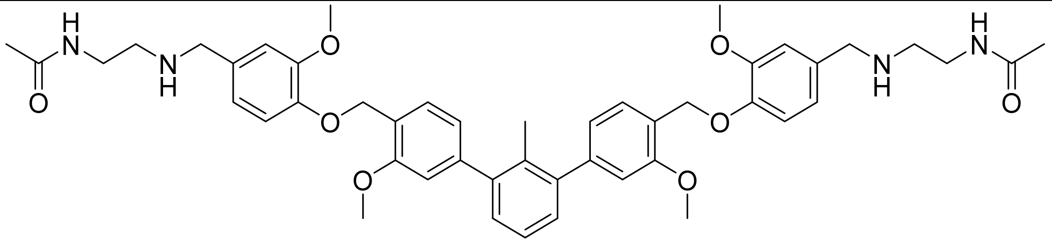
**7b**	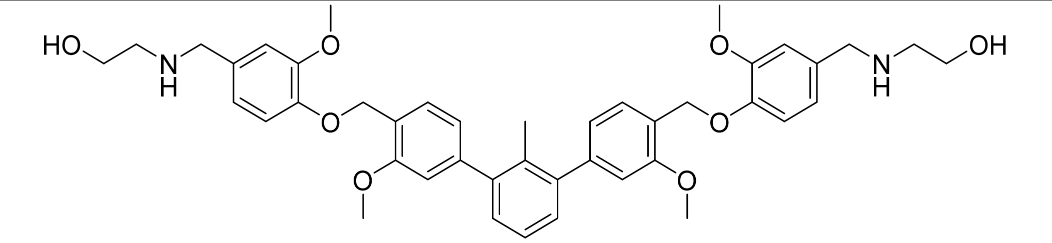
**7c**	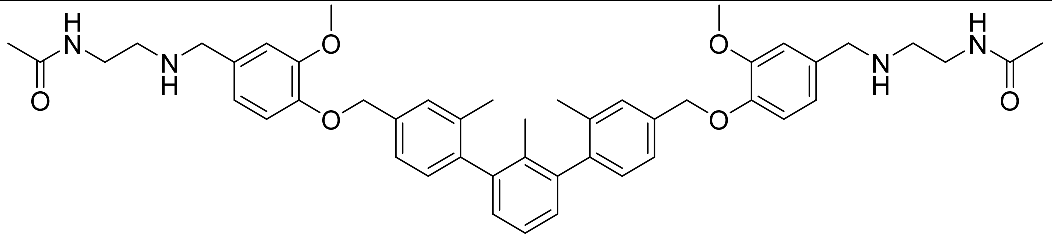
**7d**	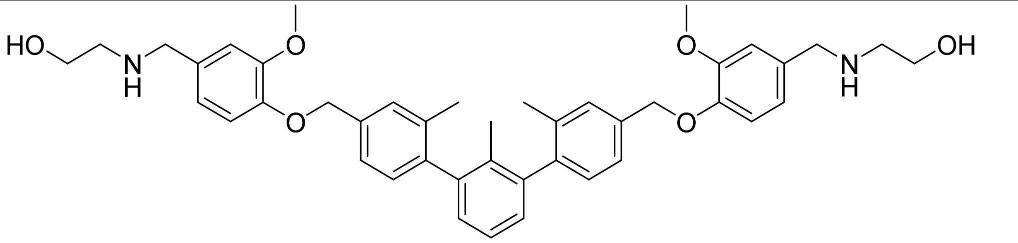
**7e**	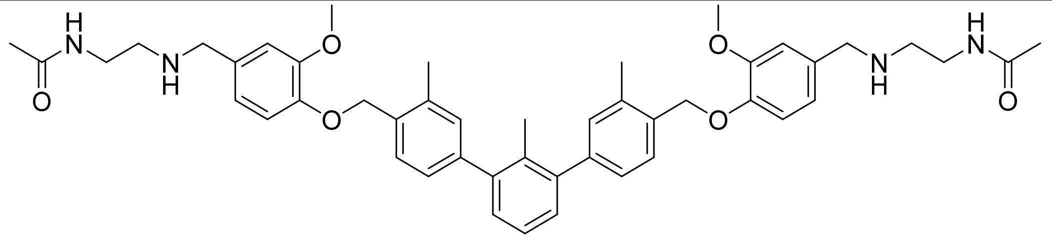
**7f**	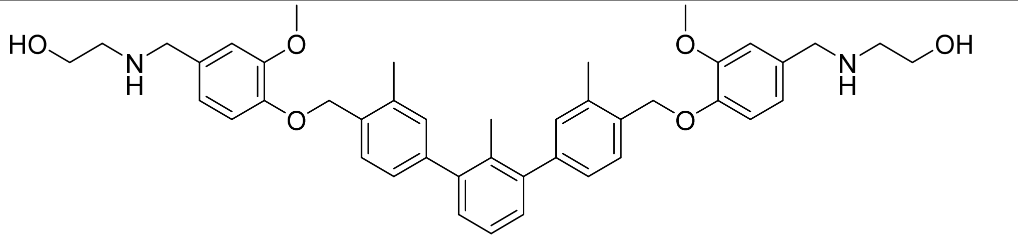
**7g**	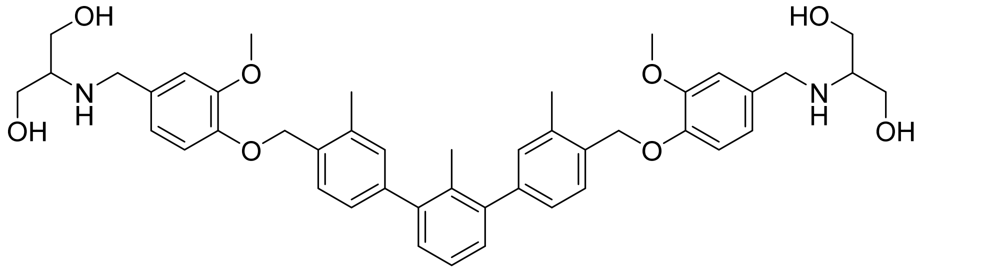

**7h**	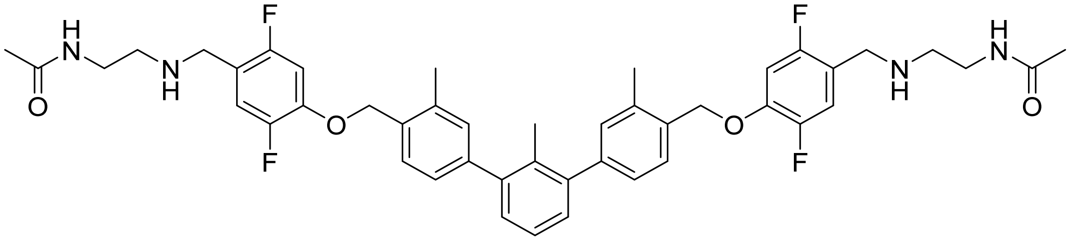
**7i**	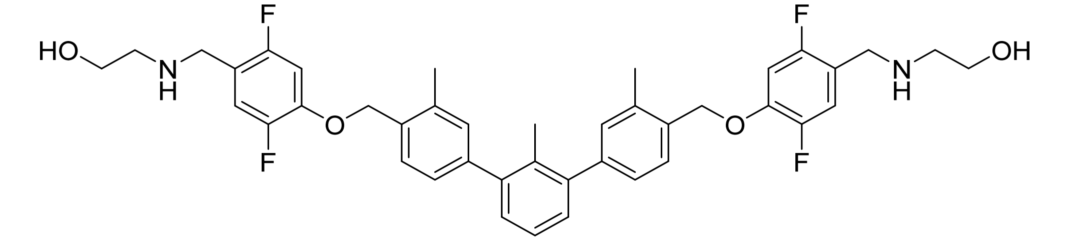
**7j**	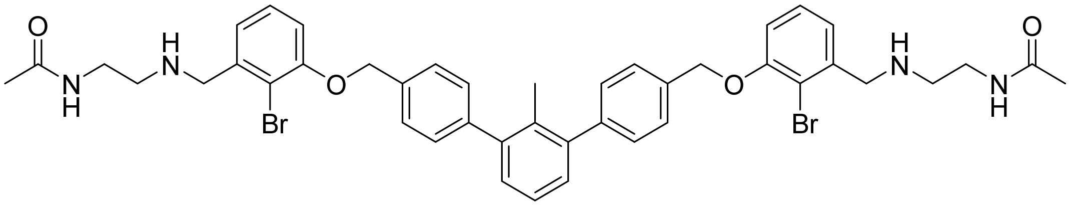
**7k**	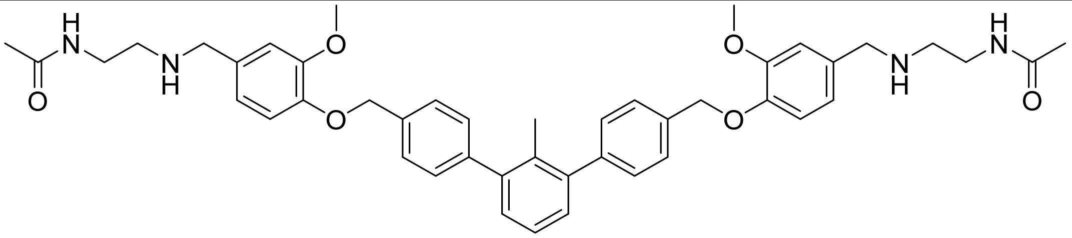
**7l**	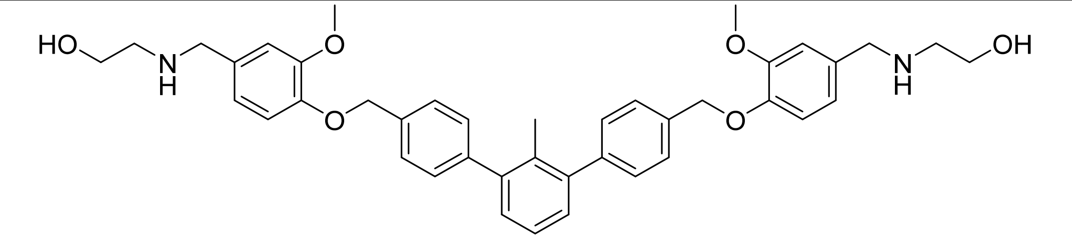
**7m**	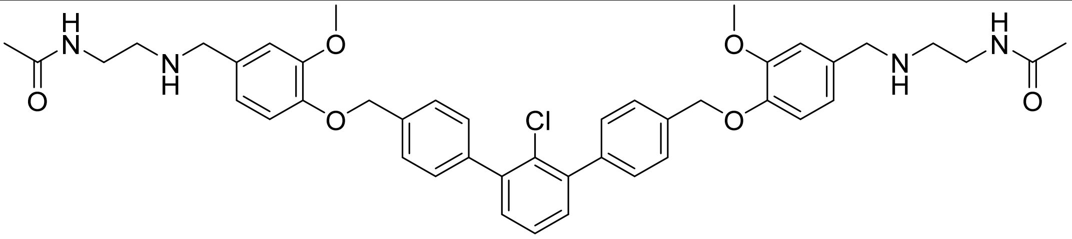
**7n**	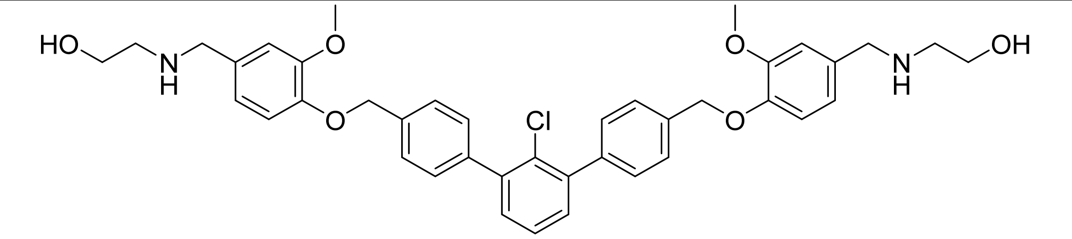
**8a**	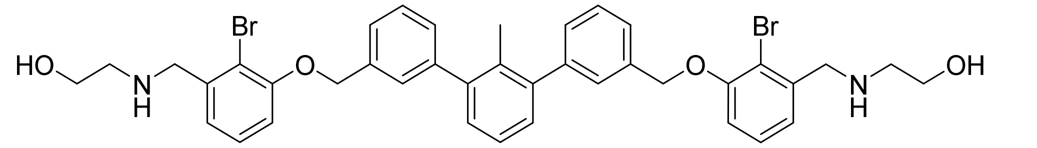

**8b**	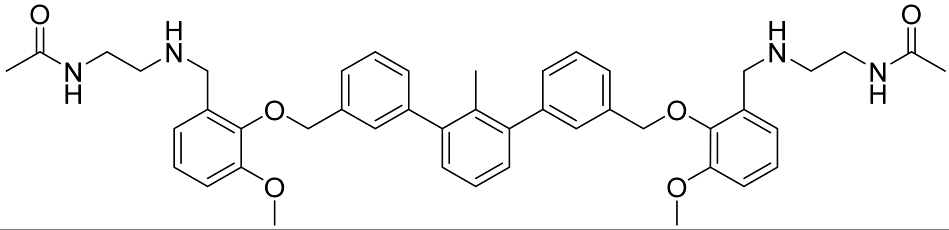
**8c**	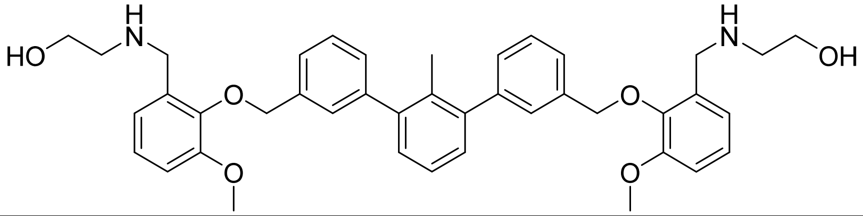
**5b**[5]	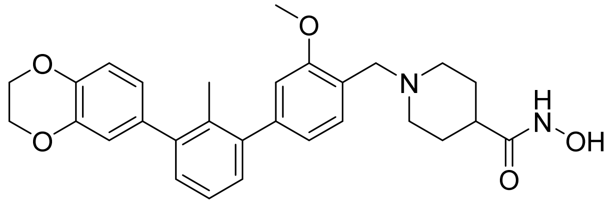
**4a**[6]	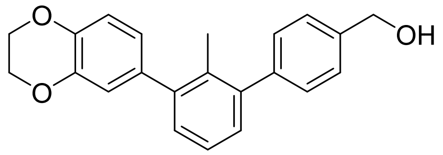

[5]Surmiak, E.; Ząber, J.; Plewka, J.; Wojtanowicz, G.; Kocik-Krol, J.; Kruc, O.; Muszak, D.; Rodríguez, I.; Musielak, B.; Viviano, M.; Castellano, S.; Skalniak, L.; Magiera-Mularz, K.; Holak, T. A.; Kalinowska-Tłuścik, J. Solubilizer Tag Effect on PD-L1/Inhibitor Binding Properties for m-Terphenyl Derivatives. *ACS Med. Chem. Lett.* **2024**, *15* (1), 36–44.; https://doi.org/10.1021/acsmedchemlett.3c00306.
[6]Muszak, D.; Surmiak, E.; Plewka, J.; Magiera-Mularz, K.; Kocik-Krol, J.; Musielak, B.; Sala, D.; Kitel, R.; Stec, M.; Weglarczyk, K.; Siedlar, M.; Dömling, A.; Skalniak, L.; Holak, T. A. Terphenyl-Based Small-Molecule Inhibitors of Programmed Cell Death-1/Programmed Death-Ligand 1 Protein-Protein Interaction. *J. Med. Chem.* **2021**, *64* (15), 11614–11636. https://doi.org/10.1021/acs.jmedchem.1c00957.

Table S2. Molecular docking score and α and β angle values measured for the final compounds and analyzed structures.

Name	Docking score (ChemPLP)	Angle α	Angle β	α + β
		***meta*-(1,3,5): (4a-4f)**		
**4a**	139,74	46,05	34,99	81,04
**4b**	140,25	77,45	56,90	134,35
**4c**	151,58	75,48	23,42	98,90
**4d**	145,28	55,95	41,68	97,63
**4e**	145,09	82,33	39,64	121,97
**4f**	144,24	87,26	22,88	110,14
		***ortho*-(1,3,5): (5a)**		
**5a**	75.86	77,17	42,38	119,55
		***para*-(1,3,5): (6a)**		
**6a**	154,24	85,29	21,02	106,31
		***para*-(1,2,3): (7a-7n)**		
**7a**	143,28	86,36	26,32	112,68
**7b**	148,94	77,68	18,12	95,80
**7c**	152,38	86,08	20,07	106,15
**7d**	139,57	77,08	28,67	105,75
**7e**	152,36	74,33	15,27	89,60
**7f**	149,52	88,41	7,17	95,58
**7g**	162,33	70,10	25,12	95,22
**7h**	156,26	83,36	17,09	100,45
**7i**	141,23	86,66	16,24	102,90
**7j**	158,74	86,49	17,90	104,39
**7k**	169,63	81,14	9,20	90,34
**7l**	151,74	72,32	23,47	95,79
**7m**	154,42	83,43	8,89	92,32
**7n**	154,46	79,13	18,99	98,12
		***meta*-(1,2,3): (8a-8c, *m*-7j)[7]**		
**8a**	153,85	79,12	23,44	102,56
**8b**	117,36	63,11	52,99	116,10
**8c**	96,08	58,19	47,43	105,62
***m*-7j**	142,07	85,59	52,22	137,81
		***ortho*-(1,2,3): (*o*-7j)[7]**		
***o*-7j**	96,77	66,18	64,18	130,36
		**"halved" *meta*-(1,3,5): (4a-half – 4f-half)**		
**4a-half**	132,32	78,14	31,56	109,70
**4b-half**	131,1	66,85	30,20	97,05
**4c-half**	129,82	86,66	28,46	115,12
**4d-half**	136,16	81,97	37,26	119,23

**4e-half**	97,86	19,46	5,93	25,39
**4f-half**	114,74	58,16	47,19	105,35
**"halved"** ***ortho*-(1,3,5): (5a-half)**				
**5a-half**	103,12	14,81	8,85	23,66
**"halved"** ***para*-(1,3,5): (6a-half)**				
**6a-half**	98,08	10,28	4,96	15,24
**"halved"** ***para*-(1,2,3): (7a-half – 7n-half)**				
**7a-half**	122,66	51,78	9,54	61,32
**7b-half**	137,4	75,59	23,19	98,78
**7c-half**	129,54	84,27	31,31	115,58
**7d-half**	118,08	71,08	29,18	100,26
**7e-half**	134,93	79,31	46,75	126,06
**7f-half**	123,83	49,33	29,88	79,21
**7g-half**	141,13	80,62	25,60	106,22
**7h-half**	130,26	84,25	19,61	103,86
**7i-half**	122,72	80,62	27,36	107,98
**7j-half**	130,69	89,73	24,03	113,76
**7k-half**	127,51	47,10	33,27	80,37
**7l-half**	124,92	75,03	28,58	103,61
**7m-half**	127,78	79,80	19,62	99,42
**7n-half**	123,41	72,39	13,72	86,11
**"halved"** ***meta*-(1,2,3): (8a-half – 8c-half,** ***m*-7j-half)**				
**8a-half**	139,09	84,88	38,57	123,45
**8b-half**	118,98	56,75	31,80	88,55
**8c-half**	85,95	18,98	19,15	38,13
**m-7j-half**	135	85,82	42,66	128,48
**"halved"** ***ortho*-(1,2,3): (*o*-7j-half)**				
**o-7j-half**	97,84	63,44	49,85	113,29
**Reference compounds**				
**native_cmpdA**	163,82	77,84		
**random_cmpdA**	146,03	59,90		
**native_7NLD**	116,43	78,16	17,67	95,83
**random_7NLD**	117,69	79,51	16,39	95,90

[7]compounds **m-7j** and **o-7j** were modeled as references for the compounds **7j** with different positioning of the distal aryl rings.

Figure S1. The superposition of native 6VQN ligand (red) and the best-docked pose of the re-docked random starting geometry of Compound A (blue*)*

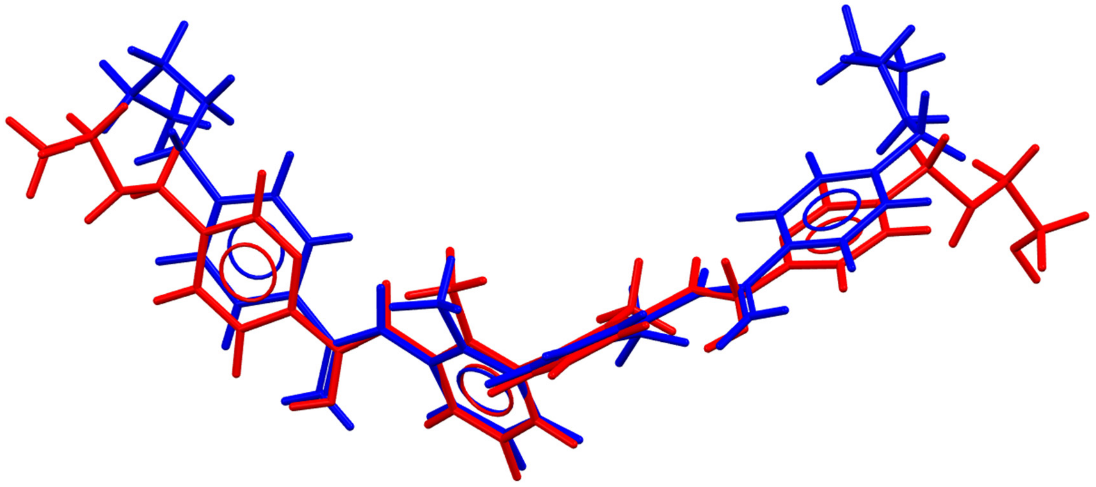

Figure S2. ^{1}H NMR spectra of the aliphatic part of the human PD-L1 protein (blue) with compounds 7k in a molar ratio of 1:1 (green), 1:10 (purple) and with BMS1166 in molar ratio 1:1 (red) used as a positive control.

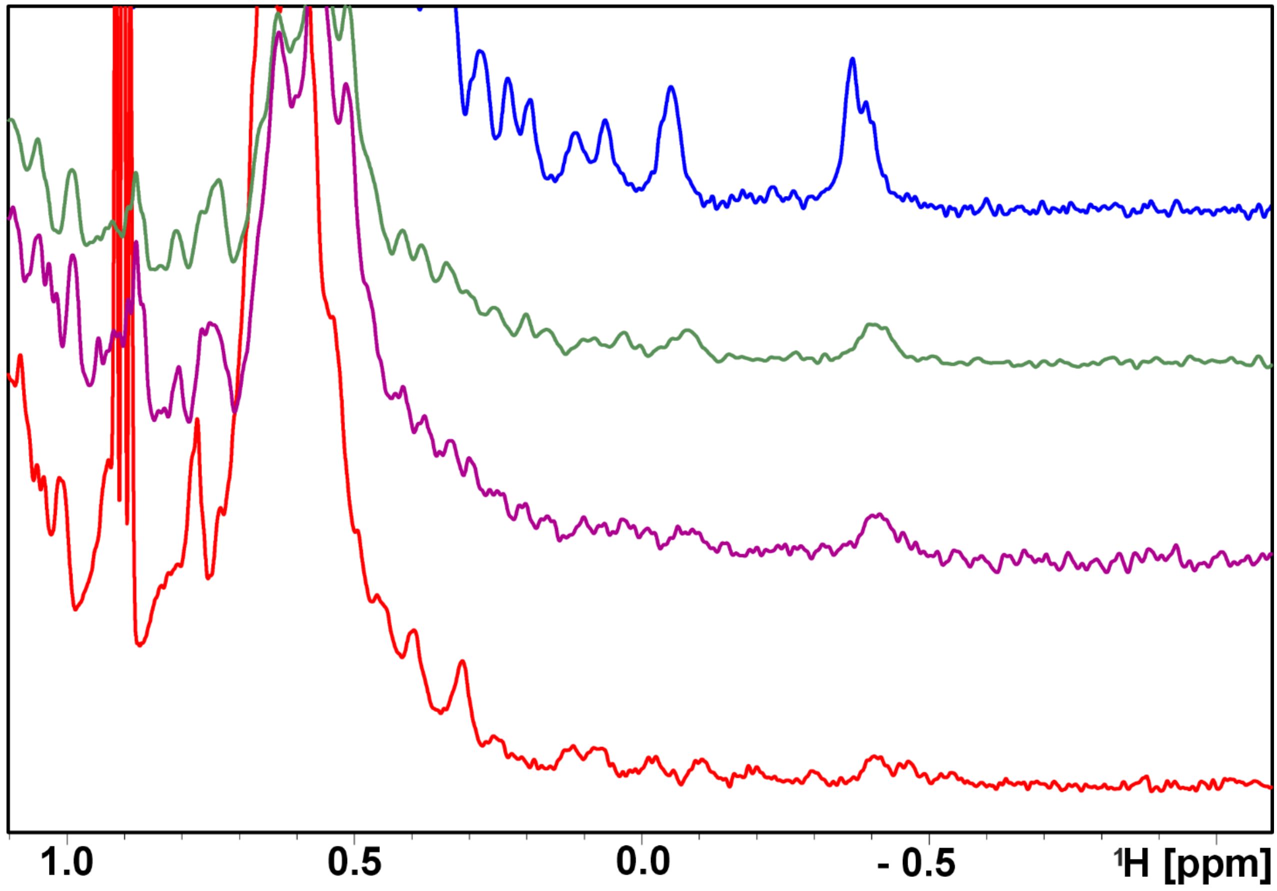

Figure S3. Schematic representation of the calculated angles in the halved-compounds and the *m*-terphenyl short derivatives 7NLD and 8R6Q. The blue color highlights α angle corresponding to the biphenyl core of Compound A, while the yellow color highlights β angle corresponding to one of the pyridine atoms of the Compound A

.

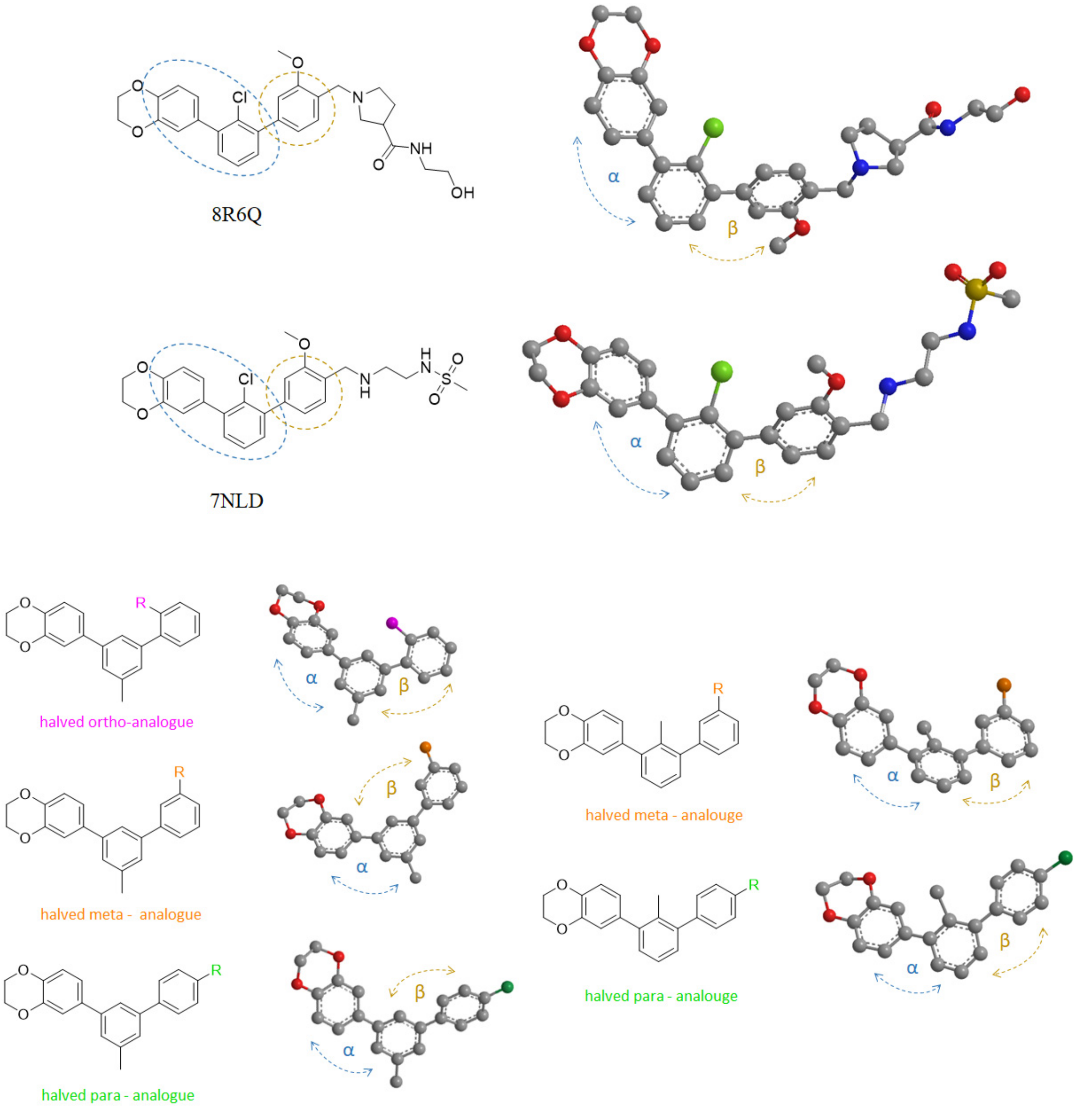

Figure S4. Analysis of PD-L1; A) elution profile, B) SDS-PAGE analysis of the increasing concentration C) ^{1}H NMR spectrum of PD-L1. Intense signals at ca. 3.30 ppm come from the TRIS buffer.

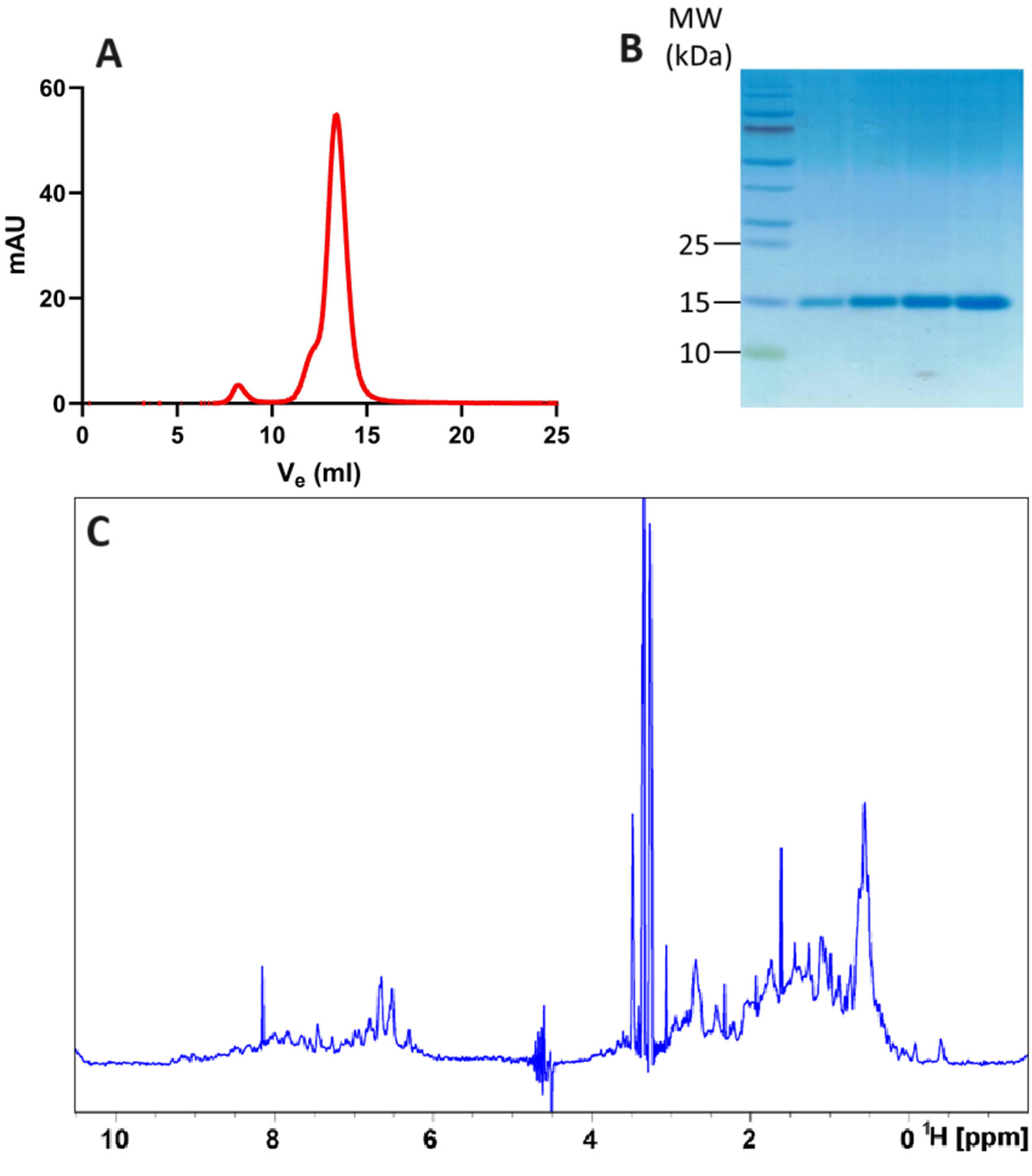

2. Synthesis of the intermediates

2.1. General procedure of Suzuki reaction

The three-neck round-bottom flask was charged with 3,5-dibromotoluene or 2,6-dibromotoluene (1 eq.), appropriate borane **(S1a-S1h)** (2.5 eq), K_2CO_3 (4 eq.), and dioxane/water mixture (2:1, 7/14 ml for 1 mmol) under argon atmosphere. The mixture was deoxygenated by rinsing with argon for half an hour, then palladium complex (Pd(dppf)Cl_2 complex with DCM or Pd(PPh_3)$_4$ was added (0.01-0.05 eq.) Reaction mixture was heated in 80°C, using preheated bath, for four hours and after this time the progression of reaction was controlled using TLC analysis (SiO_2, hexane/ethyl acetate, 4:1). When the reaction was completed the water was added and the extraction with ethyl acetate follows. Organic phases were combined, dried over anhydrous $MgSO_4$ and evaporated. Crude product was purified by column or flash chromatography giving final products **1a-1k** with 29-93% yield.

(5'-methyl-[1,1':3',1''-terphenyl]-3,3''-diyl)dimethanol (1a)

3,5-dibromotoluene (1.25 g, 5.0 mmol, 1.0 eq.), (3-(hydroxymethyl)phenyl)boronic acid **(S1a)** (1.89 g, 12.5 mmol, 2.5 eq), Pd(dppf)Cl_2 complex with DCM (0.06 g, 0.07 mmol, 0.014 eq.), K_2CO_3 (2.76 g, 20.0 mmol, 4.0 eq.). Crude product was purified by flash chromatography (SiO_2, hexane to hexane/ethyl acetate, 1:2) giving product **1a** as colorless solid with 93% (2,34 g).

R_f = 0.30 (SiO_2, hexane/ethyl acetate, 1:1); **^{1}H NMR** (600 MHz, DMSO-d_6) δ [ppm]: 7.40 (t, J = 7.9 Hz, 2H), 7.34-7.29 (m, 5H), 7.24 (d, J = 7.6 Hz, 2H), 7.19 (d, J = 7.6 Hz, 2H), 5.23 (t, J = 5.8 Hz, 2H), 4.56 (d, J = 5.8 Hz, 4H), 2.05 (s, 3H); **^{13}C NMR** (151 MHz, $CDCl_3$) δ [ppm]: 142.9, 142.8, 140.9, 133.0, 129.2, 128.8, 128.5, 128.1, 125.6, 125.5, 65.5, 18.9

(2,2''-difluoro-5'-methyl-[1,1':3',1''-terphenyl]-3,3''-diyl)dimethanol (1b)

3,5-dibromotoluene (1.00 g, 4.0 mmol, 1.0 eq.), (2-fluoro-3-(hydroxymethyl)phenyl)boronic acid **(S1b)** (2.50 g, 10.0 mmol, 2.5 eq.), Pd(PPh_3)$_4$ (0.21 g, 0.18 mmol, 0.05 eq), K_2CO_3 (1.56 g, 15.9 mmol, 4.0 eq.). Crude product was purified by flash chromatography (SiO_2, hexane to hexane/ethyl acetate, 1:2) giving product **1b** as colorless solid with 61% (0.82 g).

R_f = 0.80 (SiO_2, hexane/ethyl acetate, 1:2)

^{1}H NMR (600 MHz, $CDCl_3$) δ [ppm]: 7.51 (s, 1H), 7.45-7.38 (m, 4H), 7.37 (s, 2H), 7.22 (t, J = 7.6 Hz, 2H), 4.83 (s, 4H), 2.47 (s, 3H); **^{13}C NMR** (151 MHz, $CDCl_3$) δ [ppm]: 158.5, 156.9,

138.4, 135.9, 130.5, 130.5, 129.3, 128.5, 128.5, 127.2, 124.5, 124.4, 59.8, 59.8, 21.7; **IR (ATR)** [cm^{-1}]: 3287, 2981, 2915, 1600, 1464, 1410, 1363, 1196, 1058, 825, 770, 729, 701

(2,2'',5'-trimethyl-[1,1':3',1''-terphenyl]-3,3''-diyl)dimethanol (1c)

3,5-dibromotoluene (1.29 g, 5.2 mmol, 1.0 eq.), (2-metylo-3-(4,4,5,5-tetramethyl-1,3,2-dioxaborolan-2-yl)phenyl)methanol **(S1c)** (3.20 g, 13.90 mmol, 2.5 eq.), Pd(dppf)Cl_2 complex with DCM (0.21 g, 0.26 mmol, 0.03 eq.), K_2CO_3 (2.02 g, 20.6 mmol, 4.0 eq.). Crude product was purified by flash chromatography (SiO_2, hexane to hexane/ethyl acetate, 1:2) giving final product **1c** as colorless solid with 29% (0.79 g).

R_f = 0.80 (SiO_2, hexane/ethyl acetate, 1:2); **^{1}H NMR** (600 MHz, $CDCl_3$) δ [ppm]: 7.40 (dd, J = 6.7, 2.2 Hz, 2H), 7.32-7.23 (m, 4H), 7.12 (bs, 2H), 7.06 (bs, 1H), 4.78 (s, 4H), 2.46 (s, 3H), 2.31 (s, 6H); **^{13}C NMR** (151 MHz, $CDCl_3$) δ [ppm]: 142.9, 141.8, 139.4, 137.6, 133.7, 129.7, 128.7, 127.8, 126.9, 125.7, 64.2, 21.6, 16.1; **IR (ATR)** [cm^{-1}]: 3216, 2918, 2884, 1563, 1439, 1251, 1169, 1135, 1013, 854, 776, 717

(5'-methyl-[1,1':3',1''-terphenyl]-2,2''-diyl)dimethanol (1d)

3,5-dibromotoluene (2.50 g, 10.0 mmol, 1.0 eq.), (2-(hydroxymethyl)phenyl)boronic acid **(S1d)** (3.80 g, 25.0 mmol, 2.5 eq.) Pd(dppf)Cl_2 complex with DCM (0.40 g, 0.5 mmol, 0.05 eq.), K_2CO_3 (5.52 g, 40.0 mmol, 4.0 eq.). Crude product was purified by flash chromatography (SiO_2, hexane to hexane/ethyl acetate, 1:2) giving final product **1d** as yellowish solid with 77% (2.34 g).

R_f = 0.38 (SiO_2, hexane/ethyl acetate, 1:1); **^{1}H NMR** (600 MHz, DMSO-d_6) δ [ppm]: 7.58 (d, J = 7.6 Hz, 2H), 7.38 (td, J = 7.5, 1.3 Hz, 2H), 7.33 (td, J = 7.4, 1.2 Hz, 2H), 7.27 (dd, J = 7.6, 1.2 Hz, 2H), 7.18 (d, J = 6.4 Hz, 3H), 5.16 (t, J = 5.4 Hz, 2H), 4.47 (d, J = 5.4 Hz, 4H), 2.41 (s, 3H); **^{13}C NMR** (101 MHz, DMSO-d_6) δ [ppm]: 140.6, 140.5, 139.8, 137.9, 129.9, 129.0, 128.6, 127.8, 127.4, 127.3, 61.3, 21.6; **IR (ATR)** [cm^{-1}]: 3222, 2919, 2863, 1595, 1447, 1196, 1033, 1006, 868, 751, 717

(3,3''-dimethoxy-5'-methyl-[1,1':3',1''-terphenyl]-4,4''-diyl)dimethanol (1e)

3,5-dibromotoluene (0.75 g, 3.0 mmol, 1.0 eq), (4-(hydroxymethyl)-3-methoxyphenyl)boronic acid (**S1e**) (1.67 g, 6.3 mmol, 2.1 eq.), Pd(dppf)Cl_2 complex with DCM (0.12 g, 0.15 mmol, 0.05 eq.), K_2CO_3 (1.66 g, 12.0 mmol, 4.0 eq.). Crude product was purified by flash chromatography (SiO_2, hexane to hexane/ethyl acetate, 1:2) giving final product **1e** as yellowish solid with 93% (1.01 g).

R_f = 0.23 (SiO_2, hexane/ethyl acetate, 1:1); **^{1}H NMR** (400 MHz, DMSO-d_6) δ [ppm]: 7.69 (s, 1H), 7.48 (s, 2H), 7.45 (d, J = 7.8 Hz, 2H), 7.30 (d, J = 7.8 Hz, 2H), 7.23 (s, 2H), 5.04 (t, J = 5.6 Hz, 2H), 4.53 (d, J = 5.6 Hz, 4H), 3.88 (s, J = 4.6 Hz, 6H), 2.45 (s, 3H); **^{13}C NMR** (101 MHz, DMSO-d_6) δ [ppm]: 156.9, 141.6, 140.6, 139.1, 130.1, 127.9, 127.0, 126.9, 123.0, 119.2, 109.4, 58.3, 55.9, 21.7; **IR (ATR)** [cm^{-1}]: 3266, 2943, 1574, 1467, 1383, 1236, 1127, 1043, 820, 749

(3,3''-dimethoxy-2'-methyl-[1,1':3',1''-terphenyl]-4,4''-diyl)dimethanol (1f)

2,6-dibromotoluene (1.46 g, 5.9 mmol, 1.0 eq.), (4-(hydroxymethyl)-3-methoxyphenyl)boronic acid **(S1e)** (3.40 g, 12.9 mmol, 2.2 eq), Pd(dppf)Cl_2 complex with DCM (0.24 g, 0.3 mmol, 0.05 eq.), K_2CO_3 (3.23 g, 23.4 mmol, 4.0 eq.). Crude product was purified by column chromatography (SiO_2, hexane to hexane/ethyl acetate, 1:1) giving final product **1f** as yellowish solid with 48% (2.13 g).

R_f = 0.41 (SiO_2, hexane/ethyl acetate, 3:1); **^{1}H NMR** (400 MHz, DMSO-d_6) δ [ppm]: 7.43 (d, *J* = 7.5 Hz, 2H), 7.34 – 7.28 (m, 1H), 7.22 (d, *J* = 7.4 Hz, 2H), 6.94 (d, *J* = 7.4 Hz, 4H), 5.03 (d, *J* = 5.5 Hz, 2H), 4.54 (d, *J* = 5.5 Hz, 4H), 3.81 (s, 6H), 2.10 (s, 3H); **^{13}C NMR** (101 MHz, DMSO-d6) δ [ppm]: 156.2, 143.0, 141.9, 132.9, 129.5, 129.2, 127.3, 127.0, 121.4, 111.7, 58.3, 55.8, 19.2; **IR (ATR)** [cm^{-1}]: 3219, 2936, 2875, 1610, 1571, 1505, 1464, 1393, 1253, 1124, 1043, 1006, 911, 856, 795, 734

(2,2',2''-trimethyl-[1,1':3',1''-terphenyl]-4,4''-diyl)dimethanol (1g)

2,6-dibromotoluene (1.08 g, 4.3 mmol, 1.0 eq.), [4-(hydroxymethyl)-2-methylphenyl]boronic acid **(S1f)** (2.50 g, 9.5 mmol, 2.2 eq.), Pd(dppf)Cl_2 complex with DCM (0.39 g, 0.5 mmol, 0.05 eq.), K_2CO_3 (5.27 g, 38.1 mmol, 4.0 eq.). Crude product was purified by column chromatography (SiO_2, petroleum ether to petroleum ether/ethyl acetate, 1:1) giving final product **1g** as yellowish solid with 49% (0.71 g).

R_f = 0.13 (SiO_2, hexane/ethyl acetate, 1:1); **^{1}H NMR** (400 MHz, DMSO-d_6) δ [ppm] (mixture of rotamers): 7.32 – 7.27 (m, 1H), 7.24 (s, 2H), 7.19 (d, *J* = 7.9 Hz, 2H), 7.10 – 7.04 (m. 4H), 5.18 (t, *J* = 5.7 Hz, 2H), 4.51 (d, *J* = 5.7 Hz, 4H), 2.03 and 2.01 (singlets, 6H, from rotamers), 1.64 and 1.63 (singlets, 3H, from rotamers); **^{13}C NMR** (101 MHz, DMSO-d_6) δ [ppm] (mixture of rotamers) : 142.1, 142.1, 142.0, 142.0, 140.4, 140.3, 135.3, 135.3, 133.7, 133.5, 129.4, 129.1, 128.6, 128.5, 126.1, 125.9, 124.5, 124.5, 74.0, 63.3, 25.5, 20.1, 19.9, 17.7, 17.5; **IR (ATR)** [cm^{-1}]: 3301, 2978, 2919, 2873, 2362, 2343, 1435, 1380, 1158, 1022, 833, 738

(2',3,3''-trimethyl-[1,1':3',1''-terphenyl]-4,4''-diyl)dimethanol (1h)

2,6-dibromotoluene (0.88 g, 3.5 mmol, 1.0 eq.), [4-(hydroxymethaneyl)-3-methylphenyl]boronic acid **(S1g)** (2.03 g, 7.7 mmol, 2.2 eq.), Pd(dppf)Cl_2 complex with DCM (0.32 g, 0.4 mmol, 0.05 eq.), K_2CO_3 (4.27 g, 30.9 mmol, 4.0 eq.). Crude product was purified by column chromatography (SiO_2, petroleum ether to petroleum ether/ethyl acetate, 1:1) giving final product **1h** as yellowish solid with 59% (0.69 g).

R_f = 0.13 (SiO_2, hexane/ethyl acetate, 3:1); **^{1}H NMR** (600 MHz, DMSO-d_6) δ [ppm]: 7.41 (d, J = 7.7 Hz, 2H), 7.28 (t, J = 7.6 Hz, 1H), 7.20 – 7.11 (m, 6H), 5.09 (t, J = 5.4 Hz, 2H), 4.53 (d, J = 5.4 Hz, 4H), 2.29 (s, 6H), 2.05 (s, 3H); **^{13}C NMR** (151 MHz, DMSO-d_6) δ [ppm]: 142.9, 140.7, 139.4, 135.4, 132.6, 130.9, 129.0, 127.1, 126.7, 125.9, 74.0, 61.4, 25.4; **IR (ATR)** [cm^{-1}]: 3288, 2861, 1444, 1364, 1115, 1046, 1011, 835, 808, 774, 719

(2'-methyl-[1,1':3',1''-terphenyl]-4,4''-diyl)dimethanol (1i)

2,6-dibromotoluene (2.50 g, 10.0 mmol, 1.0 eq.), (4-(hydroxymethyl)phenyl)boronic acid **(S1h)** (3.78 g, 25.0 mmol, 2.5 eq.), Pd(dppf)Cl_2 complex with DCM (0.12 g, 0.1 mmol, 0.01 eq.), K_2CO_3 (5.52 g, 40.0 mmol, 4.0 eq.). Crude product was purified by column chromatography (SiO_2, hexane to hexane/ethyl acetate, 5:1) giving final product **1i** as colorless solid with 39% (1.18 g).

R_f = 0.27 (SiO_2, hexane/ethyl acetate, 1:1); **^{1}H NMR** (600 MHz, DMSO-d_6) δ [ppm]: 7.42-7.27 (m, 9H), 7.18 (d, J = 7.4 Hz, 2H), 5.21 (t, J = 5.7 Hz, 2H), 4.55 (d, J = 5.7 Hz, 4H), 2.06 (s, 3H); **^{13}C NMR** (151 MHz, DMSO-d_6) δ [ppm]: 142.3, 141.2, 140.2, 132.2, 128.9, 128.7, 126.4, 125.6, 62.8, 18.6; **IR (ATR)** [cm^{-1}]: 3238, 2864, 1446, 1397, 1039, 851, 790

(2'-methyl-[1,1':3',1''-terphenyl]-4,4''-diyl)dimethanol (1j)

1,3-dibromo-2-chlorobenzene (2.47 g, 9.1 mmol, 1.0 eq.), (4-(hydroxymethyl)phenyl)boronic acid **(S1h)** (3.33 g, 21.8 mmol, 2.4 eq.), Pd(dppf)Cl_2 complex with DCM (0.42 g, 0.5 mmol, 0.06 eq.), K_2CO_3 (5.26 g, 38.1 mmol, 4.2 eq.). Crude product was purified by column chromatography (SiO_2, hexane to hexane/ethyl acetate, 5:1) giving final product **1j** as light yellow solid with 93% (2.75 g).

R_f = 0.15 (SiO_2, hexane/ethyl acetate, 2:1); **^{1}H NMR** (600 MHz, DMSO-d_6) δ [ppm]: 7.46 (dd, J = 8.0, 7.1 Hz, 1H), 7.41 (s, 8H), 7.37 (d, J = 7.5 Hz, 2H), 5.24 (t, J = 5.8 Hz, 2H), 4.56 (d, J = 5.8 Hz, 4H); **^{13}C NMR** (151 MHz, DMSO-d_6) δ [ppm]: 142.0, 141.0, 137.8, 130.4, 129.9,

129.1, 127.1, 126.2, 62.7; **IR (ATR)** [cm^{-1}]: 3221, 2927, 2858, 1514, 1455, 1390, 1265, 1236, 1031, 793

(2'-methyl-[1,1':3',1''-terphenyl]-3,3''-diyl)dimethanol (1k)

2,6-dibromotoluene (3.75 g, 15.0 mmol, 1.0 eq), (3-(hydroxymethyl)phenyl)boronic acid **(S1a)** (5,70 g, 37.5 mmol, 2.5 eq), Pd(dppf)Cl_2 complex with DCM (0.61 g, 0.8 mmol, 0.05 eq), K_2CO_3 (6.22 g, 45.0 mmol, 3.0 eq.). Crude product was purified by flash chromatography (SiO_2, dichloromethane/ethyl acetate, 3:1) giving product **1k** as reddish solid with 92% yield (4.27 g).

R_f = 0.50 (SiO_2, DCM/ethyl acetate, 1:1); **^{1}H NMR** (600 MHz, $CDCl_3$) δ [ppm]: 7.37 – 7.31 (m, 2H), 7.31 – 7.24 (m, 4H), 7.24 – 7.21 (m, 2H), 7.21 – 7.18 (m, 1H), 7.18 – 7.11 (m, 2H), 4.67 (s, 4H), 2.03 (s, 3H); **^{13}C NMR** (151 MHz, DMSO-d_6) δ [ppm]: 143.0, 143.0, 142.0, 132.5, 129.1, 128.4, 127.9, 127.7, 126.0, 125.5, 63.3, 19.1; **IR (ATR)** [cm^{-1}]: 3364, 3035, 2944, 2912, 1606, 1583, 1418, 1340, 1182, 1024, 905, 793, 779, 736, 708, 622

2.2 General procedure for Williamson reaction

Appropriate benzyl alcohol **(1a-1k)** (1 eq.) was dissolved in anhydrous DCM with catalytical amount of anhydrous DMF (0.2 ml per 2 mmol alcohol) in round-bottom flask under argon atmosphere. Solution was cooled in ice bath and $SOCl_2$ (10 eq.) was added. Reaction mixture was stirred in RT for 3 hours. After this time the progression of reaction was controlled by TLC. When the reaction was completed, the mixture was poured into the solution of saturated $NaHCO_3$ and extracted with DCM. Organic phases were combined, dried over anhydrous $MgSO_4$ and evaporated. Crude product was used for the next step without further purification. Appropriate phenol (2.0-3.0 eq.) and K_2CO_3 or Cs_2CO_3 (4 eq.) were dissolved in anhydrous DMF in round-bottom flask under argon atmosphere and stirred for 15 min. Next, crude benzyl chloride obtained in the first step was added as a solution in minimal amount of the anhydrous DMF. Reaction mixture was heated in 80°C overnight. After this time water was added and the extraction with ethyl acetate follows. Organic phases were combined, dried over anhydrous $MgSO_4$ and evaporated. Crude product was purified by column or flash chromatography giving final products **2a-2o**, **3a**, **3c** with 30-87% yield.

4,4'-(((5'-methyl-[1,1':3',1''-terphenyl]-3,3''-diyl)bis(methylene))bis(oxy))bis(2-methoxybenzaldehyde) (2a)

Compound **1a** (0.50 g, 1.6 mmol, 1.0 eq.), $SOCl_2$ (1.20 ml, 16.4 mmol, 10.0 eq), vanillin (0.67 g, 4.93 mmol, 3.0 eq.), Cs_2CO_3 (2.14 g, 6.6, 4.0 eq.). Crude product was purified by flash chromatography (SiO_2, hexane to hexane/ethyl acetate, 1:1) giving product **2a** as colorless solid with 76% (0.72 g).

R_f = 0.60 (SiO_2, hexane/ethyl acetate, 1:1); **^{1}H NMR** (600 MHz, DMSO-d_6) δ [ppm]: 9.84 (s, 2H), 7.55 (dd, J = 8.2, 1.8 Hz, 2H), 7.42 (d, J = 1.8 Hz, 2H), 7.38 (m, 2H), 7.35 (t, J = 7.56 Hz, 1H), 7.30 (d, J = 8.3 Hz, 2H), 7.23 (d, J = 7.6 Hz, 2H), 5.29 (s, 4H), 3.83 (s, 6H), 2.04 (s, 3H); **^{13}C NMR** (151 MHz, DMSO-d_6) δ [ppm]: 191.9, 153.6, 149.9, 142.6, 142.3, 136.8, 132.6, 130.3, 129.39, 129.36, 129.2, 129.0, 127.1, 126.4, 126.3, 113.2, 110.3, 70.4, 56.1, 19.0; **IR (ATR)** [cm^{-1}]: 2962, 2921, 2858, 1692, 1599, 1520, 1388, 1337, 1275, 1240, 1168, 1122, 1025, 784

3,3'-(((5'-methyl-[1,1':3',1''-terphenyl]-3,3''-diyl)bis(methylene))bis(oxy))bis(2-bromobenzaldehyde) (2b)

Compound **1a** (0.50 g, 1.6 mmol, 1.0 eq.), $SOCl_2$ (1.20 ml, 16.4 mmol, 10.0 eq), 2-bromo-3-hydroxybenzaldehyde (0.99 g, 4.9 mmol, 3.0 eq.), Cs_2CO_3 (2.14 g, 6.6, 4.0 eq.). Crude product was purified by maceration with ethyl acetate/methanol (1:1) mixture giving product **2b** as colorless solid with 61% (0.68 g).

R_f = 0.90 (hexane/ethyl acetate, 1:1); **^{1}H NMR** (600 MHz, $CDCl_3$) δ [ppm]: 10.28 (s, 2H), 7.55 (dd, J = 8.2, 1.5 Hz, 2H), 7.53-7.49 (m, 8H), 7.43 (dd, J = 7.6, 1.5 Hz, 2H), 7.38-7.35 (m, 2H), 7.34 (d, J = 7.6 Hz, 1H), 7.24 (d, J = 7.6 Hz, 2H), 5.36 (s, 4H), 2.04 (s, 3H); **^{13}C NMR** (151 MHz, $CDCl_3$) δ [ppm]: 192.0, 15.9, 142.1, 141.8, 136.3, 134.5, 132.1, 128.9, 128.8, 128.5, 128.3, 126.1, 125.8, 121.6, 119.6, 116.1, 70.5, 18.5; **IR (ATR)** [cm^{-1}]: 3066, 2916, 2854, 1684, 1569, 1451, 1306, 1281, 1241, 1070, 1032, 804, 772, 703

3,3'-(((2,2'',5'-trimethyl-[1,1':3',1''-terphenyl]-3,3''-diyl)bis(methylene))bis(oxy))bis(2-bromobenzaldehyde) (2c)

Compound **1c** (0.60 g, 1.8 mmol, 1.0 eq.), $SOCl_2$ (1.31 ml, 18.0 mmol, 10.0 eq.), 2-bromo-3-hydroxybenzaldehyde (1.09 g, 5.4 mmol, 3.0 eq.), Cs_2CO_3 (2.35 g, 7.2 mmol, 4.0 eq.). Crude product was purified by flash chromatography (SiO_2, hexane to hexane/ethyl acetate, 1:1) giving product **2c** as colorless solid with 79% (1.00 g).

R_f = 0.9 (SiO_2, hexane/ethyl acetate, 1:2); **^{1}H NMR** (600 MHz, $CDCl_3$) δ [ppm]: 10.46 (s, 2H), 7.56 (dd, J = 7.7, 1.4 Hz, 2H), 7.53 9dd, J = 6.7, 2.2 Hz, 2H), 7.40-7.37 (m, 2H), 7.34-7.28 (m, 4H), 7.25 (dd, J = 8.2, 1.4 Hz, 2H), 7.16-7.13 (m, 2H), 7.09 (bs, 1H), 5.22 (s, 4H), 2.47 (s, 3H);

^{13}**C NMR** (151 MHz, $CDCl_3$) δ [ppm]: 192.4, 155.7, 143.0, 141.6, 137.8, 135.1, 134.5, 134.0, 130.4, 128.9, 128.5, 127.8, 125.8, 122.0, 118.8, 118.1, 70.6, 21.7, 16.5; **IR (ATR)** [cm^{-1}]: 2864, 1687, 1588, 1459, 1357, 1305, 1278, 1238, 1023, 783

4,4'-(((2,2'',5'-trimethyl-[1,1':3',1''-terphenyl]-3,3''-diyl)bis(methylene))bis(oxy))bis(2,5-difluorobenzaldehyde) (2d)

Compound **1c** (0.60 g, 1.8 mmol 1.0 eq.), $SOCl_2$ (1.31 ml, 18.0 mmol, 10.0 eq.), 2,5-difluoro-4-hydroxybenzaldehyde (0.82 g, 5.4 mmol, 3.0 eq.), Cs_2CO_3 (2.35 g, 7.2 mmol, 4.0 eq.). Crude product was purified by flash chromatography (SiO_2, hexane to hexane/ethyl acetate, 1:1) giving product **2d** as colorless solid with 52% (0.52 g).

$\mathbf{R_f}$ = 0.80 (SiO_2, hexane/ethyl acetate, 1:2); ^{1}**H NMR** (600 MHz, $CDCl_3$) δ [ppm]: 10.21 (d, J = 2.9 Hz, 2H), 7.59 (dd, J = 10.5, 6.4 Hz, 2H), 7.41 (dd, J = 7.4, 1.3 Hz, 2H), 7.33 (dd, J = 7.7, 1.5, 2H), 7.29 (t, J = 7.5 Hz, 2H), 7.15-7.12 (m, 2H), 7.07 (s, 1H), 6.87 (dd, J = 11.3, 6.3, 2H), 5.23 (s, 4H), 2.46 (s, 3H), 2.32 (s, 6H); ^{13}**C NMR** (151 MHz, $CDCl_3$) δ [ppm]: 185.2, 185.1, 162.9, 161.2, 153.5, 150.2, 148.6, 143.3, 141.5, 137.9, 134.5, 133.3, 131.0, 128.9, 128.1, 127.7, 126.0, 117.0, 114.3, 114.2, 71.0, 21.6, 16.5; **IR (ATR)** [cm^{-1}]: 2915, 2881, 1693, 1627, 1519, 1430, 1353, 1289, 1204, 1167, 1108, 1012, 900, 830, 797

2,2'-(((5'-methyl-[1,1':3',1''-terphenyl]-2,2''-diyl)bis(methylene))bis(oxy))bis(3-methoxybenzaldehyde) (2e)

Compound **1d** (0.53 g, 1.7 mmol, 1.0 eq.), $SOCl_2$ (1.26 ml, 17.4 mmol, 10.0 eq.), o-vaniline (0.79 g, 5.2 mmol, 3.0 eq.), K_2CO_3 (0.94 g, 6.8 mmol, 4.0 eq.). Crude product was purified by column chromatography (SiO_2, hexane to hexane/ethyl acetate, 10:1) giving product **2e** as yellow solid with 69% (0.69 g).

$\mathbf{R_f}$ = 0.75 (SiO_2, hexane/ethyl acetate, 1:1); ^{1}**H NMR** (400 MHz, $CDCl_3$) δ [ppm]: 9.96 (s, 2H), 7.57 – 7.52 (m, 2H), 7.39 – 7.35 (m, 4H), 7.33 – 7.28 (m, 4H), 7.08 (s, 2H), 7.05 – 7.00 (m, 4H), 5.16 (s, 4H), 3.72 (s, 6H), 2.37 (s, 3H); ^{13}**C NMR** (101 MHz, CDCl3) δ [ppm]: 190.3, 153.0, 151.1, 142.2, 140.3, 137.8, 133.7, 130.5, 130.4, 129.1, 128.6, 127.8, 127.4, 124.2, 118.8, 117.8, 73.9, 55.9, 21.5; **IR (ATR)** [cm^{-1}]: 2879, 1650, 1583, 1480, 1370, 1265, 1249, 1061, 948, 753

2,2'-(((3,3''-dimethoxy-5'-methyl-[1,1':3',1''-terphenyl]-4,4''-diyl)bis(methylene))bis(oxy))bis(3-methoxybenzaldehyde) (2f)

Compound **1e** (0.20 g, 0.6 mmol, 1.0 eq.), $SOCl_2$ (0.39 ml, 5.5 mmol, 10.0 eq.), o-vanillin (0.25 g, 1.7 mmol, 3.0 eq.), K_2CO_3 (0.31 g, 2.2 mmol, 4 eq.). Crude product was purified by flash chromatography (SiO_2, hexane to hexane/ethyl acetate, 1:1) giving product **2f** as yellow solid with 66% (0.23 g).

R_f = 0.19 (SiO_2, hexane/ethyl acetate, 4:1); **^{1}H NMR** (600 MHz, $CDCl_3$) δ [ppm]: 10.24 (s, 2H), 7.47 (s, 1H), 7.35 (d, J = 7.7 Hz, 2H), 7.32 (dd, J = 7.7, 1.7 Hz, 2H), 7.31 – 7.30 (m, 2H), 7.12 (dd, J = 7.6, 1.6 Hz, 2H), 7.10 (dd, J = 7.6, 1.6 Hz, 2H), 7.06 (dd, J = 7.8, 0.7 Hz, 2H), 7.03 (d, J = 1.5 Hz, 2H), 5.20 (s, 4H), 3.89 (s, 6H), 3.78 (s, 6H), 2.42 (s, 3H); **^{13}C NMR** (101 MHz, $CDCl_3$) δ [ppm]: 191.2, 158.1, 153.3, 152.0, 143.6, 141.7, 139.0, 131.1, 130.6, 127.4, 124.1, 124.0, 123.6, 119.6, 118.8, 117.9, 109.6, 71.6, 56.2, 55.5, 21.7; **IR (ATR)** [cm^{-1}]: 3266, 2940, 1687, 1575, 1466, 1386, 1265, 1038, 750

4,4'-(((3,3''-dimethoxy-2'-methyl-[1,1':3',1''-terphenyl]-4,4''-diyl)bis(methylene))bis(oxy))bis(3-methoxybenzaldehyde) (2g)

Compound **1f** (0.90 g, 2.5 mmol, 1.0 eq.), $SOCl_2$ (1.79 ml, 24.7 mmol, 10.0 eq.), vanillin (1.13 g, 7.4 mmol, 3.0 eq.), K_2CO_3 (1.37 g, 9.9 mmol, 4.0 eq.). Crude product was purified by flash chromatography (SiO_2, dichloromethane to dichloromethane/methanol, 20:1) giving product **2g** as a yellowish solid with 32% (0.50 g).

R_f = 0.35 (SiO_2, hexane/ethyl acetate, 3:1); **^{1}H NMR** (600 MHz, $CDCl_3$) δ [ppm]: 9.87 (s, 2H), 7.51 (d, J = 7.7 Hz, 2H), 7.48 – 7.45 (m, 4H), 7.34 – 7.30 (m, 1H), 7.29 – 7.27 (m, 2H), 7.12 (d, J = 8.0 Hz, 2H), 6.99 (dd, J = 7.7, 1.4 Hz, 2H), 6.94 – 6.91 (m, 2H), 5.36 (s, 4H), 3.99 (s, 6H), 3.91 (s, 6H), 2.16 (s, 3H); **^{13}C NMR** (151 MHz, $CDCl_3$) δ [ppm]: 191.0, 156.4, 153.7, 150.1, 143.5, 142.7, 133.0, 130.2, 129.0, 128.2, 126.8, 125.4, 122.9, 121.8, 112.3, 111.6, 109.4, 65.9, 56.1, 55.6, 18.7; **IR (ATR)** [cm^{-1}]: 3218, 3076, 2932, 2835, 1689, 1586, 1507, 1462, 1407, 1262, 1226, 1171, 1124, 1032, 997, 813, 787

4,4'-(((2,2',2''-trimethyl-[1,1':3',1''-terphenyl]-4,4''-diyl)bis(methylene))bis(oxy))bis(3-methoxybenzaldehyde) (2h)

Compound **(1g)** (0.58 g, 1.7 mmol 1.0 eq.), $SOCl_2$ (1.25 ml, 17.3 mmol, 10.0 eq.), vanillin (0.79 g, 5.2 mmol, 3.0 eq.), Cs_2CO_3 (2.26 g, 6.9 mmol, 3.0 eq.). Crude product was purified by flash chromatography (SiO_2, hexane to hexane/ethyl acetate, 1:2) giving product **2h** as a yellowish solid with 61% (0.64 g).

R$_f$ = 0.13 (SiO_2, hexane/ethyl acetate, 3:1); **^{1}H NMR** (400 MHz, DMSO-d_6) δ [ppm] (mixture of rotamers) : 9.85 (s, 2H), 7.57 (dd, *J* = 8.2, 1.7 Hz, 2H), 7.42 (d, *J* = 8.5 Hz, 4H), 7.38 – 7.29 (m, 5H), 7.21 – 7.15 (m, 2H), 7.10 (d, *J* = 7.5 Hz, 2H), 5.22 (s, 4H), 3.85 (s, 6H), 2.07 and 2.05 (singlets, 6H, from rotamers), 1.68 and 1.66 (singlets, 3H, from rotamers); **^{13}C NMR** (101 MHz, DMSO-d_6) δ [ppm] (mixture of rotamers): 191.9, 153.9, 149.9, 141.8, 141.7, 136.0, 135.9, 135.8, 135.7, 133.5, 133.3, 130.3, 130.2, 130.1, 129.9, 129.6, 128.7, 126.5, 126.2, 113.0, 110.2, 70.5, 56.1, 20.1, 19.9, 17.7, 17.6; **IR (ATR)** [cm^{-1}]: 3009, 2937, 2833, 2729, 1683, 1586, 1508, 1464, 1424, 1395, 1266, 1135, 1030, 999, 806, 735

4,4'-(((2',3,3''-trimethyl-[1,1':3',1''-terphenyl]-4,4''-diyl)bis(methylene))bis(oxy))bis(3-methoxybenzaldehyde) (2i)

Compound **1h** (0.96 g, 2.9 mmol 1.0 eq.), $SOCl_2$ (2.09 ml, 28.8 mmol, 10.0 eq.), vanillin (1.31 g, 8.6 mmol, 3.0 eq.), Cs_2CO_3 (2.81 g, 8.6 mmol, 3.0 eq.). Crude product was purified by flash chromatography (SiO_2, hexane to hexane/ethyl acetate, 1:3) giving product **2i** as a yellowish solid with 50% (0.87 g).

R$_f$ = 0.15 (SiO_2, hexane/ethyl acetate, 3:1); **^{1}H NMR** (600 MHz, DMSO-d_6) δ [ppm]: 9.87 (s, 2H), 7.59 (dd, J = 8.2, 1.8 Hz, 2H), 7.50 (d, J = 7.8 Hz, 2H), 7.44 (d, J = 1.8 Hz, 2H), 7.38 (d, J = 8.3 Hz, 2H), 7.32 (t, J = 7.5 Hz, 1H), 7.28 (s, 2H), 7.25 – 7.22 (m, 2H), 7.21 (d, J = 7.6 Hz, 2H), 5.25 (s, 4H), 3.85 (s, 6H), 2.39 (s, 6H), 2.10 (s, 3H); **^{13}C NMR** (151 MHz, DMSO-d_6) δ [ppm]: 191.9, 153.8, 149.9, 142.5, 142.3, 137.3, 133.5, 132.6, 131.5, 130.3, 129.2, 127.1, 126.5, 126.1, 113.0, 110.2, 69.1, 56.1, 56.0, 19.2, 18.9; **IR (ATR)** [cm^{-1}]: 3059, 3013, 2922, 2821, 2735, 1686, 1586, 1516, 1454, 1423, 1342, 1270, 1124, 1033, 990, 852, 808, 786, 734

4,4'-(((2',3,3''-trimethyl-[1,1':3',1''-terphenyl]-4,4''-diyl)bis(methylene))bis(oxy))bis(2,5-difluorobenzaldehyde) (2j)

Compound **1h** (0.96 g, 2.9 mmol 1.0 eq.), $SOCl_2$ (2.09 ml, 28.8 mmol, 10.0 eq.), 2,5-difluoro-4-hydroxybenzaldehyde (1.15 g, 7.6 mmol, 2.6 eq.), Cs_2CO_3 (2.81 g, 8.6 mmol, 3.0 eq.). Crude product was purified by flash chromatography (SiO_2, chloroform to chloroform/methanol, 9:1) giving product **2j** as a yellowish solid with 30% (0.52 g).

R$_f$ = 0.87 (SiO_2, hexane/ethyl acetate, 1:1); **^{1}H NMR** (600 MHz, DMSO-d_6) δ [ppm]: 10.09 (s, 2H), 7.65 (dd, J = 10.8, 6.5 Hz, 2H), 7.57 (dd, J = 12.1, 6.6 Hz, 2H), 7.52 (d, J = 7.8 Hz, 2H), 7.35 – 7.28 (m, 3H), 7.26 (dd, J = 7.8, 1.4 Hz, 2H), 7.22 (d, J = 7.6 Hz, 2H), 5.36 (s, 4H), 2.39 (s, 6H), 2.08 (s, 3H); **^{13}C NMR** (151 MHz, DMSO-d_6) δ [ppm]: 186.1, 160.6, 153.3, 142.7,

142.4, 137.5, 132.6, 132.5, 131.7, 129.4, 129.3, 127.2, 126.1, 114.8, 114.7, 104.3, 104.1, 70.4, 19.1, 18.9; **IR (ATR)** [cm^{-1}]: 2923, 2853, 1676, 1627, 1514, 1436, 1353, 1289, 1265, 1164, 1108, 978, 883, 837, 697, 569

3,3'-(((2'-methyl-[1,1':3',1''-terphenyl]-4,4''-diyl)bis(methylene))bis(oxy))bis(2-bromobenzaldehyde) (2k)

Compound **1i** (0.31 g, 1.0 mmol 1.0 eq.), $SOCl_2$ (0.72 ml, 10.0 mmol, 10.0 eq.), 2-bromo-3-hydroxybenzaldehyde (0.60 g, 3.0 mmol, 3.0 eq.), Cs_2CO_3 (1.30 g, 4.0 mmol, 4 eq.). Crude product was purified by flash chromatography (SiO_2, hexane to hexane/ethyl acetate, 1:1) giving product **2k** as a yellow solid with 71% (0.48 g).

$\mathbf{R_f}$ = 0.67 (SiO_2, ethyl acetate/methanol, 1:1); **^{1}H NMR** (400 MHz, $CDCl_3$) δ [ppm]: 10.46 (s, 2H), 7.55 (d, *J* = 7.9 Hz, 6H), 7.41 (d, *J* = 8.0 Hz, 4H), 7.36 (d, *J* = 7.9 Hz, 2H), 7.26 – 7.20 (m, 5H), 5.25 (s, 4H), 2.13 (s, 3H); **^{13}C NMR** (101 MHz, CDCl3) δ [ppm]: 192.4, 155.5, 142.5, 142.4, 135.0, 134.6, 133.0, 129.8, 129.3, 128.4, 126.9, 125.6, 122.0, 118.9, 118.0, 71.3, 18.9; **IR (ATR)** [cm^{-1}]: 3310, 3068, 2919, 2860, 1692, 1564, 1516, 1465, 1379, 1270, 1238, 1139, 1032, 790

4,4'-(((2'-methyl-[1,1':3',1''-terphenyl]-4,4''-diyl)bis(methylene))bis(oxy))bis(2-methoxybenzaldehyde) (2l)

Compound **1i** (0.47 g, 1.55 mmol 1.0 eq.), $SOCl_2$ (1.12 ml, 15.5 mmol, 10.0 eq.), vanillin (0.71 g, 4.6 mmol, 3.0 eq.), Cs_2CO_3 (2.02 g, 6.0 mmol, 4 eq.). Crude product was purified by flash chromatography (SiO_2, hexane to hexane/ethyl acetate, 1:1) giving product **2l** as a yellowish solid with 46% (0.41 g).

$\mathbf{R_f}$ = 0.42 (SiO_2, hexane/ethyl acetate, 1:1); **^{1}H NMR** (600 MHz, $CDCl_3$) δ [ppm]: 9.87 (s, 2H), 7.55-7.37 (m, 12H), 7.34-7.27 (m, 1H), 7.26-7.20 (m, 2H), 7.08 (d, J = 7.9 Hz, 2H), 5.30 (s, 4H), 3.98 (s, 6H), 2.12 (s, 3H); **^{13}C NMR** (151 MHz, $CDCl_3$) δ [ppm]: 191.0, 153.8, 150.2, 142.5, 142.5, 134.7, 133.0, 130.5, 129.8, 129.3, 127.3, 126.7, 125.6, 112.5, 109.5, 70.9, 56.2, 18.9; **IR (ATR)** [cm^{-1}]: 2942, 1693, 1585, 1482, 1376, 1267, 1060, 974, 782

4,4'-(((2'-chloro-[1,1':3',1''-terphenyl]-4,4''-diyl)bis(methylene))bis(oxy))bis(2-methoxybenzaldehyde) (2m)

Compound **1j** (1.30 g, 4.0 mmol, 1.0 eq.), $SOCl_2$ (2.80 ml, 38.4 mmol, 9.6 eq.), vanillin (1.84 g, 12.0 mmol, 3.0 eq.), Cs_2CO_3 (5.20 g, 16.0, 4.0 eq.). Crude product was purified by flash

chromatography (SiO_2, hexane to hexane/ethyl acetate, 1:1) giving product **2m** with 76% (1.81 g).

R_f = 0.20 (SiO_2, hexane/ethyl acetate, 2:1; **^{1}H NMR** (600 MHz, $CDCl_3$) δ [ppm]: 9.80 (s, 2H), 7.46 (d, J = 8.3 Hz, 4H), 7.43 (d, J = 8.3 Hz, 4H), 7.41-7.34 (m, 4H), 7.30 (dd, J = 8.5, 6.4 Hz, 1H), 7.25 (d, J = 1.4 Hz, 1H), 7.20 (s, 1H), 7.00 (d, J = 8.2 Hz, 2H), 5.23 (s, 4H), 3.90 (s, 6H); **^{13}C NMR** (151 MHz, $CDCl_3$) δ [ppm]: 191.0, 153.8, 150.2, 141.3, 140.1, 135.5, 131.0, 130.7, 130.5, 130,1, 127.1, 126.8, 126.7, 112.5, 109.5, 70.8, 56.2; **IR (ATR)** [cm^{-1}]: 2936, 2849, 1690, 1591, 1508, 1465, 1264, 1233, 1129, 1012, 806, 786, 735

3,3'-(((2'-methyl-[1,1':3',1''-terphenyl]-3,3''-diyl)bis(methylene))bis(oxy))bis(2-bromobenzaldehyde) (2n)

Compound **1k** (1.20 g, 3.9 mmol, 1.0 eq.), $SOCl_2$ (2.85 ml, 39.3 mmol, 10.0 eq.), 2-bromo-3-hydroxybenzaldehyde (1.66 g, 8.2 mmol, 2.1 eq.), Cs_2CO_3 (5.12 g, 15.7 mmol, 4.0 eq.). Crude product was purified by flash chromatography (SiO_2, hexane to hexane/ethyl acetate, 1:1) giving final product **2n** as a yellowish solid with 72% yield (1.92 g).

R_f = 0,39 (SiO_2, hexane/ethyl acetate, 2:1); **^{1}H NMR** (600 MHz, $CDCl_3$) δ [ppm]: 10.37 (d, J = 0.6 Hz, 1H), 10.33 (d, J = 0.7 Hz, 1H), 7.58 (dd, J = 7.7, 1.5 Hz, 2H), 7.51 (dd, J = 8.2, 1.5 Hz, 2H), 7.46 (dd, J = 7.7, 1.4 Hz, 2H), 7.43 – 7.38 (m, 2H), 7.38 – 7.34 (m, 2H), 7.29 – 7.25 (m, 2H), 7.23 (dd, J = 8.2, 6.8 Hz, 1H), 7.18 (d, J = 6.3 Hz, 2H), 7.12 (dd, J = 8.1, 1.4 Hz, 2H), 5.19 (s, 4H), 2.02 (s, 3H); **^{13}C NMR** (151 MHz, $CDCl_3$) δ [ppm]: 192.2, 191.7, 155.4, 153.7, 142.8, 142.5, 135.8, 135.1, 135.0, 132.9, 129.3, 129.2, 128.6, 128.3, 128.1, 125.7, 125.5, 124.0, 121.9, 121.6, 119.0, 118.0, 92.1, 71.4, 29.7, 18.7; **IR (ATR)** [cm^{-1}]: 3068, 2862, 1689, 1568, 1459, 1437, 1379, 1303, 1278, 1239, 1041, 1025, 933, 907, 780, 704

2,2'-(((2'-methyl-[1,1':3',1''-terphenyl]-3,3''-diyl)bis(methylene))bis(oxy))bis(3-methoxybenzaldehyde) (2o)

Compound **1k** (1.50 g, 4.9 mmol, 1.0 eq.), $SOCl_2$ (3.57 ml, 49.2 mmol, 10.0 eq.), *o*-vanillin (1.57 g, 10.3 mmol, 2.1 eq.), Cs_2CO_3 (6.42 g, 19.7 mmol, 4.0 eq.). Crude product was purified by flash chromatography (SiO_2, hexane to hexane/ethyl acetate, 1:1) giving product **2o** as a yellowish solid with 87% yield (2.46 g).

R_f = 0.37 (SiO_2, hexane/ethyl acetate, 2:1)**;** **^{1}H NMR** (600 MHz, $CDCl_3$) δ [ppm]: 10.19 (s, 2H), 7.35 (d, J = 7.8 Hz, 2H), 7.32 – 7.30 (m, 4H), 7.26 – 7.25 (m, 3H), 7.25 – 7.24 (m, 1H), 7.19 (d, J = 7.3 Hz, 1H), 7.11 (d, J = 7.7 Hz, 4H), 7.07 (d, J = 7.7 Hz, 2H), 5.16 (s, 4H), 3.87

(s, 6H), 1.91 (s, 3H); **^{13}C NMR** (151 MHz, DMSO-d_6) δ [ppm] 190.2, 153.5, 150.5, 142.5, 142.3, 136.8, 132.6, 130.3, 130.1, 129.6, 129.3, 128.8, 128.1, 126.1, 125.0, 119.2, 118.6, 75.7, 56.6, 18.7; **IR (ATR)** [cm^{-1}]: 3366, 3035, 2944, 2912, 1694, 1584, 1485, 1457, 1418, 1340, 1266, 1246, 1182, 1023, 905, 778, 708

((((5'-methyl-[1,1':3',1''-terphenyl]-3,3''-diyl)bis(methylene))bis(oxy))bis(3,1-phenylene))dimethanol (3a)

Compound **1a** (0.50 g, 1.6 mmol, 1.0 eq), $SOCl_2$ (1.19 ml, 16.0 mmol, 10.0 eq), 3-(hydroxymethyl)phenol (0.61 g, 4.8 mmol, 3.0 eq.), Cs_2CO_3 (2.14 g, 6.4 mmol, 4.0 eq.). Crude product was purified by flash chromatography (SiO_2, hexane to hexane/ethyl acetate, 3:1) giving product **3a** as colorless solid with 79% (0.67 g).

R_f = 0.70 (hexane/ethyl acetate, 1:3); **^{1}H NMR** (600 MHz, $CDCl_3$) δ [ppm]: 7.51 (d, J = 8.2 Hz, 4H), 7.43-7.38 (m, 4H), 7.32-7.29 (m, 3H), 7.26 (s, 1H), 7.24 (s, 1H), 7.09-7.05 (m, 2H), 7.00-6.96 (m, 2H), 6.99 (dd, J = 8.1, 2.7 Hz, 2H), 5.13 (s, 4H), 4.70 (s, 4H), 2.14 (s, 3H); **^{13}C NMR** (151 MHz, $CDCl_3$) δ [ppm]: 159.3, 142.8, 142.6, 142.3, 135.6, 135.1, 129.8, 129.7, 129.2, 127.5, 125.6, 119.6, 114.3, 113.4, 70.0, 65.4, 18.9; **IR (ATR)** [cm^{-1}]: 3304, 2928, 2873, 1594, 1489, 1445, 1381, 1260, 1168, 1260, 1168, 1008, 863, 795

((((5'-methyl-[1,1':3',1''-terphenyl]-3,3''-diyl)bis(methylene))bis(oxy))bis(3,1-phenylene))dimethanol (3a)

Compound **1a** (0.50 g, 1.6 mmol, 1.0 eq), $SOCl_2$ (1.19 ml, 16.0 mmol, 10.0 eq), 3-(hydroxymethyl)phenol (0.61 g, 4.8 mmol, 3.0 eq.), Cs_2CO_3 (2.14 g, 6.4 mmol, 4.0 eq.). Crude product was purified by flash chromatography (SiO_2, hexane to hexane/ethyl acetate, 3:1) giving product **3a** as colorless solid with 79% (0.67 g).

R_f = 0.70 (hexane/ethyl acetate, 1:3); **^{1}H NMR** (600 MHz, $CDCl_3$) δ [ppm]: 7.51 (d, J = 8.2 Hz, 4H), 7.43-7.38 (m, 4H), 7.32-7.29 (m, 3H), 7.26 (s, 1H), 7.24 (s, 1H), 7.09-7.05 (m, 2H), 7.00-6.96 (m, 2H), 6.99 (dd, J = 8.1, 2.7 Hz, 2H), 5.13 (s, 4H), 4.70 (s, 4H), 2.14 (s, 3H); **^{13}C NMR** (151 MHz, $CDCl_3$) δ [ppm]: 159.3, 142.8, 142.6, 142.3, 135.6, 135.1, 129.8, 129.7, 129.2, 127.5, 125.6, 119.6, 114.3, 113.4, 70.0, 65.4, 18.9; **IR (ATR)** [cm^{-1}]: 3304, 2928, 2873, 1594, 1489, 1445, 1381, 1260, 1168, 1260, 1168, 1008, 863, 795

((((2,2''-difluoro-5'-methyl-[1,1':3',1''-terphenyl]-3,3''-diyl)bis(methylene))bis(oxy))bis(3,1-phenylene))dimethanol (3c)

Compound **1b** (0.71 g, 2.1 mmol, 1.0 eq.), $SOCl_2$ (1.52 ml, 21.0 mmol, 10.0 eq), 3-(hydroxymethyl)phenol (1.74 g, 6.3 mmol, 3.0 eq.), Cs_2CO_3 (1.92 g, 8.4 mmol, 4.0 eq.). Crude product was purified by flash chromatography (SiO_2, hexane to hexane/ethyl acetate, 3:1) giving product **3c** was obtained as yellowish oil with 69% (0.56 g).

R_f = 0.30 (hexane/ethyl acetate, 1:2); **^{1}H NMR** (600 MHz, $CDCl_3$) δ [ppm]: 7.56 (s, 1H), 7.50 (t, J = 6.8 Hz, 2H),7.46 (t, J = 7.4, 2H), 7.40 (s, 2H), 7.29 (t, J = 7.9 Hz, 2H), 7.24 (t, J = 7.6 Hz, 2H), 7.05 (s, 2H), 6.97 (d, J = 7.5 Hz, 2H), 6.94 (dd, J = 8.2, 2.3 Hz, 2H), 5.20 9s, 4H), 4.66 (s, 4H), 2.49 (s, 3H); **^{13}C NMR** (101 MHz, $CDCl_3$) δ [ppm]: 158.9, 142.7, 138.4, 135.8, 130.8, 129.8, 129.4, 129.1, 129.0, 128.8, 125.0, 124.8, 124.4, 119.7, 114.1, 113.4, 65.3, 64.0, 21.7; **IR (ATR)** [cm^{-1}]: 3286, 2918, 2850, 1604, 1585, 1485, 1449, 1381, 1258, 1013, 870, 787

2.3. General procedure for aldehyde reduction

Dialdehyde **(2a-2o)** (1.0 eq.) was dissolved in methanol/DCM 1:1 mixture (40 ml for 1 mmol) Then the $NaBH_4$ was added in one portion (2.0-3.0 eq.) and stirred in room temperature overnight. Reaction was controlled by TLC and then quenched with 1M HCl until pH was lower the 6. Reaction mixture was then extracted with dichloromethane. Organic phases was collected, dried over anhydrous $MgSO_4$ and evaporated. The final product was purified by maceration with cold methanol giving dialcohols **3b-3l** with sufficient purity and 50-100% yield.

((((5'-methyl-[1,1':3',1''-terphenyl]-3,3''-diyl)bis(methylene))bis(oxy))bis(2-methoxy-4,1-phenylene))dimethanol (3b)

Compound **2a** (0.69 g, 1.2 mmol, 1.0 eq.), $NaBH_4$ (0.14 g, 3.2 mmol, 3.0 eq.). Product **3b** was obtained with sufficient purity as colorless solid with 95% (0.67 g).

R_f = 0.61 (hexane/ethyl acetate, 1:2); **^{1}H NMR** (600 MHz, $CDCl_3$) δ [ppm]: 7.47-7.39 (m, 6H), 7.32-7.24 (m, 3H), 7.22 (d, J = 6.9 Hz, 2H), 6.94 (d, J = 2.0 Hz, 2H), 6.89 (d, J = 8.2 Hz, 2H), 6.82 (dd, J = 8.1, 2.0 Hz, 2H), 5.20 (s, 4H), 4.60 (s, 4H), 3.89 (s, 3H); **^{13}C NMR** (151 MHz, $CDCl_3$) δ [ppm]: 150.0, 147.8, 142.74, 142.71, 137.1, 134.4, 133.1, 129.2, 129.0, 128.5, 128.5, 126.0, 125.5, 119.5, 114.4, 111.1, 71.4, 65.4, 56.1, 29.8, 18.8; **IR (ATR)** [cm^{-1}]: 3314, 2916, 2852, 1591, 1516, 1450, 1418, 1381, 1262, 1235, 1159, 1133, 1026, 1007, 921, 856, 791, 704

((((2,2'',5'-trimethyl-[1,1':3',1''-terphenyl]-3,3''-diyl)bis(methylene))bis(oxy))bis(2-bromo-3,1-phenylene))dimethanol (3d)

Compound **2c** (0.40 g, 0.6 mmol 1.0 eq.), $NaBH_4$ (0.065 g, 1.8 mmol, 3.0 eq.). Product 3d was purified by the maceration with methanol and obtained as colorless solid with 92% (0.37 g).

$\mathbf{R_f}$ = 0.63 (hexane/ethyl acetate, 1:2); **^{1}H NMR** (600 MHz, $CDCl_3$) δ [ppm]: 7.54 (dd, J = 7.4, 1.0 Hz, 2H), 7.37 (t, J = 7.9 Hz, 2H), 7.30 (t, J = 7.5 Hz, 2H), 7.26 (dd, J = 7.6, 1.4 Hz, 2H), 7.20-7.24 (m, 6H), 7.00 (s, 1H), 5.40 (t, J = 5.7 Hz, 2H), 5.24 (s, 4H), 4.52 (d, J = 5.6 Hz, 4H), 2.43 (s, 3H), 2.29 (s, 6H); **^{13}C NMR** (151 MHz, $CDCl_3$) δ [ppm]: 154.1, 142.8, 141.9, 141.1, 137., 135.4, 133.7, 129.6, 128.5, 128.0, 127.4, 127.2, 125.5, 119.9, 112.1, 110.3, 69.2, 62.9, 21.1, 15.9; **IR (ATR)** [cm^{-1}]: 3330, 2916, 2875, 1597, 1573, 1465, 1379, 1269, 1087, 1023, 855, 772, 761, 716

((((2,2'',5'-trimethyl-[1,1':3',1''-terphenyl]-3,3''-diyl)bis(methylene))bis(oxy))bis(2,5-difluoro-4,1-phenylene))dimethanol (3e)

Compound **2d** (0.30 g, 0.4 mmol 1.0 eq.) was dissolved in 3 ml anhydrous THF and cooled in ice-bath, then $LiAlH_4$ (0.075 g, 1.9 mmol, 4.0 eq.) in 3 ml anhydrous THF was added portion wise. After 2 hours reaction was quenched with ethyl acetate, water and then 1M HCl until pH approximately 6.0. Organic phase was collected washed with water and brine, then dried over anhydrous $MgSO_4$ and evaporated. Product **3e** was obtained with sufficient purity as gray solid with 99% (0.23 g).

$\mathbf{R_f}$ = 0.58 (hexane/ethyl acetate, 1:2); **^{1}H NMR** (600 MHz, $CDCl_3$) δ [ppm]: 7.41 (dd, J = 7.3, 1.4 Hz, 2H), 7.32-7.23 (m, 5H), 7.17 (dd, J = 11.1, 6.9 Hz, 2H), 7.14-7.11 (m, 2H), 7.06 (s, 1H), 6.81 (dd, J = 10.8, 6.8 Hz, 2H), 5.14 (s, 4H), 4.67 (s, 4H), 2.46 (s, 3H), 2.31 (s, 6H); **^{13}C NMR** (151 MHz, $CDCl_3$) δ [ppm]: 157.0, 155.4, 150.0, 148.4, 147.0, 143.1, 141.6, 137.8, 134.4, 134.4, 130.6, 128.9, 128.0, 127.8, 125.8, 120.2, 120.1, 116.4, 116.4, 116.3 116.22, 103.7, 108.5, 71.0, 58.0, 21.6, 16.4; **IR (ATR)** [cm^{-1}]: 3313, 2923, 1638, 1520, 1423, 1382, 1335, 1286, 1163, 1105, 993, 877, 785

((((3,3''-dimethoxy-2'-methyl-[1,1':3',1''-terphenyl]-4,4''-diyl)bis(methylene))bis(oxy))bis(3-methoxy-4,1-phenylene))dimethanol (3f)

Compound **2g** (0.26 g, 0.40 mmol, 1.0 eq.), $NaBH_4$ (0.04 g, 1.0 mmol, .,5 eq.). Product **3f** was obtained with sufficient purity as grey solid with 78% (0.26 g).

R_f = 0.12 (SiO_2, hexane/ethyl acetate, 1:1), **^{1}H NMR** (600 MHz, DMSO-d_6) δ 7.46 [ppm]: (d, J = 7.6 Hz, 2H), 7.36 – 7.32 (m, 1H), 7.27 (d, J = 7.5 Hz, 2H), 7.05 (s, 2H), 7.02 – 6.94 (m, 6H), 6.87 – 6.80 (m, 2H), 5.06 (s, 4H), 4.40 (s, 4H), 3.86 (s, 6H), 3.77 (s, 6H), 2.11 (s, 3H); **^{13}C NMR** (101 MHz, DMSO-d_6) δ [ppm]: 157.1, 149.4, 147.3, 143.5, 142.8, 135.9, 132.9, 129.5, 129.3, 126.1, 124.1, 121.6, 119.1, 113.5, 112.5, 111.2, 65.6, 63.3, 56.1, 56.0, 19.3; **IR (ATR)** [cm^{-1}]: 3201, 2932, 2837, 2361, 2343, 1611, 1573, 1512, 1463, 1261, 1226, 1136, 1032, 805

((((2,2',2''-trimethyl-[1,1':3',1''-terphenyl]-4,4''-diyl)bis(methylene))bis(oxy))bis(3-methoxy-4,1-phenylene))dimethanol (3g)

Compound **2h** (0.60 g, 0.9 mmol, 1.0 eq.), $NaBH_4$ (0.09 g, 2.5 mmol, 2.5 eq.). Product **3g** was obtained with sufficient purity as grey solid with 77% (0.46 g).

R_f = 0.10 (SiO_2, hexane/ethyl acetate, 1:1); **^{1}H NMR** (400 MHz, DMSO-d_6) δ [ppm] (mixture of rotamers): 7.38 (s, 2H), 7.35 – 7.28 (m, 3H), 7.18 – 7.13 (m, 2H), 7.11 – 7.07 (m, 2H), 7.01 (d, J = 8.2 Hz, 2H), 6.96 (s, 2H), 6.83 (d, J = 8.2 Hz, 2H), 5.10 (t, J = 5.7 Hz, 2H), 5.05 (s, 4H), 4.43 (d, J = 5.7 Hz, 4H), 3.77 (s, 6H), 2.06 and 2.04 (singlets, 6H, from rotamers), 1.68 and 1.66 (singlets, 3H, from rotamers); **^{13}C NMR** (101 MHz, DMSO-d_6) δ [ppm] (mixture of rotamers): 149.5, 147.2, 141.9, 141.8, 141.5, 141.4, 136.8, 136.7, 136.1, 135.8, 135.8, 133.6, 133.4, 129.9, 129.8, 129.4, 128.7, 125.9, 125.8, 119.0, 113.8, 111.2, 70.4, 63.3, 55.9, 20.1, 19.9, 17.7, 17.6; **IR (ATR)** [cm^{-1}]: 3310, 3062, 3012, 2934, 2859, 2726, 1682, 1587, 1511, 1464, 1423, 1339, 1263, 1136, 1030, 1005, 808, 737

((((2',3,3''-trimethyl-[1,1':3',1''-terphenyl]-4,4''-diyl)bis(methylene))bis(oxy))bis(3-methoxy-4,1-phenylene))dimethanol (3h)

Compound **2i** (0.70 g, 1.2 mmol, 1.0 eq.), $NaBH_4$ (0.11 g, 2.9 mmol, 2.5 eq.). Product **3h** was obtained with sufficient purity as colorless solid with 96% (0.71 g.).

R_f = 0.12 (SiO_2, hexane/ethyl acetate, 1:1); **^{1}H NMR** (600 MHz, DMSO-d_6) δ [ppm]: 7.47 (d, J = 7.8 Hz, 2H), 7.34 – 7.29 (m, 1H), 7.25 (s, 2H), 7.24 – 7.18 (m, 4H), 7.06 (d, J = 8.2 Hz, 2H), 6.97 (s, 2H), 6.88 – 6.81 (m, 2H), 5.08 (s, 4H), 4.44 (s, 4H), 3.77 (s, 3H), 2.38 (s, 6H), 2.09 (s, 3H); **^{13}C NMR** (151 MHz, DMSO-d_6) δ [ppm]: 149.5, 147.2, 142.6, 142.0, 137.1, 136.1, 134.4, 132.6, 131.4, 129.2, 129.0, 127.0, 126.0, 119.0, 113.9, 111.2, 69.0, 63.3, 55.9, 19.2, 18.9; **IR (ATR)** [cm^{-1}]: 3207, 3009, 2932, 2870, 2360, 1605, 1520, 1463, 1421, 1333, 1264, 1244, 1163, 1135, 1039, 995, 846, 818, 791

((((2',3,3''-trimethyl-[1,1':3',1''-terphenyl]-4,4''-diyl)bis(methylene))bis(oxy))bis(2,5-difluoro-4,1-phenylene))dimethanol (3i)

Compound **2j** (0.50 g, 0.8 mmol, 1.0 eq.), $NaBH_4$ (0.07 g, 2.1 mmol, 2.5 eq.). Product **3i** was obtained with sufficient purity as colorless solid with 99% (0,50 g).

R_f = 0.44 (SiO_2, hexane/ethyl acetate, 1:1); **^{1}H NMR** (400 MHz, DMSO-d_6) δ [ppm]: 7.48 (d, J = 7.8 Hz, 2H), 7.30 (t, J = 7.5 Hz, 1H), 7.27 – 7.19 (m, 10H), 5.20 (s, 4H), 4.46 (s, 4H), 4.06 (s, 2H), 2.39 (s, 6H), 2.07 (s, 3H); **^{13}C NMR** (101 MHz, DMSO-d_6) δ [ppm]: 162.9, 156.9, 154.5, 149.7, 147.5, 146.3, 142.6, 137.4, 133.3, 132.7, 131.6, 129.2, 127.1, 126.1, 121.7, 116.0, 103.8, 69.8, 56.5, 36.3, 31.3, 19.2, 18.5; **IR (ATR)** [cm^{-1}]: 3211, 2925, 2262, 1664, 1639, 1520, 1462, 1335, 1197, 1165, 1103, 987, 884, 832, 791

((((2'-chloro-[1,1':3',1''-terphenyl]-4,4''-diyl)bis(methylene))bis(oxy))bis(3-methoxy-4,1-phenylene))dimethanol (3j)

Compound **2m** (1.03 g, 1.7 mmol, 1.0 eq.), $NaBH_4$ (0.11 g, 3.0 mmol, 1.8 mmol). The crude was purified by flash chromatography (SiO_2, hexane/ethyl acetate, 1:1 to ethyl acetate/methanol, 3:1) giving product **3j** as colorless solid with 50% (0.52 g).

R_f = 0.18 (SiO_2, hexane/ethyl acetate, 1:2); **^{1}H NMR** (600 MHz, $CDCl_3$) δ [ppm]: 7.61-7.43 (m, 9H), 7.43-7.37 (m, 2H), 7.04-6.94 (m, 4H), 6.81 (d, J = 8.7 Hz, 2H), 5.11 (s, 4H), 5.05 (bs, 2H), 4.41 (s, 4H), 3.77 (s, 6H); **^{13}C NMR** (151 MHz, $CDCl_3$) δ [ppm]: 169.6, 149.5, 147.0, 141.3, 139.3, 137.3, 134.4, 131.1, 130.3, 129.9, 128.0, 127.7, 120.3, 113.8, 112.5, 70.2, 55.9, 52.9, 48.5, 39.2, 23.1; **IR (ATR)** [cm^{-1}]: 3289, 2936, 2863, 1635, 1559, 1515, 1466, 1379, 1261, 1235, 1139, 1035, 1003, 790

((((2'-methyl-[1,1':3',1''-terphenyl]-3,3''-diyl)bis(methylene))bis(oxy))bis(2-bromo-3,1-phenylene))dimethanol (3k)

Compound **2n** (1.80 g, 2.7 mmol, 1.0 eq), $NaBH_4$ (0.203 g, 5.4 mmol, 2.0 eq). After extraction the product precipitated from the DCM solution and ethyl acetate was added to keep it in the organic phase. The crude was purified by flash chromatography (SiO_2, hexane to hexane/ethyl acetate, 2:1) giving product **3k** as yellowish solid with 40% (0.73 g).

R_f = 0,25 (SiO_2, hexane/ethyl acetate, 2:1); **^{1}H NMR** (300 MHz, $CDCl_3$) δ [ppm]: 7.52 – 7.43 (m, 5H), 7.37 – 7.28 (m, 4H), 7.27 (s, 2H), 7.26 – 7.23 (m, J = 2.3 Hz, 2H), 7.07 (dd, 2H), 6.95

(td, J = 8.5, 1.3 Hz, 2H), 5.23 (d, J = 3.3 Hz, 4H), 4.77 (t, J = 3.1 Hz, 4H), 2.11 (s, 3H); **IR (ATR)** [cm^{-1}]: 3275, 2917, 1597, 1571, 1466, 1435, 1404, 1357, 1310, 1246, 1030, 896, 767

((((2'-methyl-[1,1':3',1''-terphenyl]-3,3''-diyl)bis(methylene))bis(oxy))bis(3-methoxy-2,1-phenylene))dimethanol (3l)

Compound **2o** (2.00 g, 3.5 mmol, 1.0 eq.), $NaBH_4$ (0.26 g, 2.9 mmol, 2.5 eq.). Product **3l** was obtained with sufficient purity as a colorless crystalline solid with quantitative yield (2.01 g) $\mathbf{R_f}$ = 0,39 (SiO_2, hexane/ethyl acetate, 3:1); **^{1}H NMR** (400 MHz, $CDCl_3$) δ [ppm]: 7.35 (d, J = 4.6 Hz, 4H), 7.34 – 7.33 (m, 2H), 7.25 (qt, J = 3.5, 1.8 Hz, 2H), 7.21 – 7.16 (m, 1H), 7.13 (d, J = 7.4 Hz, 2H), 6.96 (t, 2H), 6.85 – 6.80 (m, J = 11.5, 6.4, 4.9 Hz, 4H), 5.04 (s, 4H), 4.48 (s, 4H), 3.81 (s, 6H), 1.95 (s, 3H); **^{13}C NMR** (101 MHz, $CDCl_3$) δ [ppm]: 153.2, 145.5, 142.8, 142.6, 137.4, 135.1, 133.1, 129.7, 129.4, 129.0, 128.6, 127.1, 125.5, 124.5, 121.0, 112.2, 75.1, 61.6, 56.1, 19.1; **IR (ATR)** [cm^{-1}]: 3356, 3035, 2913, 2837, 1585, 1481, 1436, 1375, 1269, 1204, 1183, 1082, 1013, 974, 907, 778, 733, 706

3. Copies of the HTRF plot for compounds 7b, 7j, 7m

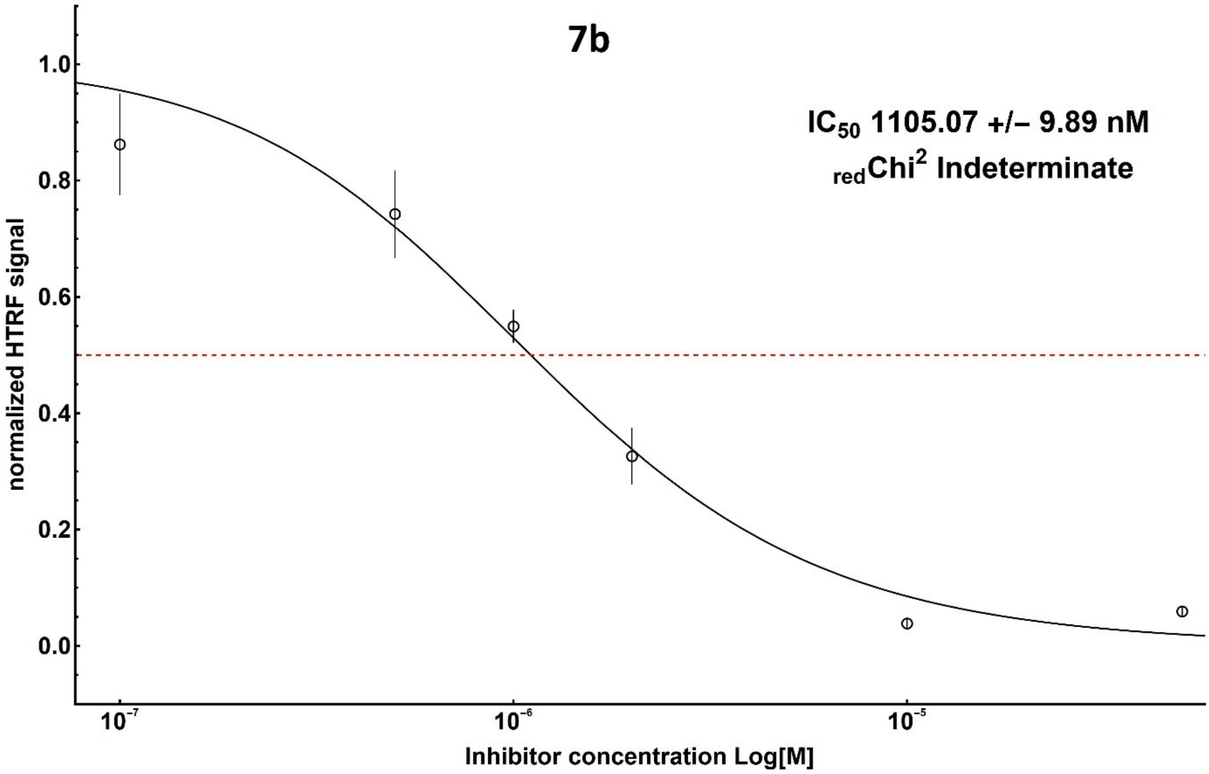

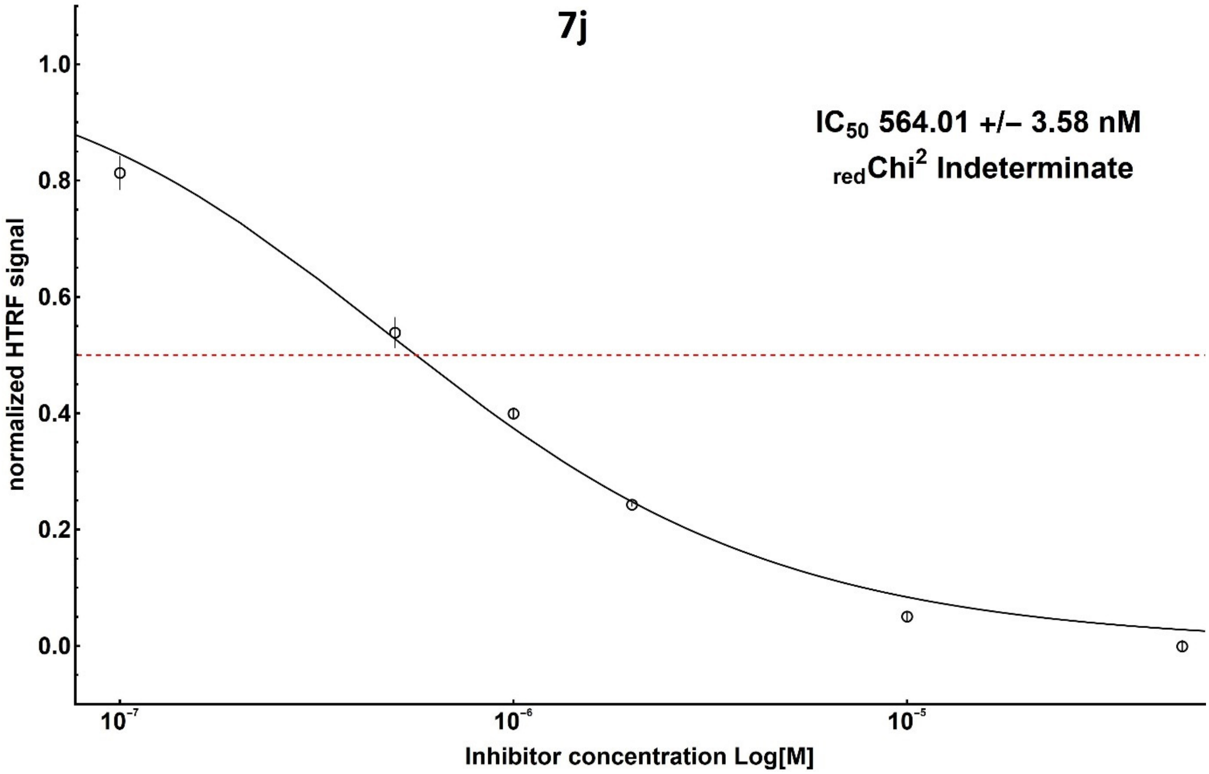

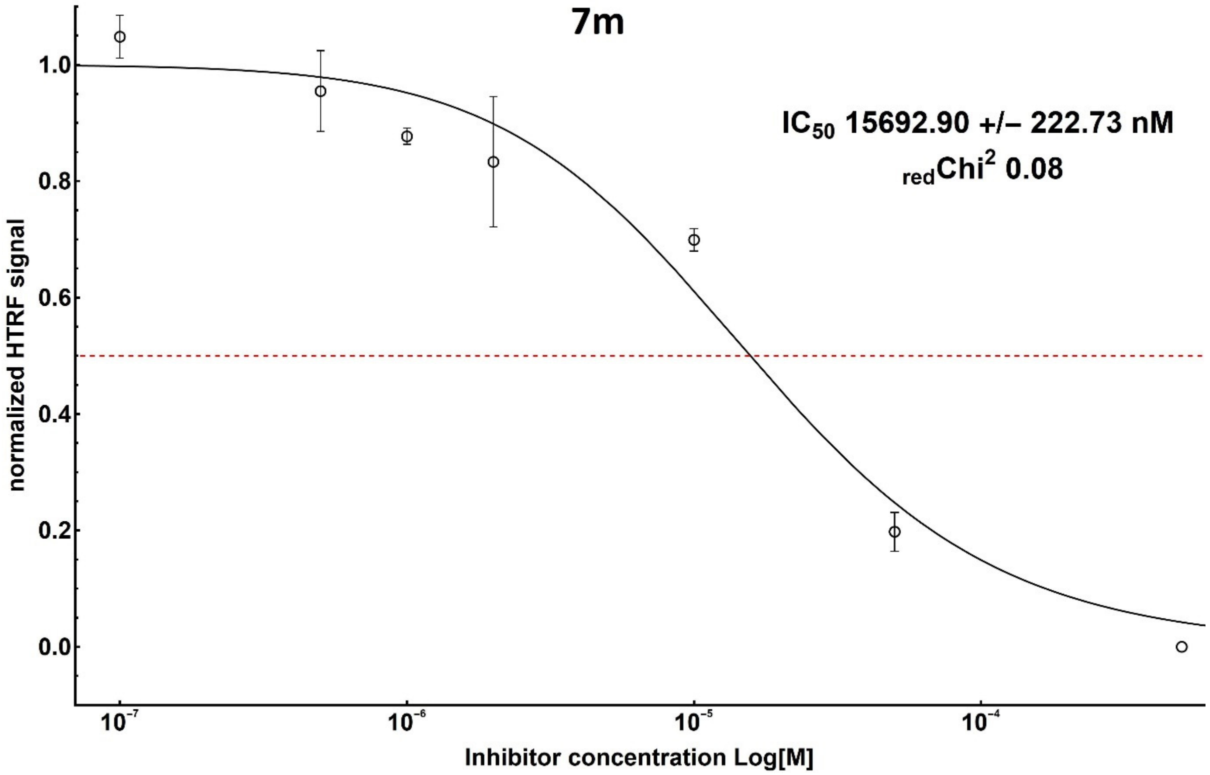

7m
IC50 15692.90 +/– 222.73 nM
redChi2 0.08
1.0
0.8
0.6
0.4
0.2
0.0
normalized HTRF signal
10−7
10−6
10−5
10−4
Inhibitor concentration Log[M]

4. Copies of ^{1}H and ^{13}C NMR spectra and SFC/MS results for final compounds

4a

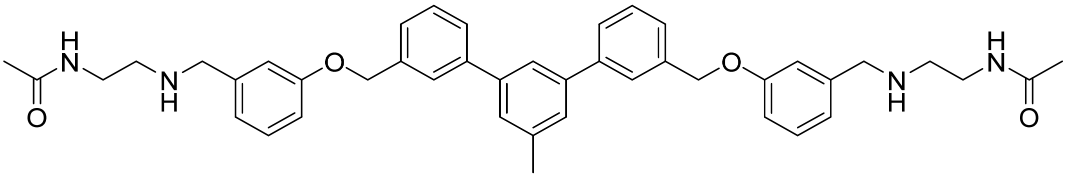

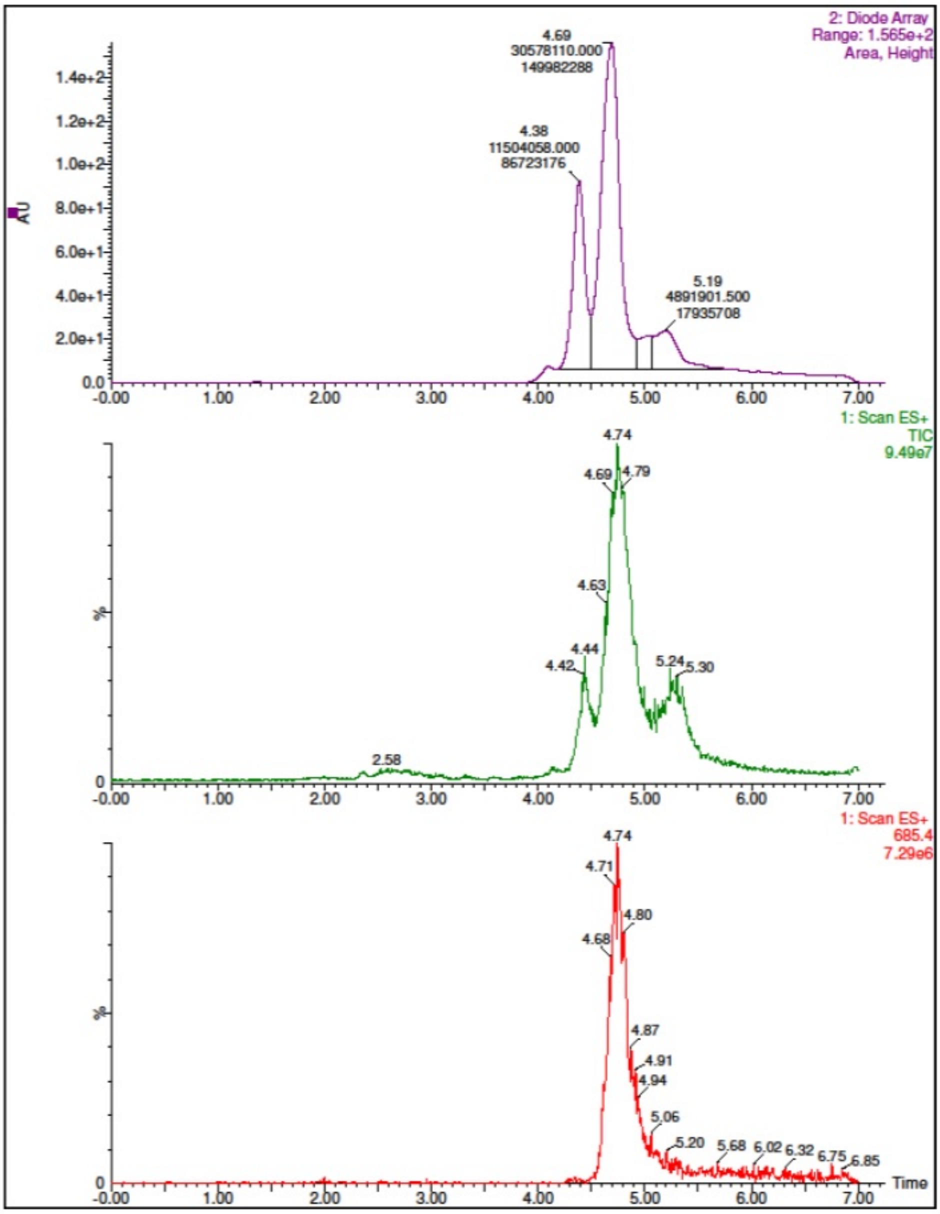

^{1}H NMR (600 MHz, $CDCl_3$)

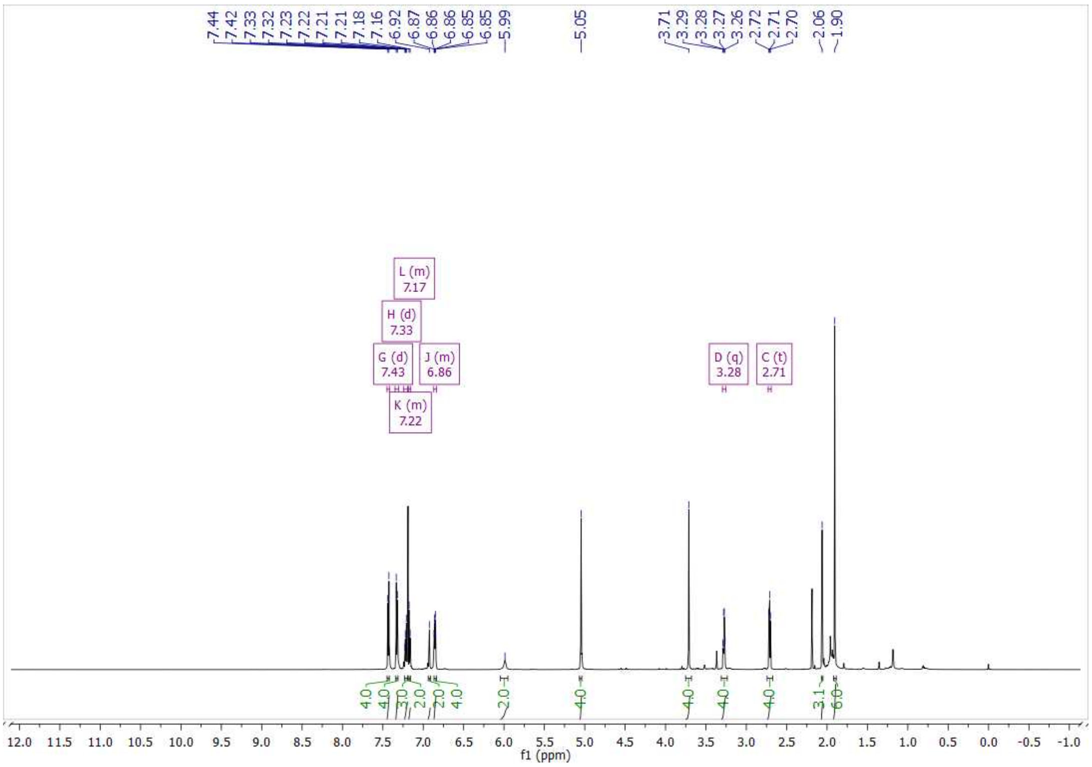

^{13}C NMR (151 MHz, $CDCl_3$)

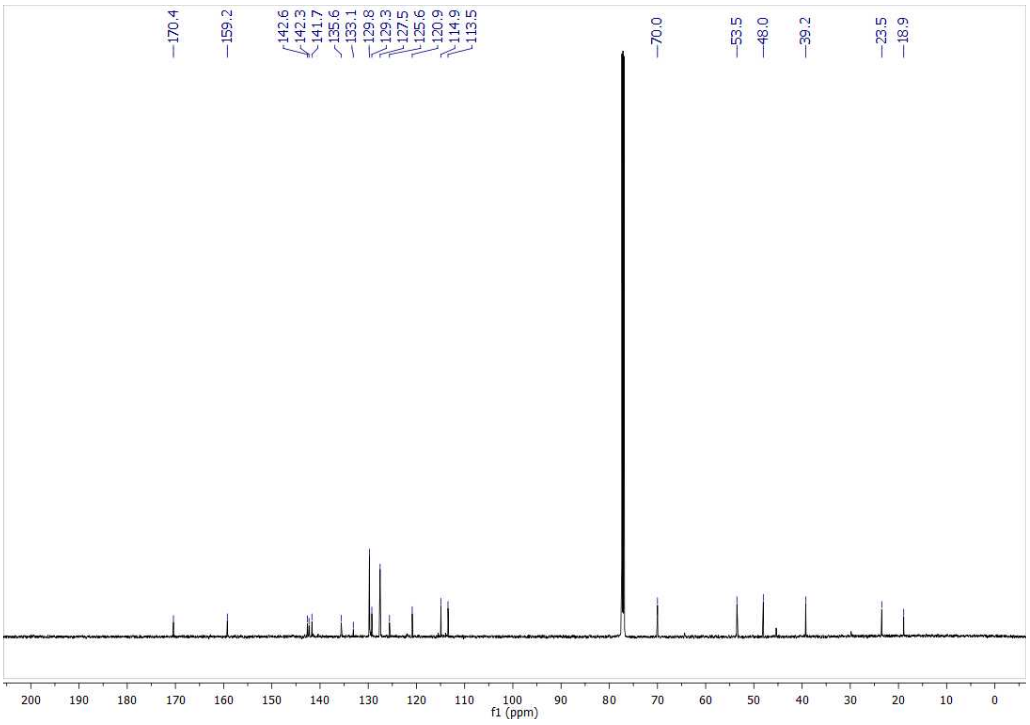

4b

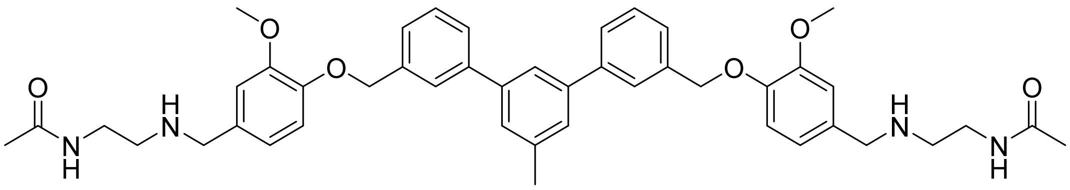

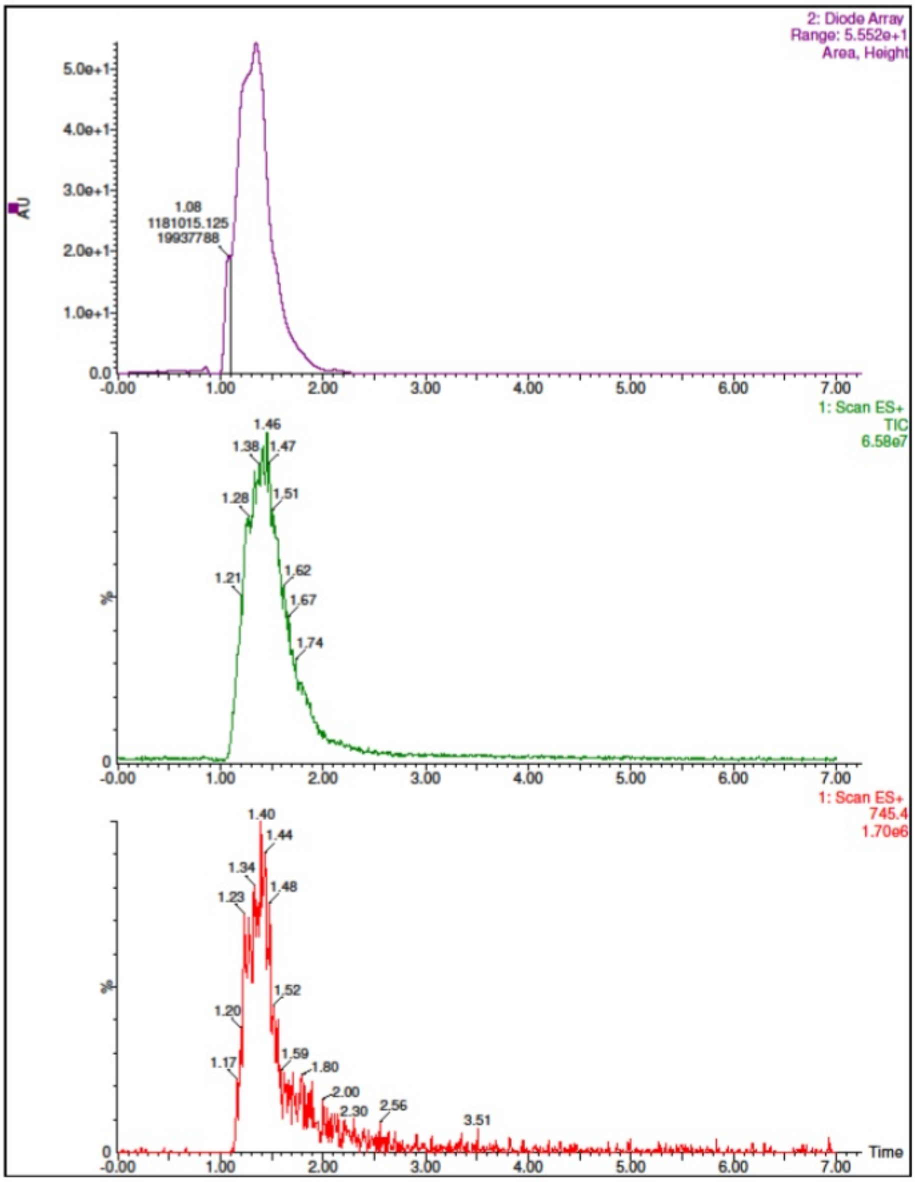
2: Diode Array
Range: 5.552e+1
Area, Height
AU
1.08
1181015.125
19937788
1: Scan ES+
TIC
6.58e7
1.46
1.38
1.47
1.28
1.51
1.21
1.62
1.67
1.74
1: Scan ES+
745.4
1.70e6
1.40
1.44
1.34
1.23
1.48
1.52
1.20
1.17
1.59
1.80
2.00
2.30
2.56
3.51
%
Time

*^{1}**H NMR** (600 MHz, $CDCl_3$)*

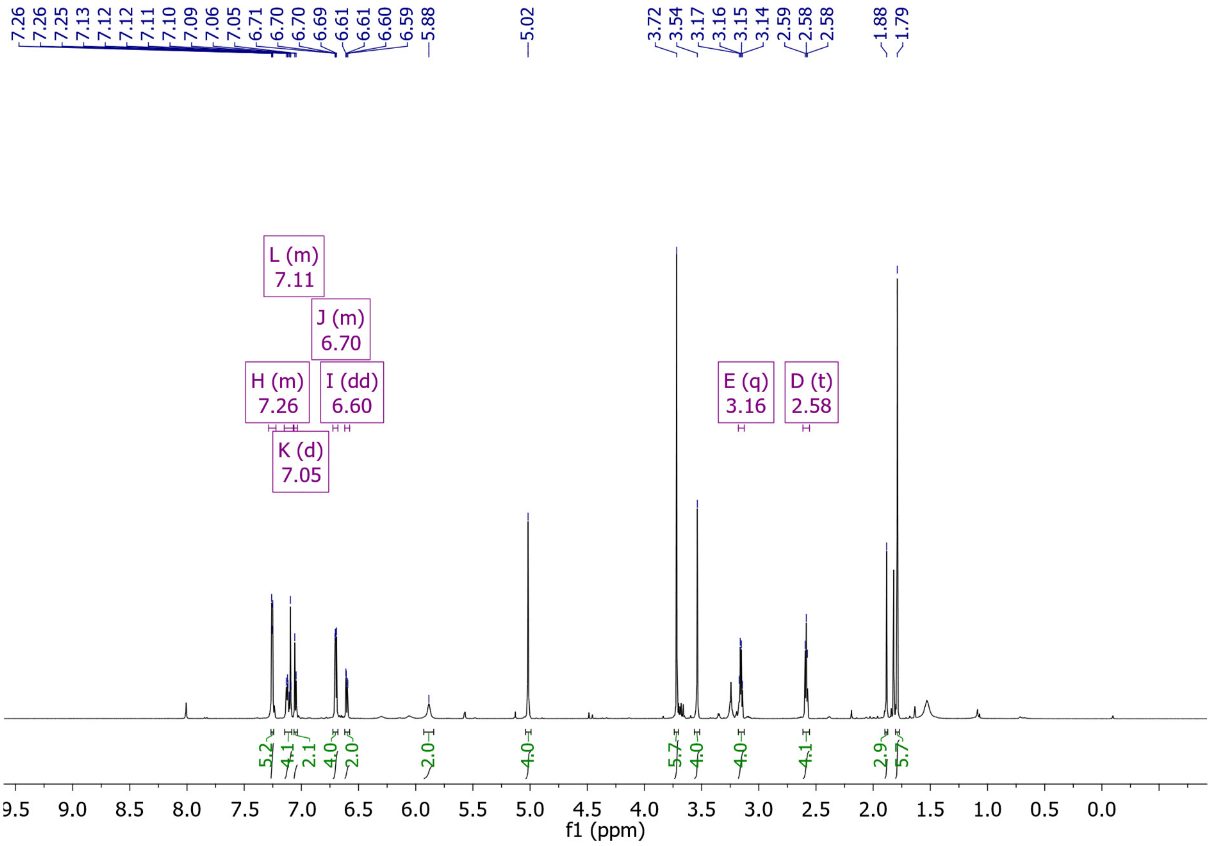

*^{13}**C NMR** (151 MHz, $CDCl_3$)*

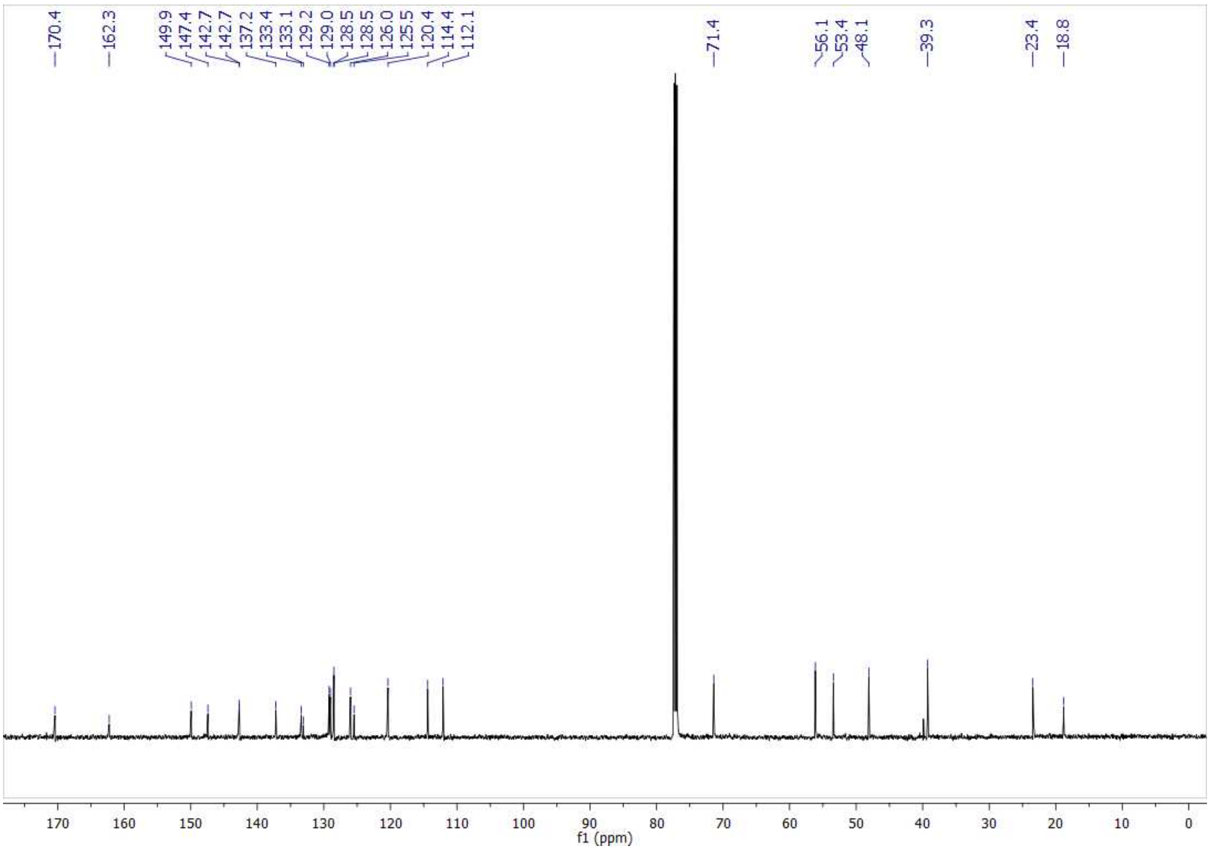

4c

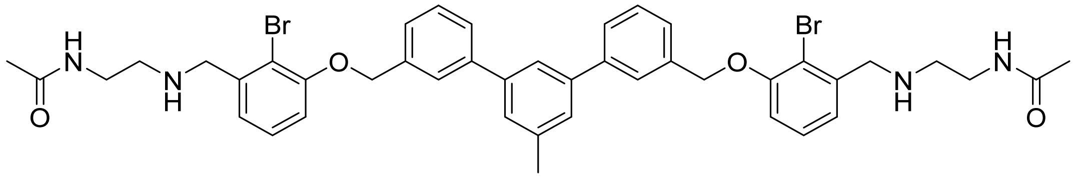

Br
Br
O
O
N
H
N
H
N
H
N
H
O
O

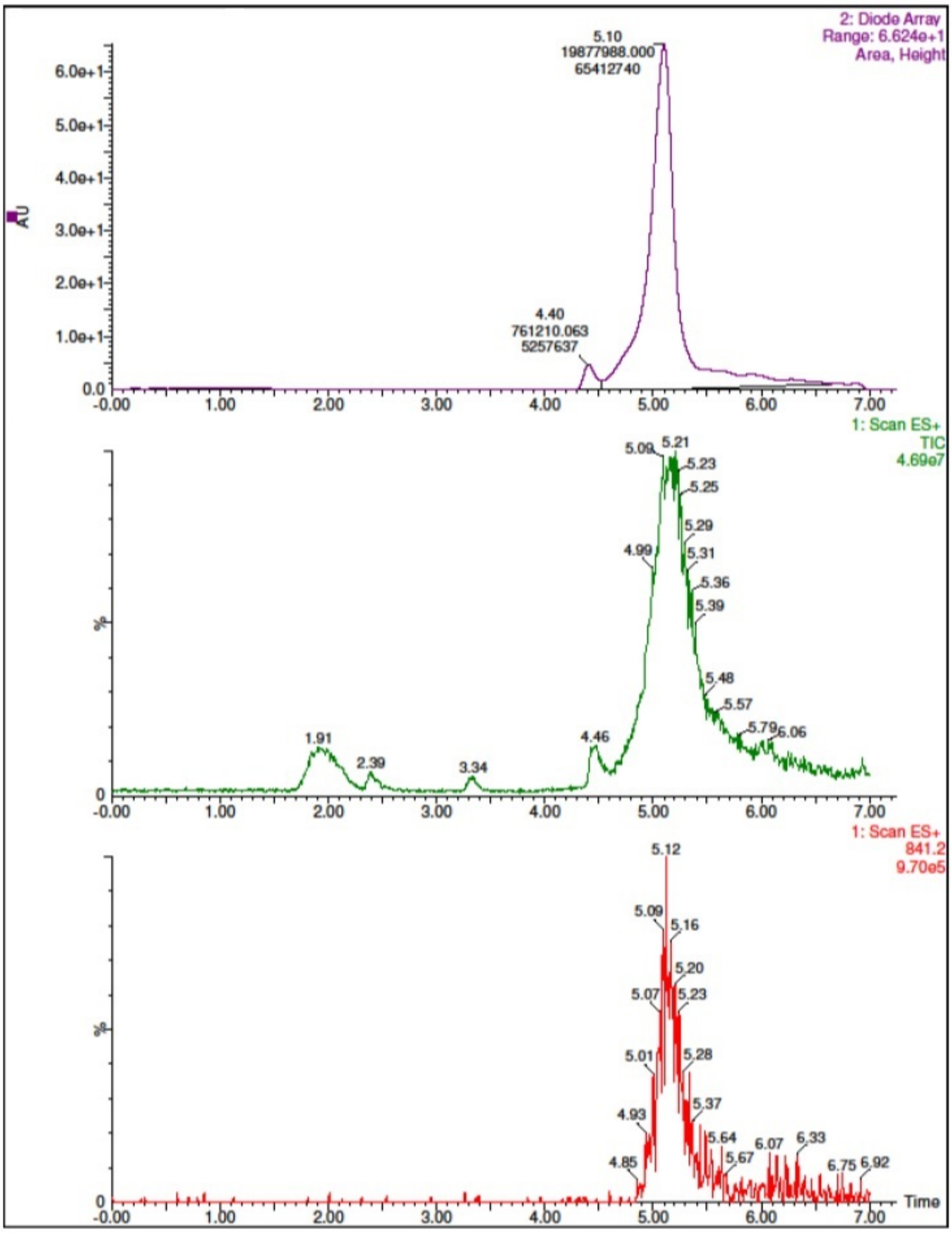

2: Diode Array
Range: 6.624e+1
Area, Height
5.10
19877988.000
65412740
4.40
761210.063
5257637
AU
1: Scan ES+
TIC
4.69e7
1: Scan ES+
841.2
9.70e5
%
Time

^{1}H NMR *(600 MHz, MeOD-d_4)*

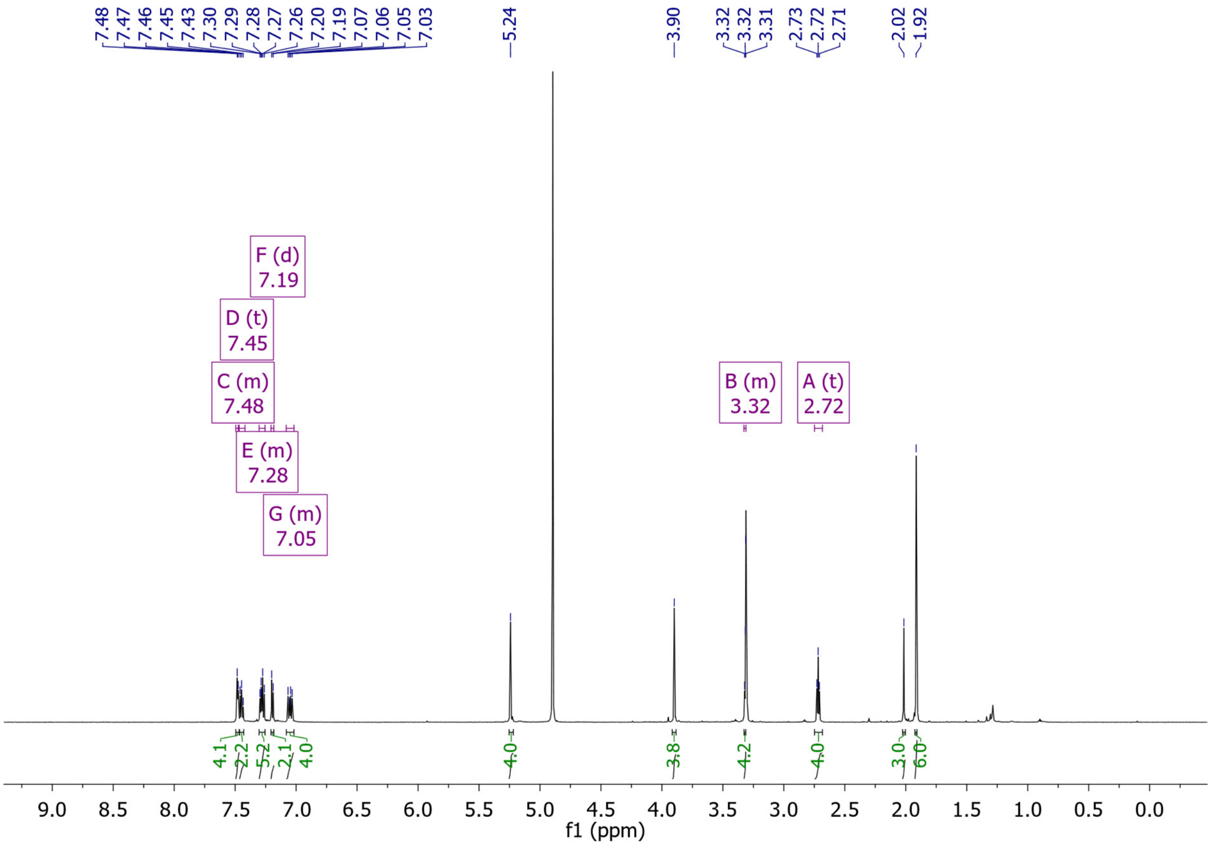

^{13}C NMR *(151 MHz, $CDCl_3$)*

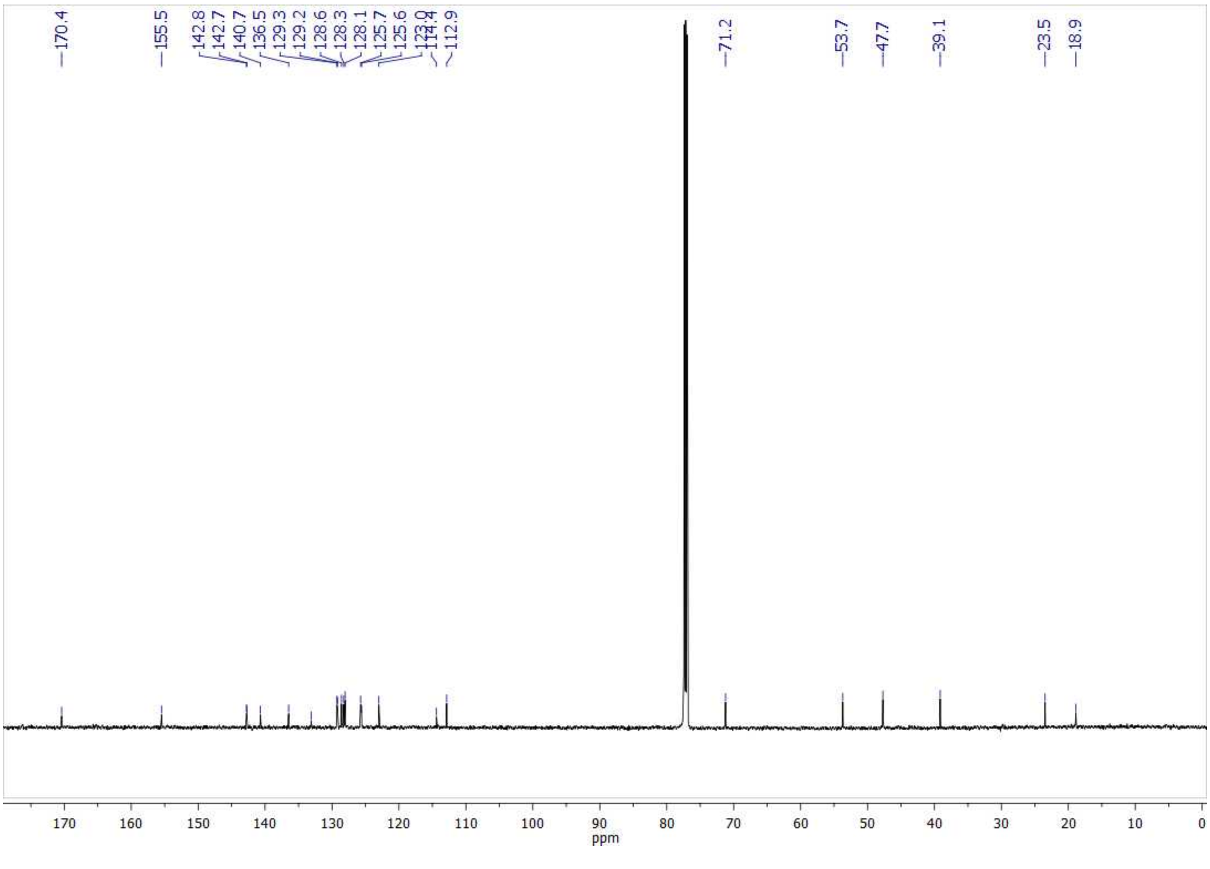

4d

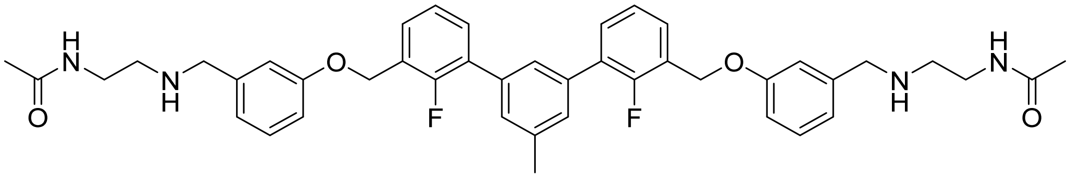

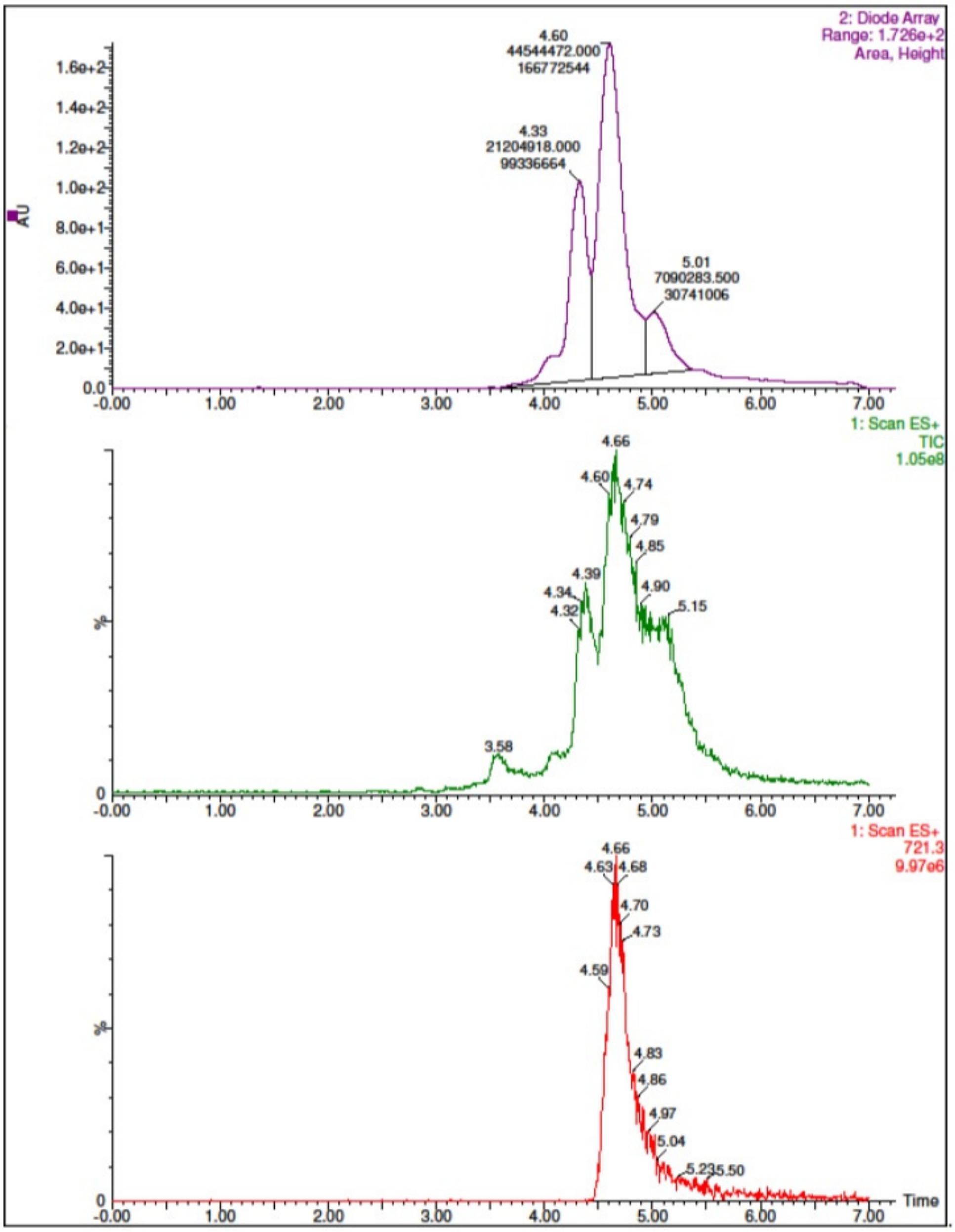
2: Diode Array
Range: 1.726e+2
Area, Height
4.60
44544472.000
166772544
4.33
21204918.000
99336664
5.01
7090283.500
30741006
AU
1: Scan ES+
TIC
1.05e8
1: Scan ES+
721.3
9.97e6
%
Time

***^{1}H NMR** (600 MHz, DMSO-d6)*

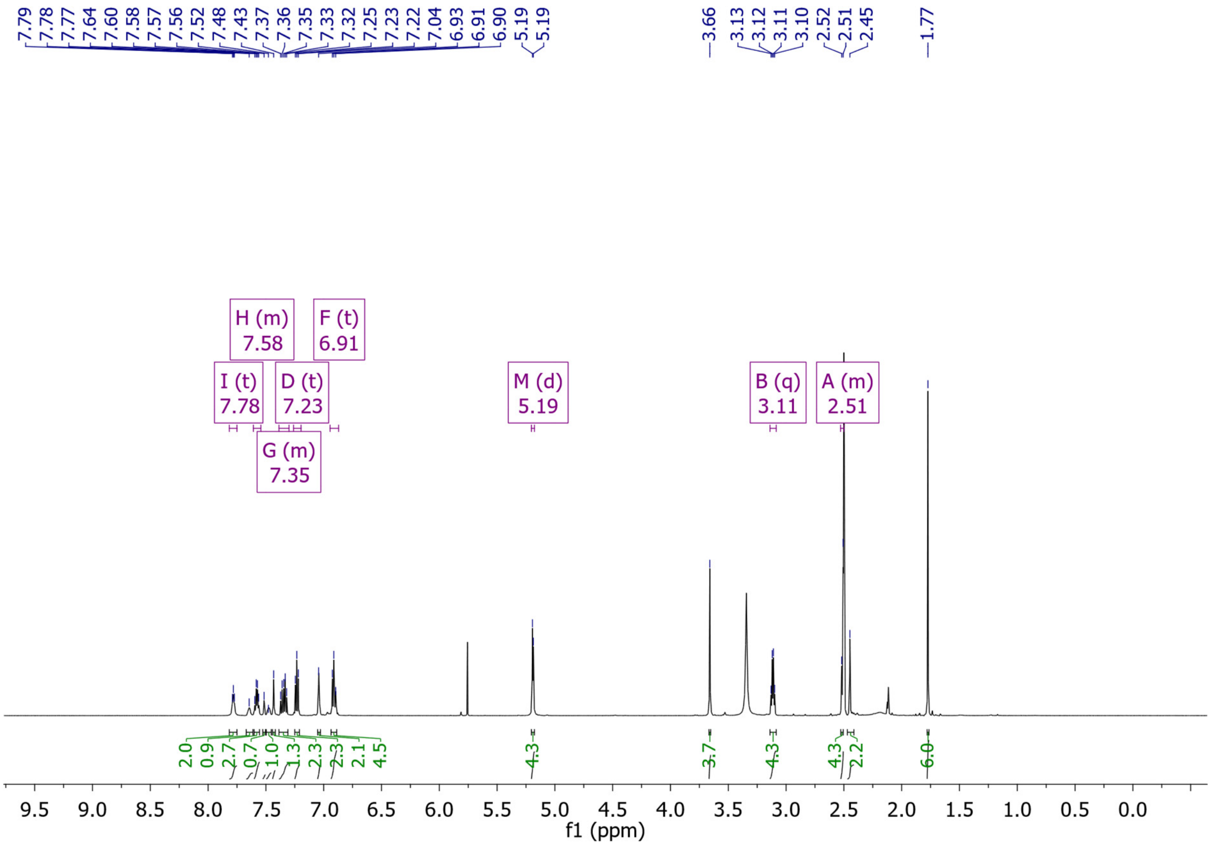

***^{13}C NMR** (151 MHz, CDCl3)*

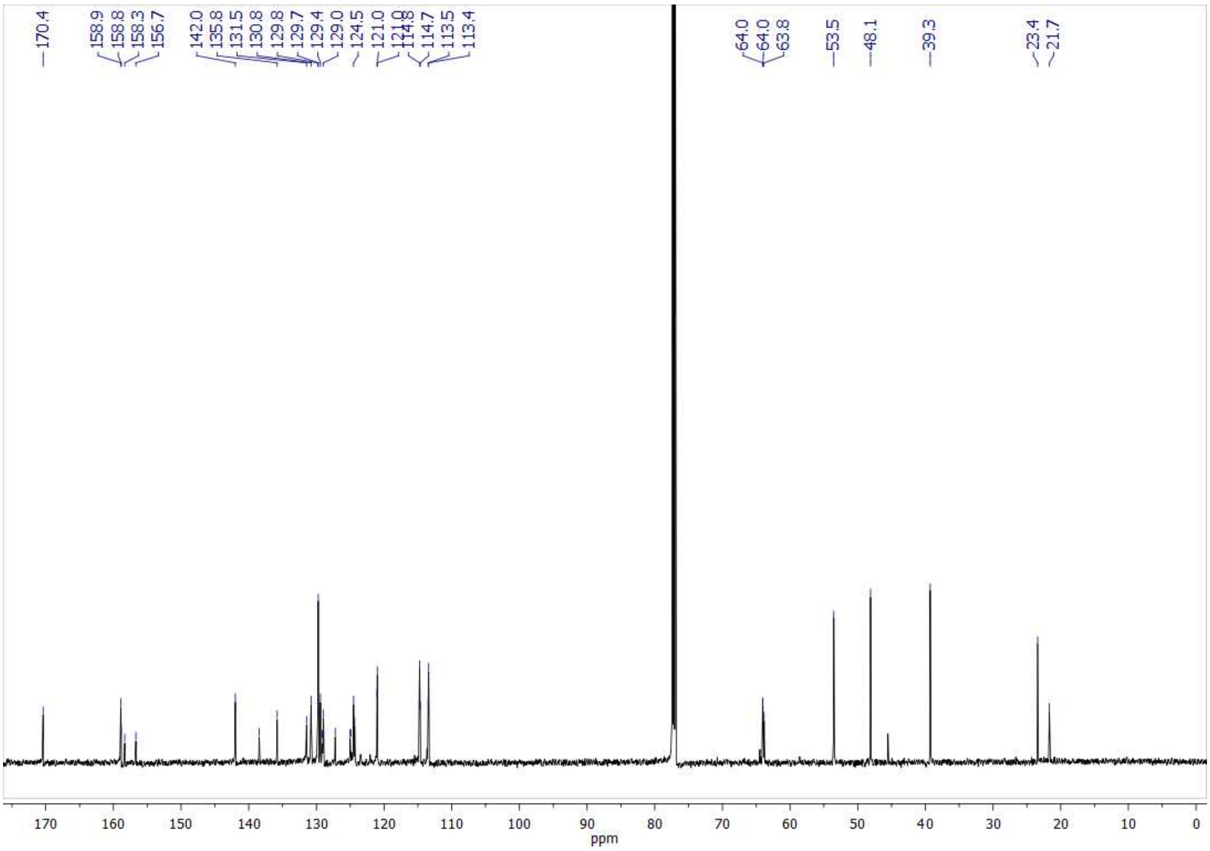

4e

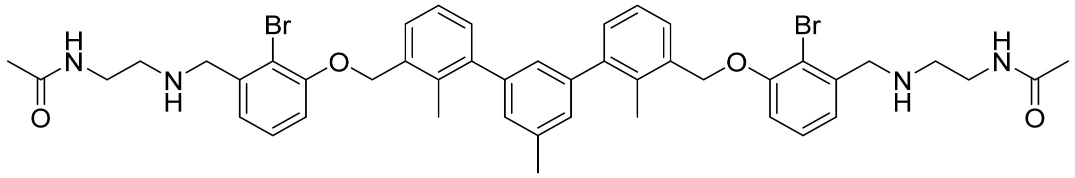
Br
O
N
H
O
Br
O
N
H
O

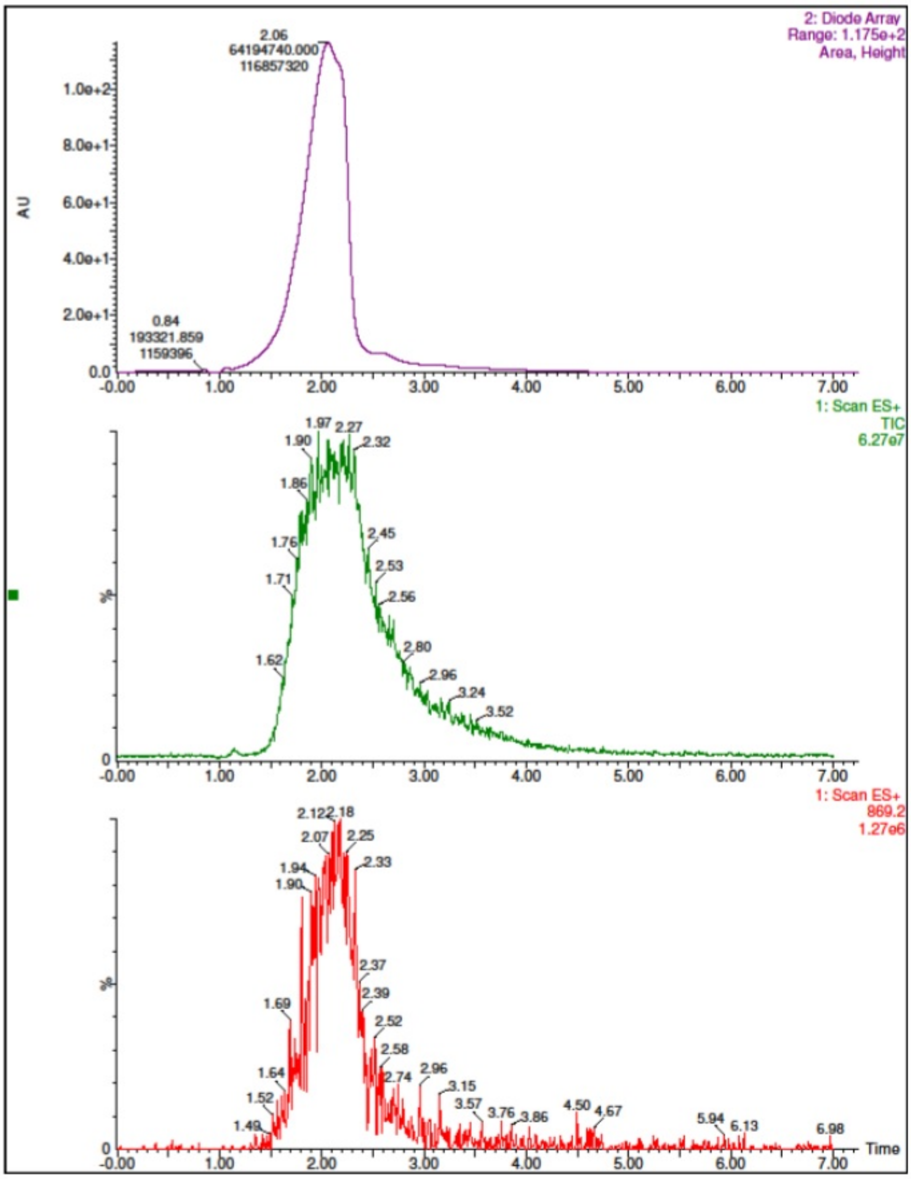
2: Diode Array
Range: 1.175e+2
Area, Height
2.06
64194740.000
116857320
0.84
193321.859
1159396
AU
1: Scan ES+
TIC
6.27e7
1: Scan ES+
869.2
1.27e6
Time

***^{1}H NMR** (600 MHz, $CDCl_3$)*

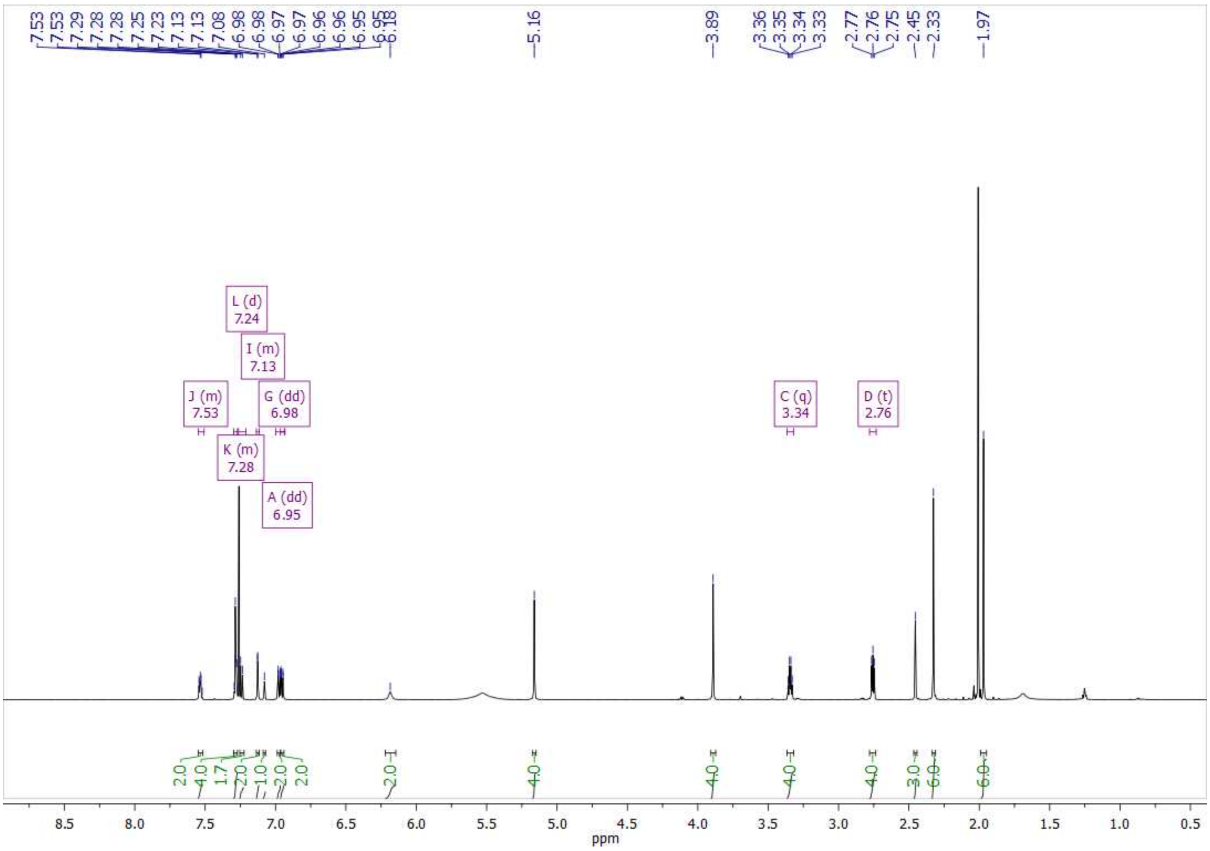

***^{13}C NMR** (151 MHz, $CDCl_3$)*

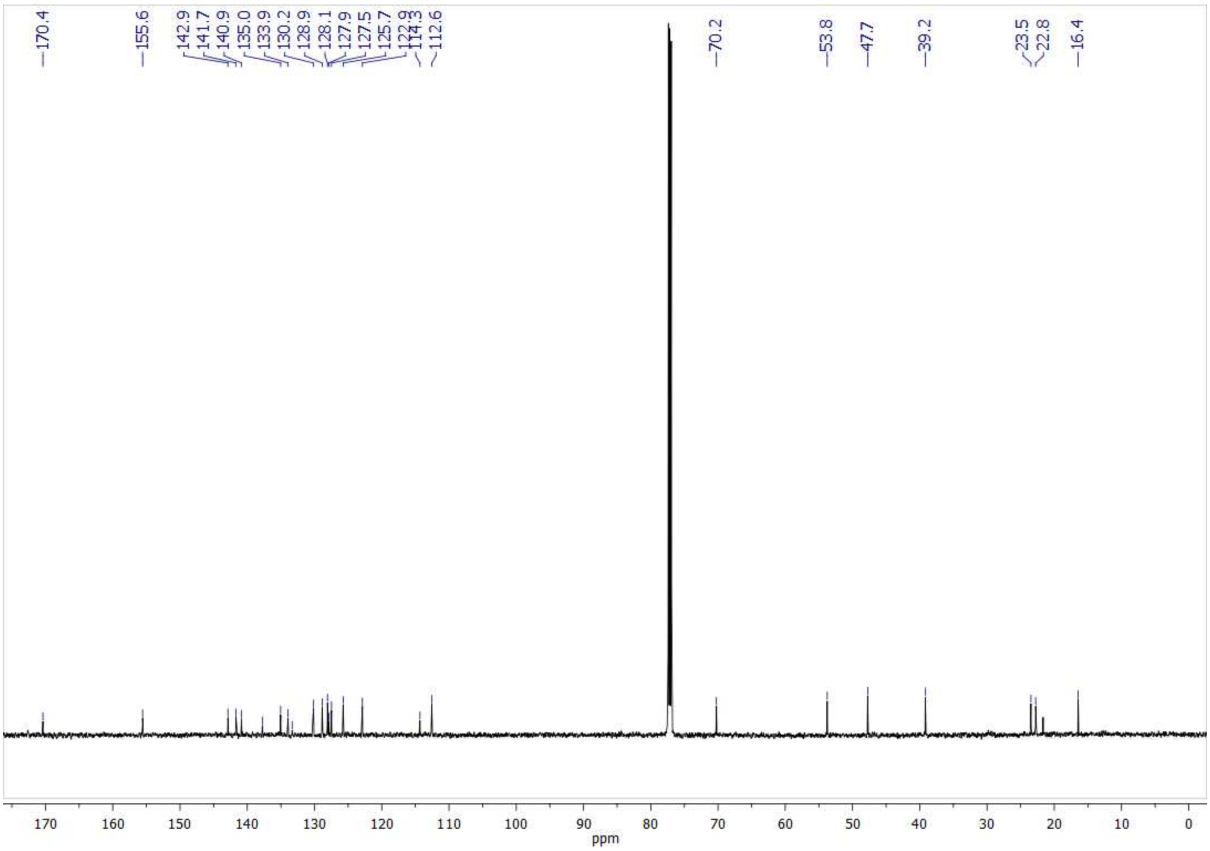

4f

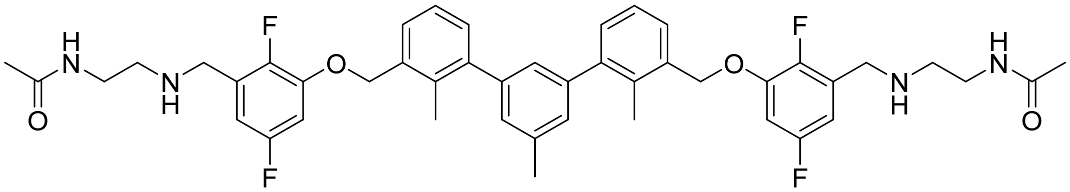

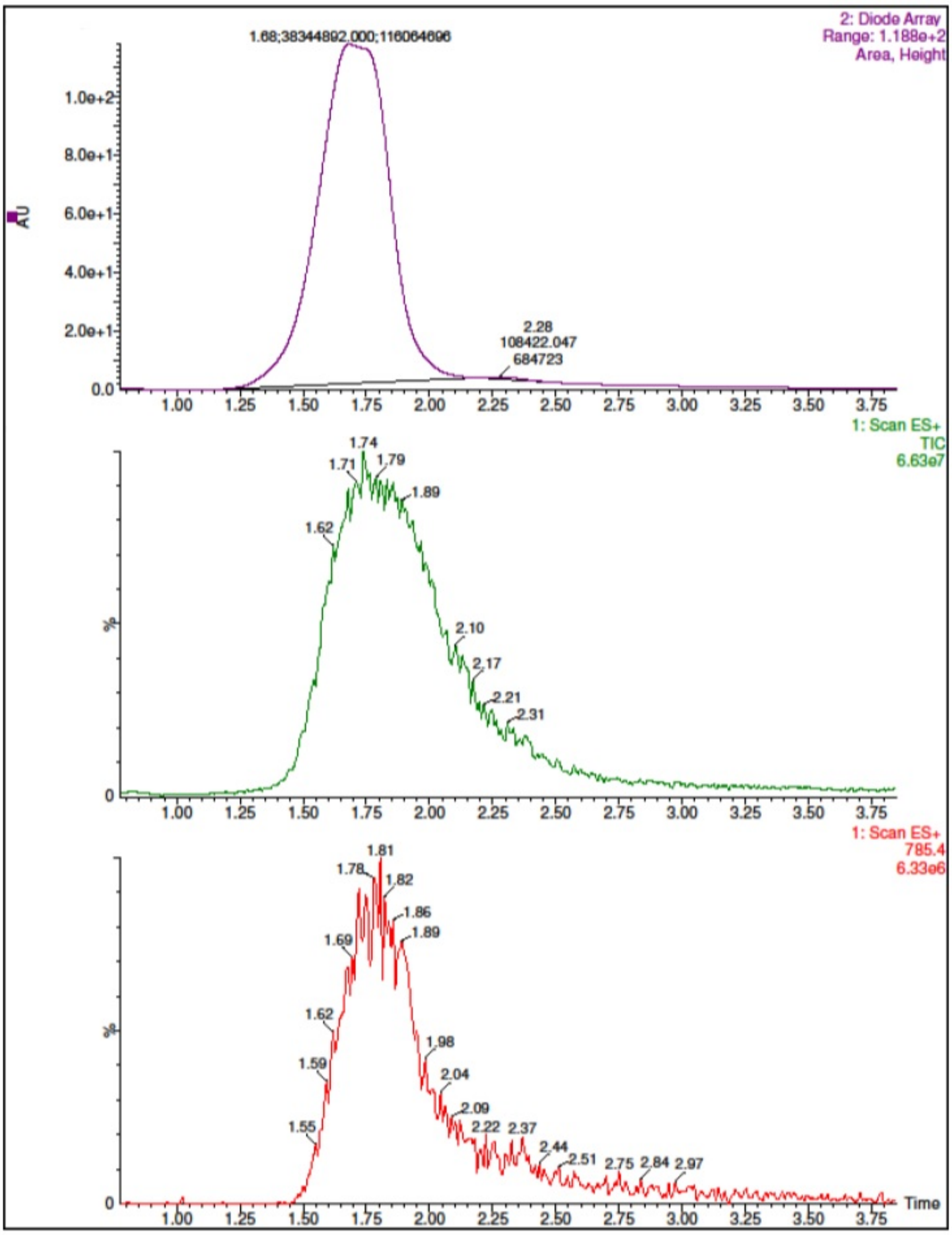
1.68;38344892.000;116064696
2: Diode Array
Range: 1.188e+2
Area, Height
AU
2.28
108422.047
684723
1: Scan ES+
TIC
6.63e7
1: Scan ES+
785.4
6.33e6
Time

***^{1}H NMR** (600 MHz, $CDCl_3$)*

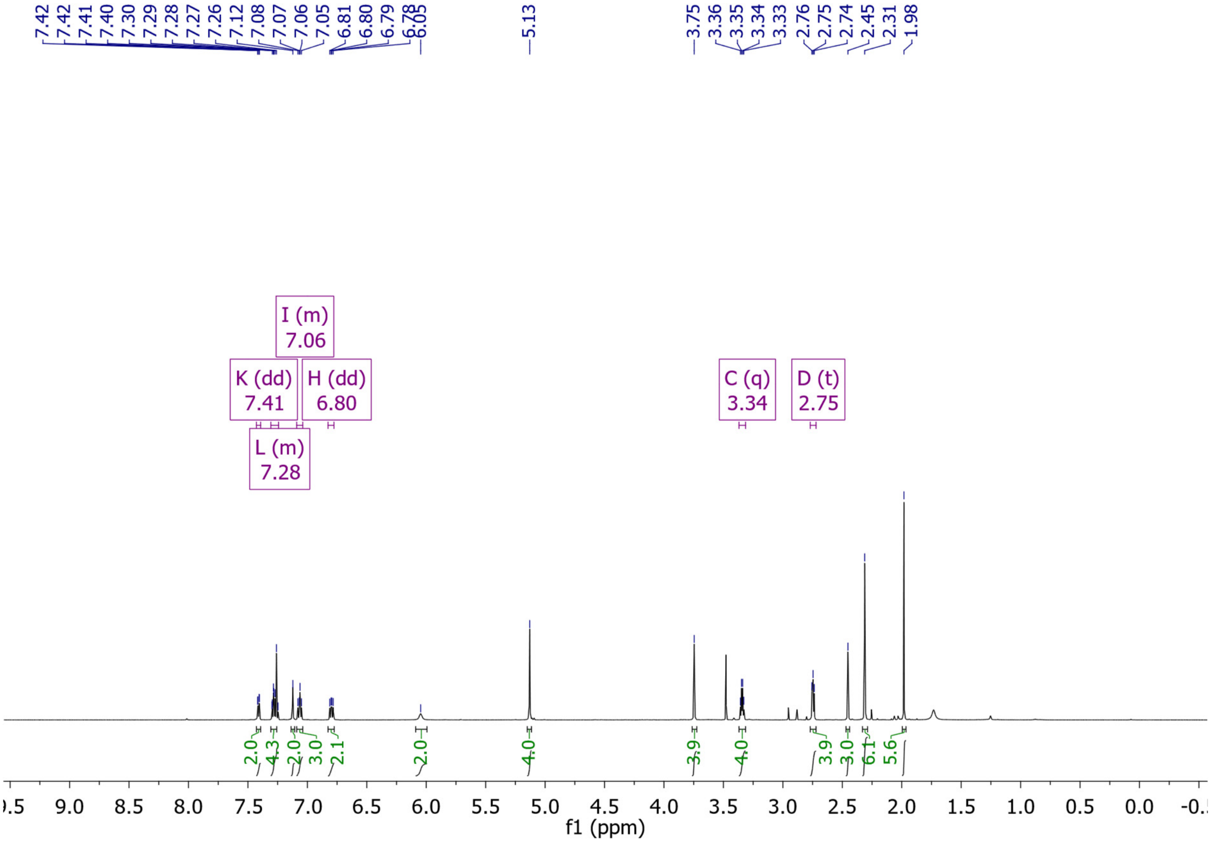

***^{13}C NMR** (151 MHz, $CDCl_3$)*

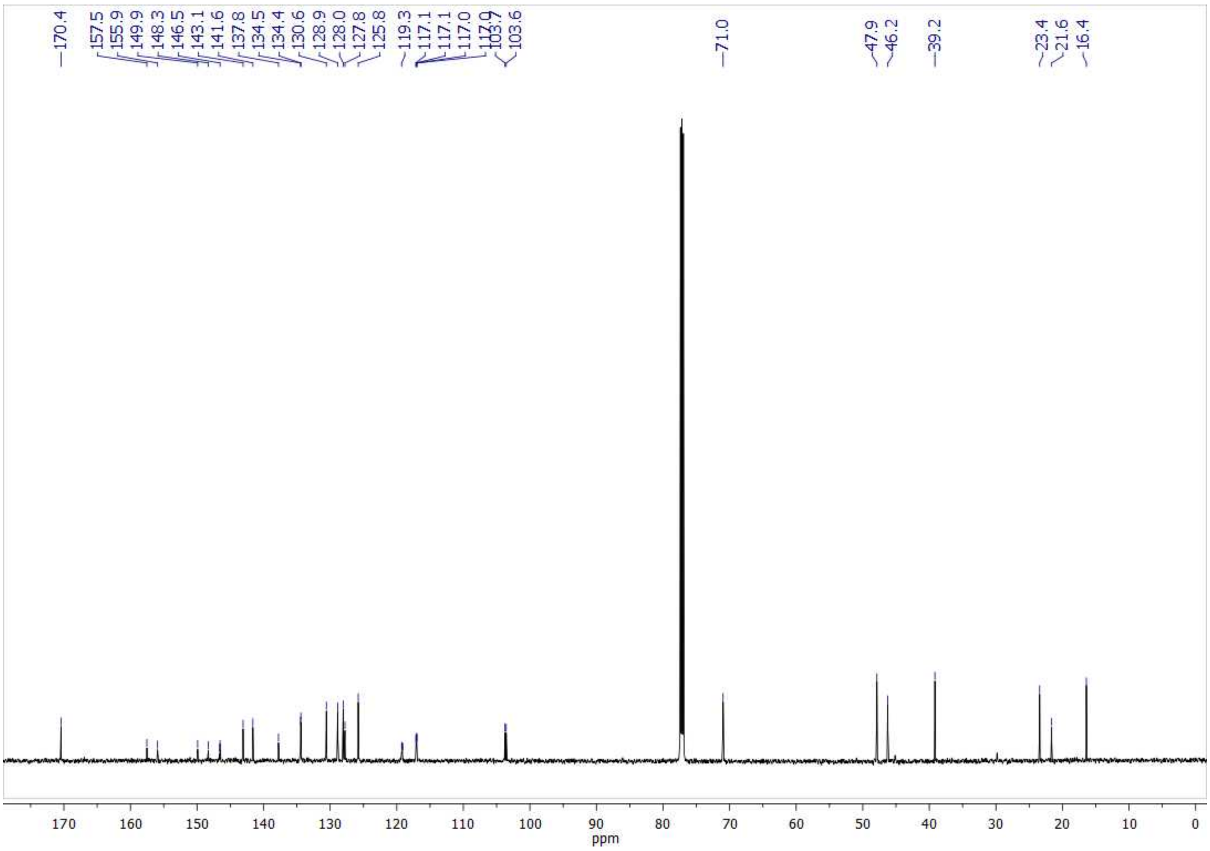

5a

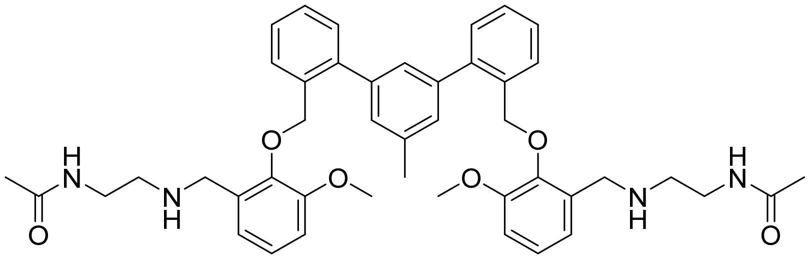

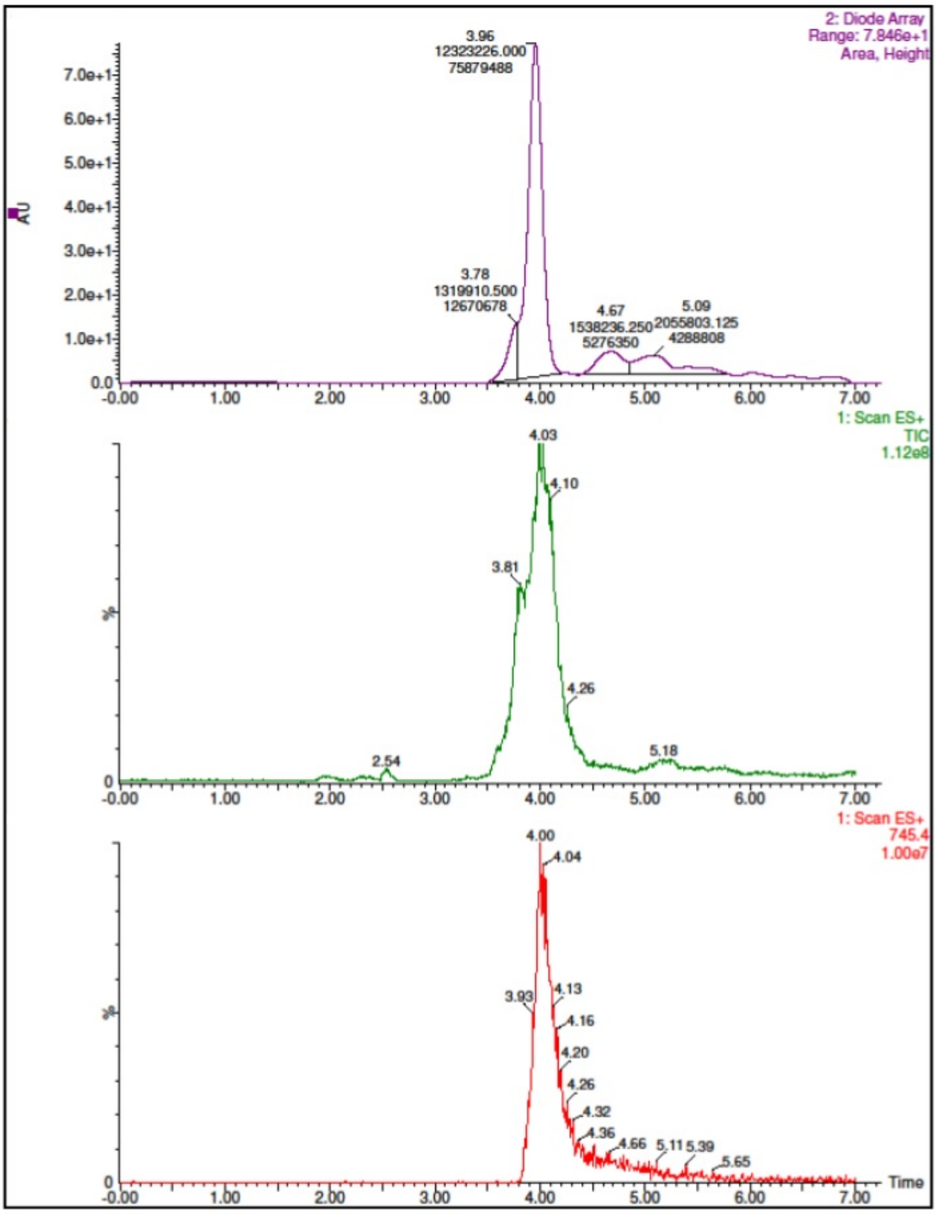

2: Diode Array
Range: 7.846e+1
Area, Height
3.96
12323226.000
75879488
3.78
1319910.500
12670678
4.67
1538236.250
5276350
5.09
2055803.125
4288808
AU
1: Scan ES+
TIC
1.12e8
4.03
4.10
3.81
4.26
2.54
5.18
1: Scan ES+
745.4
1.00e7
4.00
4.04
3.93
4.13
4.16
4.20
4.26
4.32
4.36
4.66
5.11
5.39
5.65
Time

***^{1}H NMR** (600 MHz, $CDCl_3$)*

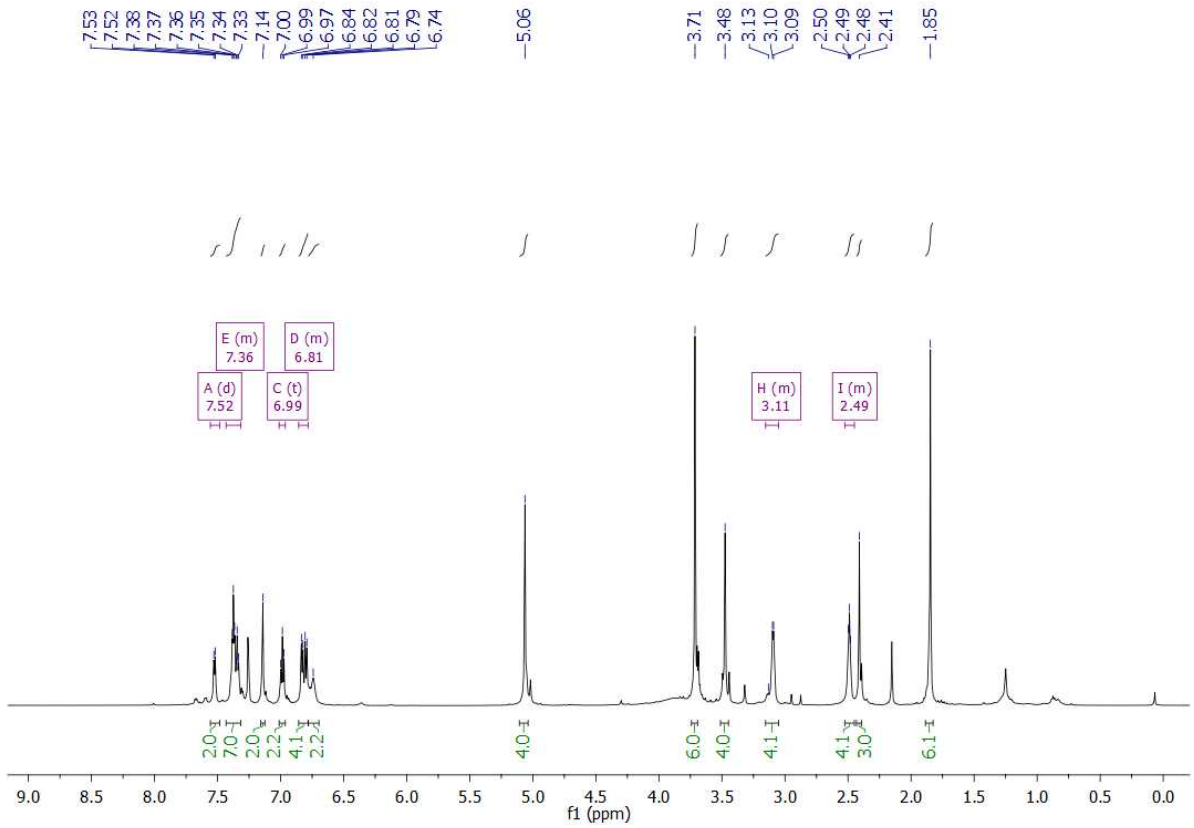

***^{13}C NMR** (151 MHz, $CDCl_3$)*

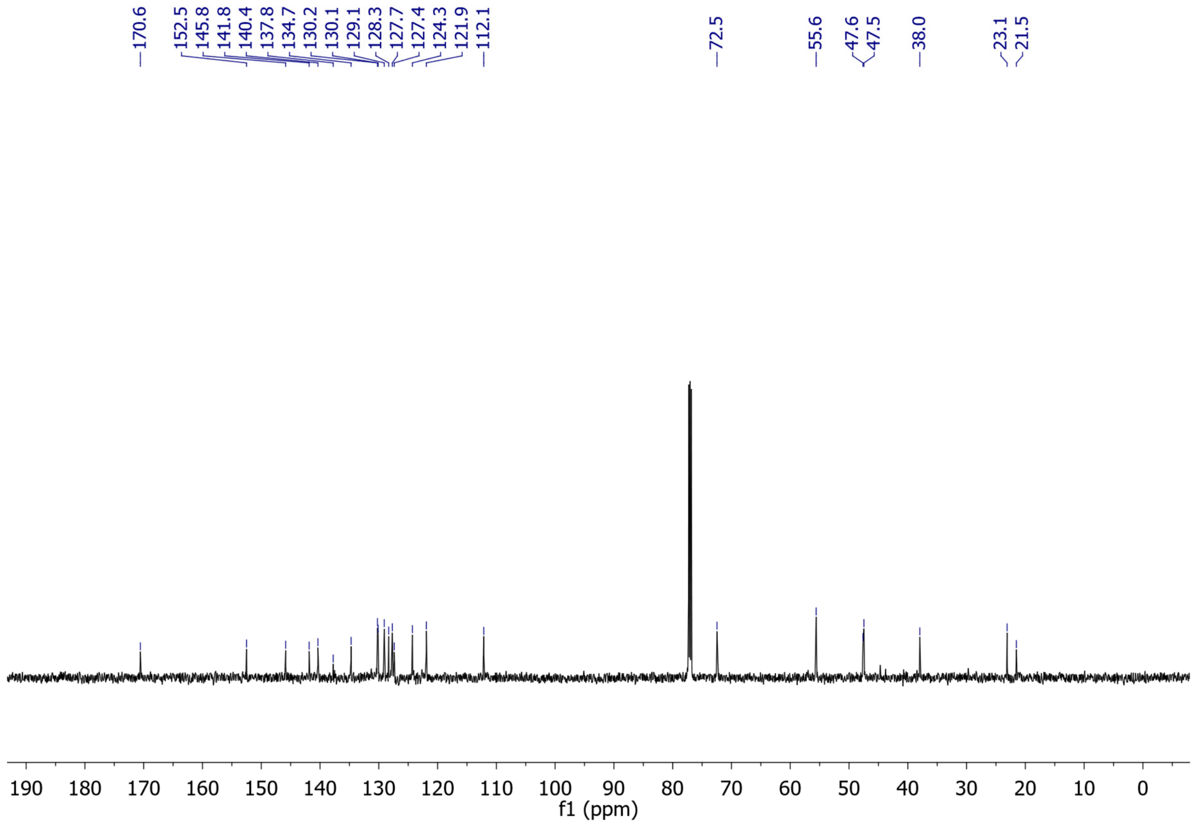

6a

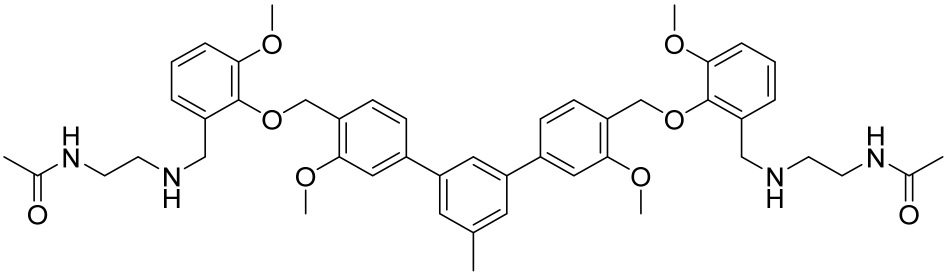

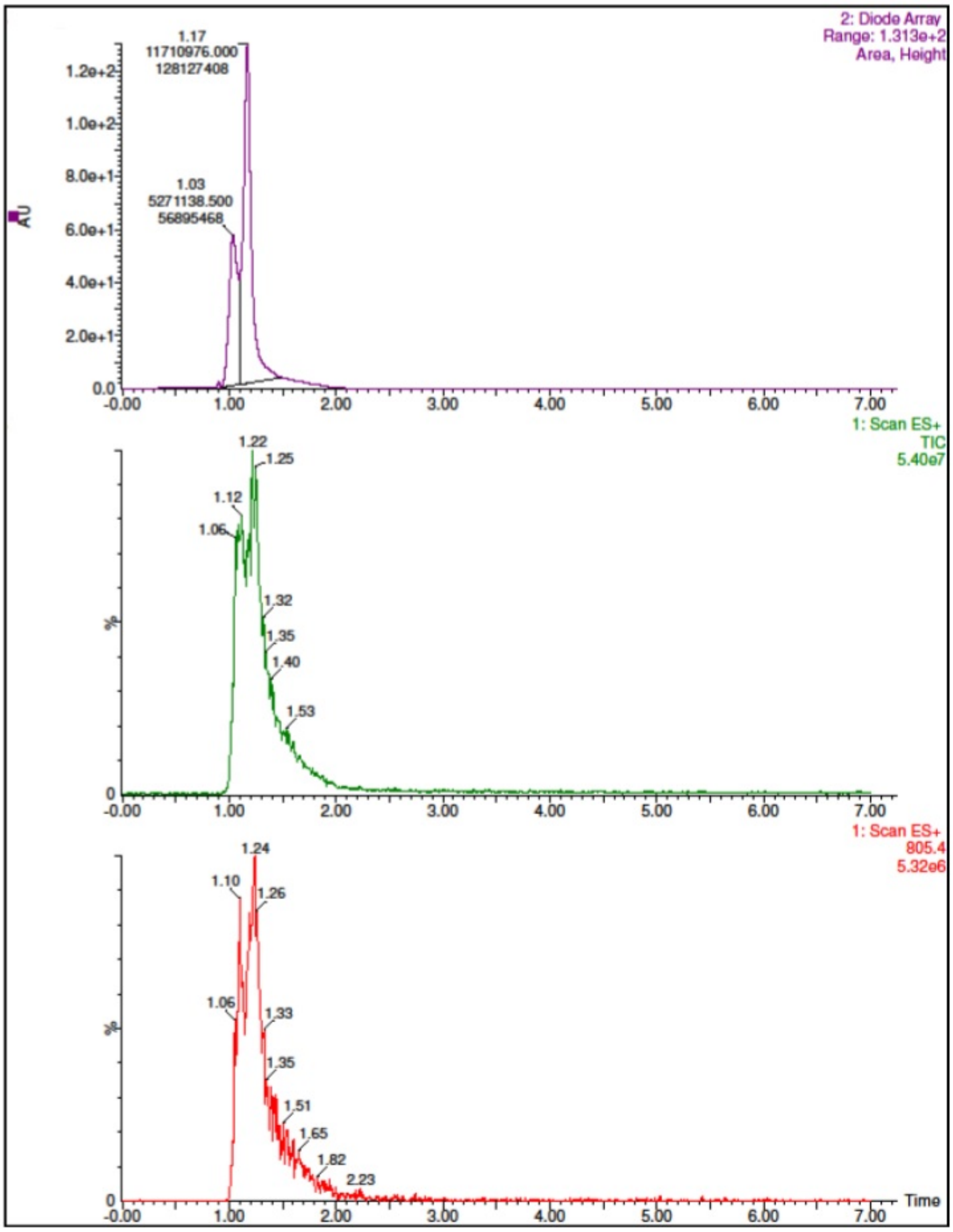

2: Diode Array
Range: 1.313e+2
Area, Height
1.17
11710976.000
128127408
1.03
5271138.500
56895468
AU
1.2e+2
1.0e+2
8.0e+1
6.0e+1
4.0e+1
2.0e+1
0.0
1: Scan ES+
TIC
5.40e7
1.22
1.25
1.12
1.06
1.32
1.35
1.40
1.53
%
1: Scan ES+
805.4
5.32e6
1.24
1.10
1.26
1.06
1.33
1.35
1.51
1.65
1.82
2.23
Time
-0.00
1.00
2.00
3.00
4.00
5.00
6.00
7.00

¹H NMR *(600 MHz, MeOD-d_4)*

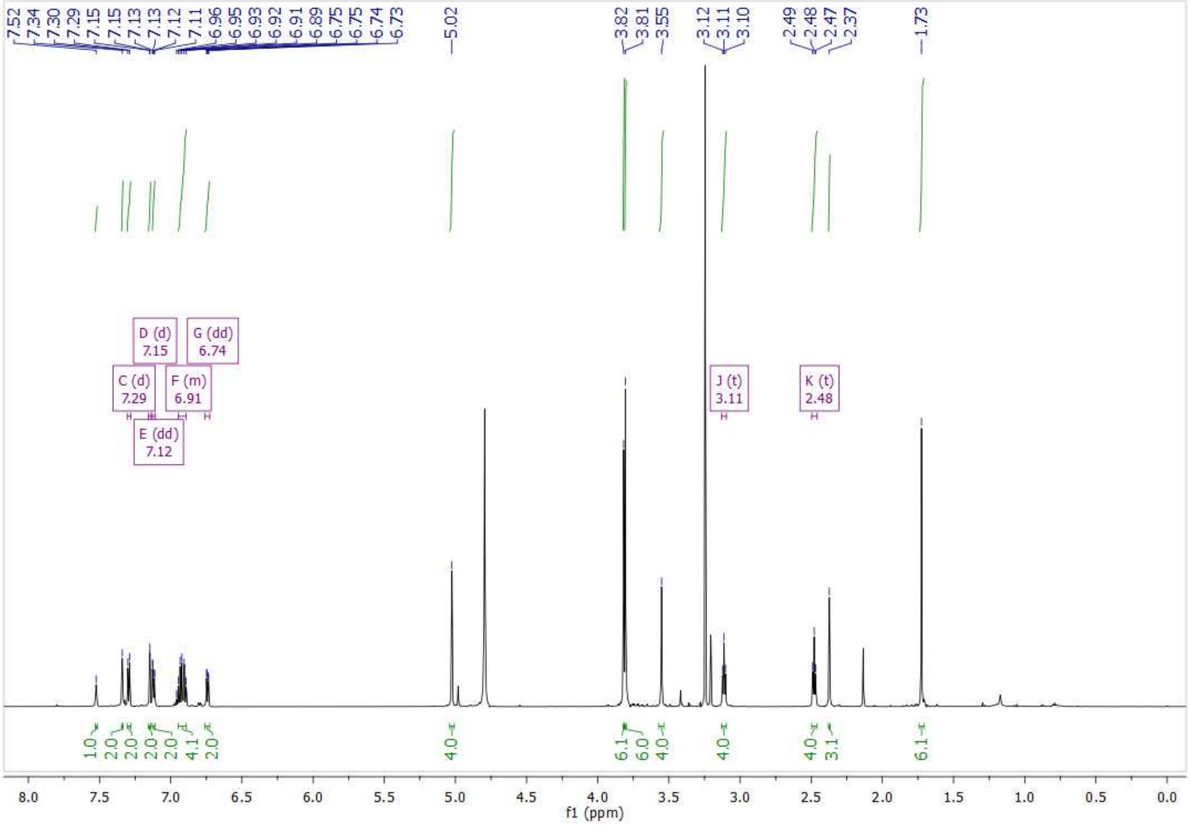

¹³C NMR *(151 MHz, MeOD-d_4)*

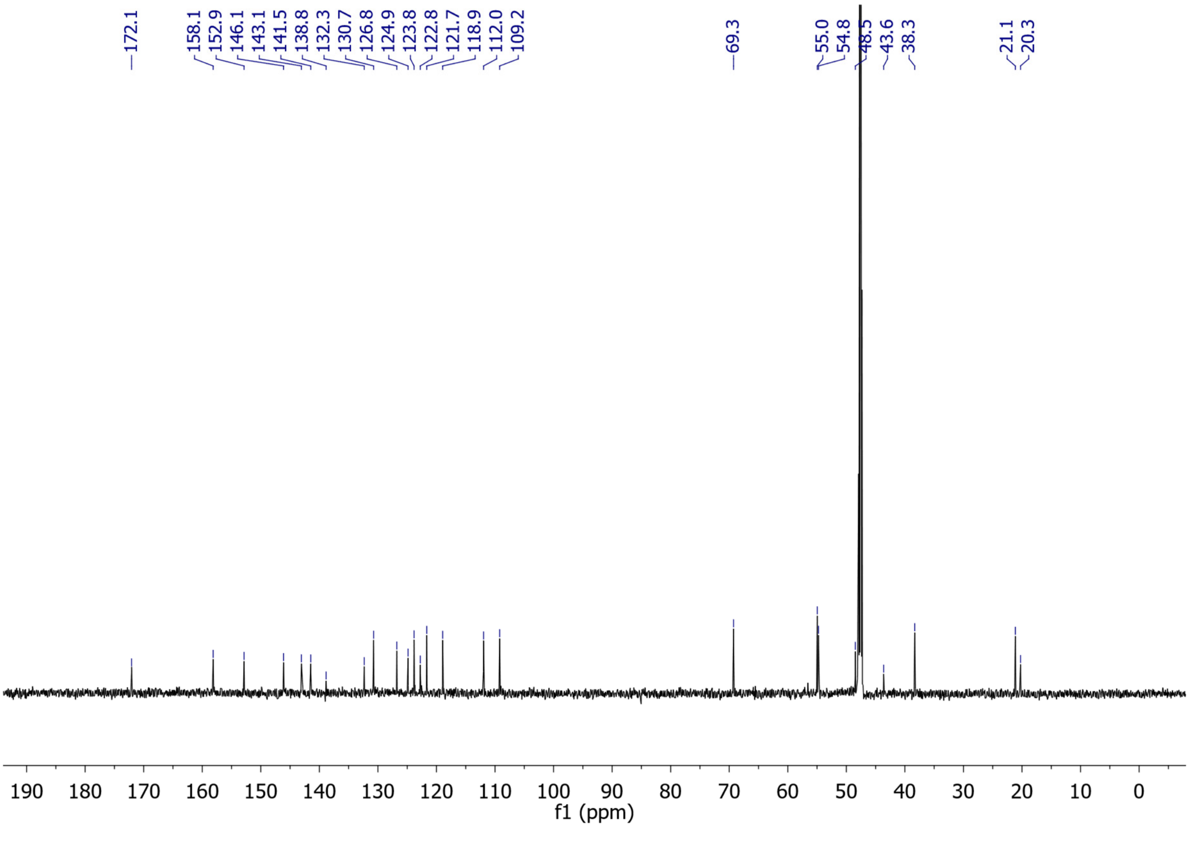

7a

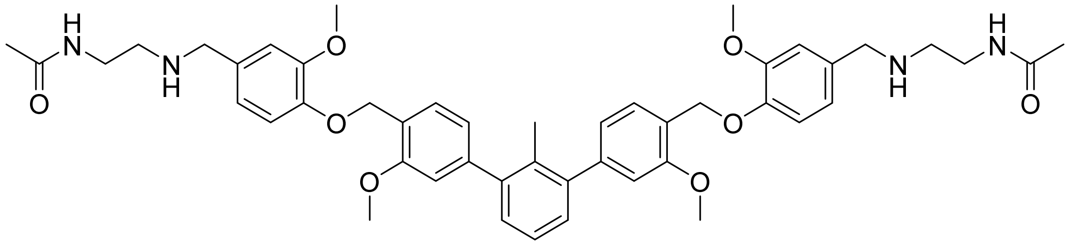

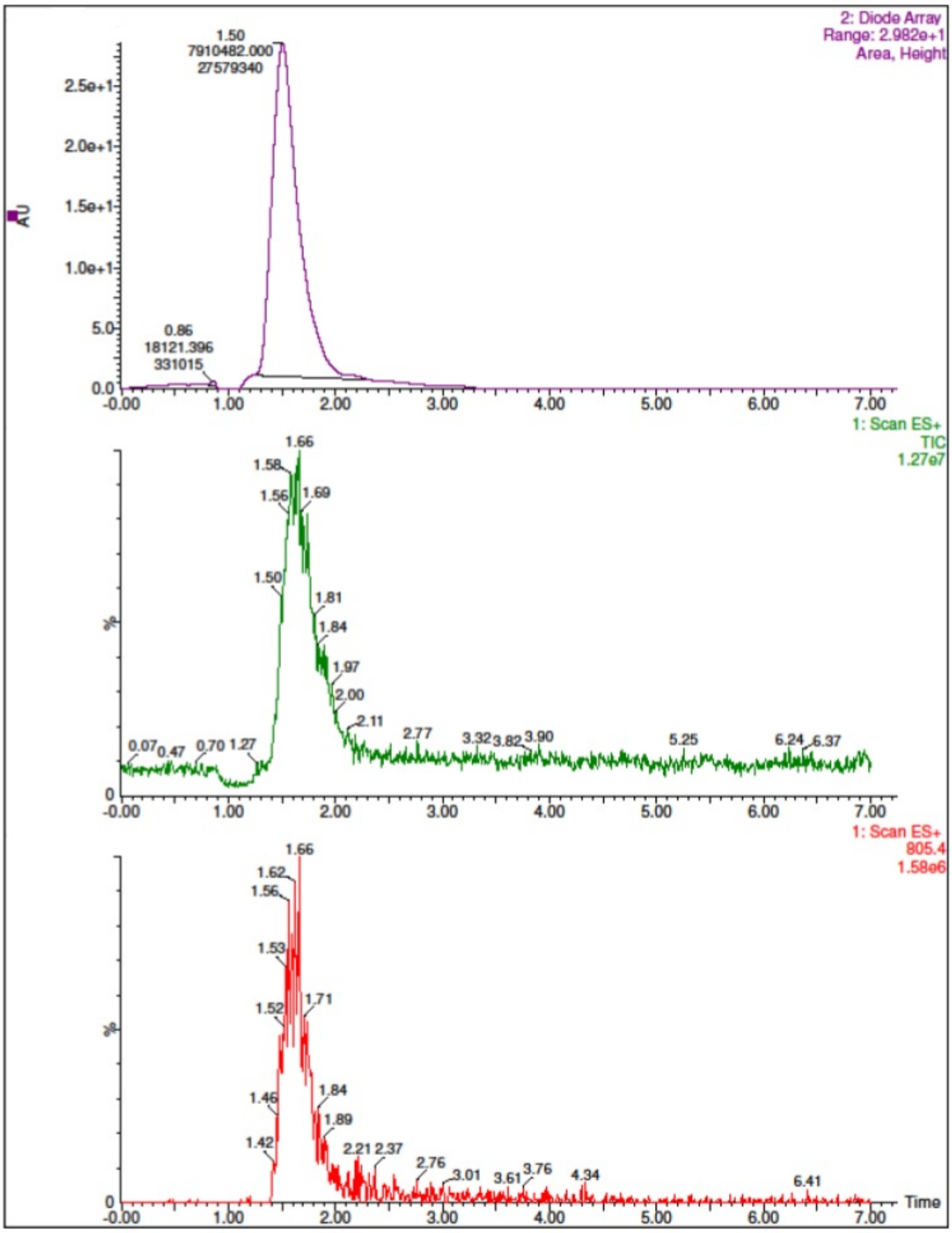
2: Diode Array
Range: 2.982e+1
Area, Height
1.50
7910482.000
27579340
0.86
18121.396
331015
AU
1: Scan ES+
TIC
1.27e7
1: Scan ES+
805.4
1.58e6
Time

***^{1}H NMR** (600 MHz, $CDCl_3$)*

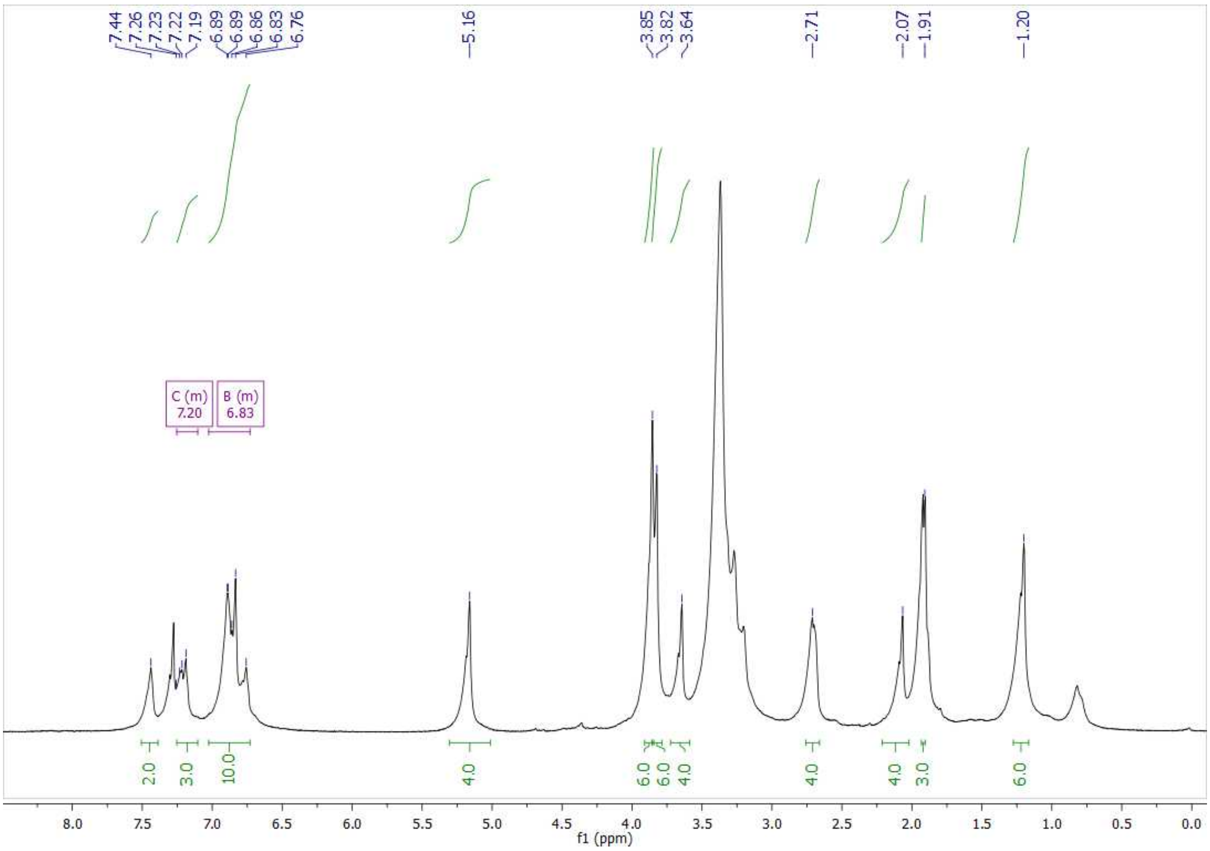

7b

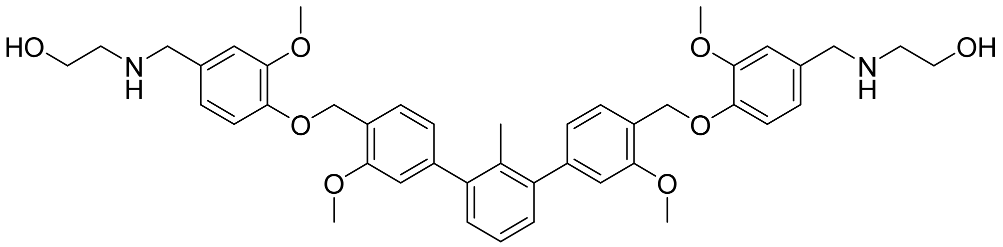
HO
N
H
O
O
O
O
O
O
O
O
N
H
OH

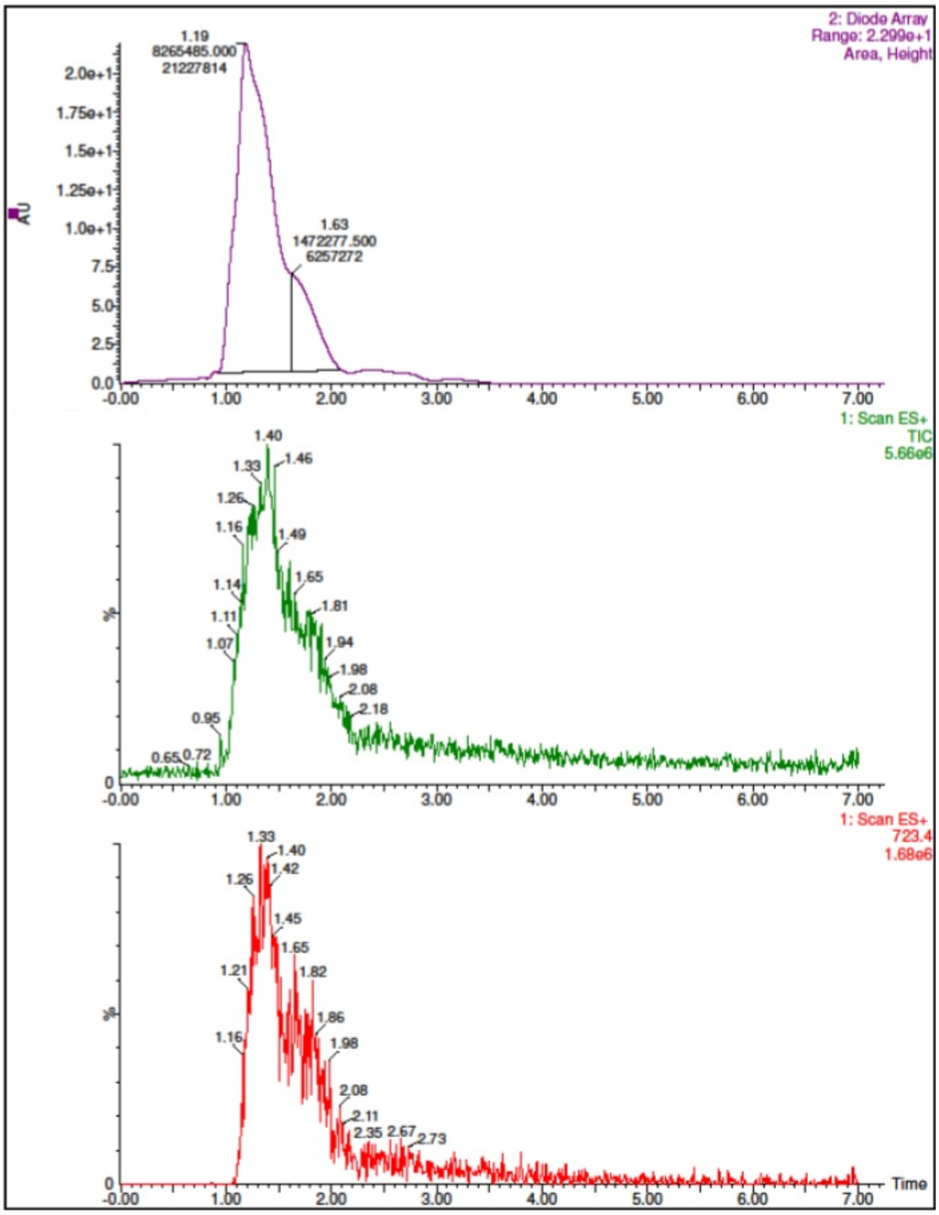
2: Diode Array
Range: 2.299e+1
Area, Height
1.19
8265485.000
21227814
1.63
1472277.500
6257272
AU
1: Scan ES+
TIC
5.66e6
1: Scan ES+
723.4
1.68e6
Time

*[1]**H NMR** (600 MHz, CDCl$_3$)*

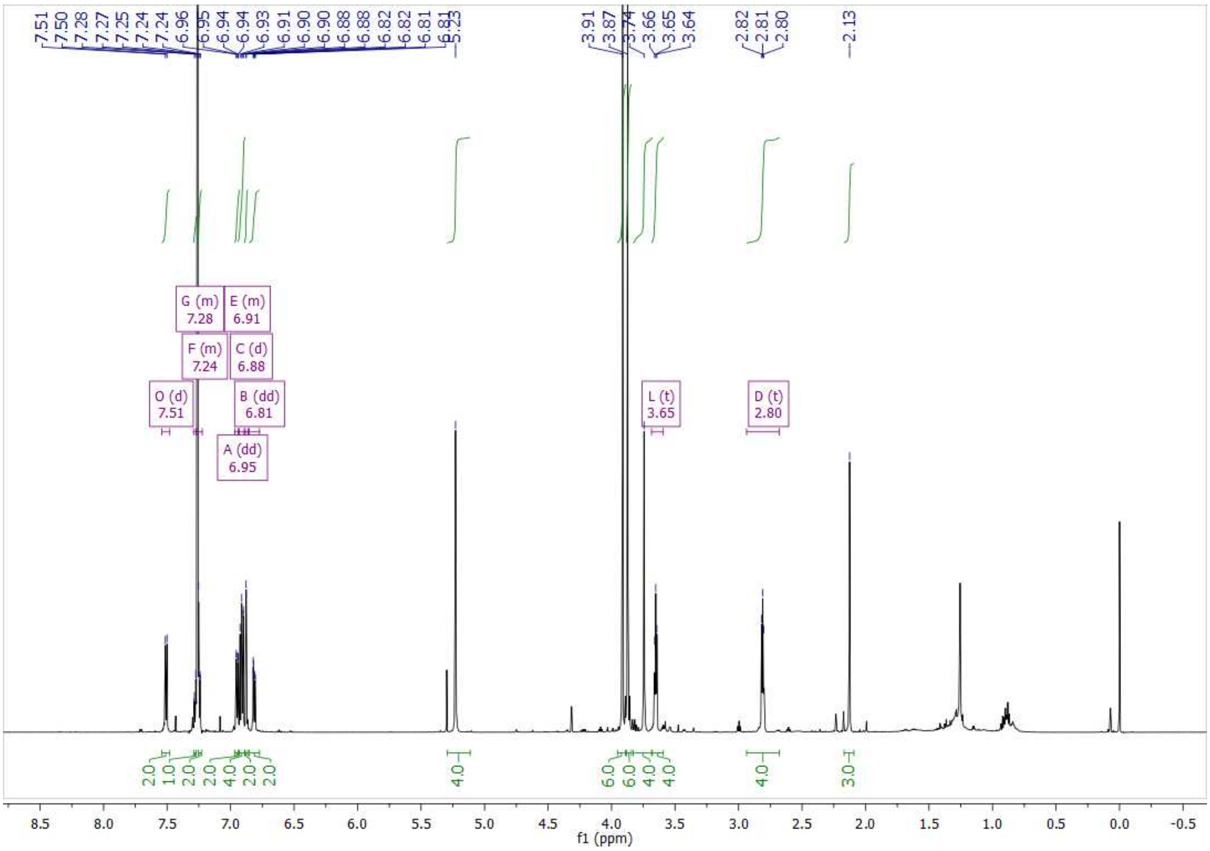

*[13]**C NMR** (151 MHz, CDCl$_3$)*

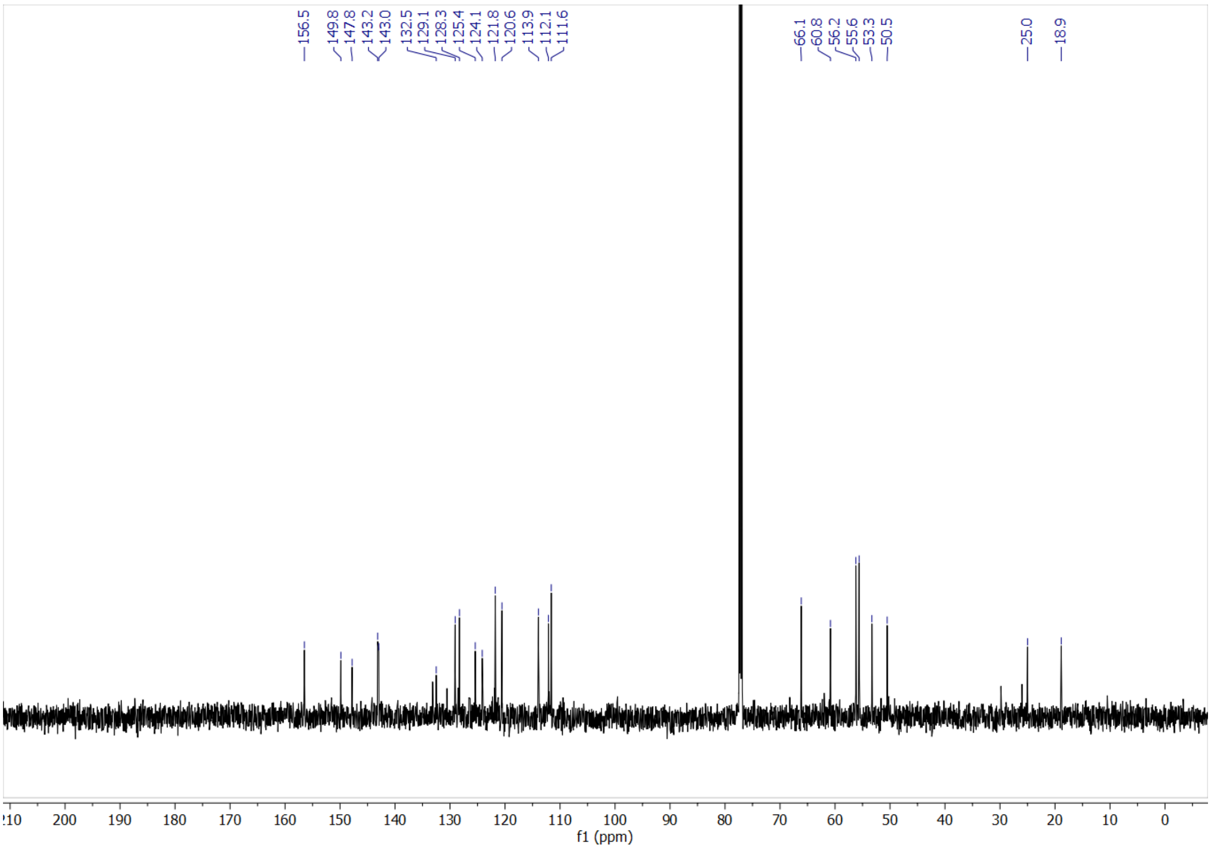

7c

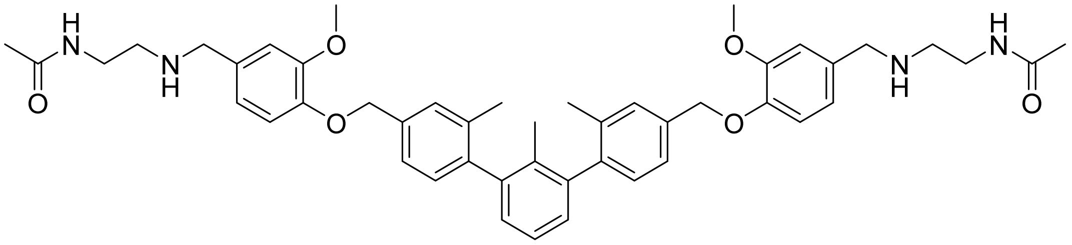

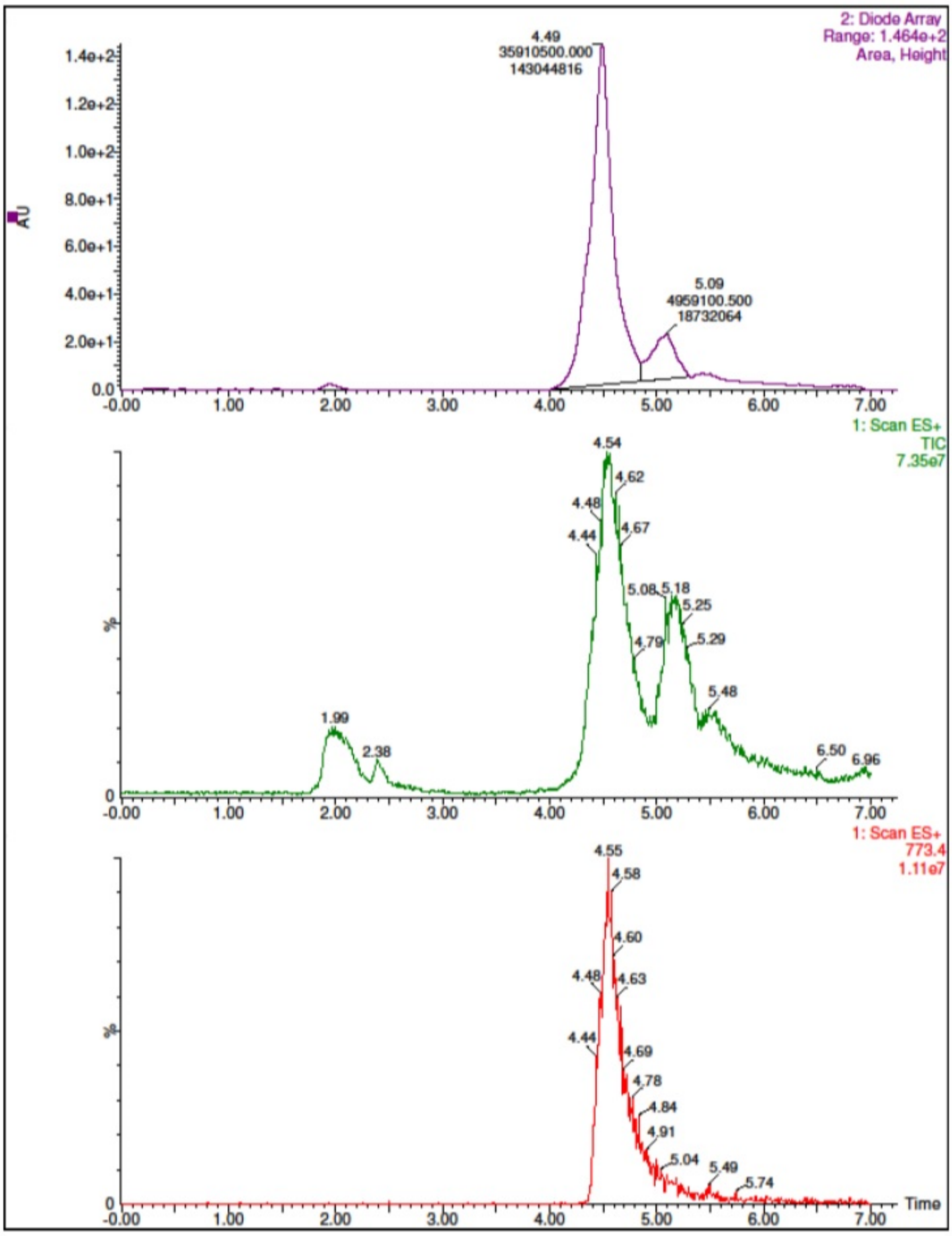

2: Diode Array
Range: 1.464e+2
Area, Height
4.49
35910500.000
143044816
5.09
4959100.500
18732064
AU
1: Scan ES+
TIC
7.35e7
4.54
4.62
4.48
4.44
4.67
5.08
5.18
5.25
4.79
5.29
5.48
1.99
2.38
6.50
6.96
1: Scan ES+
773.4
1.11e7
4.55
4.58
4.60
4.48
4.63
4.44
4.69
4.78
4.84
4.91
5.04
5.49
5.74
Time

^{1}H NMR *(600 MHz, DMSO-d6)*

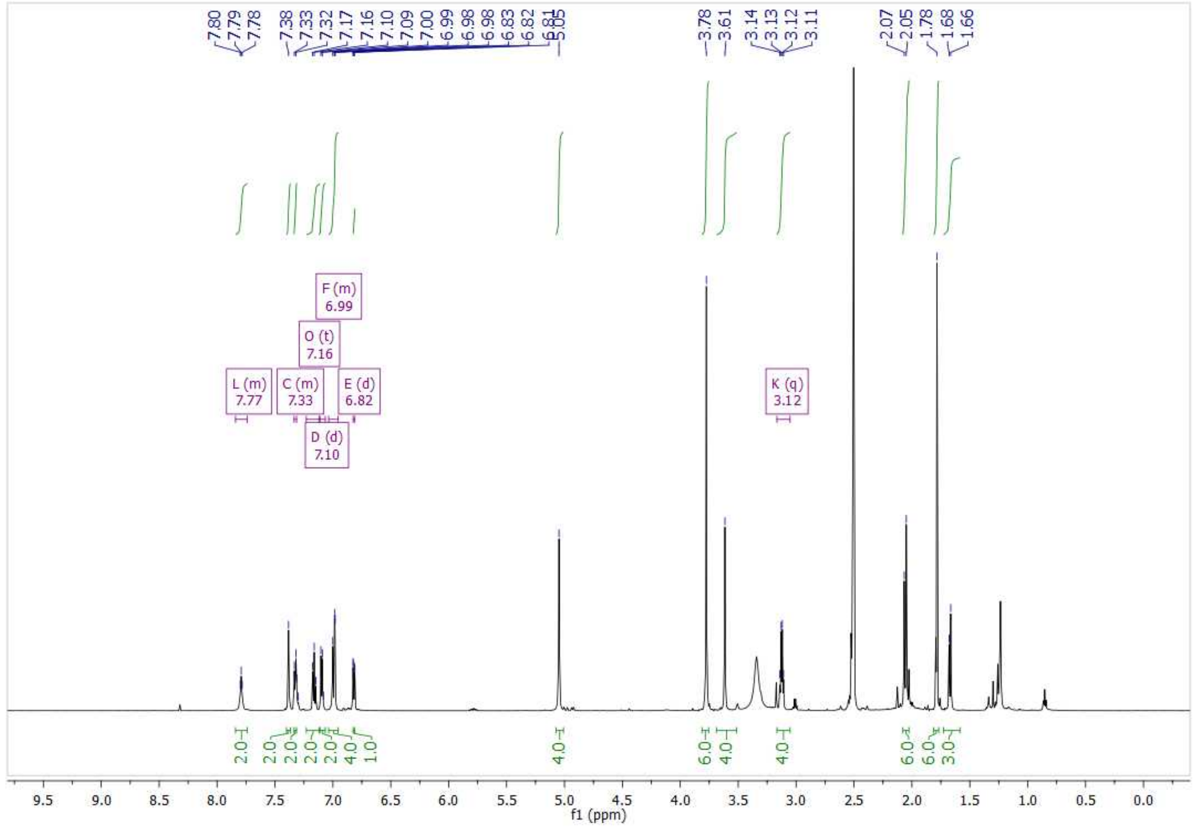

^{13}C NMR *(151 MHz, DMSO-d6)*

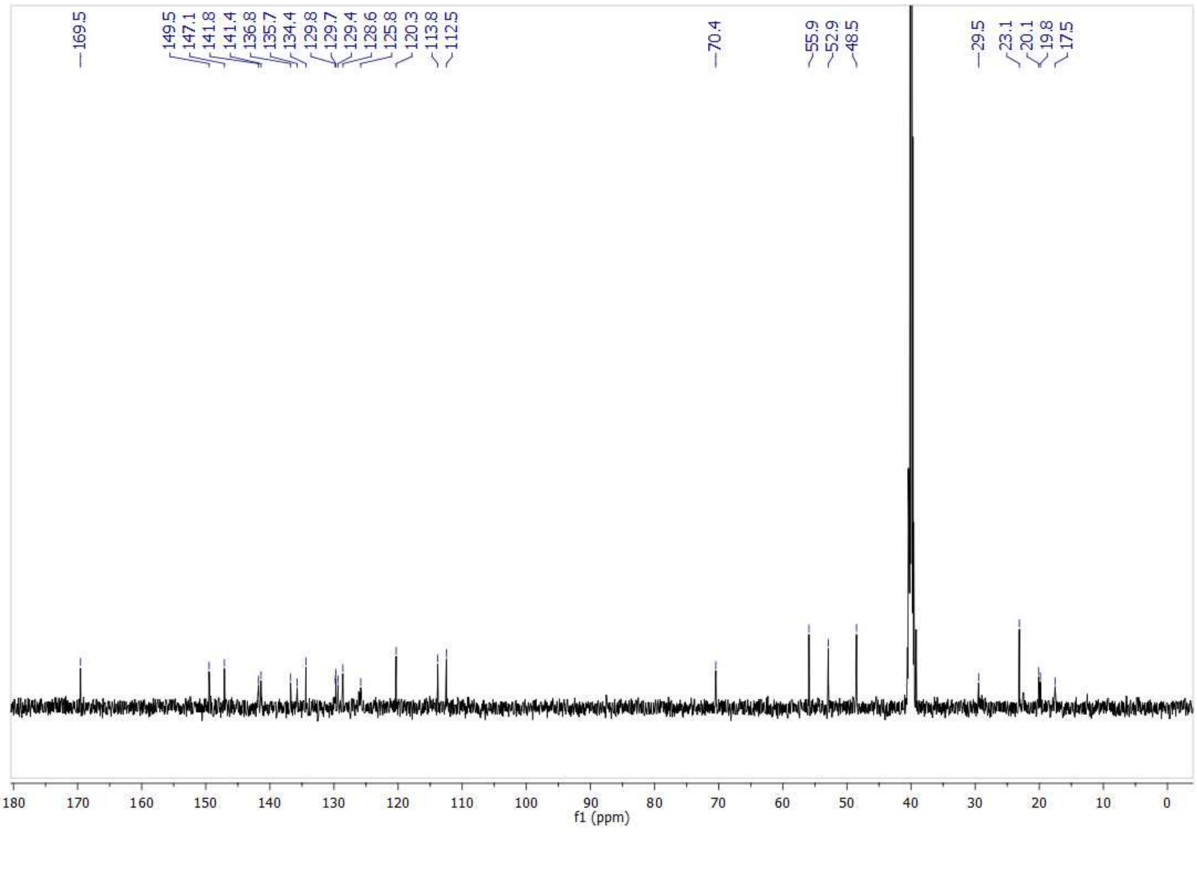

7d

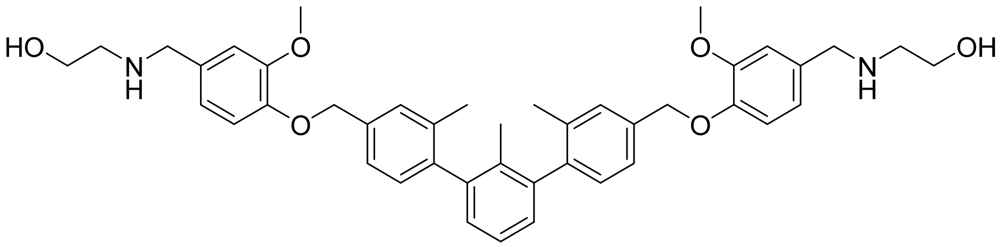
HO
N
H
O
O
O
O
N
H
OH

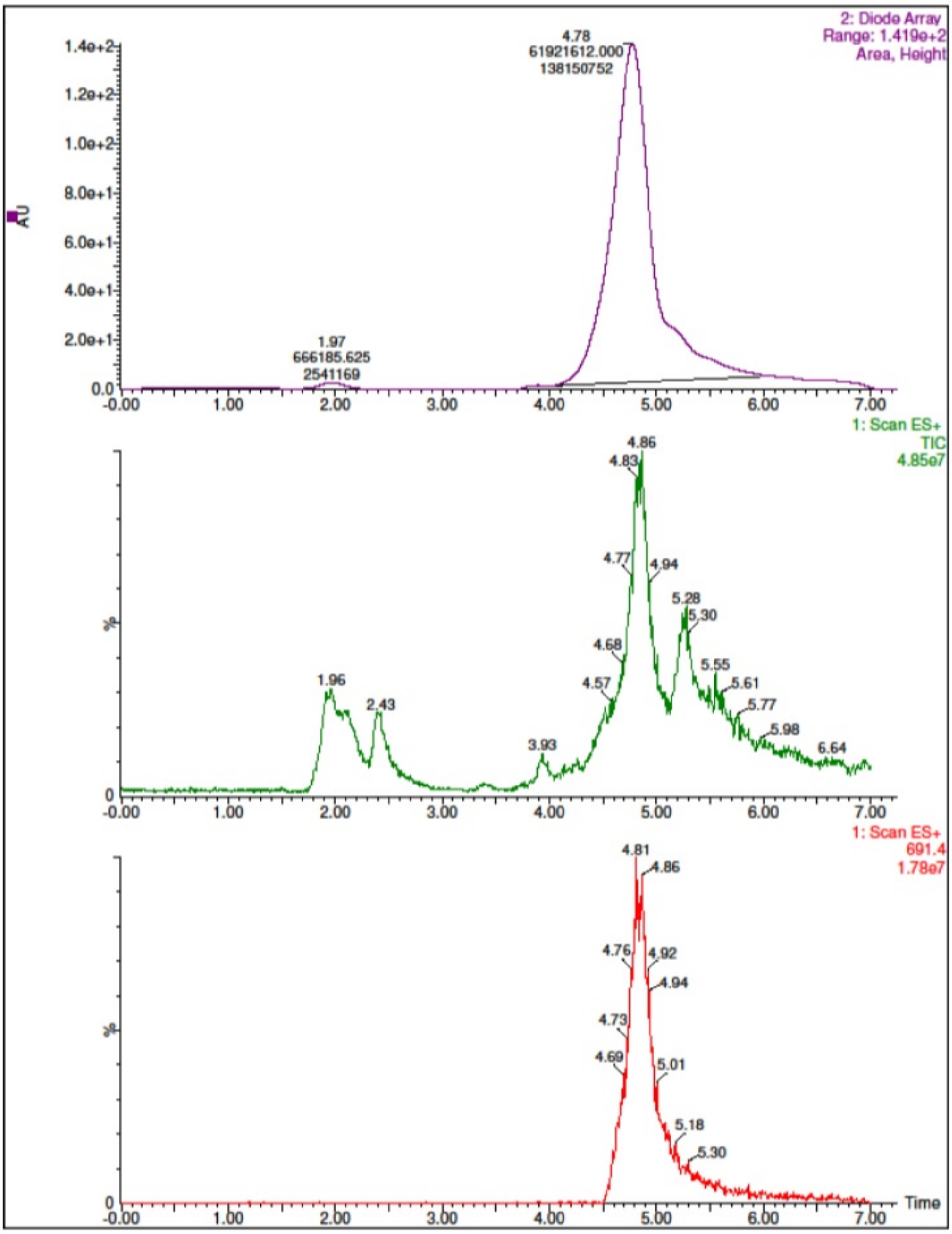
2: Diode Array
Range: 1.419e+2
Area, Height
4.78
61921612.000
138150752
1.97
666185.625
2541169
AU
1: Scan ES+
TIC
4.85e7
4.86
4.83
4.77
4.94
5.28
5.30
4.68
5.55
4.57
5.61
5.77
5.98
6.64
1.96
2.43
3.93
%
1: Scan ES+
691.4
1.78e7
4.81
4.86
4.76
4.92
4.94
4.73
4.69
5.01
5.18
5.30
Time
-0.00
1.00
2.00
3.00
4.00
5.00
6.00
7.00

^{1}H NMR *(600 MHz, DMSO-d_6)*

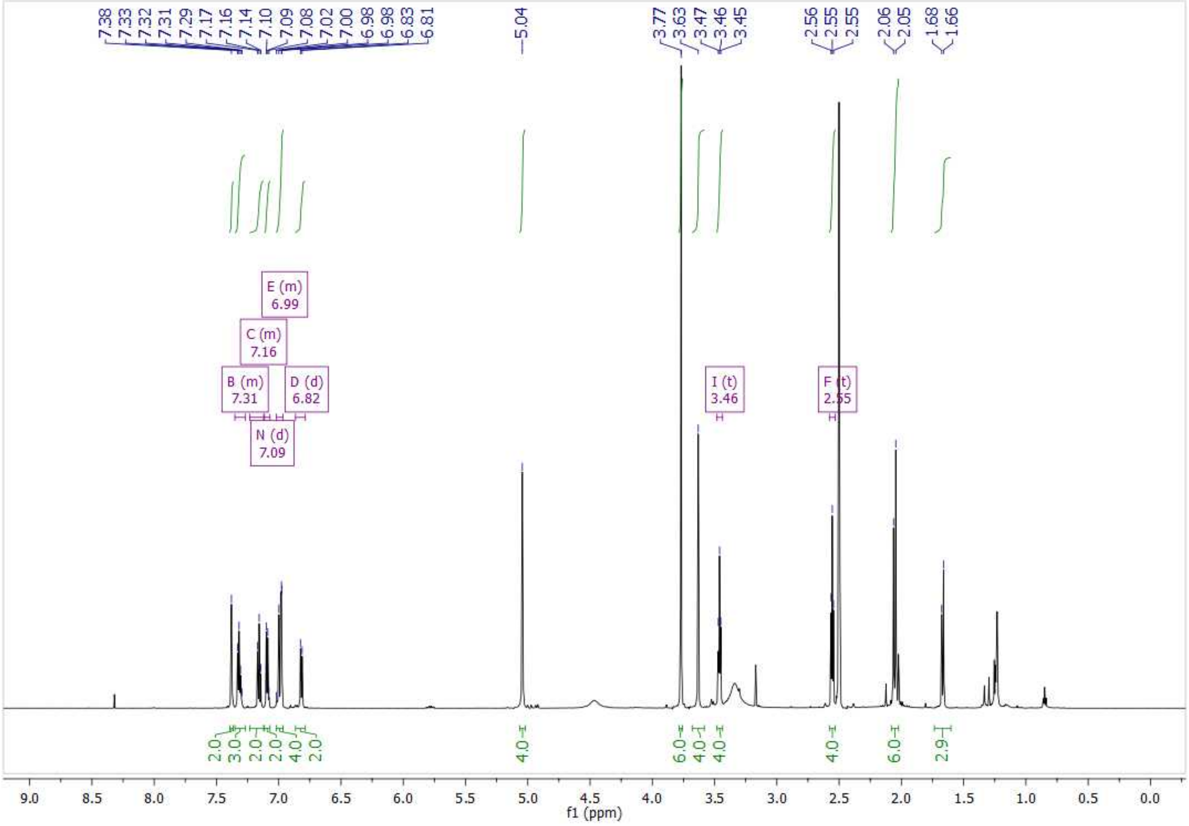

^{13}C NMR *(151 MHz, DMSO-d_6)*

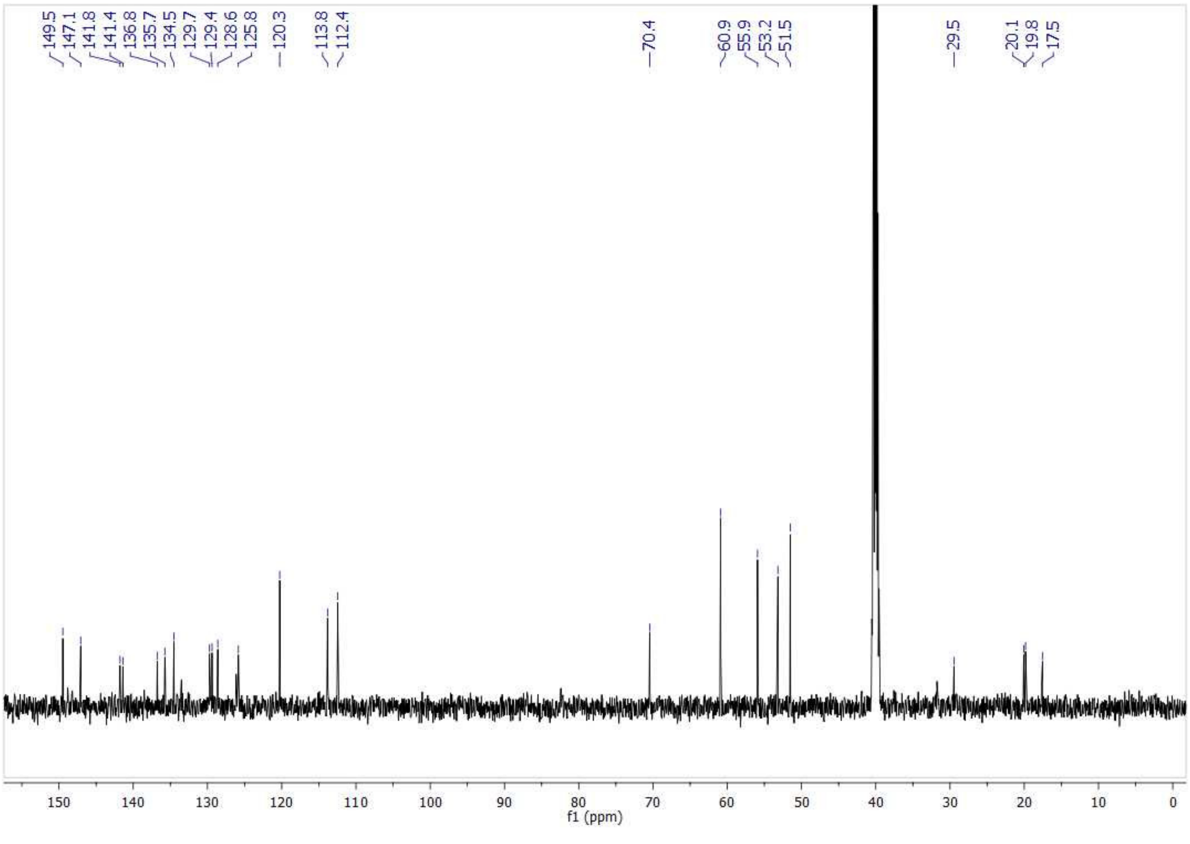

7e

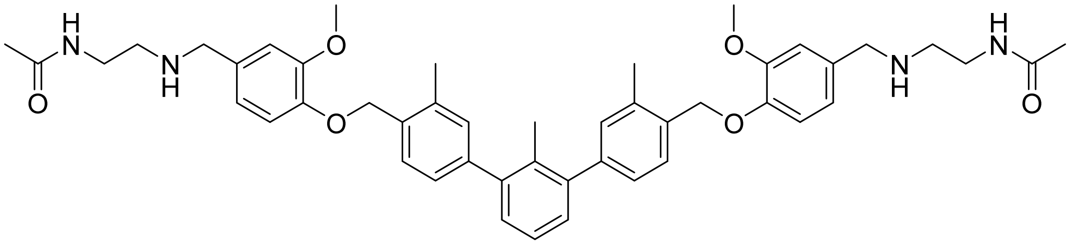

H
N
O
N
H
O
O
O
O
N
H
H
N
O

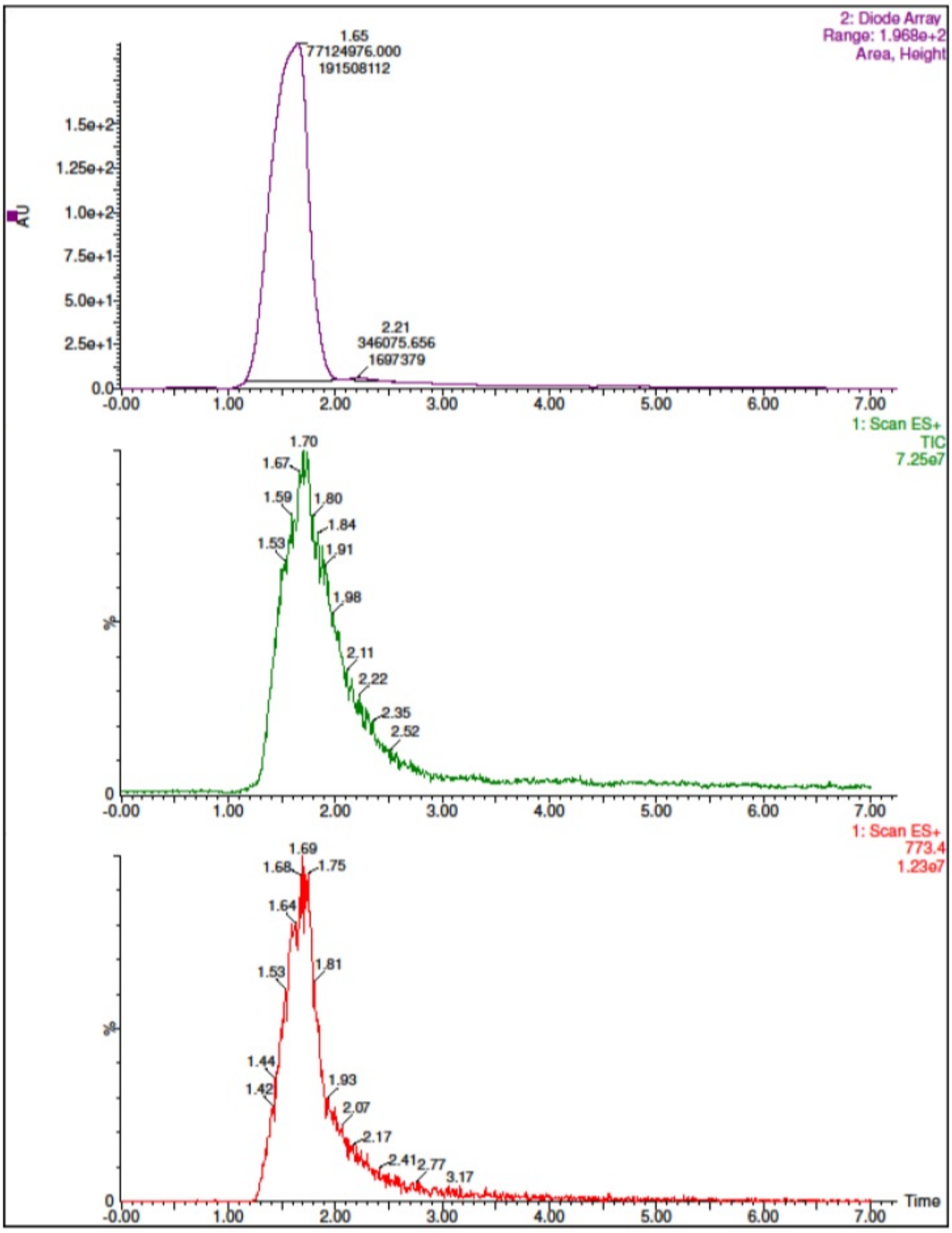

2: Diode Array
Range: 1.968e+2
Area, Height
1.65
77124976.000
191508112
2.21
346075.656
1697379
AU
1.5e+2
1.25e+2
1.0e+2
7.5e+1
5.0e+1
2.5e+1
0.0
1: Scan ES+
TIC
7.25e7
1.70
1.67
1.59
1.80
1.84
1.53
1.91
1.98
2.11
2.22
2.35
2.52
%
1: Scan ES+
773.4
1.23e7
1.69
1.68
1.75
1.64
1.53
1.81
1.44
1.42
1.93
2.07
2.17
2.41
2.77
3.17
Time
-0.00
1.00
2.00
3.00
4.00
5.00
6.00
7.00

^{1}H NMR *(600 MHz, $CDCl_3$)*

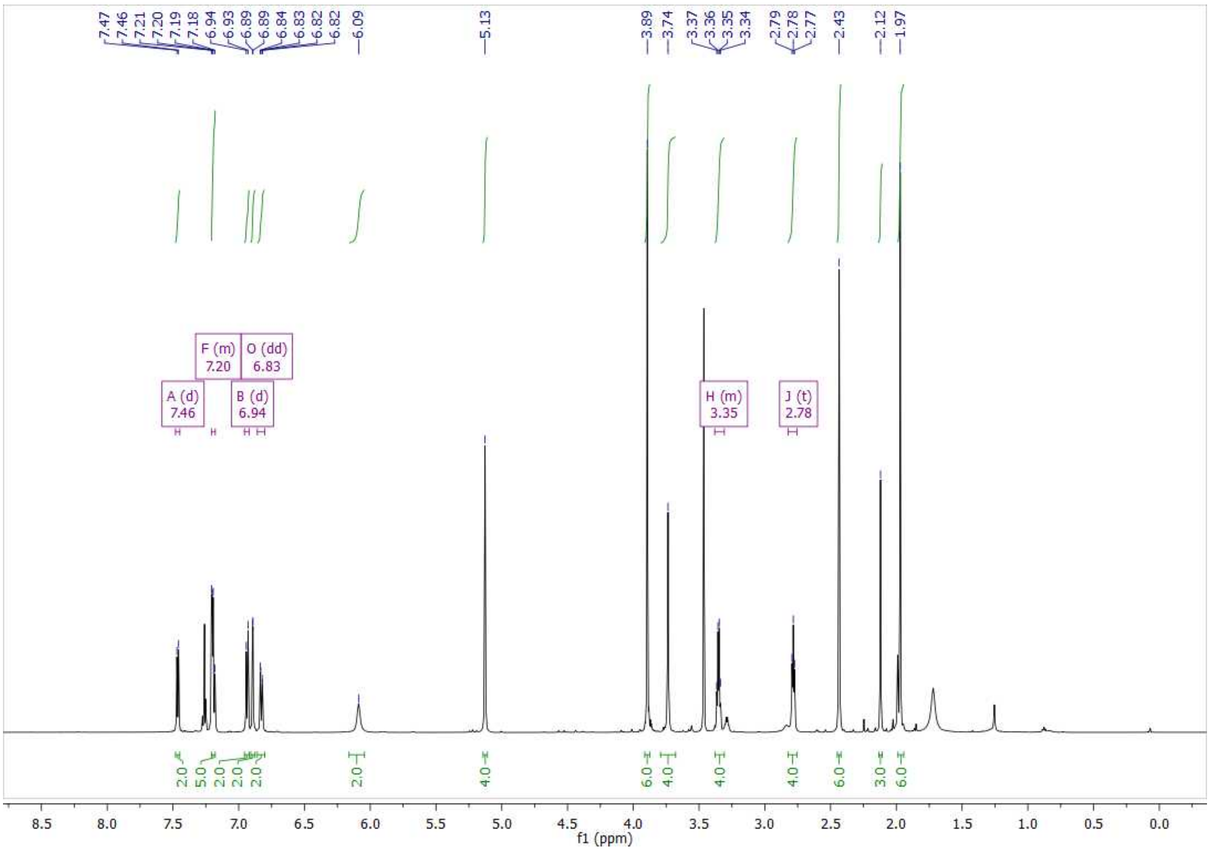

^{13}C NMR *(151 MHz, $CDCl_3$)*

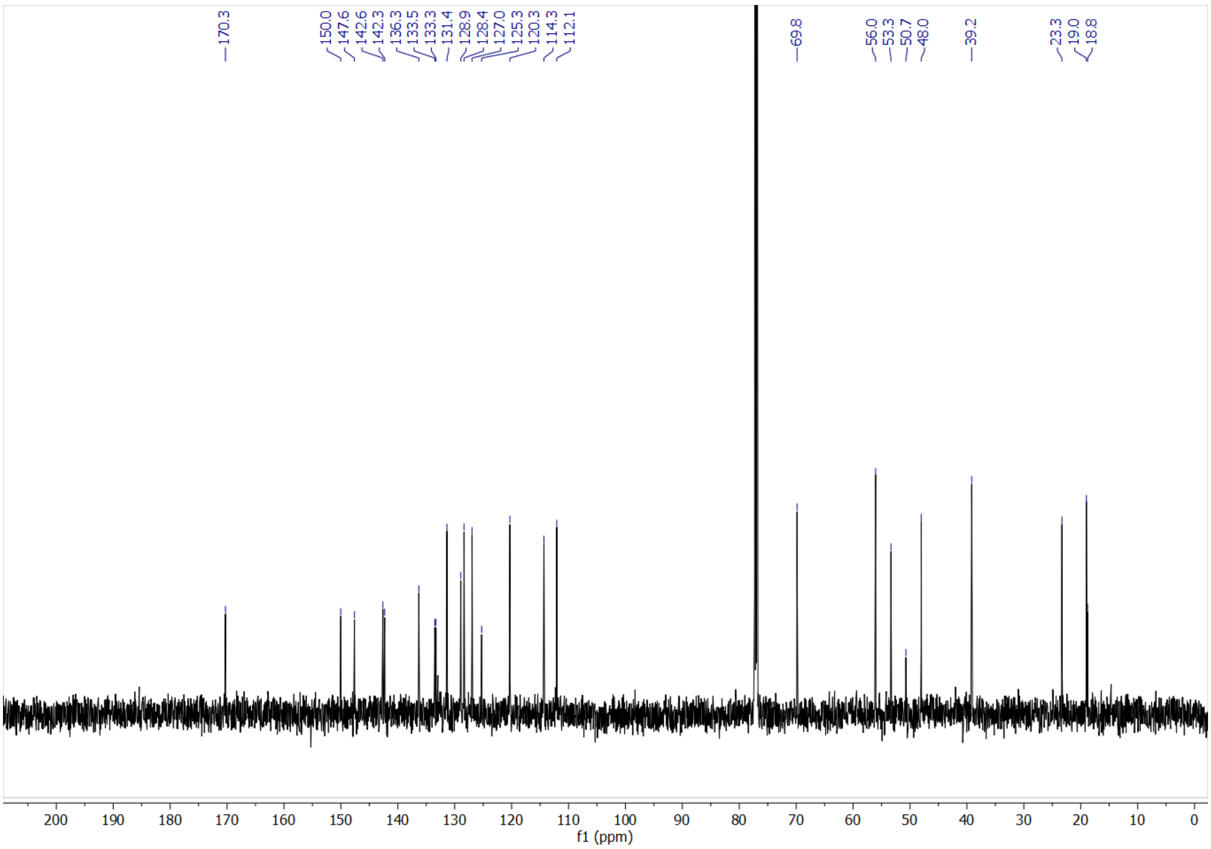

7f

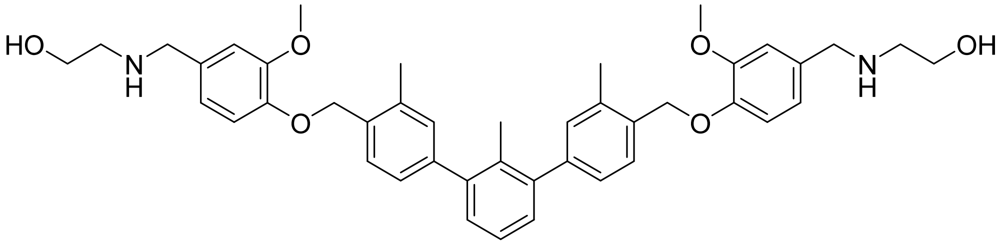
HO
N
H
O
O
O
O
N
H
OH

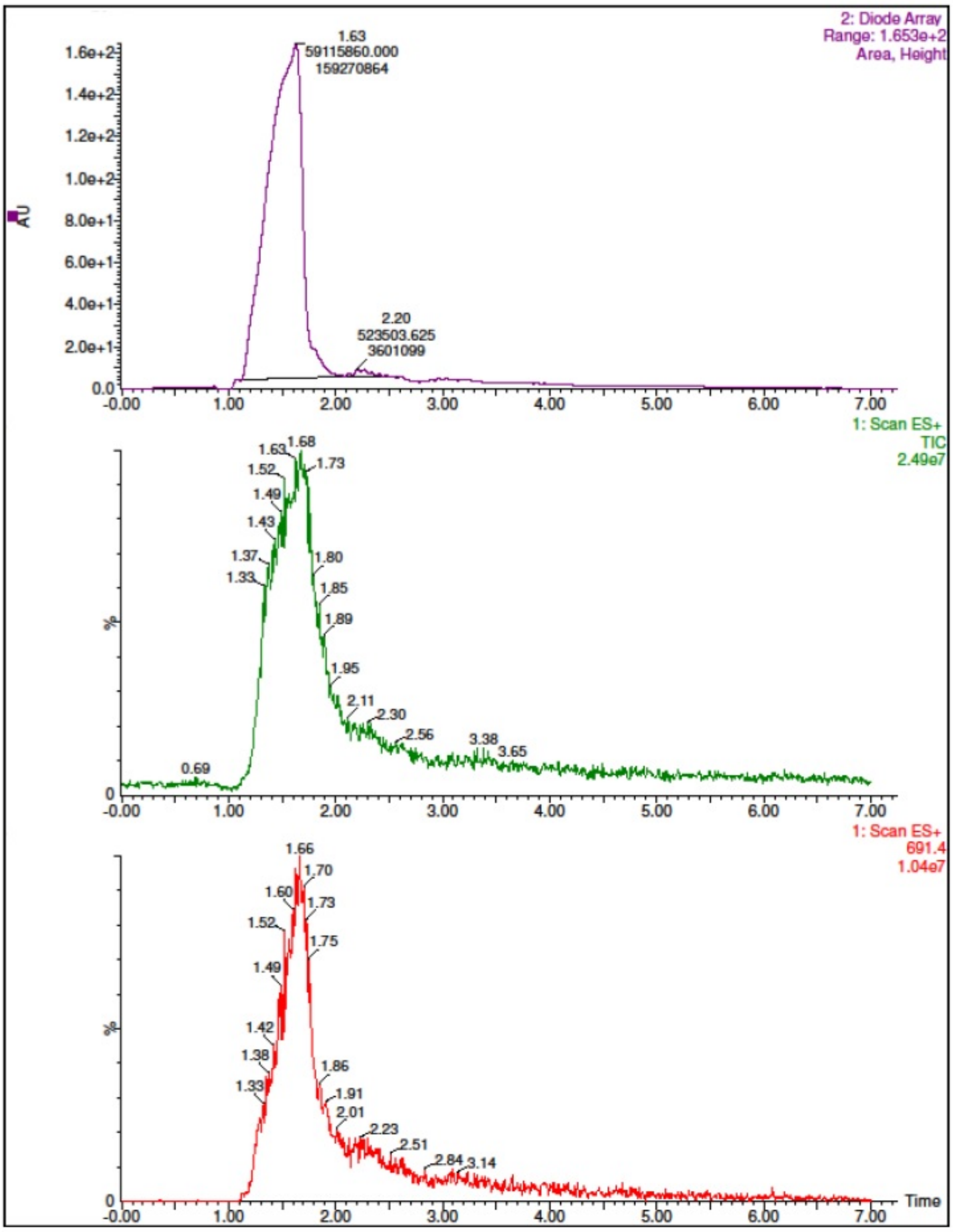
2: Diode Array
Range: 1.653e+2
Area, Height
1.63
59115860.000
159270864
2.20
523503.625
3601099
AU
1: Scan ES+
TIC
2.49e7
1: Scan ES+
691.4
1.04e7
%
Time

***^{1}H NMR** (600 MHz, $CDCl_3$)*

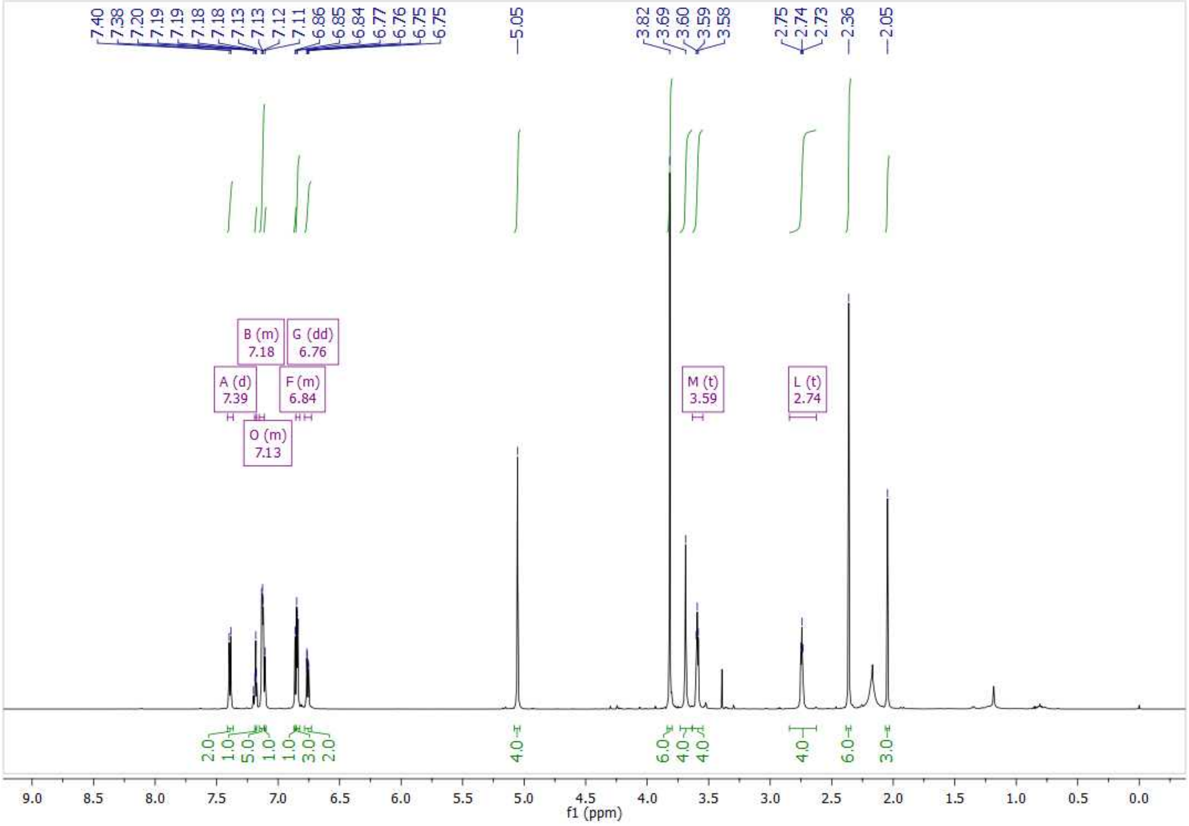

***^{13}C NMR** (151 MHz, $CDCl_3$)*

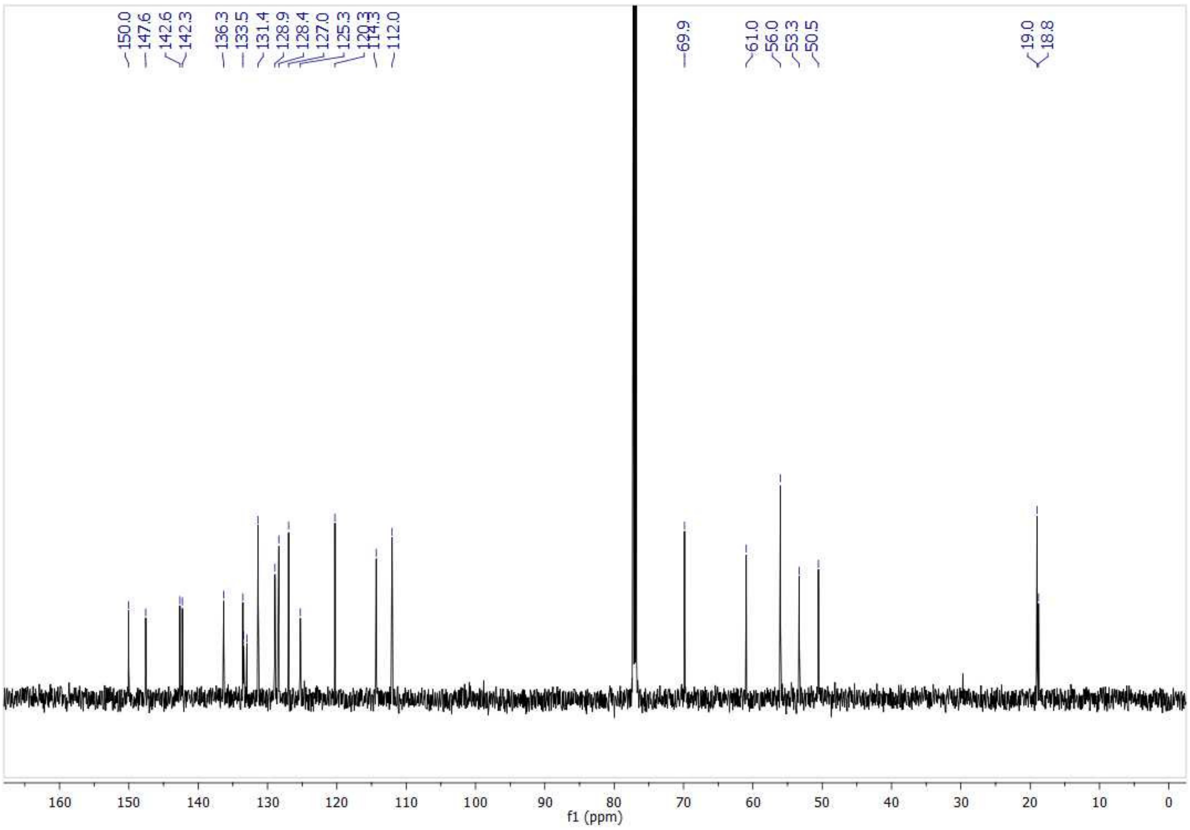

7g

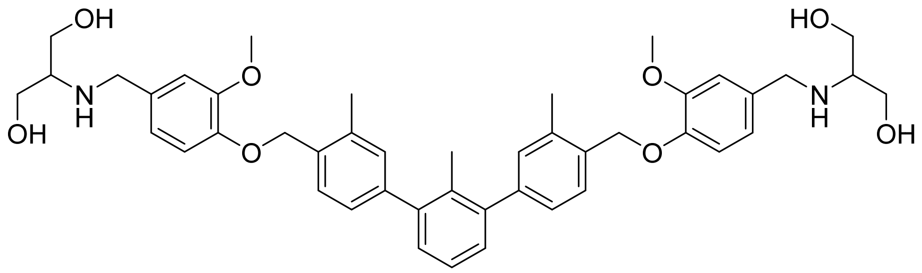

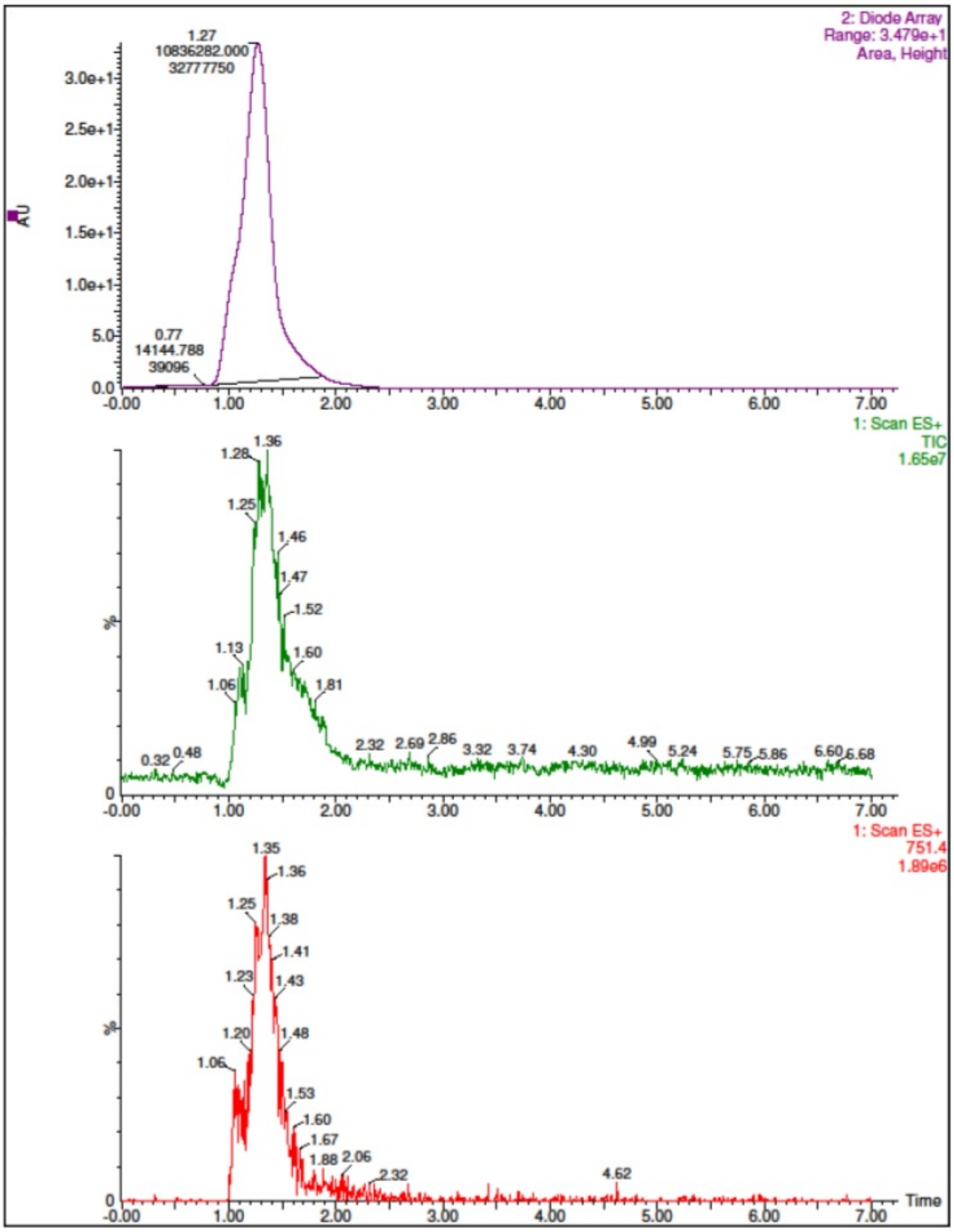
2: Diode Array
Range: 3.479e+1
Area, Height
1.27
10836282.000
32777750
0.77
14144.788
39096
AU
1: Scan ES+
TIC
1.65e7
%
1: Scan ES+
751.4
1.89e6
Time

***^{1}H NMR** (600 MHz, DMF-d7)*

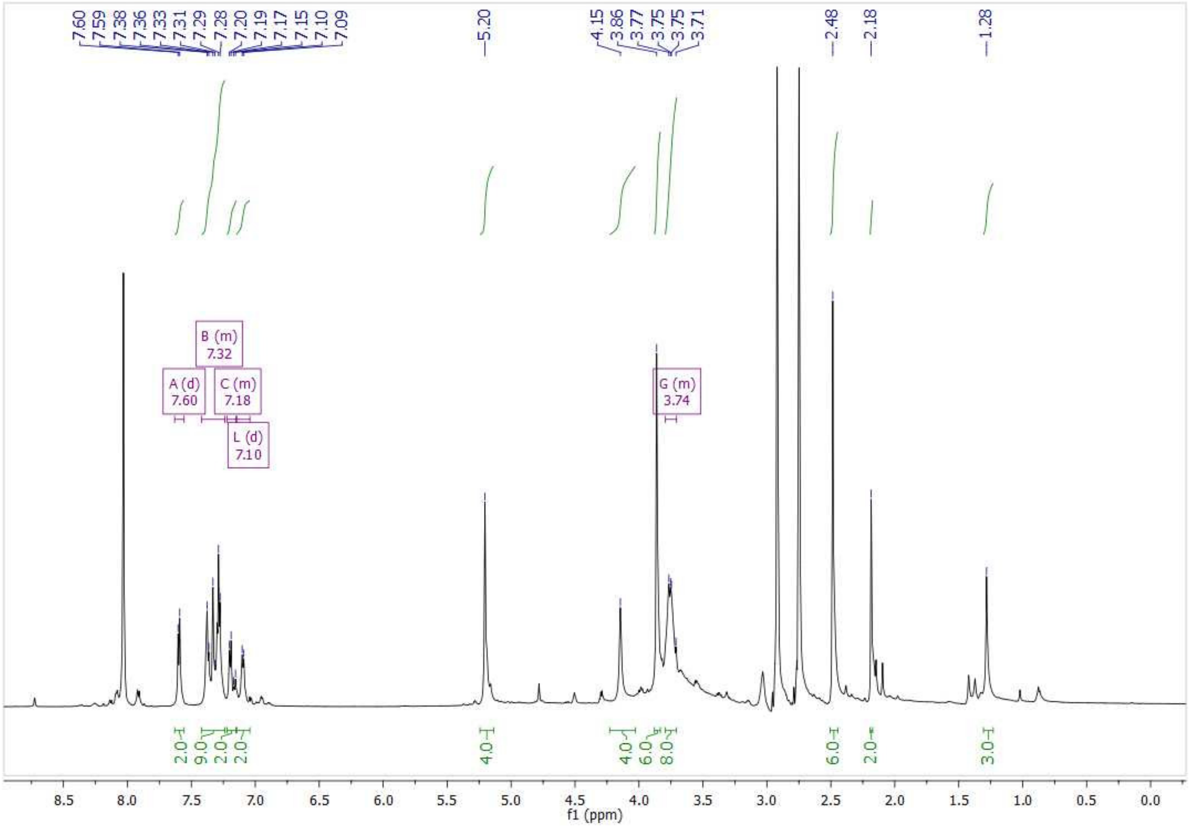

***^{13}C NMR** (151 MHz, $CDCl_3$)*

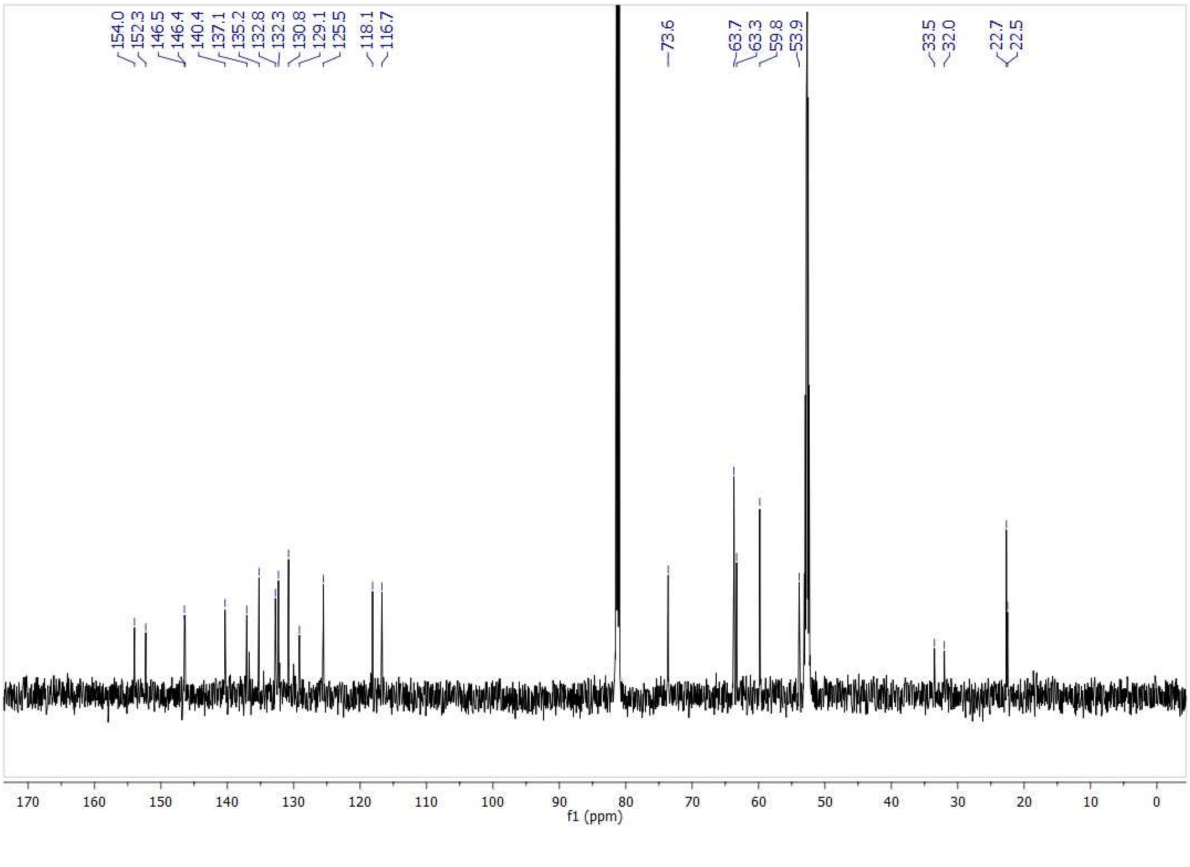

7h

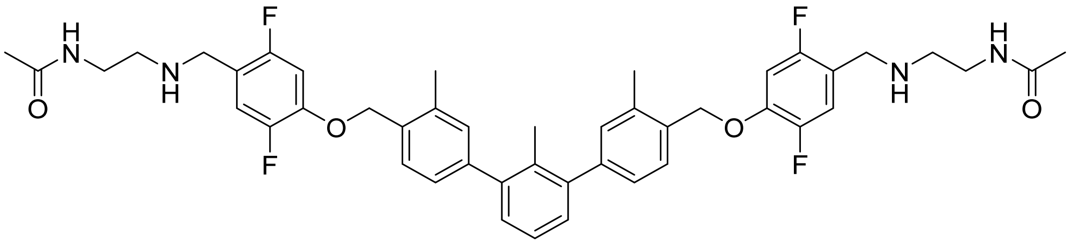

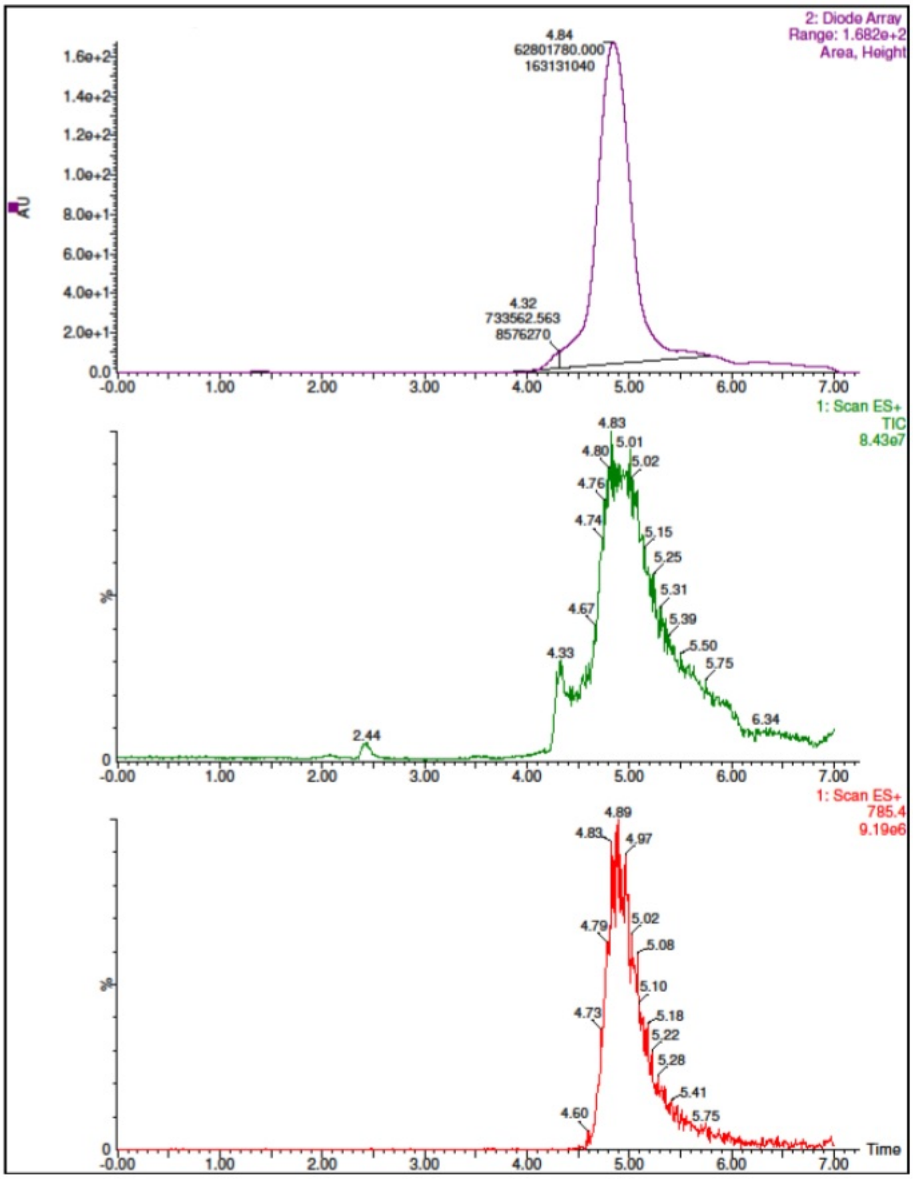

2: Diode Array
Range: 1.682e+2
Area, Height
4.84
62801780.000
163131040
4.32
733562.563
8576270
AU
1: Scan ES+
TIC
8.43e7
1: Scan ES+
785.4
9.19e6
Time

***^{1}H NMR** (600 MHz, $CDCl_3$)*

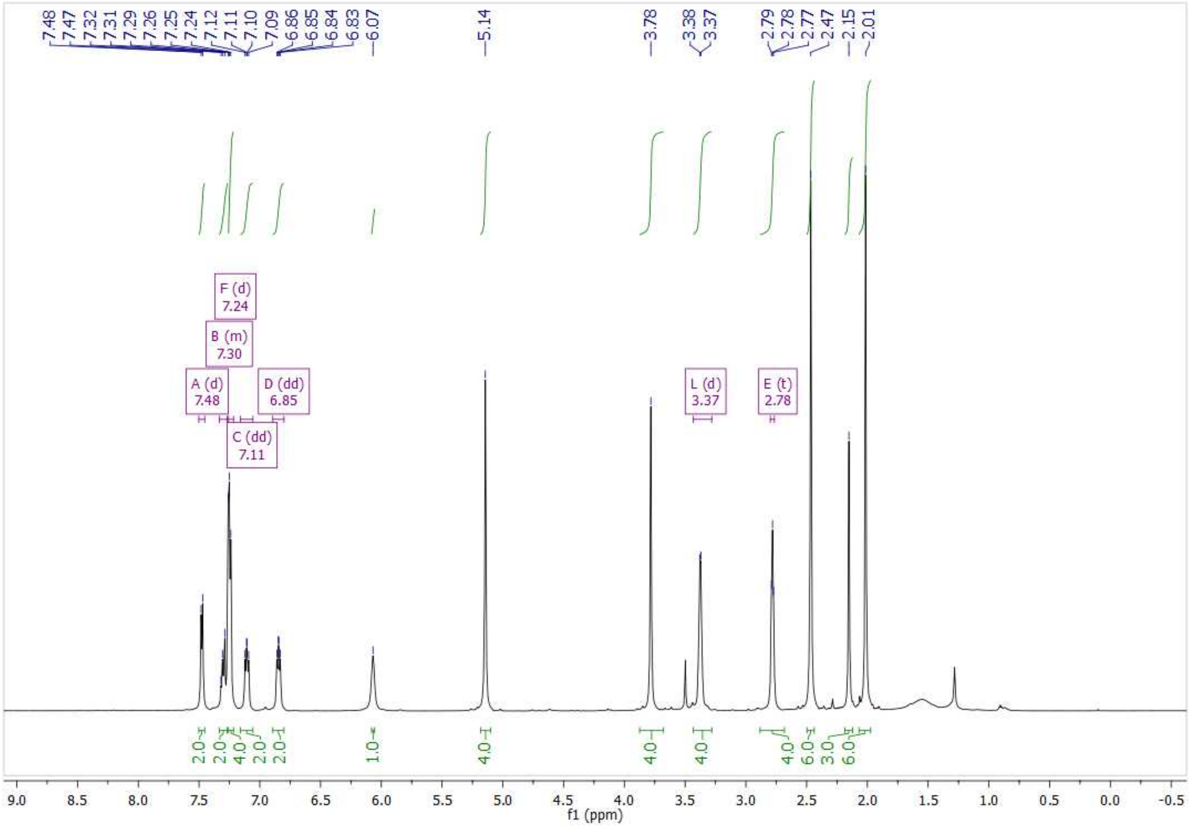

***^{13}C NMR** (151 MHz, $CDCl_3$)*

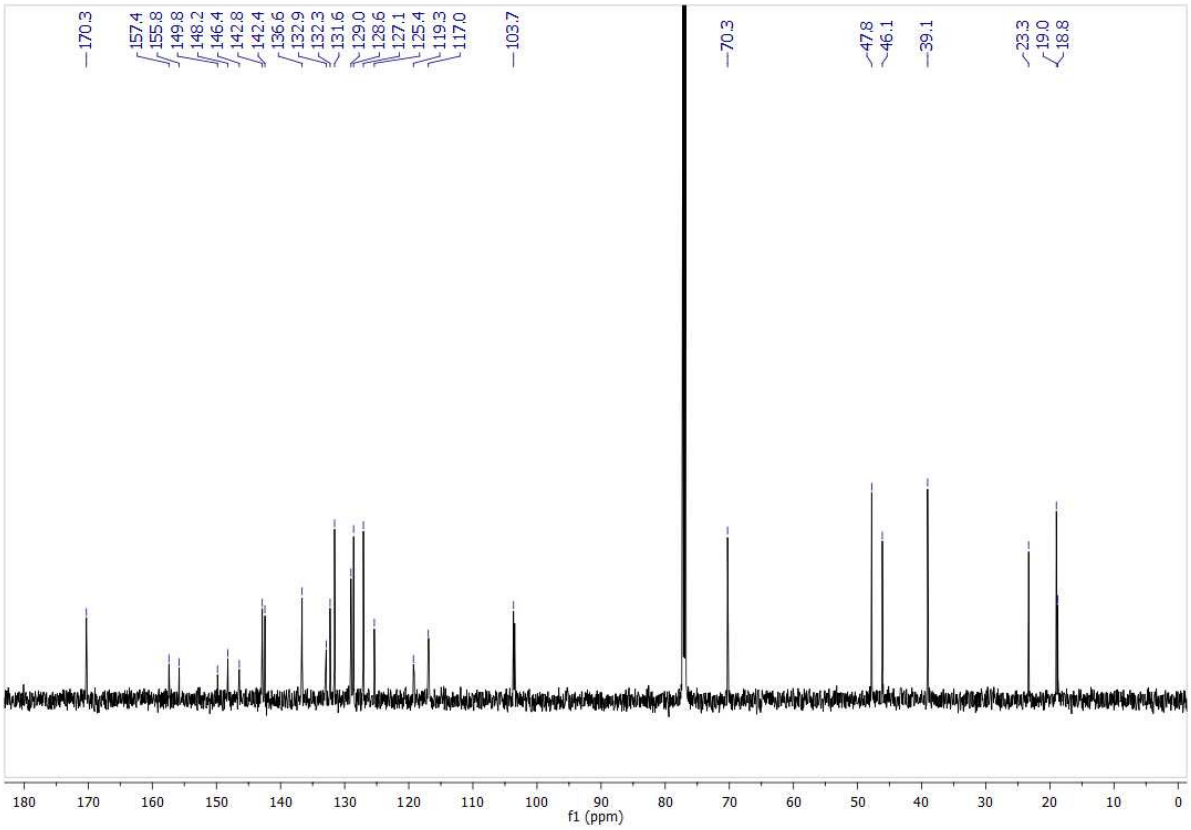

7i

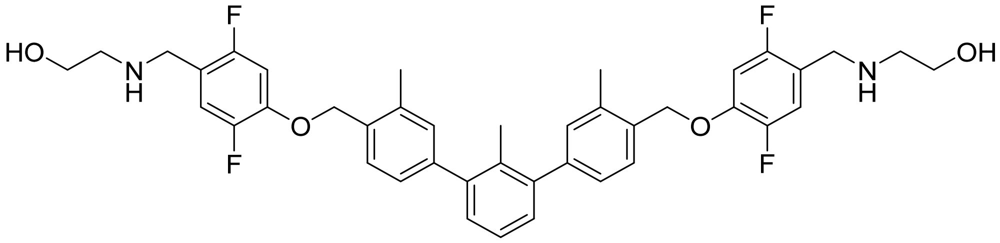

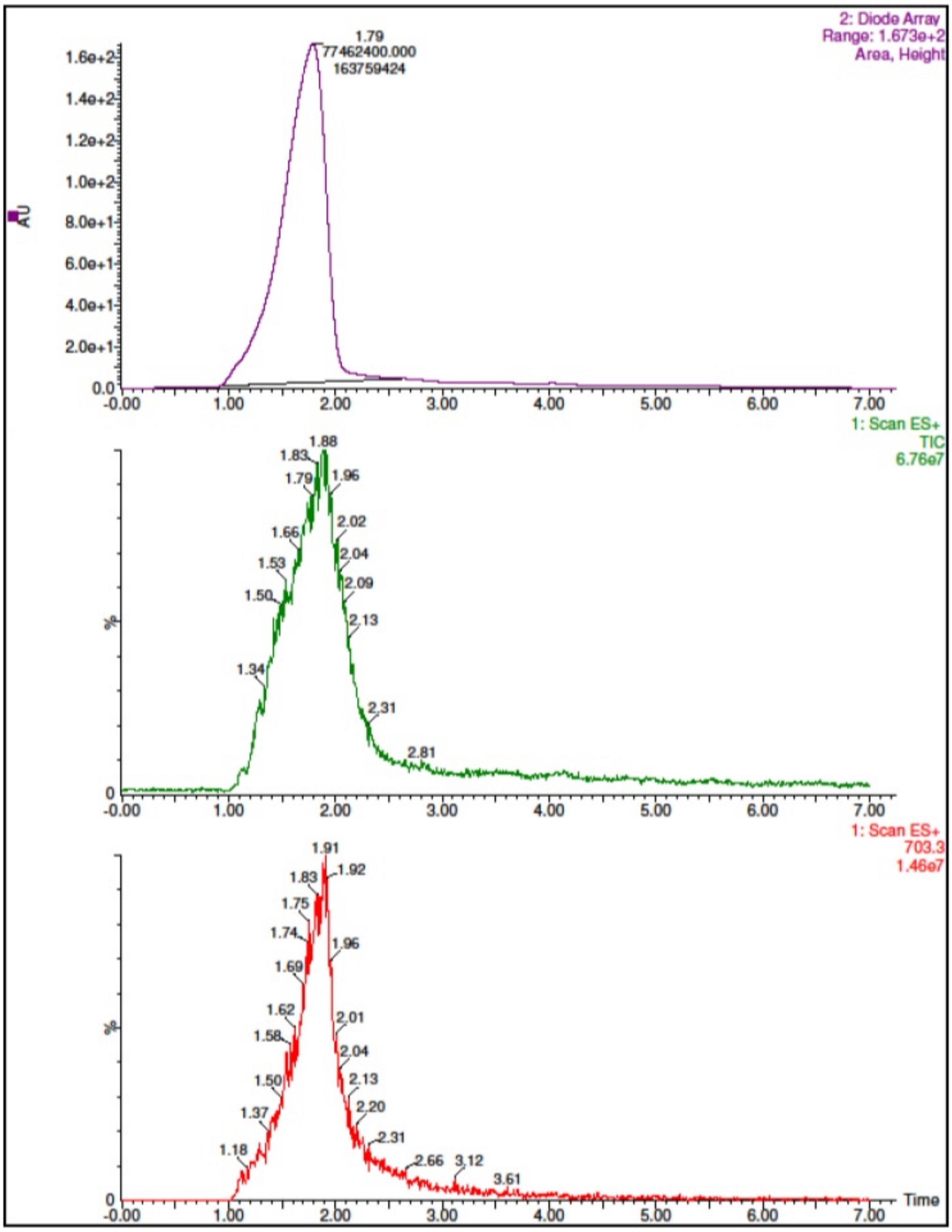
2: Diode Array
Range: 1.673e+2
Area, Height
1.79
77462400.000
163759424
AU
1.6e+2
1.4e+2
1.2e+2
1.0e+2
8.0e+1
6.0e+1
4.0e+1
2.0e+1
0.0
-0.00 1.00 2.00 3.00 4.00 5.00 6.00 7.00
1: Scan ES+
TIC
6.76e7
1.88
1.83
1.79
1.96
1.66
2.02
1.53
2.04
1.50
2.09
2.13
1.34
2.31
2.81
%
0
-0.00 1.00 2.00 3.00 4.00 5.00 6.00 7.00
1: Scan ES+
703.3
1.46e7
1.91
1.83
1.92
1.75
1.74
1.96
1.69
1.62
2.01
1.58
2.04
1.50
2.13
1.37
2.20
1.18
2.31
2.66
3.12
3.61
%
0
-0.00 1.00 2.00 3.00 4.00 5.00 6.00 7.00
Time

^{1}H NMR *(600 MHz, CDCl₃)*

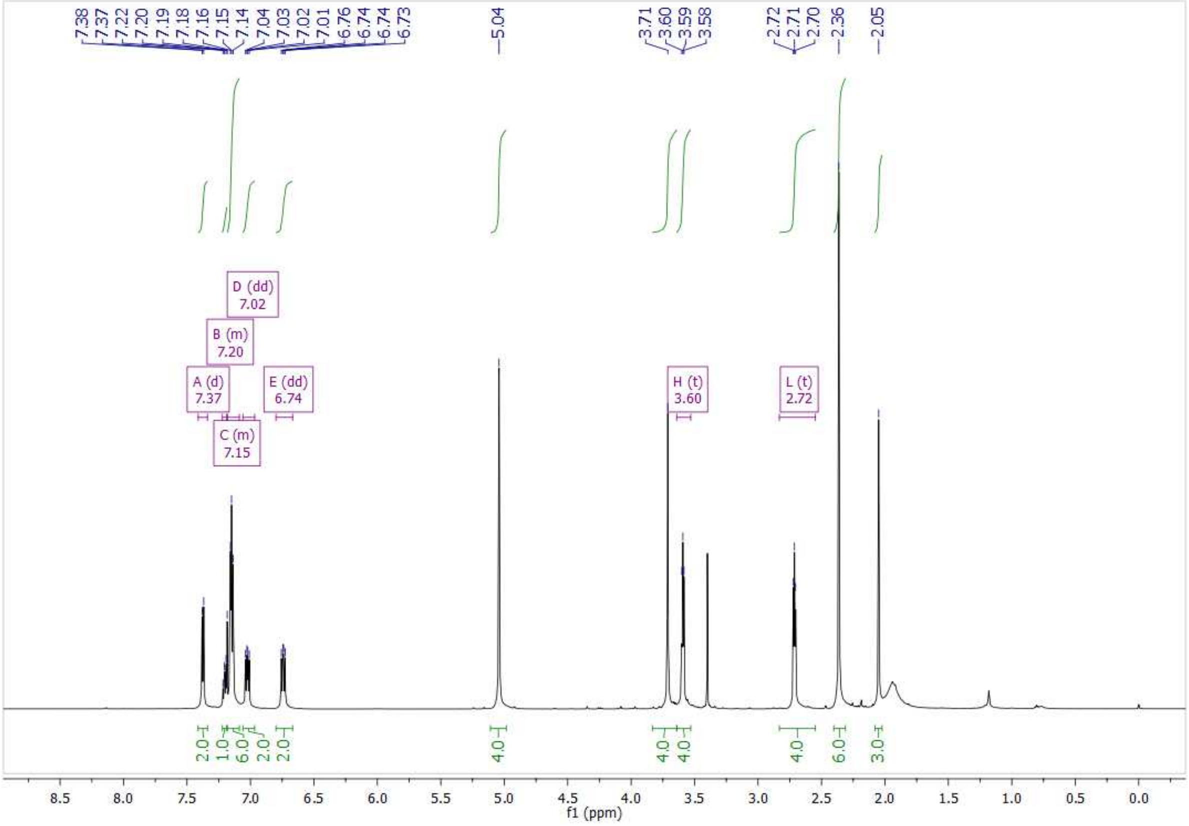

^{13}C NMR *(151 MHz, CDCl₃)*

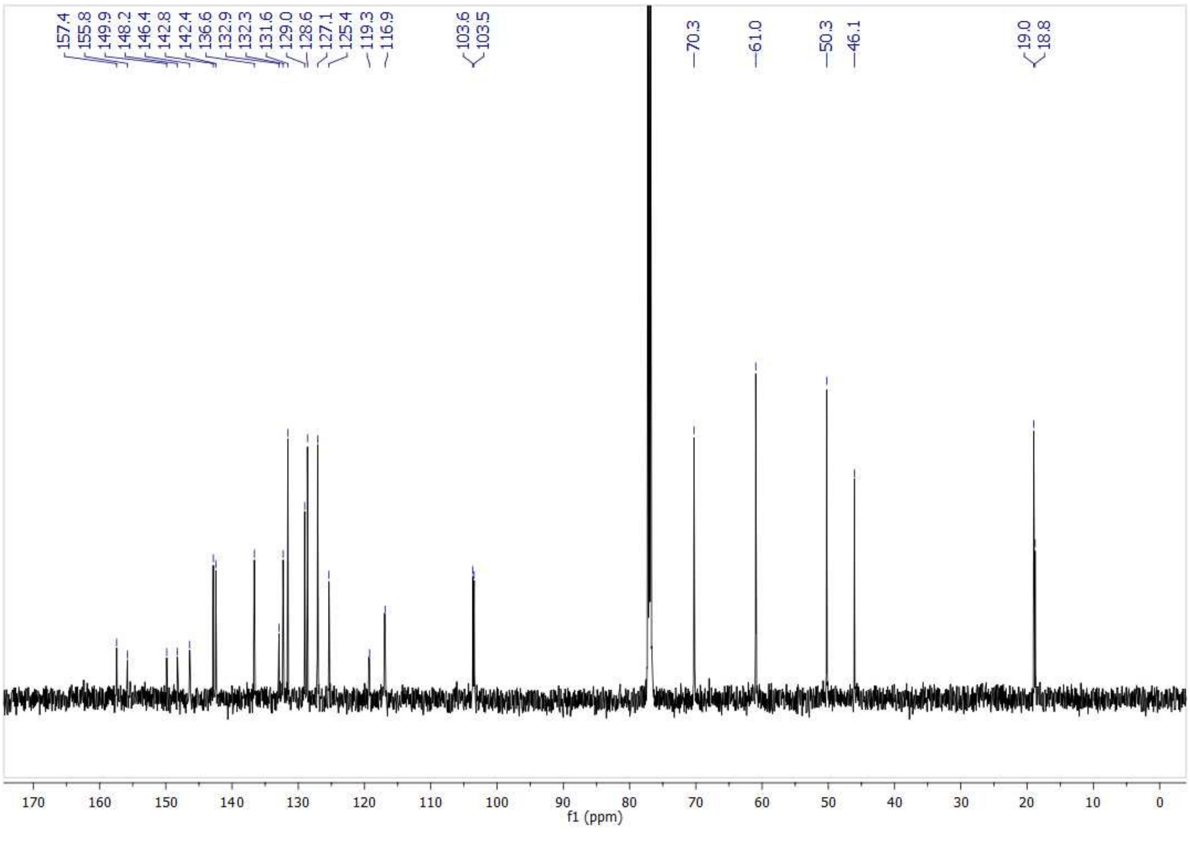

7j

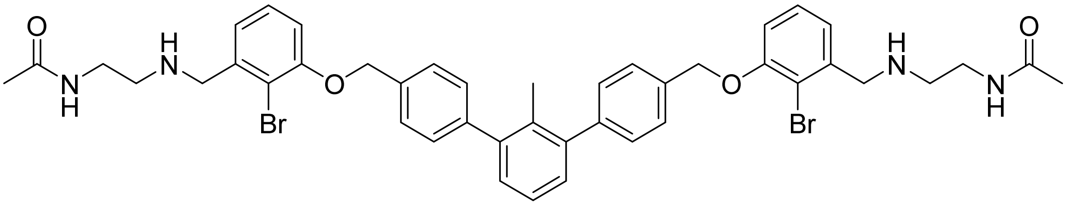
O
H
N
N
H
Br
O
O
Br
H
N
N
H
O

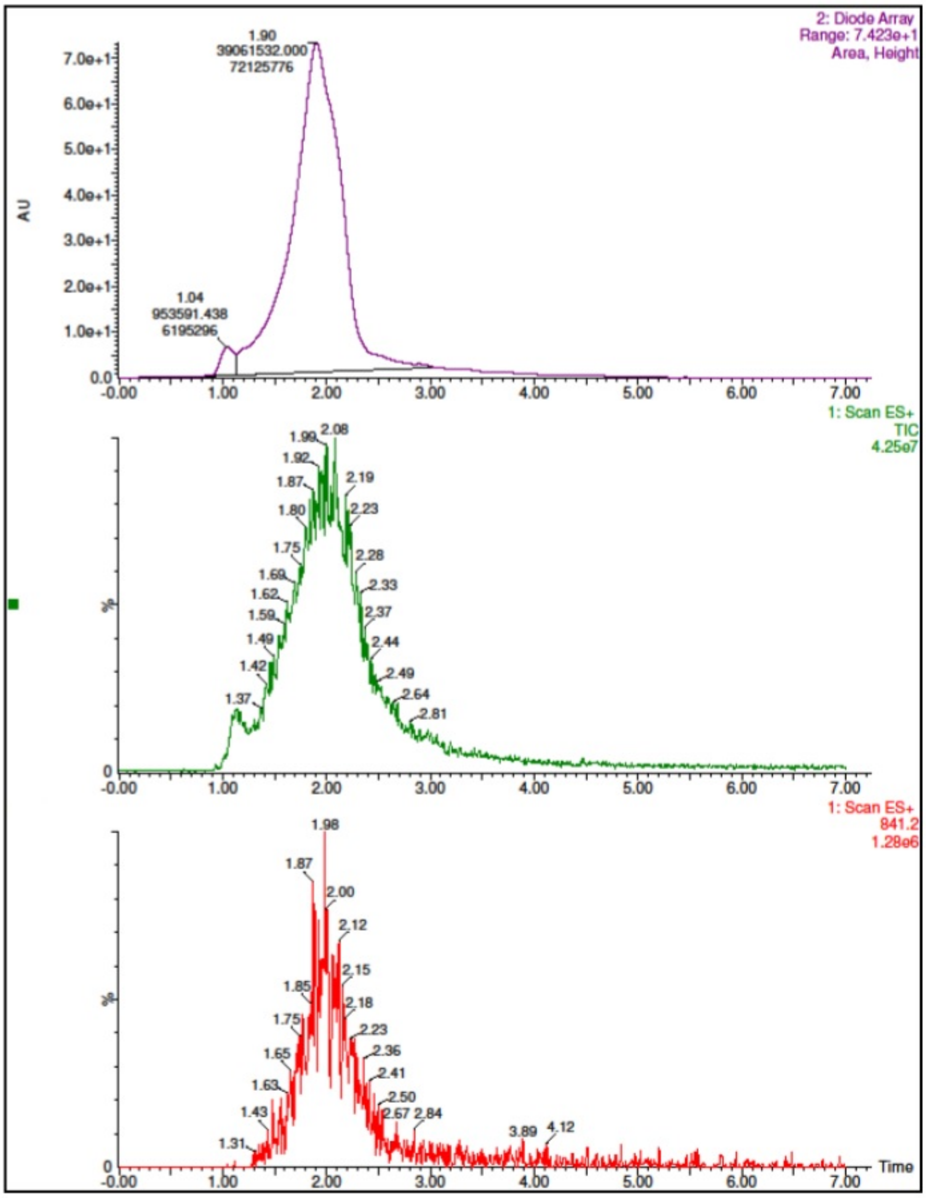
2: Diode Array
Range: 7.423e+1
Area, Height
1.90
39061532.000
72125776
1.04
953591.438
6195296
AU
1: Scan ES+
TIC
4.25e7
1: Scan ES+
841.2
1.28e6
%
Time

^{1}H NMR *(600 MHz, DMSO-d₆)*

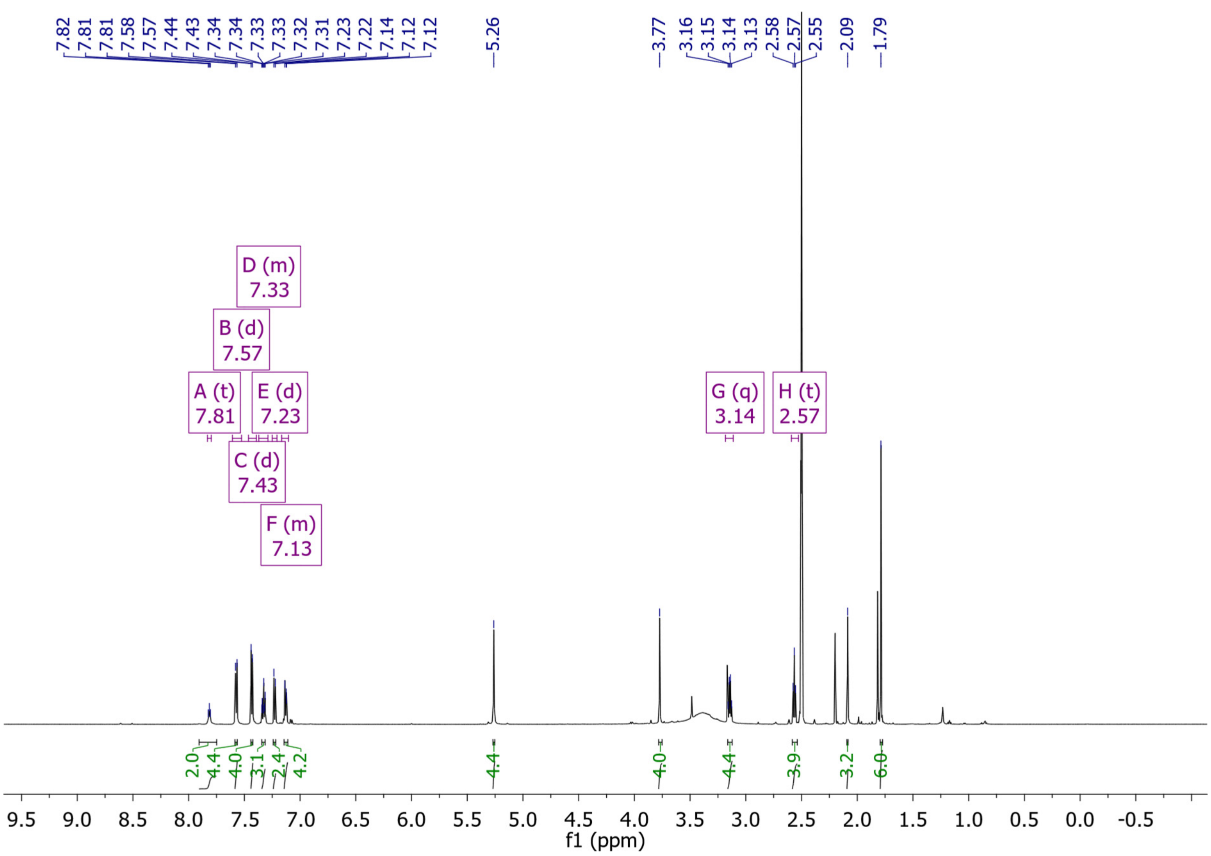

^{13}C NMR *(151 MHz, DMSO-d₆)*

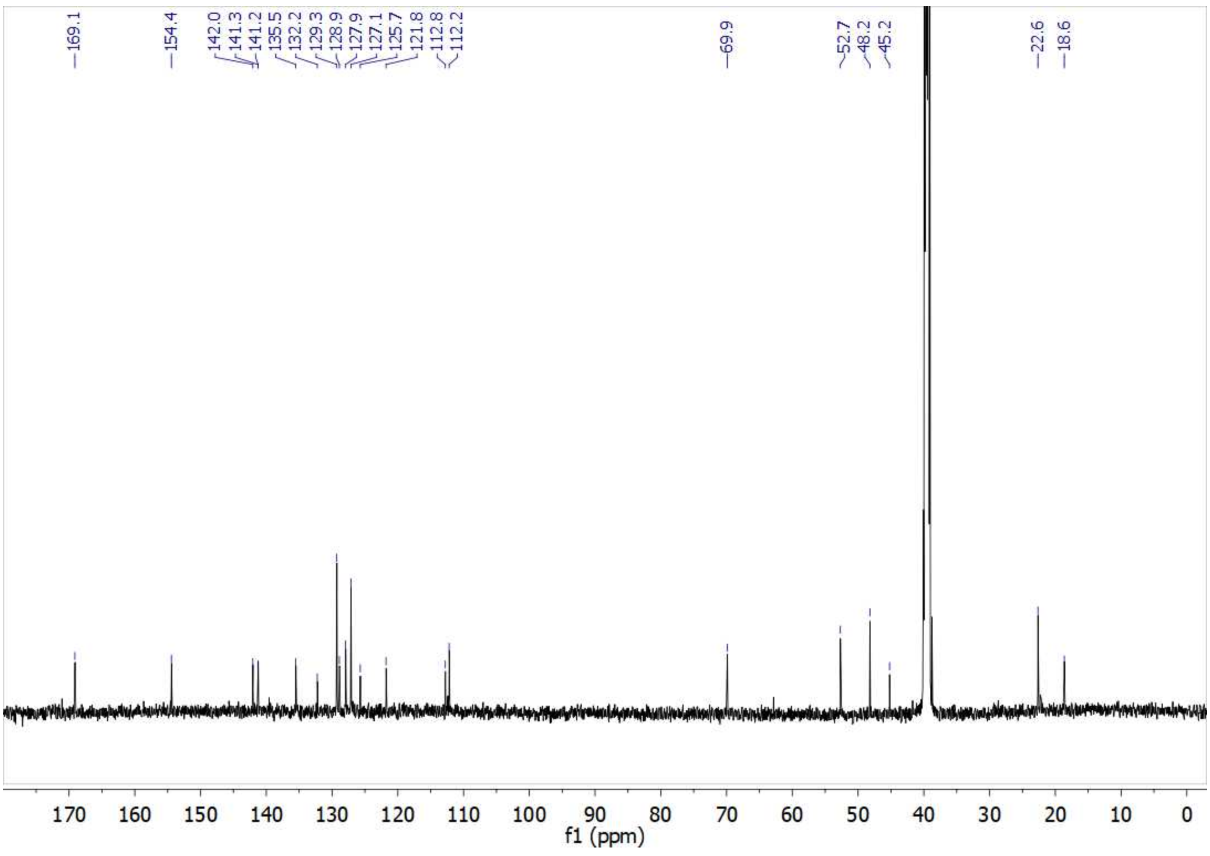

7k

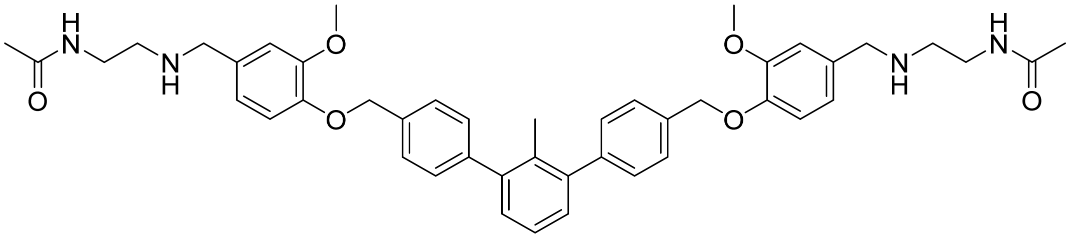

H
N
O
N
H
O
O
O
O
H
N
N
H
O

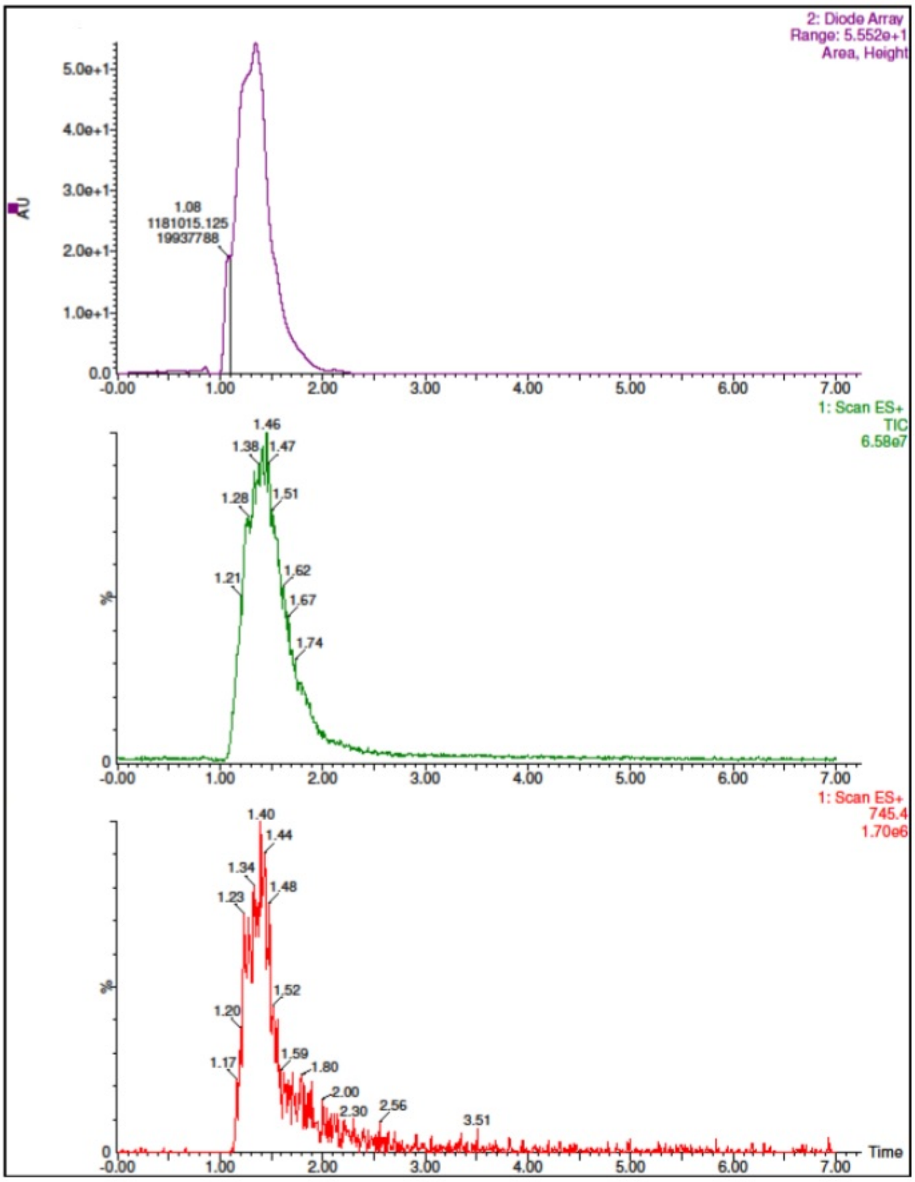

2: Diode Array
Range: 5.552e+1
Area, Height
AU
1.08
1181015.125
19937788
1: Scan ES+
TIC
6.58e7
1: Scan ES+
745.4
1.70e6
Time

¹H NMR *(600 MHz, DMSO-d₆)*

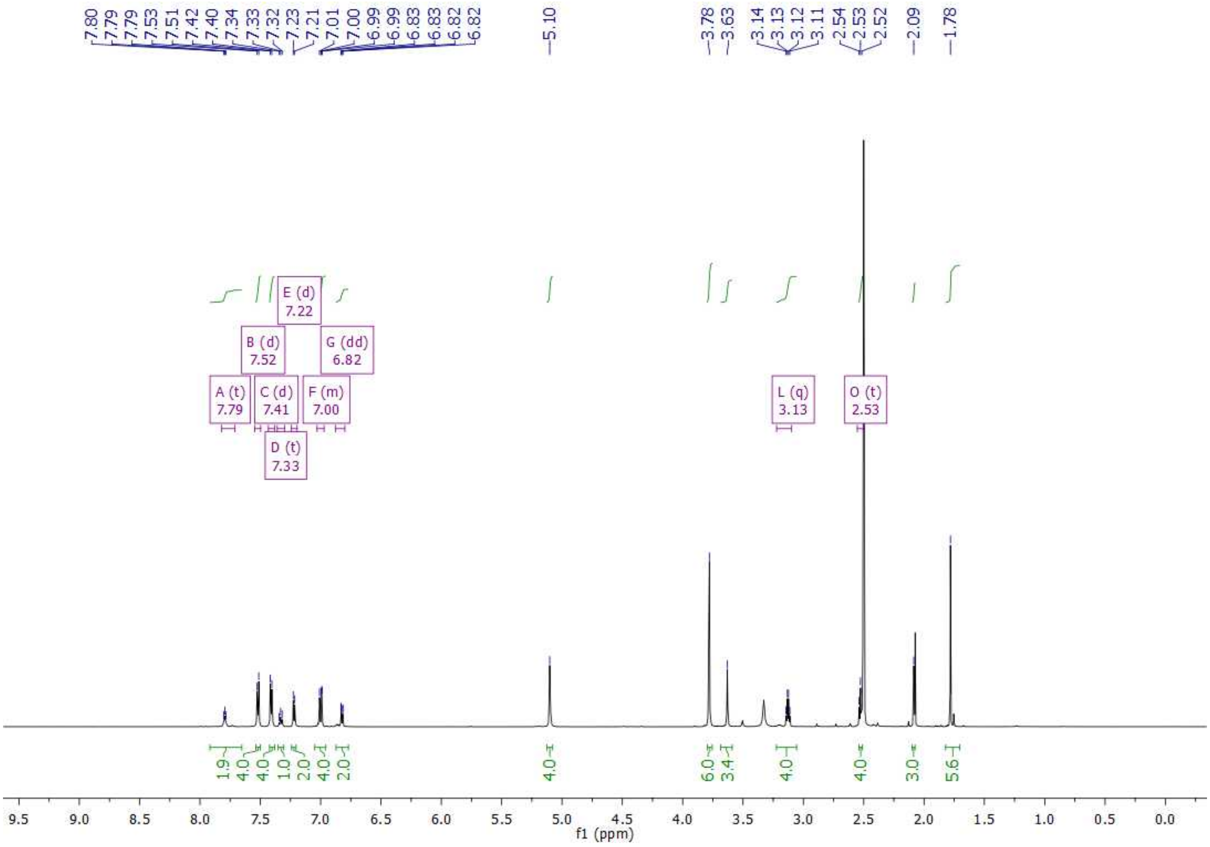

¹³C NMR *(151 MHz, DMSO-d₆)*

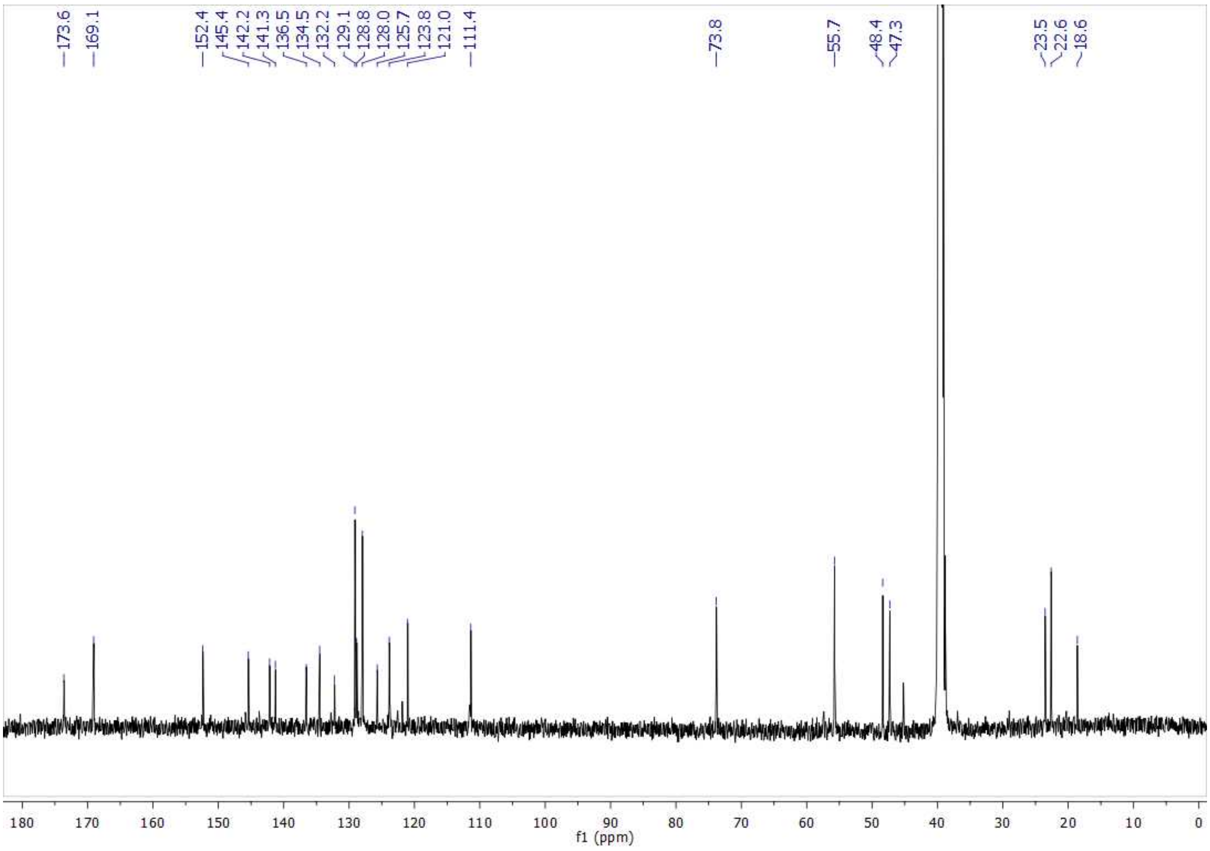

7l

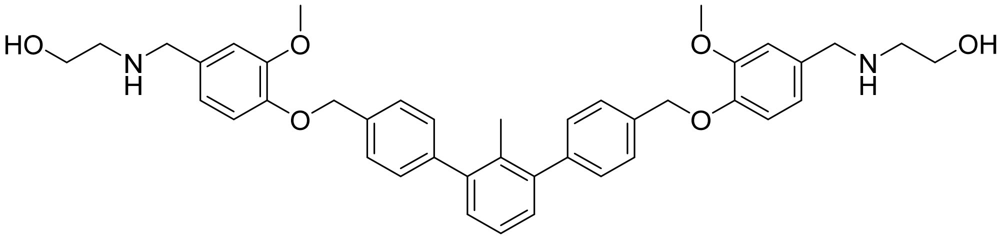

HO
N
H
O
O
O
O
N
H
OH

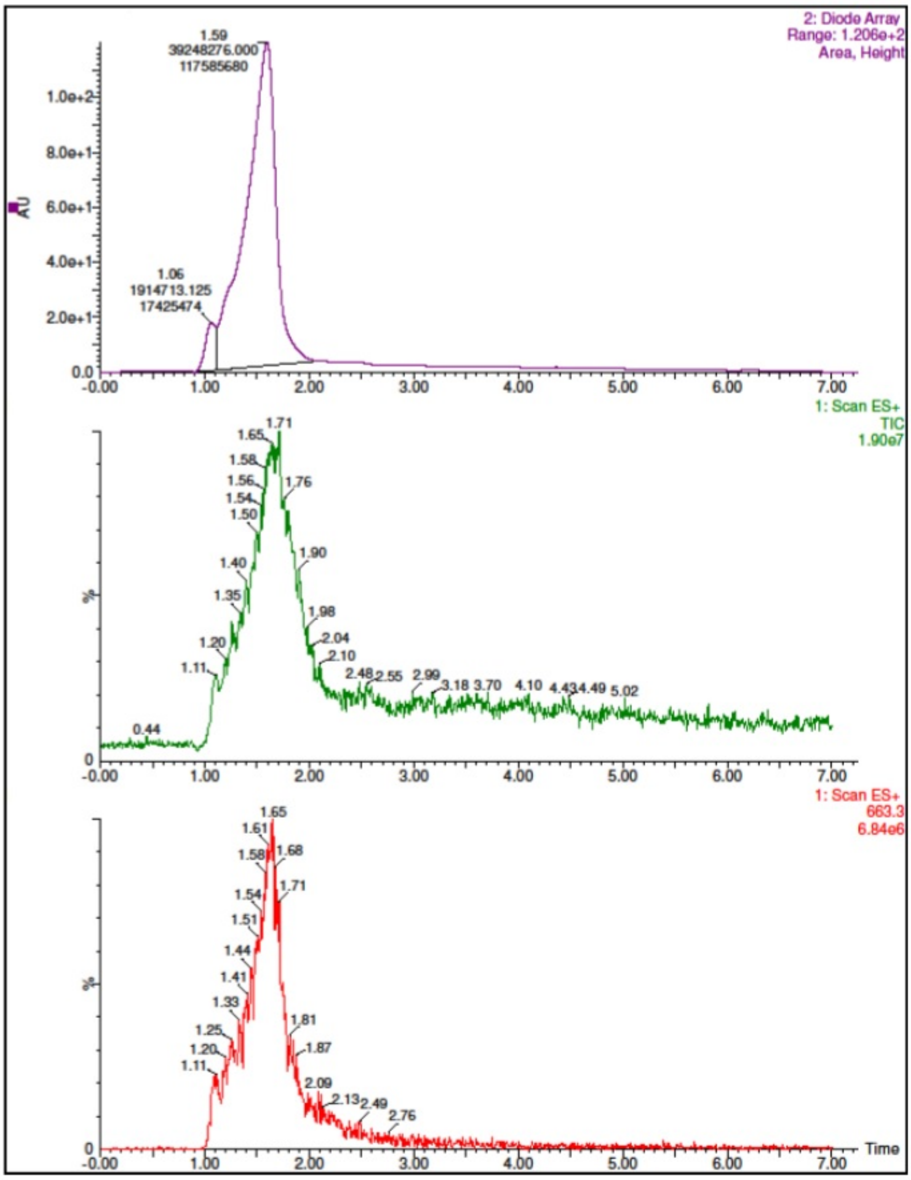

2: Diode Array
Range: 1.206e+2
Area, Height
1.59
39248276.000
117585680
1.06
1914713.125
17425474
AU
1: Scan ES+
TIC
1.90e7
1: Scan ES+
663.3
6.84e6
Time

***^{1}H NMR** (600 MHz, DMSO-d6)*

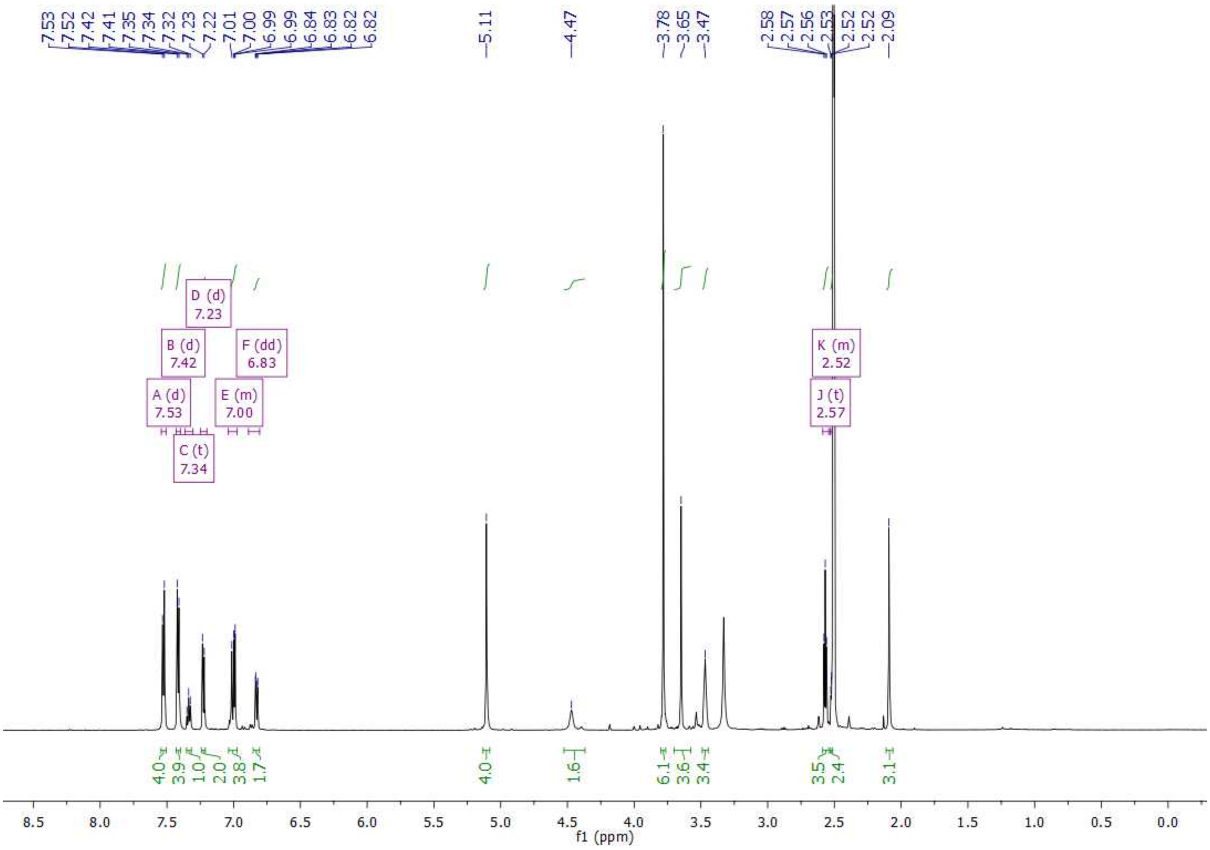

***^{13}C NMR** (151 MHz, DMSO-d6)*

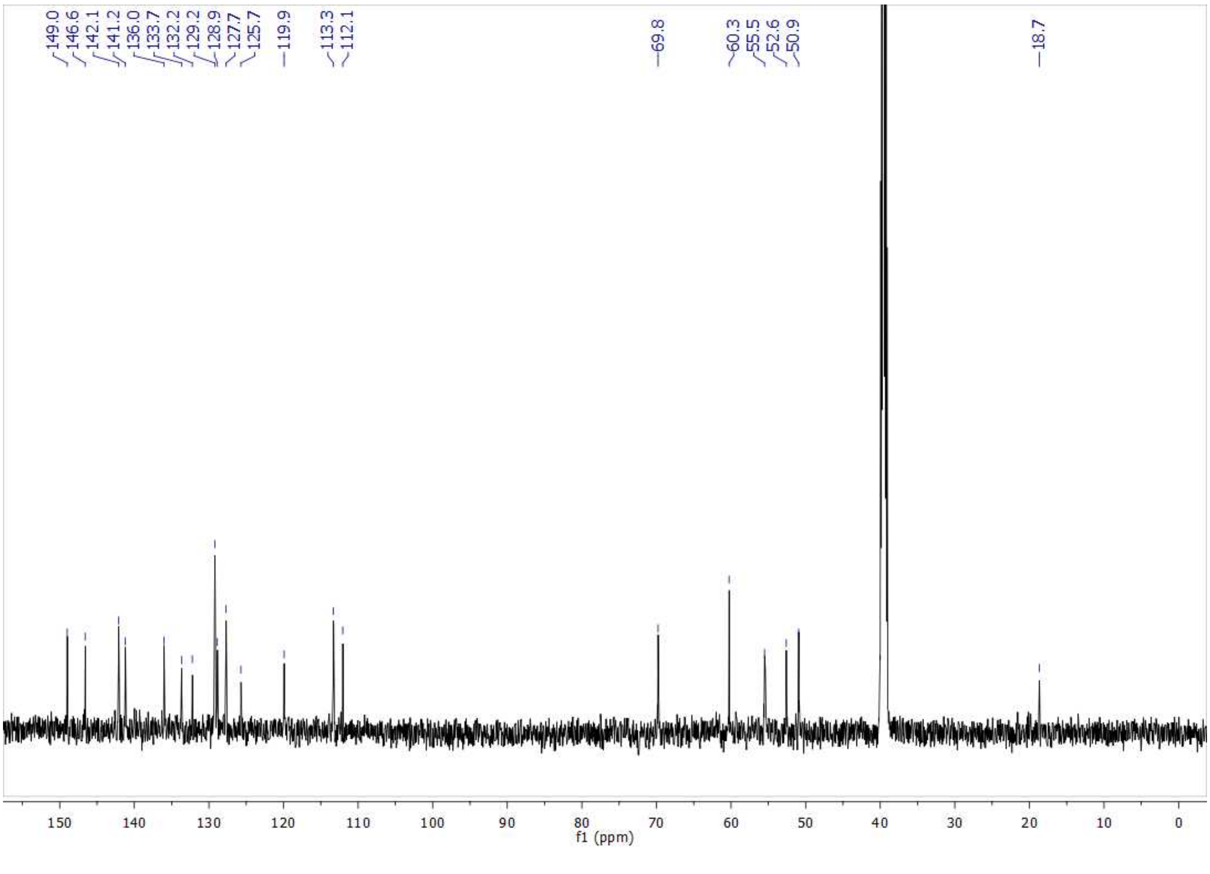

7m

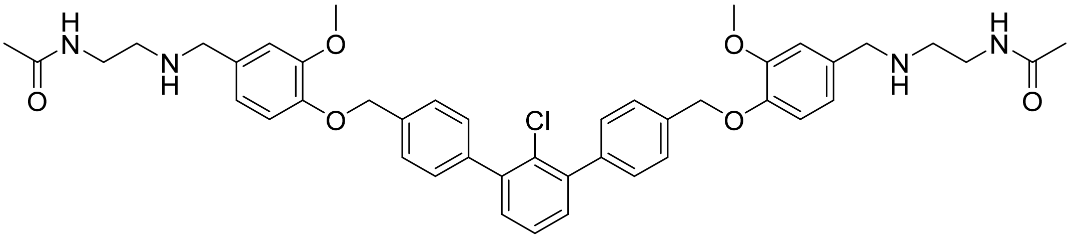
H
N
O
N
H
O
O
Cl
O
O
N
H
H
N
O

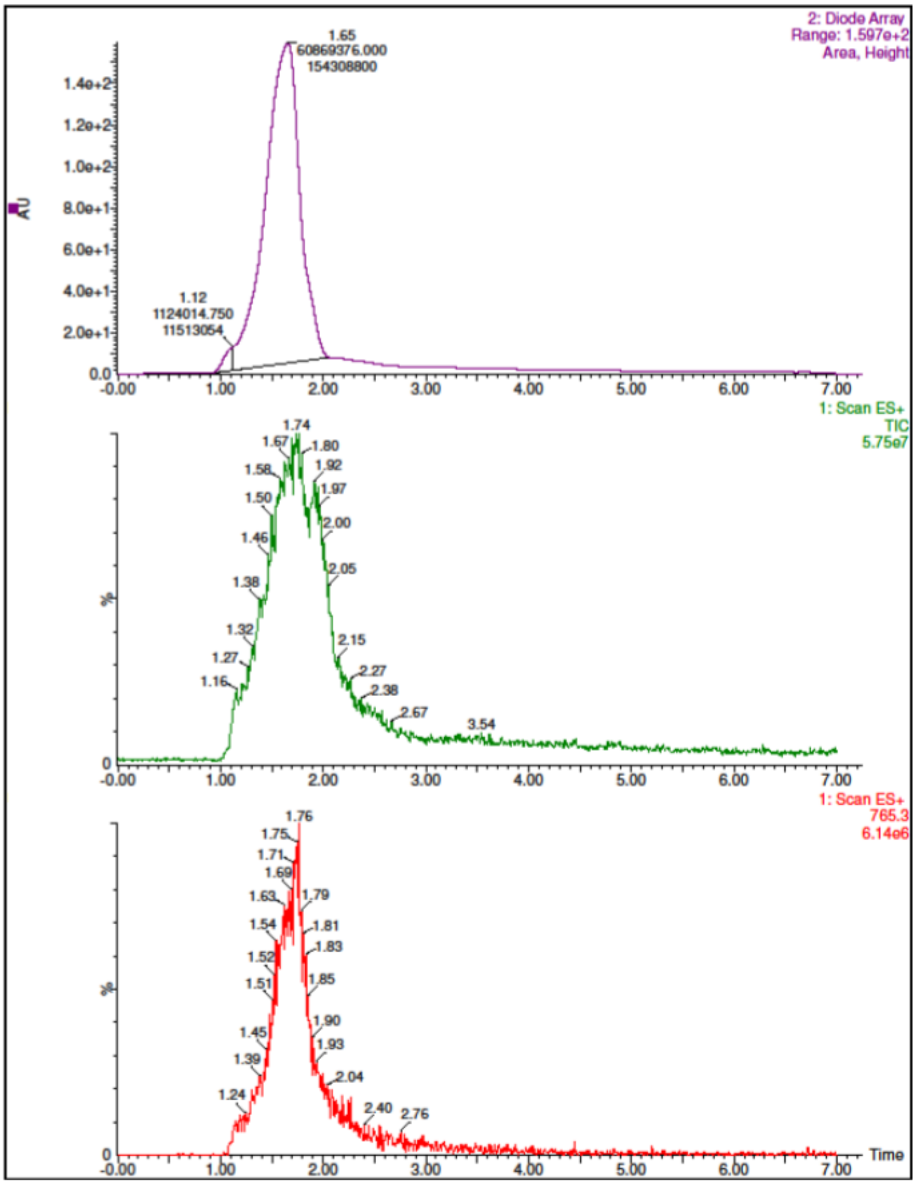
2: Diode Array
Range: 1.597e+2
Area, Height
AU
1.65
60869376.000
154308800
1.12
1124014.750
11513054
1: Scan ES+
TIC
5.75e7
%
1: Scan ES+
765.3
6.14e6
%
Time

^{1}H NMR *(600 MHz, DMSO-d6)*

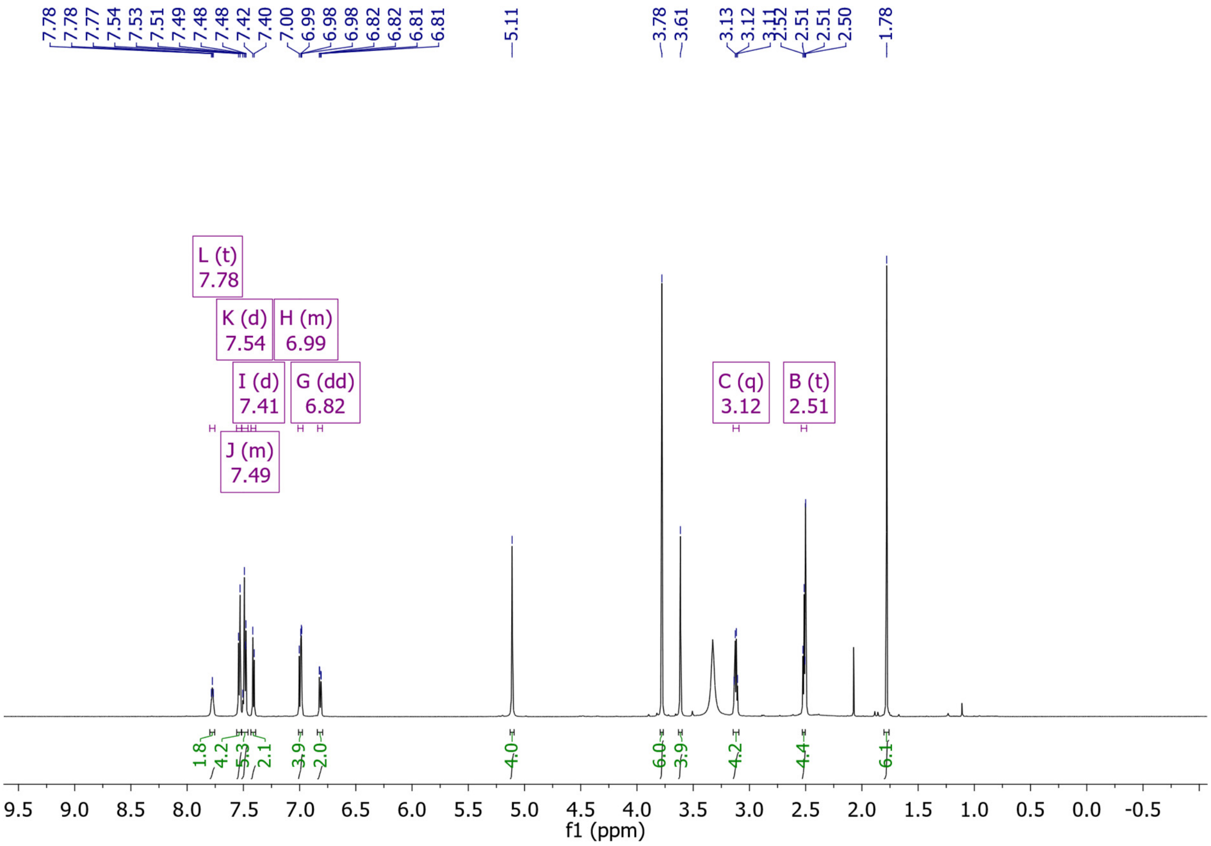

^{13}C NMR *(151 MHz, DMSO-d6)*

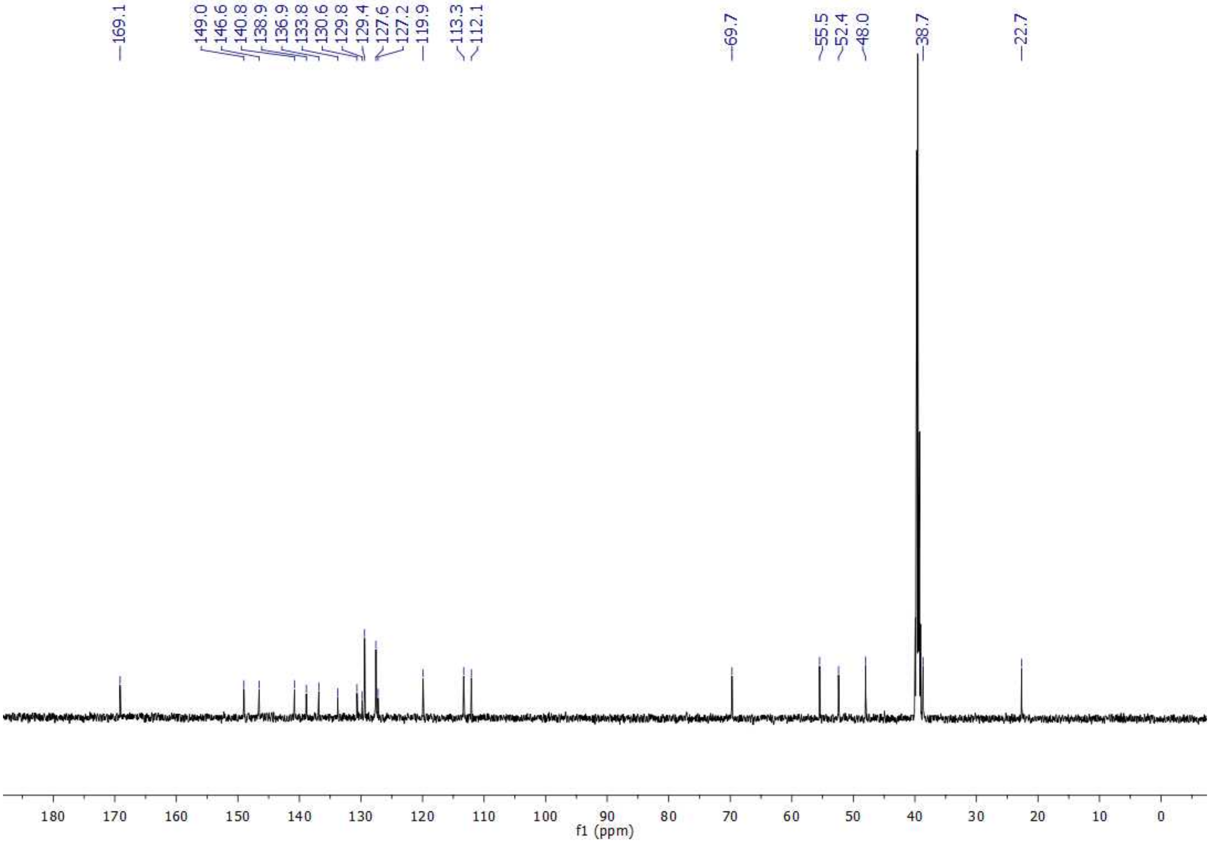

7n

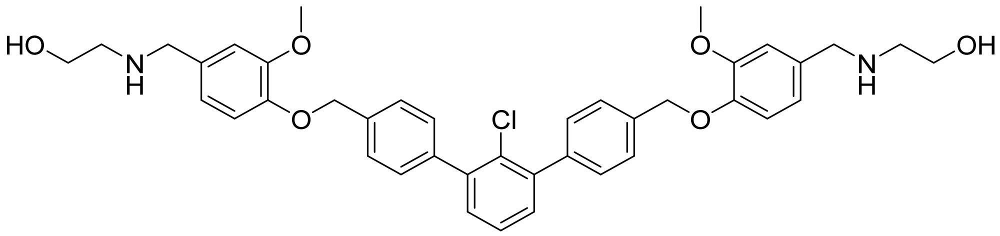

HO
N
H
O
O
Cl
O
O
N
H
OH

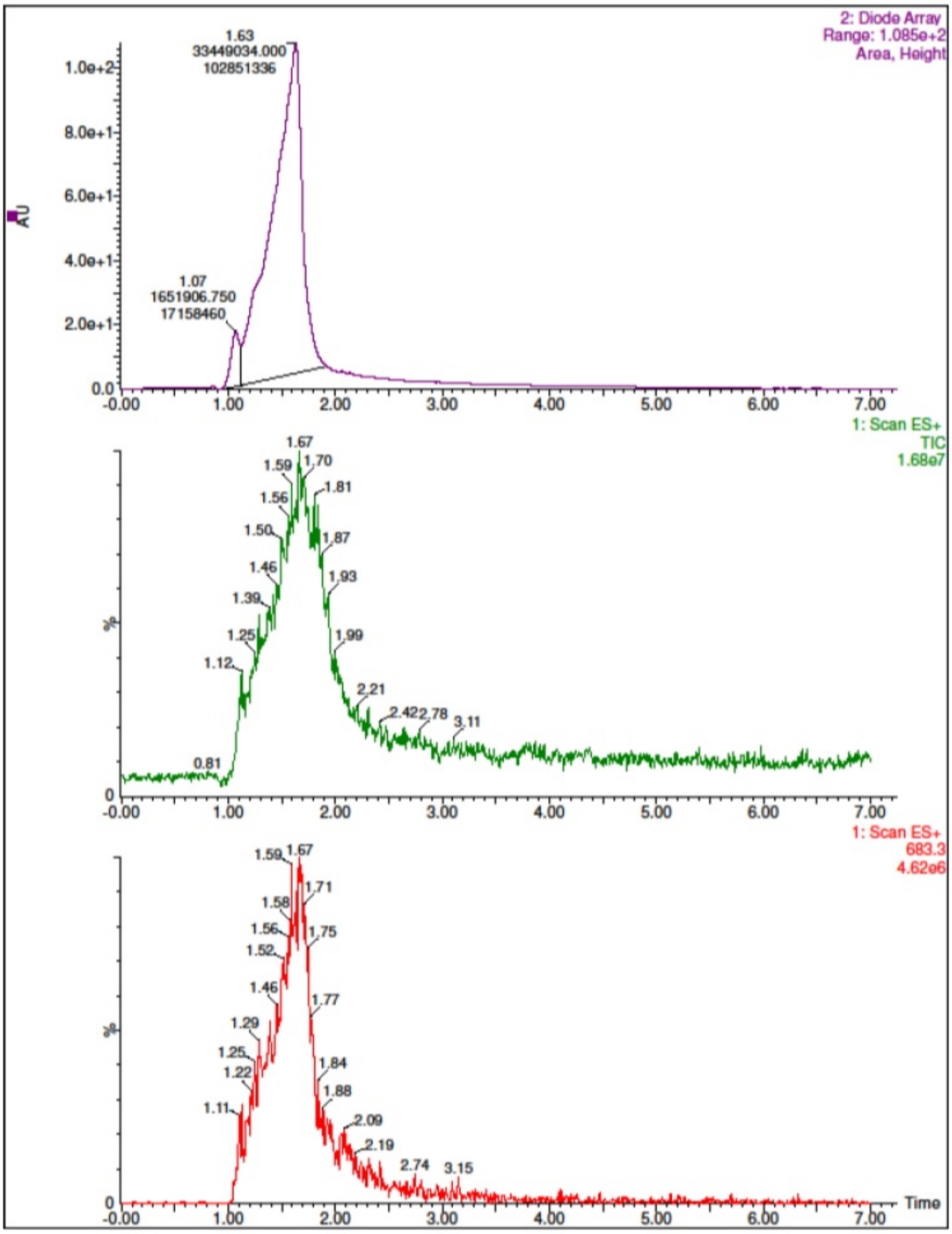

2: Diode Array
Range: 1.085e+2
Area, Height
1.63
33449034.000
102851336
1.07
1651906.750
17158460
AU
1: Scan ES+
TIC
1.68e7
1: Scan ES+
683.3
4.62e6
%
Time

***^{1}H NMR** (600 MHz, DMSO-d₆)*

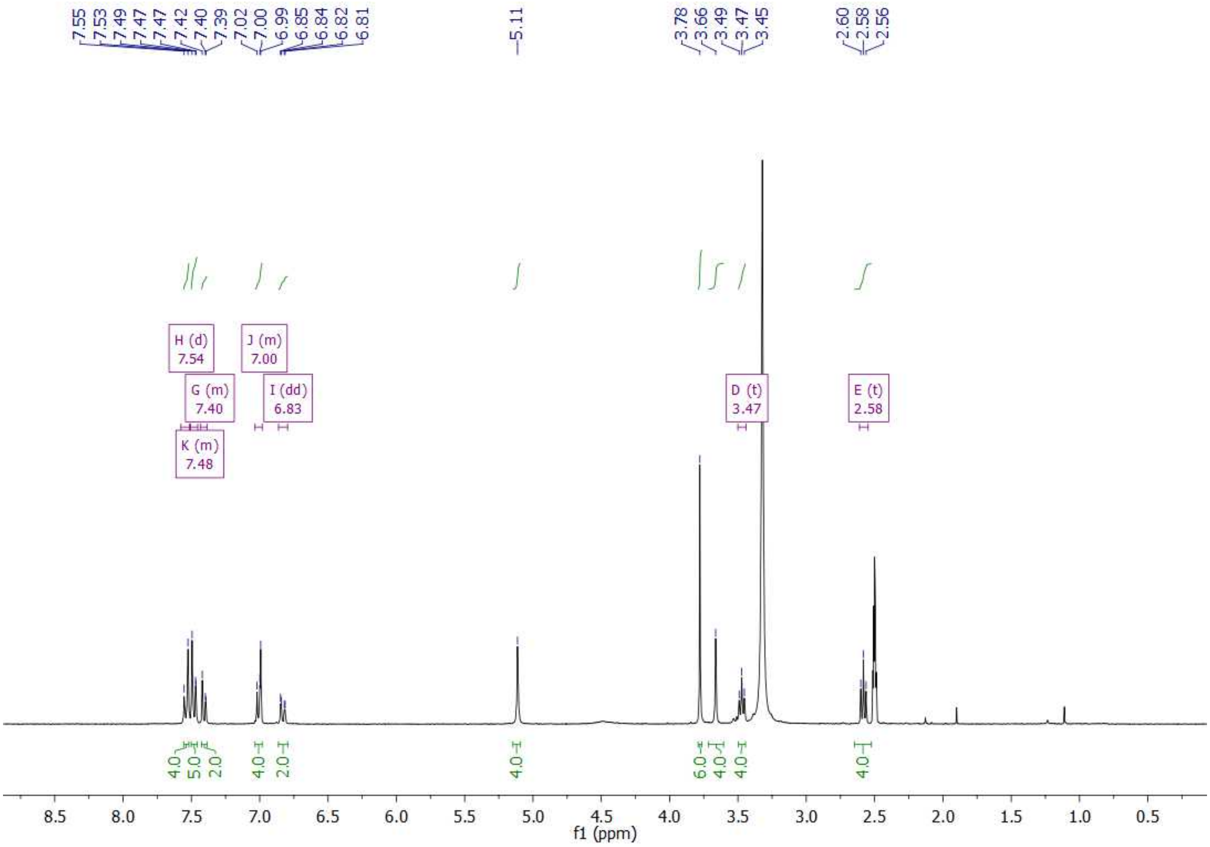

***^{13}C NMR** (151 MHz, DMSO-d₆)*

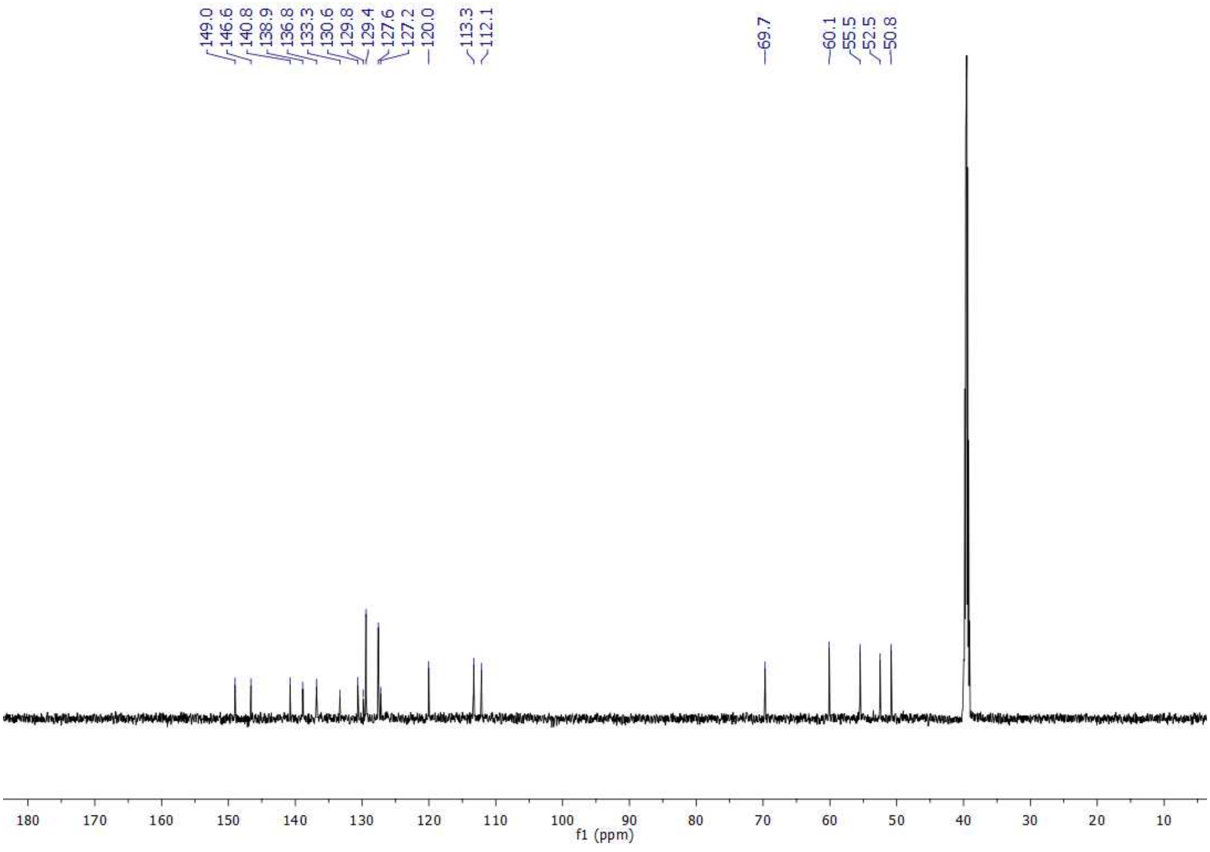

8a

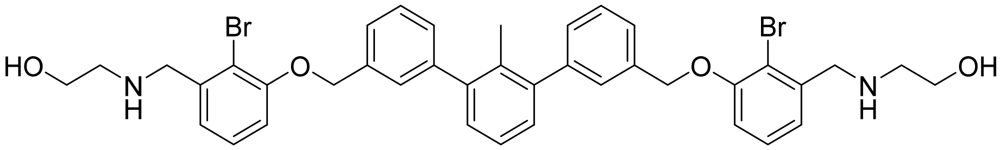
HO
N
H
Br
O
O
Br
N
H
OH

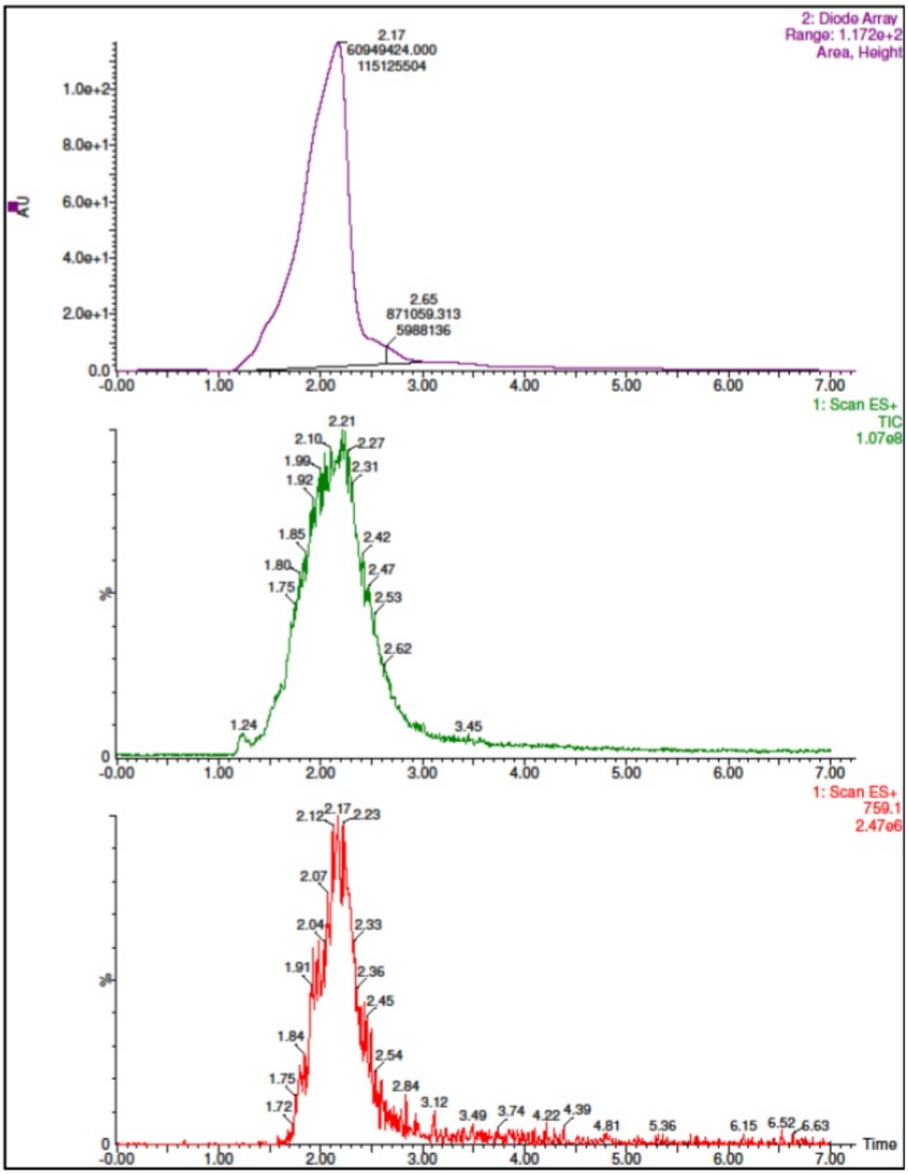
2: Diode Array
Range: 1.172e+2
Area, Height
2.17
60949424.000
115125504
2.65
871059.313
5988136
AU
1.0e+2
8.0e+1
6.0e+1
4.0e+1
2.0e+1
0.0
1: Scan ES+
TIC
1.07e8
1: Scan ES+
759.1
2.47e6
%
0
Time
-0.00
1.00
2.00
3.00
4.00
5.00
6.00
7.00

***¹H NMR** (400 MHz, CDCl₃)*

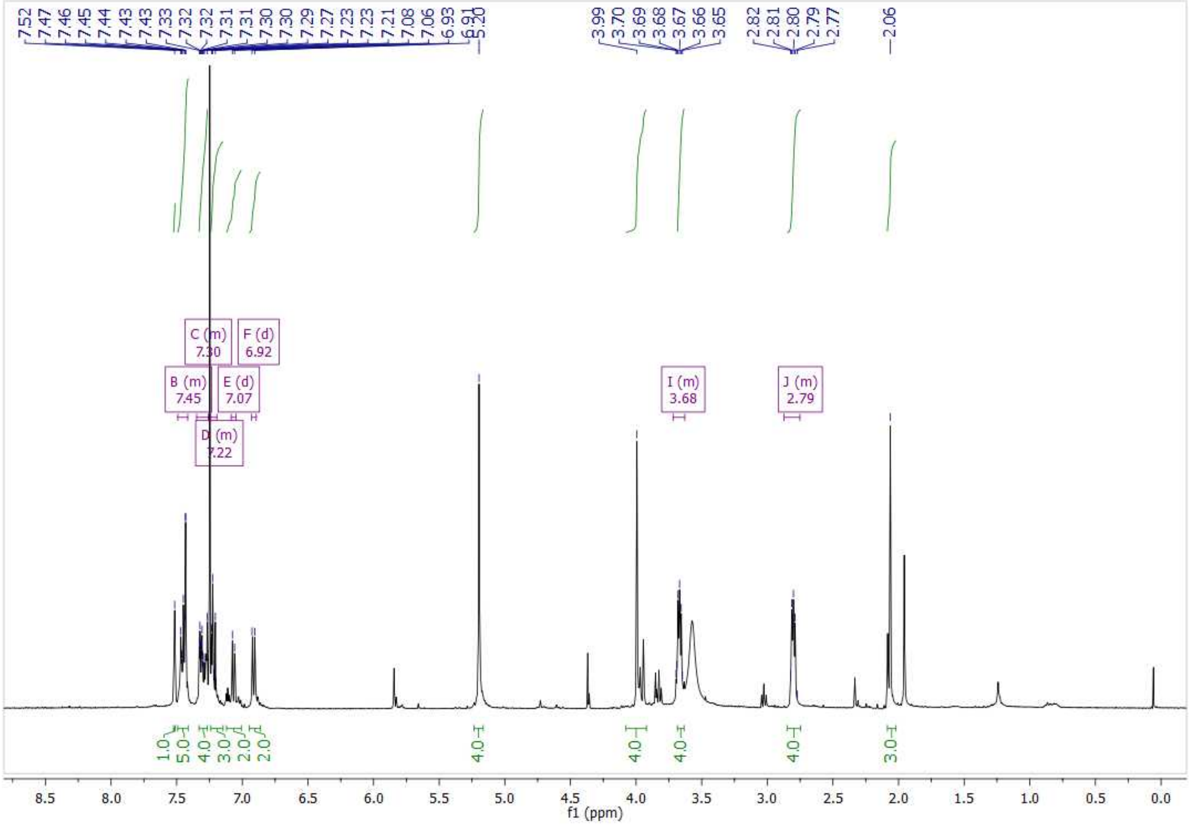

***¹³C NMR** (101 MHz, CDCl₃)*

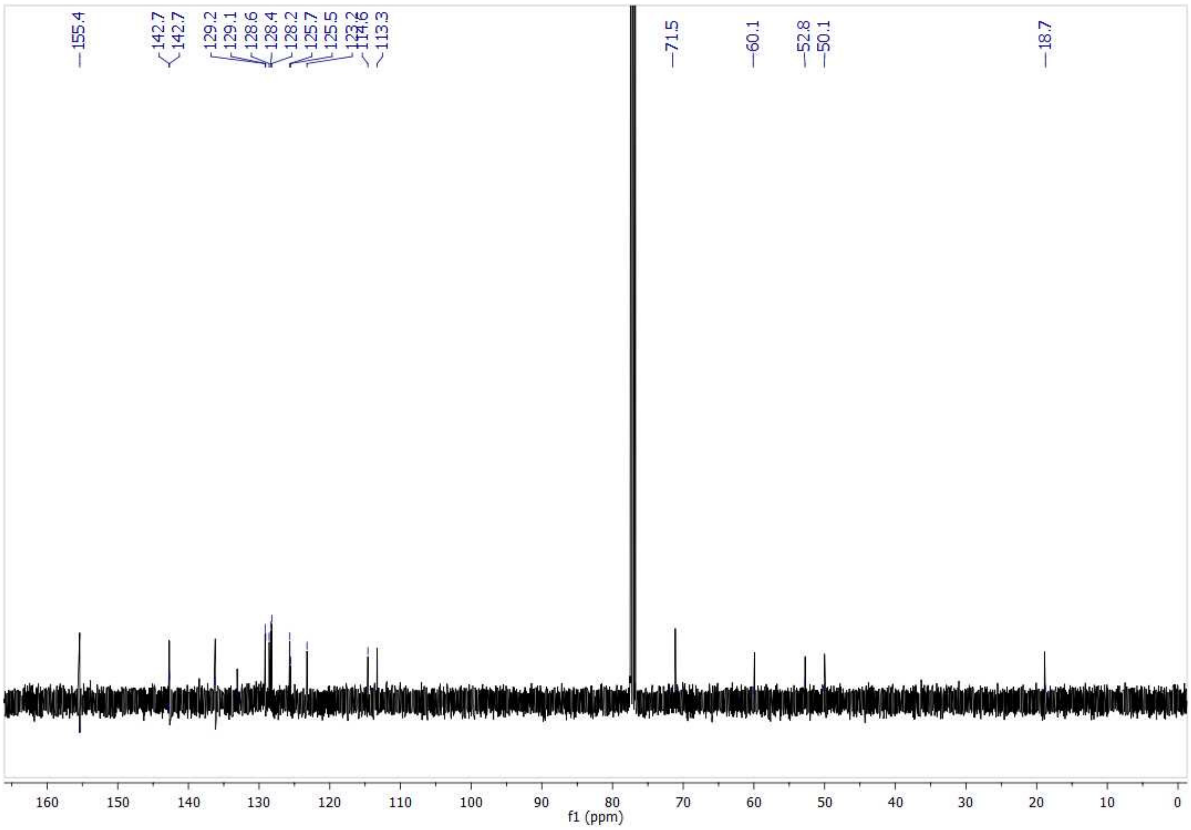

8b

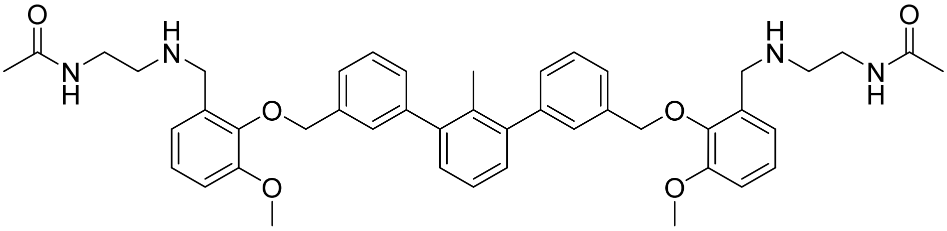
O
H
N
N
H
O
O
O
O
H
N
N
H
O

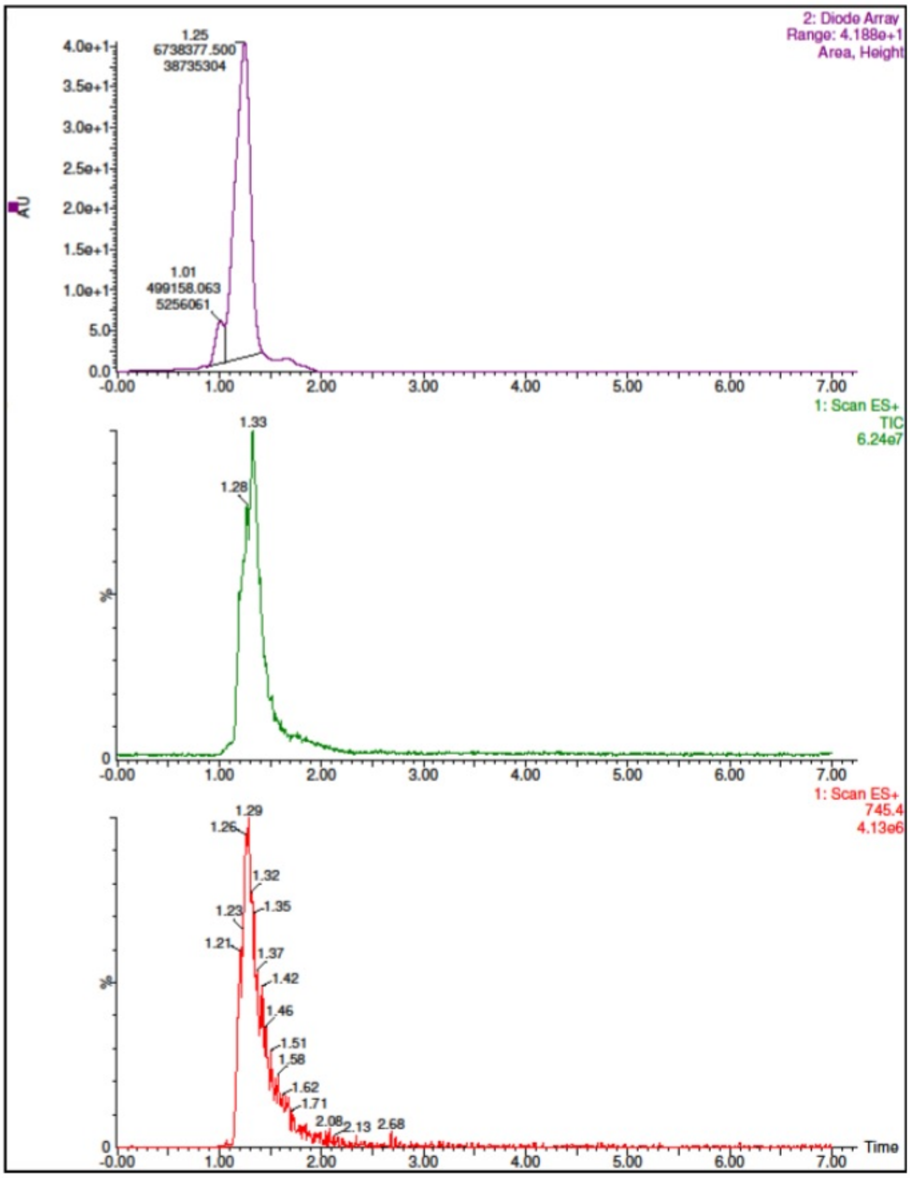
2: Diode Array
Range: 4.188e+1
Area, Height
1.25
6738377.500
38735304
1.01
499158.063
5256061
AU
4.0e+1
3.5e+1
3.0e+1
2.5e+1
2.0e+1
1.5e+1
1.0e+1
5.0
0.0
-0.00
1.00
2.00
3.00
4.00
5.00
6.00
7.00
1: Scan ES+
TIC
6.24e7
1.33
1.28
%
0
1: Scan ES+
745.4
4.13e6
1.29
1.26
1.32
1.23
1.35
1.21
1.37
1.42
1.46
1.51
1.58
1.62
1.71
2.08
2.13
2.68
Time

***¹H NMR** (600 MHz, CDCl₃)*

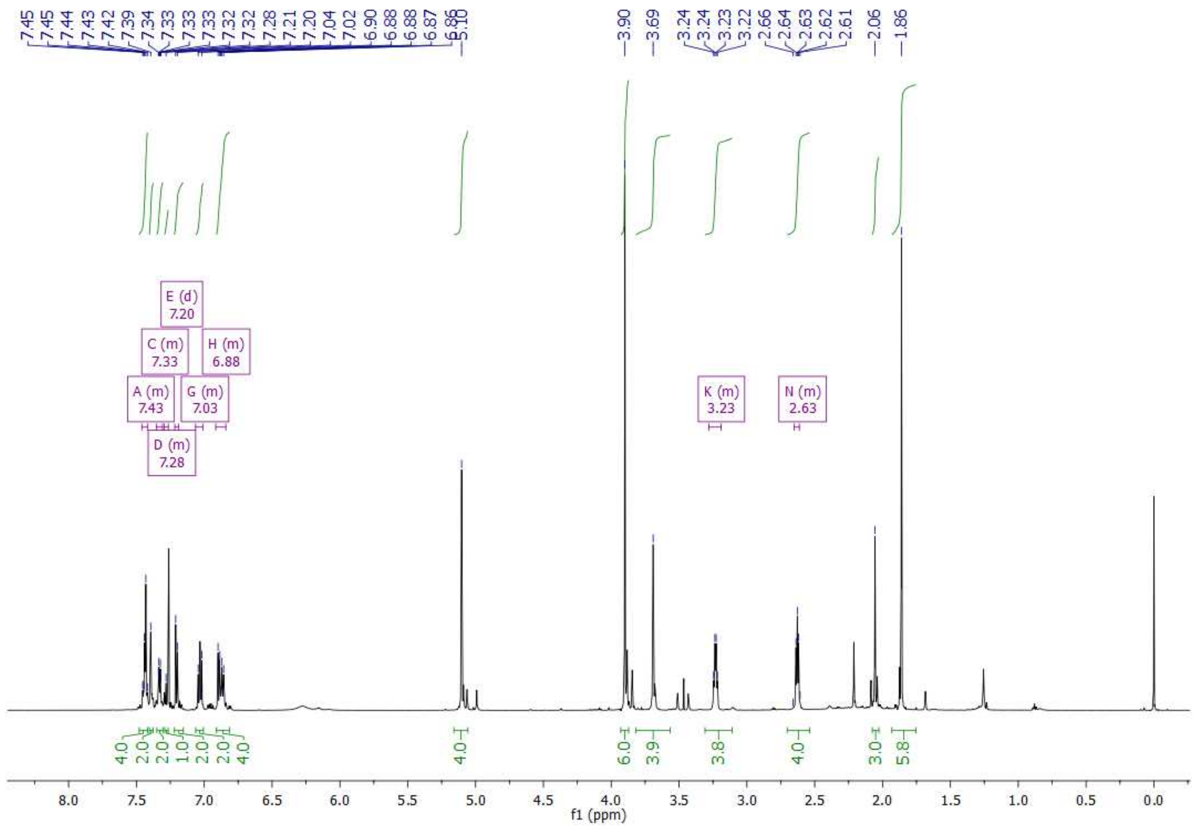

***¹³C NMR** (151 MHz, CDCl₃)*

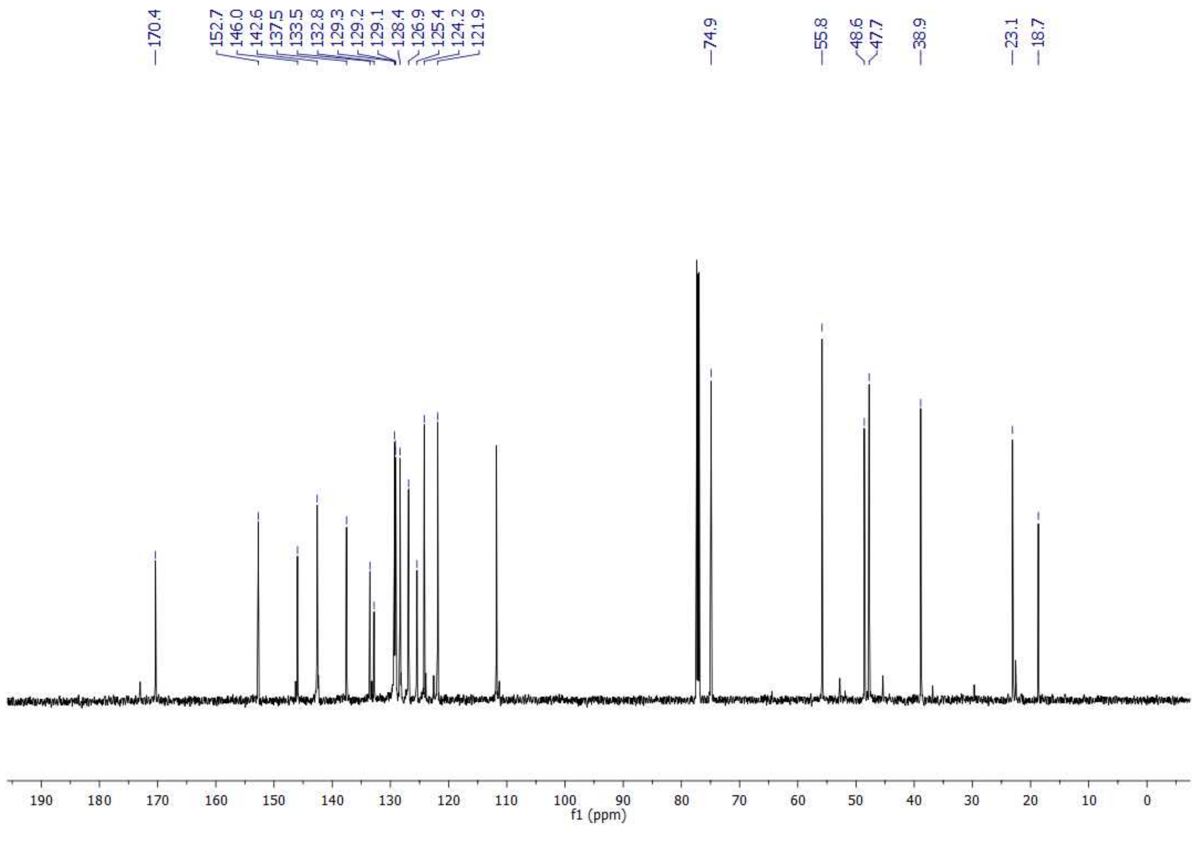

8c

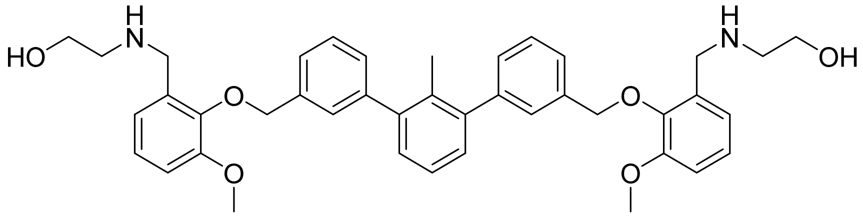
HO
H
N
O
O
H
N
OH
O
O

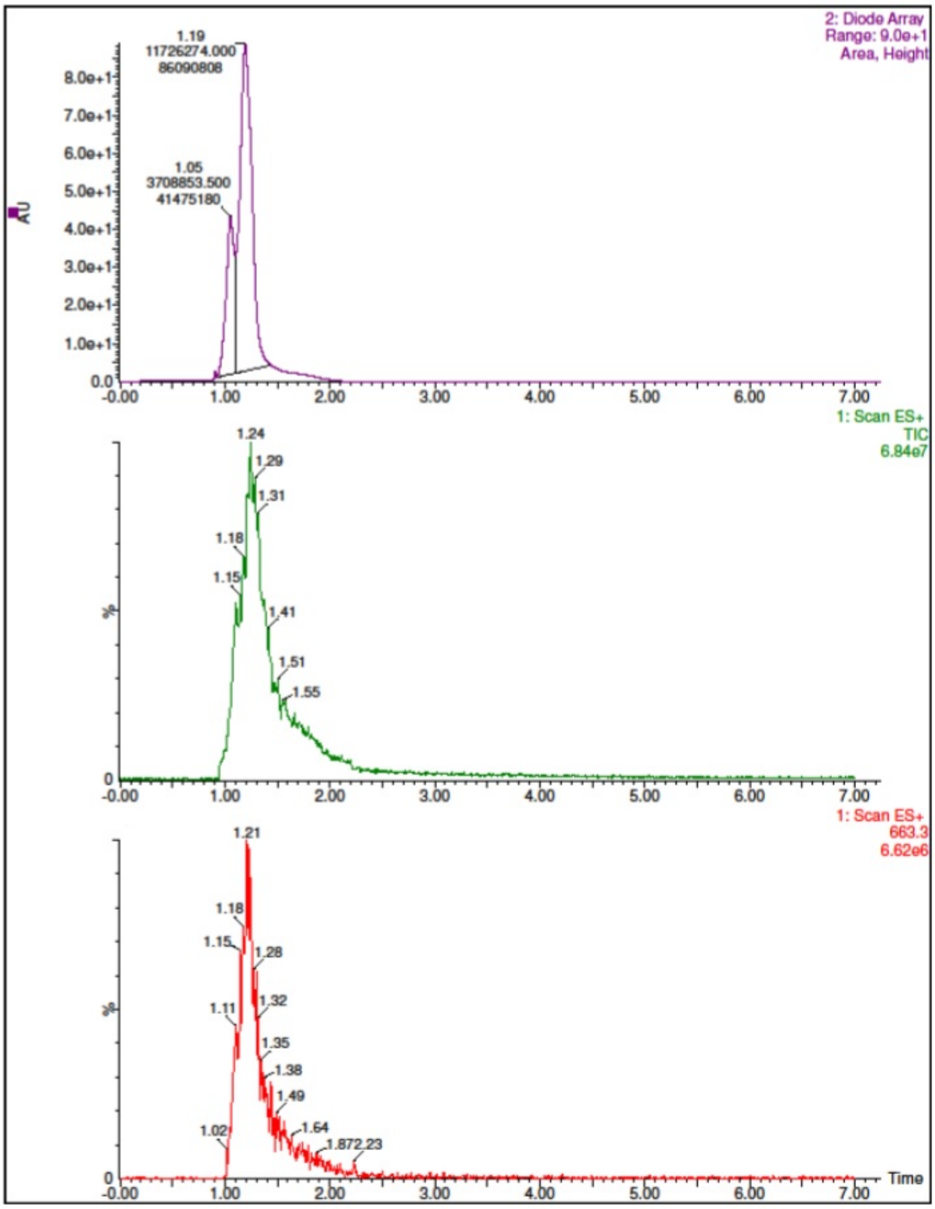
2: Diode Array
Range: 9.0e+1
Area, Height
1.19
11726274.000
86090808
1.05
3708853.500
41475180
AU
1: Scan ES+
TIC
6.84e7
1.24
1.29
1.31
1.18
1.15
1.41
1.51
1.55
%
1: Scan ES+
663.3
6.62e6
1.21
1.18
1.15
1.28
1.11
1.32
1.35
1.38
1.49
1.02
1.64
1.872.23
Time

***^{1}H NMR** (600 MHz, $CDCl_3$)*

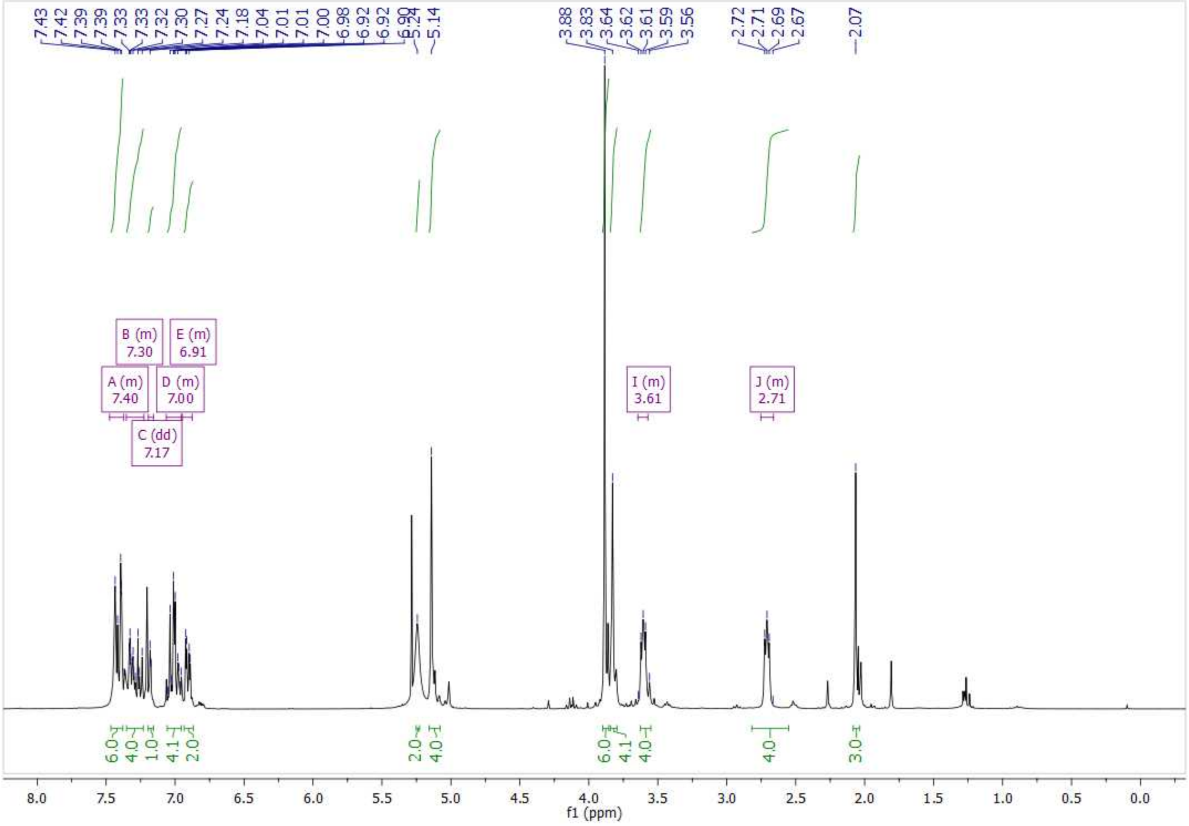

***^{13}C NMR** (151 MHz, $CDCl_3$)*

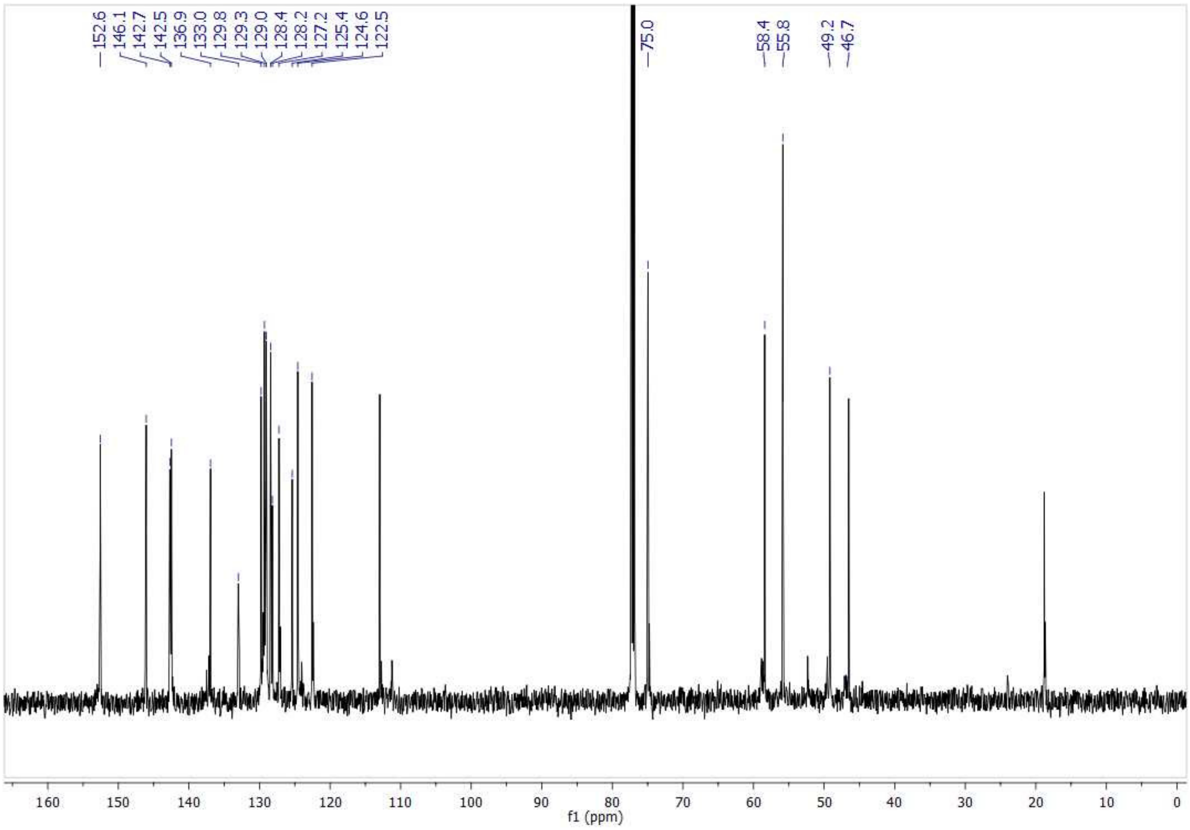

Supplement: Supplementary file 1 [file molecules-29-02646-s001.zip › molecules-3014359-supplementary.pdf]
